# Supplementary material for: CO2-promoted photocatalytic aryl migration from nitrogen to carbon for switchable transformation of N-arylpropiolamides
Source: Nat Commun. 2024 Nov 23;15:10153. doi: 10.1038/s41467-024-54239-5 (PMC11584665; doi:10.1038/s41467-024-54239-5)
Supplement: Supplementary file 1 — Supplementary Information [file 41467_2024_54239_MOESM1_ESM.pdf]

Supplementary Information

for

CO_2-Promoted Photocatalytic Aryl Migration from Nitrogen to Carbon for Switchable Transformation of *N*-arylpropiolamides

Ge Liu[1,2,3,4], Denghui Ma[2], Jianchen Zhang[1,3,4], Fanyuanhang Yang[1,3,4], Yuzhen Gao*[,1,3,4] and Weiping Su*[,1,3,4]

[1]State Key Laboratory of Structural Chemistry, Fujian Institute of Research on the Structure of Matter, Chinese Academy of Sciences, Fuzhou 350002, PR China

[2]School of New Energy, Ningbo University of Technology, Ningbo 315336, PR China

[3]Fujian Science & Technology Innovation Laboratory for Optoelectronic Information of China, Fuzhou, Fujian, PR China

[4]University of Chinese Academy of Sciences, Beijing 100049, PR China

*Corresponding authors: Yuzhen Gao (e-mail: gyz@fjirsm.ac.cn); Weiping Su (e-mail: wpsu@fjirsm.ac.cn)

Table of Contents

1. General Information S3

2. Experimental Section S3

2.1 General procedures for the reactions reported herein S3

2.2 Optimization of reaction conditions S5

2.3 Characterization of products S7

2.4 One-pot two-steps synthesis of *tetra*- and *tri*-substituted olefins S35

2.5 Gram-scale experiments S35

2.6 Unsuccessful substrates S36

2.7 General photoredox reaction setup S36

3. Mechanistic studies S37

3.1 Stern-Volmer fluorescence quenching experiments S37

3.2 Control experiments with radical scavengers S39

3.3 Reaction with radical clock S40

3.4 Desulfonylated exploration S40

3.5 *Z*/*E* isomerization of product **32** S41

3.6 Isotope-labelling experiments S41

3.7 Computational details S42

4. NMR Spectra of Compounds S49

5. X-Ray Crystallographic Spectrum S160

6. References S166

1. General Information

All reactions were conducted under an atmosphere of carbon dioxided. Unless otherwise noted, chemical reagents were purchased from commercial supplies (Accela, Acros Organics, Adamas-beta®, Alfa Aesar, Aladdin, Bidepharmatech Energy Chemical, TCI Chemicals, Innochem, J&K Chemicals, Laajoo, Leyan, Sigma-Aldrich, Sinocompound, and 3A Chemicals) and used directly without further purification. Flash chromatography was performed with Sepaflash columns produced by Santai Technologies. Purification of products was performed by flash chromatography (FC) using silica gel or preparative thin layer chromatography. ^{1}H and ^{13}C NMR spectra were recorded on a Bruker AVANCE III spectrometer (400 MHz and 101 MHz, respectively). The following abbreviations (or combinations thereof) were used to explain multiplicities: s = singlet, d = doublet, t = triplet, q = quartet, dd = doublet of doublet, td = triplet of doublet and m = multiplet. High-resolution mass spectra (HRMS) were obtained on an Impact II UHR-TOF mass spectrometry equipped with an ESI source from Bruker at Fujian Institute of Research on the Structure of Matter. The Blue LED strips (1 meter, 40 W and 72 W) were purchased from GeAo Chemical (China). CO_2 gas (Purity: 99.995%) was purchased from Linde. The staring materials **1** were synthesized following the known procedures[1], and all the products were identified by ^{1}H NMR, ^{13}C NMR and HRMS.

2. Experimental Section

2.1 General procedures for the reactions reported herein

(a) The preparation of *tetra*-substituted alkenyl amides (standard conditions A)

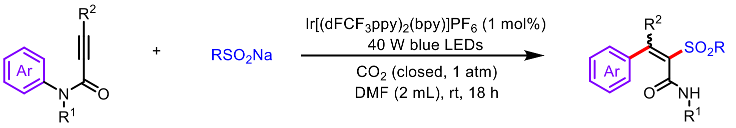

1 **2** **3**, **5-63**

The oven-dried Schlenk tube (38 mL) containing a stirring bar was charged with **1** (0.1 mmol), RSO_2Na (0.15 mmol, 1.5 equiv), Ir[(dFCF$_3$ppy)$_2$(bpy)]PF$_6$ (0.001 mmol, 1 mmol%) and DMF (2 mL). The tube was then evacuated and back-filled with CO_2 for 3 times. The mixture was placed under a 40 W blue LED (λ_{max} = 465 nm, 1.0 cm away from the LEDs, with cooling fan to keep the reaction temperature at 25~30 ^{o}C) light source and stirred at ambient temperature for 18 h. Upon completion of the reaction, all the solvents were removed under reduced pressure at high temperature. The crude reaction mixture was diluted with EtOAc (5 mL) and filtered through a short pad of Celite. The sealed tube and Celite pad were washed with an additional 25 mL of EtOAc. The filtrate was concentrated in vacuo, and crude ^{1}H NMR spectrum was taken using $CHCl_2CHCl_2$ as internal standard. The resulting residue was purified by flash silica gel chromatography or preparative thin layer chromatography using petroleum ether/EtOAc (4:1-1:1) as the eluent to give the desired products.

(b) The preparation of *tri*-substituted alkenyl amides (standard conditions B)

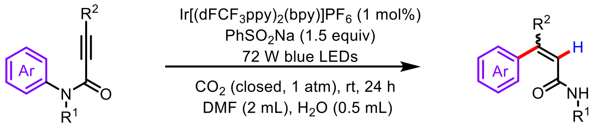

1 **4**, **64-90**

The oven-dried Schlenk tube (38 mL) containing a stirring bar was charged with **1** (0.1 mmol), $PhSO_2Na$ (0.15 mmol, 1.5 equiv), Ir[(dFCF$_3$ppy)$_2$(bpy)]PF$_6$ (0.001 mmol, 1 mmol%), DMF (2 mL) and H_2O (0.5 mL). The

tube was then evacuated and back-filled with CO_2 for 3 times. The mixture was placed under a 72 W blue LED (λ_{max} = 465 nm, 1.0 cm away from the LEDs, with cooling fan to keep the reaction temperature at 25~30 ^oC) light source and stirred at ambient temperature for 24 h. Upon completion of the reaction, all the solvents were removed under reduced pressure at high temperature. The crude reaction mixture was diluted with EtOAc (5 mL) and filtered through a short pad of Celite. The sealed tube and Celite pad were washed with an additional 25 mL of EtOAc. The filtrate was concentrated in vacuo, and crude ^1H NMR spectrum was taken using $CHCl_2CHCl_2$ as internal standard. The resulting residue was purified by flash silica gel chromatography or preparative thin layer chromatography using petroleum ether/EtOAc (4:1-1:1) as the eluent to give the desired products.

(c) The preparation of CF_3-containing *tetra*-substituted alkenyl amides (standard conditions C)

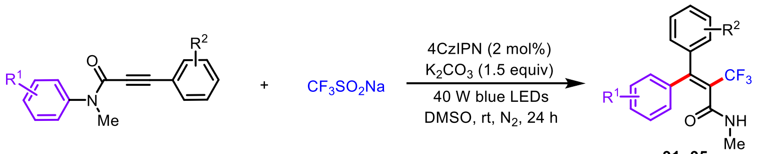

The oven-dried Schlenk tube (38 mL) containing a stirring bar was charged with **1** (0.2 mmol), CF_3SO_2Na (0.3 mmol, 1.5 equiv), 4 CzIPN (2 mmol%), DMSO (2 mL). The tube was then evacuated and back-filled with N_2 for 3 times. The mixture was placed under a 40 W blue LED (λ_{max} = 465 nm, 1.0 cm away from the LEDs, with cooling fan to keep the reaction temperature at 25~30 ^oC) light source and stirred at ambient temperature for 24 h. Upon completion of the reaction, all the solvents were removed under reduced pressure at high temperature. The crude reaction mixture was diluted with EtOAc (5 mL) and filtered through a short pad of Celite. The sealed tube and Celite pad were washed with an additional 25 mL of EtOAc. The filtrate was concentrated in vacuo, and crude ^1H NMR spectrum was taken using $CHCl_2CHCl_2$ as internal standard. The resulting residue was purified by flash silica gel chromatography or preparative thin layer chromatography using petroleum ether/EtOAc (5:1) as the eluent to give the desired products.

(d) Photocatalytic isomerization of the *tetra*- and *tri*-substituted alkenyl amides

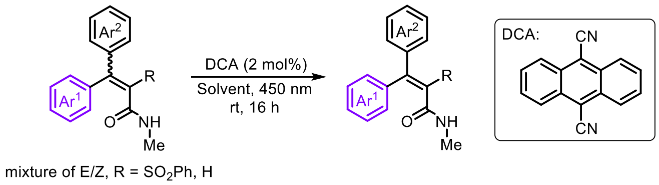

The oven-dried Schlenk tube (38 mL) containing a stirring bar was charged with tri-substituted alkenyl amides (0.05 mmol), DCA (0.001 mmol, 2 mmol%), EA (for **72**, **74**, **79**, **82**, **83**, **86**, **87**, **89**) or DCM (for **32**, **38**) (2 mL). The tube was then evacuated and back-filled with N_2 for 3 times. The mixture was placed under a 40 W blue LED (λ_{max} = 465 nm, 1.0 cm away from the LEDs, with cooling fan to keep the reaction temperature at 25~30 ^oC) light source and stirred at ambient temperature for 16 h. Upon completion of the reaction, all the solvents were removed under reduced pressure. The crude reaction mixture was diluted with EtOAc (5 mL) and filtered through a short pad of Celite. The sealed tube and Celite pad were washed with an additional 25 mL of EtOAc. The filtrate was concentrated in vacuo, and crude ^1H NMR spectrum was taken using $CHCl_2CHCl_2$ as internal standard. The resulting residue was purified by flash silica gel chromatography or preparative thin layer chromatography using petroleum ether/EtOAc (10:1-2:1) as the eluent to give the desired products. *The ^1H NMR spectra of these products were provided in the "NMR Spectra of Compounds" part.*

2.2 Optimization of reaction conditions

Table S1: Screening of the Photocatalysts[a]

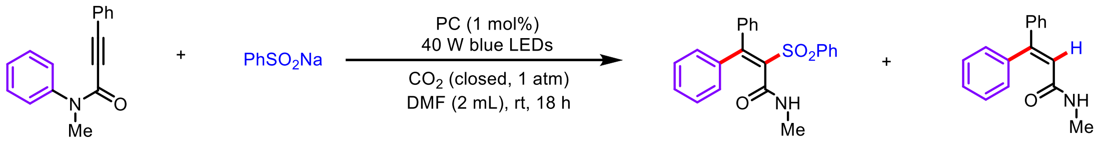

Entry	[PC] (1 mol%)	Yield of **3** (%)	Yield of **4** (%)
1	4CzIPN	85	trace
2	EosinY[b]	60	14
3	$Ru(bpy)_3Cl_2$	79	7
4	*fac*-$Ir(ppy)_3$	65	13
5	$[Ir(ppy)_2(dtbbpy)]PF_6$	47	27
6	$[Ir(dMppy)_2(dtbbpy)]PF_6$	41	30
7	$[Ir(dFCF_3ppy)_2(bpy)]PF_6$	95	0

[a]Reaction conditions: **1a** (0.1 mmol), **2a** (0.15 mmol, 1.5 equiv), PC (1 mol %), DMF (2 mL), 40 W blue LEDs, rt, 18 h in CO_2 (closed, 1 atm) atmosphere; [b]PC (3 mol %); Yields were determined by ^1H NMR with $CHCl_2CHCl_2$ as internal standard. 4CzIPN: 2,4,5,6-tetra(9*H*-carbazol-9-yl)isophthalonitrile; bpy: 2,2'-bipyridine; ppy: 2-phenylpyridine; dtbbpy: 4,4-di-*tert*-butyl-2,2'-bipyridine; dMppy: 5-methyl-2-(*p*-tolyl)pyridine; $dFCF_3ppy$: 2-(2,4-difluorophenyl)-5-(trifluoromethyl)pyridine.

Table S2: Screening of the Solvents[a]

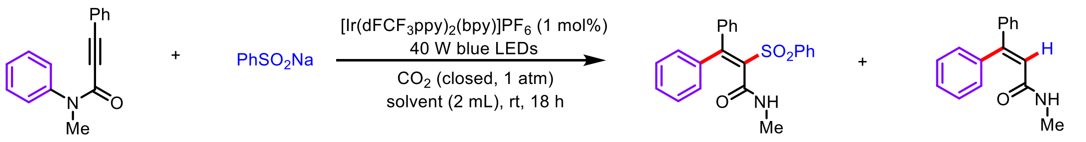

Entry	Solvents	Yield of **3** (%)	Yield of **4** (%)
1	DMA	85	0
2	DMSO	86	0
3	NMP	57	0
4	Toluene	0	0
5	THF	26	0
6	EA	39	0
7	CH_3CN	80	3
8	Acetone	76	13
9	CH_3CH_2OH	20	50
10	DMF	95	0
11	DMF : H_2O = 1 :1	0	29
12	DMF : H_2O = 3 :1	0	69

[a]Reaction conditions: **1a** (0.1 mmol), **2a** (0.15 mmol, 1.5 equiv), PC (1 mol %), Solvent (2 mL), 40 W blue LEDs, rt, 18 h in CO_2 (closed, 1 atm) atmosphere; Yields were determined by ^1H NMR with $CHCl_2CHCl_2$ as internal standard.

Table S3: Screening of the Reaction Time[a]

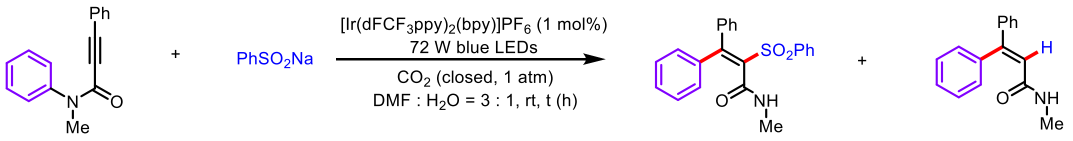

1a **2a** **3** **4**

Entry	Reaction time (h)	Yield of **3** (%)	Yield of **4** (%)
1	18	0	73
2	24	0	78
3	48	0	68

[a]Reaction conditions: **1a** (0.1 mmol), **2a** (0.15 mmol, 1.5 equiv), PC (1 mol %), DMF (1.5 mL), H_2O (0.5 mL), 72 W blue LEDs, rt, t (h) in CO_2 (closed, 1 atm) atmosphere; Yields were determined by ^{1}H NMR with $CHCl_2CHCl_2$ as internal standard.

Table S4: Screening the Equiv of $PhSO_2Na$[a]

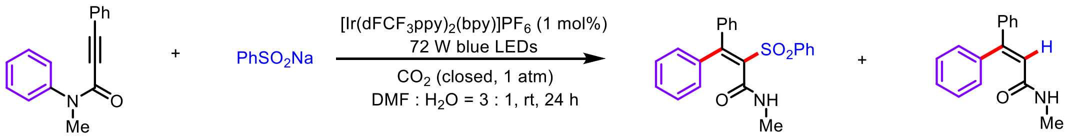

1a **2a** **3** **4**

Entry	$PhSO_2Na$ (x equiv)	Yield of **3** (%)	Yield of **4** (%)
1	0.2	15	3
2	0.5	40	5
3	1.0	25	51
4	1.5	0	78
5	2.0	0	75

[a]Reaction conditions: **1a** (0.1 mmol), **2a** (x equiv), PC (1 mol %), DMF (1.5 mL), H_2O (0.5 mL), 72 W blue LEDs, rt, 24 h in CO_2 (closed, 1 atm) atmosphere; Yields were determined by ^{1}H NMR with $CHCl_2CHCl_2$ as internal standard.

Table S5: Screening the Amount of H_2O[a]

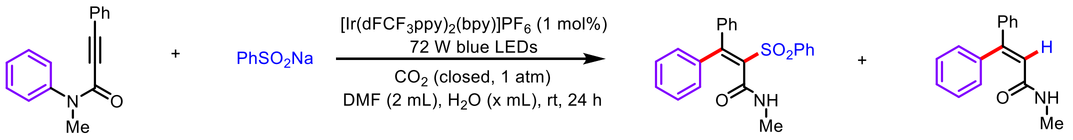

1a **2a** **3** **4**

Entry	H_2O (x mL)	Yield of **3** (%)	Yield of **4** (%)
1	0.1	17	70
2	0.3	7	76
3	0.5	0	**85**
4	1.0	18	64

[a]Reaction conditions: **1a** (0.1 mmol), **2a** (1.5 equiv), PC (1 mol %), DMF (2.0 mL), H_2O (x mL), 72 W blue LEDs, rt, 24 h in CO_2 (closed, 1 atm) atmosphere; Yields were determined by ^{1}H NMR with $CHCl_2CHCl_2$ as internal standard.

Table S6: Screening of the Sulfinates[a]

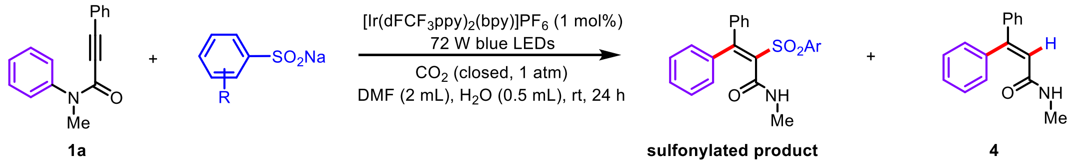

Entry	sulfinates	Yield of sulfonylated product (%)	Yield of **4** (%)
1	$PhSO_2Na$	0	85
2	4-Me$PhSO_2Na$	0	80
3	2-OMe$PhSO_2Na$	76	0
4	4-CF_3PhSO_2Na	0	64
5	4-Br$PhSO_2Na$	24	0
6	3-CF_3PhSO_2Na	12	67
7	3-Br$PhSO_2Na$	0	61
8	2-F$PhSO_2Na$	10	65

[a]Reaction conditions: **1a** (0.1 mmol), sulfinate (1.5 equiv), PC (1 mol %), DMF (2.0 mL), H_2O (0.5 mL), 72 W blue LEDs, rt, 24 h in CO_2 (closed, 1 atm) atmosphere; Yields were determined by ^{1}H NMR with $CHCl_2CHCl_2$ as internal standard.

2.3 Characterization of products

***N*-methyl-3,3-diphenyl-2-(phenylsulfonyl)acrylamide (3)**

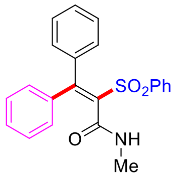

Standard conditions A was followed. White solid. Yield: 35.8 mg, 95%.

^{1}H NMR (400 MHz, $CDCl_3$) δ 7.64 (d, *J* = 7.8 Hz, 2H), 7.51 (t, *J* = 7.5 Hz, 1H), 7.36 (t, *J* = 8.1 Hz, 2H), 7.25 (d, *J* = 7.7 Hz, 8H), 7.05 (d, *J* = 7.6 Hz, 2H), 6.18 (d, *J* = 5.4 Hz, 1H), 2.69 (d, *J* = 4.8 Hz, 3H). **^{13}C NMR (101 MHz, $CDCl_3$)** δ 163.90, 154.95, 141.07, 139.59, 139.23, 136.78, 133.21, 129.53, 129.10, 128.56, 128.34, 128.14, 127.66, 26.95. **HRMS (m/z, ESI$^+$):** Calcd for $C_{22}H_{19}NO_3SNa^+$ [M+Na$^+$] 400.0978, found 400.0982.

***N*-methyl-3,3-diphenylacrylamide (4)**[2]

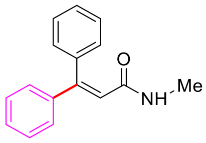

Standard conditions B was followed. White solid. Yield: 18.0 mg, 76%.

^{1}H NMR (400 MHz, $CDCl_3$) δ 7.40 (h, *J* = 2.3 Hz, 3H), 7.35 – 7.28 (m, 3H), 7.26 (ddt, *J* = 6.8, 4.6, 2.1 Hz, 4H), 6.37 (s, 1H), 5.23 (s, 1H), 2.63 (d, *J* = 4.2 Hz, 3H). **^{13}C NMR (101 MHz, $CDCl_3$)** δ 167.36, 149.52, 140.83, 138.50, 129.35, 128.91, 128.63, 128.59, 128.40, 128.01, 122.54, 26.24.

***N*-ethyl-3,3-diphenyl-2-(phenylsulfonyl)acrylamide (5)**

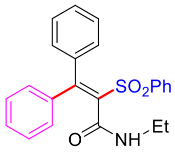

Standard conditions A was followed. Yellowish solid. Yield: 64.5 mg, 82%.

^{1}H NMR (400 MHz, CDCl$_3$) δ 7.68 (d, *J* = 7.7 Hz, 2H), 7.50 (t, *J* = 7.4 Hz, 1H), 7.36 (t, *J* = 7.8 Hz, 2H), 7.31 – 7.21 (m, 8H), 7.10 (d, *J* = 7.0 Hz, 2H), 6.35 (t, *J* = 6.0 Hz, 1H), 3.17 (p, *J* = 7.1 Hz, 2H), 0.76 (t, *J* = 7.2 Hz, 3H). **^{13}C NMR (101 MHz, CDCl$_3$)** δ 162.99, 154.96, 141.26, 139.91, 139.36, 136.84, 133.20, 129.39, 129.29, 128.98, 128.67, 128.55, 128.48, 128.41, 128.29, 128.23, 128.18, 127.96, 127.65, 122.93, 34.97, 34.14, 14.24, 13.84. **HRMS (m/z, ESI$^+$):** Calcd for $C_{23}H_{21}NO_3S\ Na^+$ [M+Na$^+$] 414.1134, found 414.1136.

***N*-allyl-3,3-diphenyl-2-(phenylsulfonyl)acrylamide (6)**

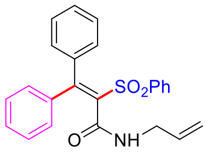

Standard conditions A was followed. Yellowish solid. Yield: 17.0 mg, 42%.

^{1}H NMR (400 MHz, CDCl$_3$) δ 7.64 (d, *J* = 7.8 Hz, 2H), 7.50 (t, *J* = 7.4 Hz, 1H), 7.38 – 7.24 (m, 10H), 7.05 (d, *J* = 7.5 Hz, 2H), 6.11 (t, *J* = 6.0 Hz, 1H), 5.50 – 5.43 (m, 1H), 4.90 (dd, *J* = 26.2, 13.7 Hz, 2H), 3.78 (t, *J* = 5.9 Hz, 2H).**^{13}C NMR (101 MHz, CDCl$_3$)** δ 163.04, 155.05, 141.04, 139.71, 139.27, 136.72, 133.21, 132.93, 129.52, 129.06, 128.63, 128.55, 128.42, 128.39, 128.18, 127.68, 116.86, 42.34. **HRMS (m/z, ESI$^+$):** Calcd for $C_{24}H_{21}NO_3S\ Na^+$ [M+Na$^+$] 426.1134, found 426.1138.

***N*-benzyl-3,3-diphenyl-2-(phenylsulfonyl)acrylamide (7)**

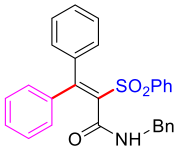

Standard conditions A was followed. White solid. Yield: 28.2 mg, 62%.

^{1}H NMR (400 MHz, CDCl$_3$) δ 7.62 (d, *J* = 7.9 Hz, 2H), 7.50 (t, *J* = 7.4 Hz, 1H), 7.37 – 7.15 (m, 13H), 7.03 (d, *J* = 7.5 Hz, 2H), 6.83 (d, *J* = 7.1 Hz, 2H), 6.37 (t, *J* = 5.9 Hz, 1H), 4.36 (d, *J* = 5.8 Hz, 2H). **^{13}C NMR (101 MHz, CDCl$_3$)** δ 163.03, 155.17, 140.97, 139.64, 139.22, 136.98, 136.72, 133.22, 129.47, 129.02, 128.63, 128.59, 128.57, 128.48, 128.39, 128.16, 127.77, 127.69, 127.41, 44.10. **HRMS (m/z, ESI$^+$):** Calcd for $C_{28}H_{23}NO_3S\ Na^+$ [M+Na$^+$] 476.1291, found 476.1291.

3,3-diphenyl-*N*-(1-phenylethyl)-2-(phenylsulfonyl)acrylamide (8)

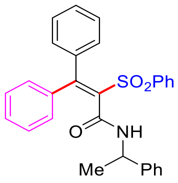

Standard conditions A was followed. White solid. Yield: 24.0 mg, 51%.

^{1}H NMR (400 MHz, CDCl$_3$) δ 7.64 (d, *J* = 7.8 Hz, 2H), 7.50 (t, *J* = 7.5 Hz, 1H), 7.37 – 7.14 (m, 13H), 7.06 (d, *J* = 7.5 Hz, 2H), 6.98 (m, 2H), 6.12 (d, *J* = 8.1 Hz, 1H), 5.01 (p, *J* = 7.1 Hz, 1H), 1.28 (d, *J* = 7.0 Hz, 3H). **^{13}C NMR (101 MHz, CDCl$_3$)** δ 162.19, 154.94, 141.75, 140.98, 139.70, 139.23, 136.78, 133.23, 129.41, 129.00, 128.65, 128.60, 128.55, 128.44, 128.31, 128.23, 127.69, 127.30, 126.26, 49.27, 20.51. **HRMS (m/z, ESI$^+$):** Calcd for

$C_{29}H_{25}NO_3SNa^+$ [M+Na$^+$] 490.1447, found 490.1448.

3,3-diphenyl-2-(phenylsulfonyl)acrylamide (9)

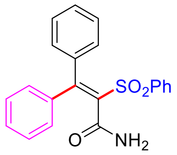

Standard conditions A was followed. White solid. Yield: 70.4 mg, 97%.

^{1}H NMR (400 MHz, CDCl$_3$) δ 8.50 (s, 1H), 7.93 (d, *J* = 7.6 Hz, 3H), 7.57 (dd, *J* = 7.9, 3.7 Hz, 3H), 7.49 (dd, *J* = 14.4, 7.7 Hz, 4H), 7.37 – 7.26 (m, 5H), 7.11 (t, *J* = 7.4 Hz, 1H). **^{13}C NMR (101 MHz, CDCl$_3$)** δ 160.22, 142.16, 139.09, 137.20, 136.64, 134.02, 131.58, 131.38, 130.49, 129.34, 129.14, 129.06, 128.38, 125.25, 120.31. **HRMS (m/z, ESI$^+$):** Calcd for $C_{21}H_{17}NO_3SNa^+$ [M+Na$^+$] 386.0821, found 386.0821.

***N*,3,3-triphenyl-2-(phenylsulfonyl)acrylamide (10)**

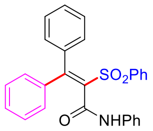

Standard conditions A was followed. White solid. Yield: 37.2 mg, 85%.

^{1}H NMR (400 MHz, CDCl$_3$) δ 8.04 (s, 1H), 7.71 (d, *J* = 7.8 Hz, 2H), 7.54 (t, *J* = 7.4 Hz, 1H), 7.40 (t, *J* = 7.7 Hz, 2H), 7.35 – 7.18 (m, 12H), 7.14 (d, *J* = 7.4 Hz, 2H), 7.09 (t, *J* = 7.3 Hz, 1H). **^{13}C NMR (101 MHz, CDCl$_3$)** δ 161.41, 156.16, 141.09, 139.46, 139.24, 137.19, 136.96, 133.53, 129.77, 129.02, 128.95, 128.81, 128.78, 128.58, 128.35, 128.31, 127.85, 125.14, 120.68. **HRMS (m/z, ESI$^+$):** Calcd for $C_{27}H_{21}NO_3SNa^+$ [M+Na$^+$] 462.1134, found 462.1136.

***N*-(4-fluorophenyl)-3,3-diphenyl-2-(phenylsulfonyl)acrylamide (11a)**
3-(4-fluorophenyl)-*N*,3-diphenyl-2-(phenylsulfonyl)acrylamide (11b)

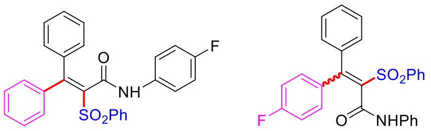

11a **11b**

Standard conditions A was followed. White solid. Yield of **11a**: 8.9 mg, 17%. Yield of **11b**: 27.3 mg, 52% (**11a** and **11b** could not be separated, the yields of which were determined by ^{1}H NMR and ^{19}F NMR).

^{1}H NMR (600 MHz, CDCl$_3$) mixture of 11a and 11b: δ 8.34 (s, 0.2H), 8.22 (s, 0.8H), 7.75 (d, *J* = 7.8 Hz, 0.5H), 7.70 (d, *J* = 7.4 Hz, 1.5H), 7.59 – 7.50 (m, 1H), 7.41 (m, 2H), 7.35 – 7.12 (m, 12H), 6.96 – 6.84 (m, 2H). **The accurate ^{13}C NMR data for product 11 (containing 11a, *E*-11b and *Z*-11b) could not provide. Original data can be requested from the corresponding authors. ^{19}F NMR (376 MHz, CDCl$_3$)** δ -110.30, -111.99, -116.97. **HRMS (m/z, ESI$^+$):** Calcd for $C_{27}H_{20}FNO_3SNa^+$ [M+Na$^+$] 480.1040, found 480.1040.

***N*-(4-methoxyphenyl)-3,3-diphenyl-2-(phenylsulfonyl)acrylamide (12a)**

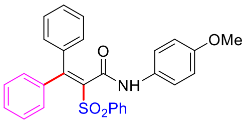

Standard conditions A was followed. White solid. Yield: 14.8 mg, 36%.

^{1}H NMR (600 MHz, CDCl$_3$) δ 7.85 (s, 1H), 7.77 – 7.61 (m, 2H), 7.53 – 7.49 (m, 1H), 7.38 (dd, *J* = 8.4, 7.4 Hz, 2H), 7.33 – 7.30 (m, 2H), 7.28 – 7.19 (m, 6H), 7.14 (m, 4H), 6.76 (d, *J* = 8.9 Hz, 2H), 3.75 (s, 3H). **^{13}C NMR (101**

MHz, $CDCl_3$) δ 161.30, 157.10, 155.85, 141.07, 139.54, 139.31, 136.96, 133.46, 130.11, 129.75, 129.08, 128.79, 128.77, 128.56, 128.41, 128.40, 128.31, 127.83, 122.61, 114.11, 55.53. **HRMS (m/z, ESI$^+$):** Calcd for $C_{28}H_{23}NO_4SNa^+$ [M+Na$^+$] 492.1240, found 492.1240.

3-(4-methoxyphenyl)-*N*,3-diphenyl-2-(phenylsulfonyl)acrylamide (12b)

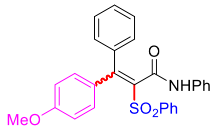

Standard conditions A was followed. White solid. Yield: 26.2 mg, 51%.

^{1}H NMR (600 MHz, $CDCl_3$) mixture of *Z*/*E* isomers: δ 8.05 (s, 0.4H), 7.98 (s, 0.6H), 7.73 (dd, *J* = 8.5, 1.3 Hz, 1.1H), 7.67 (dd, *J* = 8.5, 1.2 Hz, 0.9H), 7.52 (q, *J* = 7.3 Hz, 1H), 7.41 – 7.35 (m, 3H), 7.33 (t, *J* = 7.4 Hz, 0.7H), 7.29 – 7.21 (m, 7.3H), 7.18 – 7.05 (m, 3H), 6.76 (d, *J* = 8.7 Hz, 1.1H), 6.72 (d, *J* = 8.9 Hz, 0.9H), 3.78 (s, 1.6H), 3.70 (s, 1.4H). **^{13}C NMR (101 MHz, $CDCl_3$) mixture of *Z*/*E* isomers:** δ 161.89, 161.55, 161.04, 160.39, 156.63, 156.04, 141.25, 139.84, 138.69, 137.65, 137.38, 137.34, 137.23, 133.42, 133.35, 131.54, 131.21, 130.69, 129.84, 129.43, 129.35, 129.02, 128.94, 128.84, 128.76, 128.74, 128.48, 128.18, 127.74, 125.12, 125.10, 120.60, 113.95, 113.22, 55.36, 55.34. **HRMS (m/z, ESI$^+$):** Calcd for $C_{28}H_{23}NO_4SNa^+$ [M+Na$^+$] 492.1240, found 492.1240.

***N*-methyl-3-phenyl-2-(phenylsulfonyl)-3-(*p*-tolyl)acrylamide (13)**

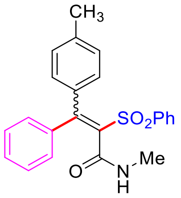

Standard conditions A was followed. White solid. Yield: 75.8 mg, 97%.

^{1}H NMR (400 MHz, $CDCl_3$) mixture of *Z*/*E* isomers: δ 7.67 (d, *J* = 7.8 Hz, 1H), 7.64 (d, *J* = 7.6 Hz, 1H), 7.50 – 7.46 (m, 1H), 7.35 (q, *J* = 7.1 Hz, 2H), 7.30 – 7.16 (m, 5H), 7.06 – 6.96 (m, 4H), 6.53 – 6.38 (m, 1H), 2.70 (d, *J* = 4.0 Hz, 1.6H), 2.65 (d, *J* = 4.0 Hz, 1.4H), 2.35 (s, 1.4H), 2.26 (s, 1.6H). **^{13}C NMR (101 MHz, $CDCl_3$) mixture of *Z*/*E* isomers:** δ 164.20, 164.06, 155.34, 155.06, 141.30, 141.27, 139.79, 139.58, 139.28, 138.86, 138.58, 137.04, 136.42, 134.07, 133.16, 133.13, 129.41, 129.23, 129.19, 129.02, 128.55, 128.50, 128.44, 128.42, 128.30, 128.26, 128.14, 128.10, 127.57, 27.00, 26.92, 21.43, 21.32. **HRMS (m/z, ESI$^+$):** Calcd for $C_{23}H_{21}NO_4SNa^+$ [M+Na$^+$] 430.1083, found 480.1080.

3-([1,1'-biphenyl]-4-yl)-*N*-methyl-3-phenyl-2-(phenylsulfonyl)acrylamide (14)

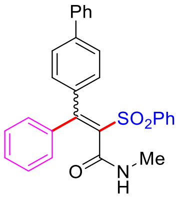

Standard conditions A was followed. White solid. Yield: 69.3 mg, 76%.

^{1}H NMR (400 MHz, $CDCl_3$) mixture of *Z*/*E* isomers: δ 7.84 (d, *J* = 8.2 Hz, 1H), 7.72 – 7.64 (m, 2H), 7.55 (t, *J* = 7.7 Hz, 1H), 7.51 – 7.44 (m, 2H), 7.38 (d, *J* = 7.7 Hz, 3H), 7.27 – 7.10 (m, 7H), 6.99 (q, *J* = 8.3 Hz, 3H), 6.57 (d, *J* = 5.1 Hz, 1H), 2.76 (d, *J* = 4.9 Hz, 3H). **^{13}C NMR (101 MHz, $CDCl_3$) mixture of *Z*/*E* isomers:** δ 164.04, 163.20, 154.62, 153.26, 142.20, 141.79, 141.07, 139.98, 138.20, 136.57, 136.24, 133.55, 133.34, 133.21, 132.89, 132.85, 130.00, 129.96, 129.67, 129.31, 129.24, 128.93, 128.85, 128.67, 128.52, 128.43, 128.34, 128.11, 128.08, 127.57,

126.94, 126.25, 125.92, 125.69, 125.21, 125.12, 125.03, 124.60, 27.07, 26.56. **HRMS (m/z, ESI$^+$):** Calcd for $C_{26}H_{21}NO_3SNa^+$ [M+Na$^+$] 450.1134, found 450.1137.

3-(4-hydroxyphenyl)-*N*-methyl-3-phenyl-2-(phenylsulfonyl)acrylamide (15)

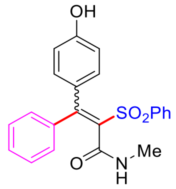

Standard conditions A was followed. White solid. Yield: 29.7 mg, 75%.

^{1}H NMR (400 MHz, DMSO-d_6) mixture of *Z*/*E* isomers: δ 9.88 (s, 0.7H), 9.69 (s, 0.3H), 8.53 – 8.42 (m, 1H), 7.64 – 7.41 (m, 5H), 7.34 – 6.98 (m, 5H), 6.94 – 6.73 (m, 2H), 6.73 – 6.57 (m, 2H), 2.50 (d, *J* = 4.3 Hz, 2H), 2.40 (d, *J* = 4.6 Hz, 1H).**^{13}C NMR (101 MHz, DMSO-d_6) mixture of *Z*/*E* isomers:** δ 164.00, 163.41, 159.12, 158.12, 153.31, 142.34, 142.10, 140.43, 139.49, 137.69, 137.62, 133.57, 133.53, 130.99, 130.64, 130.22, 129.41, 129.38, 129.10, 129.06, 128.59, 128.45, 128.37, 127.95, 127.75, 127.69, 115.34, 114.69, 26.59, 26.47. **HRMS (m/z, ESI$^+$):** Calcd for $C_{22}H_{19}NO_4SNa^+$ [M+Na$^+$] 416.0927, found 416.0928.

3-(4-methoxyphenyl)-*N*-methyl-3-phenyl-2-(phenylsulfonyl)acrylamide (16)

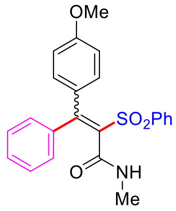

Standard conditions A was followed. White solid. Yield: 79.2 mg, 97%.

^{1}H NMR (400 MHz, CDCl$_3$) mixture of *Z*/*E* isomers: δ 7.68 (d, *J* = 7.7 Hz, 1H), 7.62 (d, *J* = 7.7 Hz, 1H), 7.49 (m, 1H), 7.40 – 7.20 (m, 7H), 7.04 – 7.00 (m, 2H), 6.77 – 6.72 (m, 2H), 6.54 – 6.44 (m, 1H), 3.80 (s, 1.3H), 3.72 (s, 1.7H), 2.73 (d, *J* = 4.2 Hz, 1.7H), 2.64 (d, *J* = 4.2 Hz, 1.3H).**^{13}C NMR (101 MHz, CDCl$_3$) mixture of *Z*/*E* isomers:** δ 164.49, 164.09, 160.67, 160.01, 155.26, 154.70, 141.38, 141.32, 139.79, 139.07, 137.92, 137.12, 133.13, 133.05, 131.53, 131.10, 130.50, 129.48, 129.44, 129.20, 128.62, 128.52, 128.49, 128.22, 128.03, 128.01, 127.53, 113.64, 113.00, 55.34, 55.30, 27.02, 26.92. **HRMS (m/z, ESI$^+$):** Calcd for $C_{23}H_{21}NO_3SNa^+$ [M+Na$^+$] 414.1134, found 414.1138.

***N*-methyl-3-phenyl-2-(phenylsulfonyl)-3-(4-(trifluoromethoxy)phenyl)acrylamide (17)**

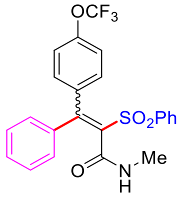

Standard conditions A was followed. Yellowish solid. Yield: 74.1 mg, 80%.

^{1}H NMR (400 MHz, CDCl$_3$) mixture of *Z*/*E* isomers: δ 7.65 (t, *J* = 6.9 Hz, 2H), 7.53 (t, *J* = 7.3 Hz, 1H), 7.40-7.24 (m, 7H), 7.08 (m, 4H), 6.68 (s, 0.4H), 6.57 (s, 0.6H), 2.70 (d, *J* = 8.3 Hz, 3H). **^{13}C NMR (101 MHz, CDCl$_3$) mixture of *Z*/*E* isomer:** δ 163.69, 163.59, 153.33, 149.74, 149.22, 140.96, 140.91, 140.53, 140.34, 138.66, 137.63, 136.40, 135.29, 133.43, 133.39, 130.92, 130.17, 129.70, 129.08, 128.76, 128.69, 128.66, 128.46, 128.30, 128.13, 127.98, 127.81, 120.42 (q, *J* = 259.6 Hz), 120.39, 120.27 (q, *J* = 258.6 Hz), 120.05, 26.93. **^{19}F NMR (376 MHz, CDCl$_3$) mixture of *Z*/*E* isomers:** δ -57.71, -57.75. **HRMS (m/z, ESI$^+$):** Calcd for $C_{23}H_{18}F_3NO_4SNa^+$

[M+Na+] 484.0801, found 484.0804.

N-methyl-3-phenyl-2-(phenylsulfonyl)-3-(4-(trifluoromethyl)phenyl)acrylamide (18)

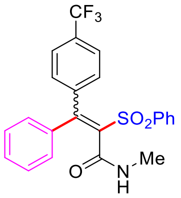

Standard conditions A was followed. White solid. Yield: 63.4 mg, 71%.

^{1}H NMR (400 MHz, CDCl$_3$) mixture of *Z*/*E* isomers: δ 7.67 (m, 2H), 7.58 – 7.35 (m, 6H), 7.34 – 7.05 (m, 6H), 6.69 (q, *J* = 4.9 Hz, 0.4H), 6.54 (q, *J* = 4.9 Hz, 0.6H), 2.69 (m, 3H). **^{13}C NMR (101 MHz, CDCl$_3$) mixture of *Z*/*E* isomers:** δ 163.48, 163.36, 153.24, 153.08, 142.68, 141.00, 140.82, 140.73, 140.47, 140.39, 138.28, 136.15, 133.58, 133.52, 131.01 (q, *J* = 32.5 Hz), 130.46 (q, *J* = 32.6 Hz), 129.80, 129.43, 128.89, 128.84, 128.78, 128.72, 128.65, 128.53, 128.22, 128.17, 128.07, 127.90, 125.26 (q, *J* = 3.5 Hz), 124.61 (q, *J* = 3.9 Hz), 123.80 (q, *J* = 252.5 Hz), 123.59 (q, *J* = 251.5 Hz), 26.91. **^{19}F NMR (376 MHz, CDCl$_3$) mixture of *Z*/*E* isomers:** δ -62.68, -62.86. **HRMS (m/z, ESI+):** Calcd for $C_{23}H_{18}F_3NO_3SNa^+$ [M+Na$^+$] 468.0852, found 468.0852.

N-methyl-3-phenyl-2-(phenylsulfonyl)-3-(*m*-tolyl)acrylamide (19)

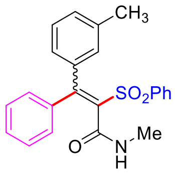

Standard conditions A was followed. White solid. Yield: 73.4 mg, 94%.

^{1}H NMR (400 MHz, CDCl$_3$) mixture of *Z*/*E* isomers: δ 7.65 (d, *J* = 8.0 Hz, 2H), 7.49 (t, *J* = 7.4 Hz, 1H), 7.38 – 7.16 (m, 7H), 7.13 – 7.01 (m, 3.4H), 6.61 (s, 0.6H), 6.40 (m, 1H), 2.67 (m, 3H), 2.21 (s, 1.4H), 2.17 (s, 1.6H). **^{13}C NMR (101 MHz, CDCl$_3$) mixture of *Z*/*E* isomers:** δ 163.97, 163.92, 155.18, 155.16, 141.30, 141.25, 139.68, 139.40, 139.23, 139.20, 138.02, 137.25, 136.89, 136.69, 133.17, 133.03, 130.28, 129.40, 129.23, 129.15, 129.04, 128.76, 128.56, 128.45, 128.29, 128.26, 128.18, 128.15, 128.13, 127.61, 127.53, 126.42, 125.38, 26.93, 21.34. **HRMS (m/z, ESI$^+$):** Calcd for $C_{23}H_{21}NO_3SNa^+$ [M+Na$^+$] 414.1134, found 414.1134.

N-methyl-3-(naphthalen-1-yl)-3-phenyl-2-(phenylsulfonyl)acrylamide (20)

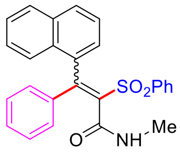

Standard conditions A was followed. White solid. Yield: 29.9 mg, 70%.

^{1}H NMR (400 MHz, CDCl$_3$) mixture of *Z*/*E* isomers: δ 7.67 (t, *J* = 8.7 Hz, 2H), 7.58 (d, *J* = 7.7 Hz, 1H), 7.52 – 7.22 (m, 12H), 7.15 – 7.09 (m, 2H), 6.58 – 6.24 (m, 1H), 2.73 – 2.69 (m, 3H). **^{13}C NMR (101 MHz, CDCl$_3$) mixture of *Z*/*E* isomers:** δ 164.09, 163.85, 154.69, 154.53, 142.20, 141.33, 141.20, 141.16, 140.31, 139.89, 139.84, 139.44, 139.24, 138.09, 136.83, 135.85, 133.23, 133.21, 130.05, 129.79, 129.58, 129.29, 129.09, 128.95, 128.91, 128.61, 128.57, 128.49, 128.45, 128.39, 128.24, 128.16, 127.90, 127.77, 127.71, 127.12, 127.08, 127.03, 126.89, 126.26, 27.07, 26.99. **HRMS (m/z, ESI$^+$):** Calcd for $C_{28}H_{23}NO_3SNa^+$ [M+Na$^+$] 476.1291, found 476.1294.

3-(furan-3-yl)-N-methyl-3-phenyl-2-(phenylsulfonyl)acrylamide (21)

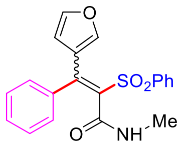

Standard conditions A was followed. Light brown solid. Yield: 30.6 mg, 83%.

^{1}H NMR (400 MHz, CDCl$_3$) mixture of *Z*/*E* isomers: δ 7.90 – 7.84 (m, 0.4H), 7.73 (d, *J* = 1.7 Hz, 0.2H), 7.58 – 7.38 (m, 3H), 7.37 – 7.18 (m, 6H), 7.08 – 7.03 (m, 0.8H), 7.03 – 6.96 (m, 1.6H), 6.67 (q, *J* = 5.0 Hz, 0.8H), 6.45 (d, *J* = 2.0 Hz, 0.8H), 6.11 (q, *J* = 4.6 Hz, 0.2H), 5.94 (d, *J* = 1.9 Hz, 0.2H), 2.98 (d, *J* = 4.9 Hz, 2.4H), 2.60 (d, *J* = 4.9 Hz, 0.6H). **^{13}C NMR (101 MHz, CDCl$_3$) mixture of *Z*/*E* isomers:** δ 164.51, 163.64, 146.75, 146.07, 145.10, 144.96, 143.95, 142.40, 141.26, 139.39, 138.63, 136.20, 135.21, 133.22, 133.11, 129.57, 129.18, 128.60, 128.53, 128.25, 127.55, 125.42, 121.16, 111.84, 109.69, 27.19, 26.83. **HRMS (m/z, ESI$^+$):** Calcd for $C_{20}H_{17}NO_4SNa^+$ [M+Na$^+$] 390.0770, found 390.0773.

N-methyl-3-(1-methyl-1H-indol-5-yl)-3-phenyl-2-(phenylsulfonyl)acrylamide (22)

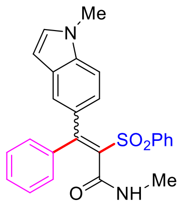

Standard conditions A was followed. Brown solid. Yield: 42.5 mg, 98%.

^{1}H NMR (400 MHz, CDCl$_3$) mixture of *Z*/*E* isomers: δ 7.67 – 7.57 (m, 2H), 7.56 – 7.39 (m, 2H), 7.37 – 7.20 (m, 6H), 7.17 – 7.04 (m, 3H), 7.00 (d, *J* = 3.2 Hz, 0.6H), 6.86 (m, 0.4H), 6.39 (m, 1H), 6.23 (q, *J* = 5.0 Hz, 0.4H), 6.12 (q, *J* = 5.0 Hz, 0.6H), 3.76 (s, 1H), 3.69 (s, 2H), 2.65 (m, 3H). **^{13}C NMR (101 MHz, CDCl$_3$) mixture of *Z*/*E* isomers:** δ 164.83, 164.40, 157.38, 156.86, 141.65, 141.53, 140.46, 138.48, 137.84, 137.38, 137.11, 136.64, 132.92, 132.89, 130.61, 130.06, 129.66, 129.55, 129.36, 128.72, 128.48, 128.39, 128.30, 128.13, 128.08, 128.07, 127.50, 127.42, 123.27, 122.49, 122.25, 109.08, 102.08, 101.76, 32.97, 32.95, 27.01, 26.97. **HRMS (m/z, ESI$^+$):** Calcd for $C_{25}H_{22}N_2O_3S\ Na^+$ [M+Na$^+$] 453.1243, found 453.1245.

3-(1H-indazol-6-yl)-N-methyl-3-phenyl-2-(phenylsulfonyl)acrylamide (23)

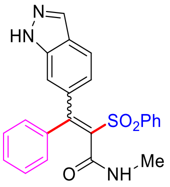

Standard conditions A was followed. White solid. Yield: 21.2 mg, 51%.

^{1}H NMR (400 MHz, CDCl$_3$) mixture of *Z*/*E* isomers: δ 8.53 (s, 0.6H), 8.46 (s, 0.4H), 8.32 (s, 0.4H), 8.08 (d, *J* = 8.2 Hz, 0.6H), 8.03 (d, *J* = 8.9 Hz, 0.4H), 7.91 (s, 0.6H), 7.84 – 7.47 (m, 6H), 7.40 – 7.21 (m, 6H), 7.01 (d, *J* = 7.4 Hz, 0.5H), 6.83 (s, 0.5H), 2.72 (d, *J* = 8.3 Hz, 1.8H), 2.64 (d, *J* = 8.0 Hz, 1.2H). **^{13}C NMR (101 MHz, CDCl$_3$) mixture of *Z*/*E* isomers:** δ 163.63, 163.45, 151.44, 151.15, 149.57, 147.57, 147.32, 141.32, 141.26, 140.84, 140.68, 138.53, 136.46, 135.99, 135.73, 133.67, 133.46, 132.27, 131.04, 130.50, 130.26, 130.03, 129.29, 129.22, 129.09, 129.02, 128.78, 128.71, 128.62, 128.36, 128.23, 128.09, 127.99, 127.56, 127.26, 126.91, 126.71, 26.98, 26.93. **HRMS (m/z, ESI$^+$):** Calcd for $C_{25}H_{20}N_2O_3SNa^+$ [M+Na$^+$] 451.1087, found 451.1090.

3-(dibenzo[b,d]furan-4-yl)-N-methyl-3-phenyl-2-(phenylsulfonyl)acrylamide (24)

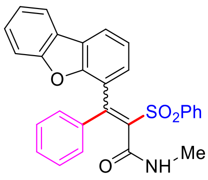

Standard conditions A was followed. White solid. Yield: 40.9 mg, 87%.

^{1}H NMR (400 MHz, CDCl$_3$) mixture of *Z*/*E* isomers: δ 7.96 – 7.67 (m, 2H), 7.55 – 7.51 (m, 2H), 7.49 – 7.17 (m, 11H), 7.12 – 7.02 (m, 2H), 6.30-6.27 (m, 1H), 2.75 (d, *J* = 4.8 Hz, 2.5H), 2.51 (d, *J* = 4.9 Hz, 0.5H). **^{13}C NMR (101 MHz, CDCl$_3$) mixture of *Z*/*E* isomers:** δ 163.68, 156.01, 155.88, 152.19, 150.99, 149.61, 141.96, 141.79, 140.26, 137.93, 136.09, 133.40, 132.92, 129.64, 128.71, 128.69, 128.68, 128.39, 128.24, 128.08, 128.05, 127.95, 127.82, 127.61, 127.30, 126.39, 124.30, 123.89, 123.61, 123.20, 122.89, 122.23, 121.69, 121.22, 121.05, 120.82, 120.58, 111.81, 27.00, 26.72. **HRMS (m/z, ESI$^+$):** Calcd for $C_{28}H_{21}NO_4SNa^+$ [M+Na$^+$] 490.1083, found 490.1082.

3-(dibenzo[b,d]thiophen-4-yl)-*N*-methyl-3-phenyl-2-(phenylsulfonyl)acrylamide (25)

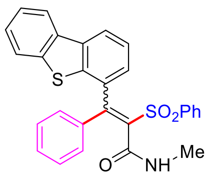

Standard conditions A was followed. Yellowish solid. Yield: 43.3 mg, 89%.

^{1}H NMR (400 MHz, CDCl$_3$) mixture of *Z*/*E* isomers: δ 8.12 (d, *J* = 7.9 Hz, 0.8H), 8.06 (d, *J* = 7.4 Hz, 1.2H), 7.72 (d, *J* = 7.5 Hz, 1.3H), 7.62 – 7.53 (m, 1.8H), 7.49 (t, *J* = 7.5 Hz, 3H), 7.37 (m, 3H), 7.30 – 7.18 (m, 3.5H), 7.12 – 6.95 (m, 1.5H), 6.34 (q, *J* = 4.0 Hz, 0.8H), 6.18 – 6.07 (m, 0.2H), 2.76 (d, *J* = 4.9 Hz, 2.5H), 2.47 (d, *J* = 4.9 Hz, 0.5H). **^{13}C NMR (101 MHz, CDCl$_3$) mixture of *Z*/*E* isomers:** δ 163.68, 163.03, 153.29, 151.79, 141.95, 141.31, 140.81, 139.88, 139.39, 139.20, 138.78, 137.95, 136.50, 136.35, 135.82, 134.97, 134.72, 134.30, 133.37, 132.93, 131.04, 130.06, 129.91, 129.40, 128.84, 128.77, 128.65, 128.33, 128.20, 128.12, 127.54, 127.19, 126.89, 126.49, 124.76, 124.65, 124.43, 123.83, 122.77, 122.48, 122.14, 121.85, 121.79, 121.56, 27.07, 26.79. **HRMS (m/z, ESI$^+$):** Calcd for $C_{28}H_{21}NO_3S_2Na^+$ [M+Na$^+$] 506.0855, found 506.0860.

4-(3-(methylamino)-3-oxo-1-phenyl-2-(phenylsulfonyl)prop-1-en-1-yl)benzyl 2-(3-benzoylphenyl)propanoate (26)

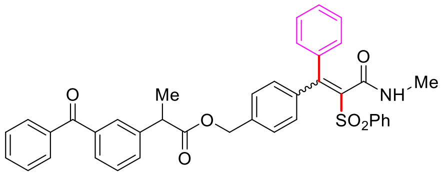

Standard conditions A was followed. Light brown solid. Yield: 59.4 mg, 61%.

^{1}H NMR (400 MHz, CDCl$_3$) mixture of *Z*/*E* isomers: δ 7.82 – 7.66 (m, 3H), 7.66 – 7.51 (m, 5H), 7.51 – 7.24 (m, 9H), 7.21 (m, 2H), 7.10 (dd, *J* = 13.6, 8.0 Hz, 2H), 7.03 – 6.91 (m, 2H), 6.53 (dq, *J* = 9.9, 4.9 Hz, 0.5H), 6.33 (dq, *J* = 10.9, 4.8 Hz, 0.5H), 5.13 (s, 1H), 5.04 (d, *J* = 2.7 Hz, 1H), 3.89 (q, *J* = 7.2 Hz, 0.5H), 3.82 (q, *J* = 7.2 Hz, 0.5H), 2.66 (m, 3H), 1.58 (d, *J* = 7.1 Hz, 1.5H), 1.51 (d, *J* = 7.2 Hz, 1.5H). **^{13}C NMR (101 MHz, CDCl$_3$) mixture of *Z*/*E* isomers:** δ 196.54, 196.49, 173.81, 173.63, 163.83, 163.78, 154.32, 154.17, 141.09, 140.98, 140.66, 139.83, 139.03, 139.01, 137.95, 137.83, 137.39, 137.35, 137.16, 136.68, 136.61, 136.26, 133.26, 133.22, 132.67, 132.60, 131.61, 131.53, 130.08, 130.06, 129.57, 129.31, 129.24, 129.19, 129.15, 129.07, 129.01, 128.68, 128.58, 128.56, 128.41, 128.36, 128.33, 128.13, 128.10, 127.65, 127.56, 126.94, 66.12, 65.85, 45.42, 45.30, 26.93, 26.90, 18.50, 18.41. **HRMS (m/z, ESI$^+$):** Calcd for $C_{39}H_{33}NO_6SNa^+$ [M+Na$^+$] 666.1921, found 666.1922.

N-methyl-3-phenyl-2-(phenylsulfonyl)-3-(p-tolyl)acrylamide (27)

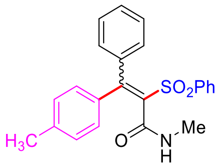

Standard conditions A was followed. White solid. Yield: 76.3 mg, 97%.

^{1}H NMR (400 MHz, CDCl$_3$) mixture of *Z*/*E* isomers: δ 7.68 – 7.63 (m, 2H), 7.51 – 7.46 (m, 1H), 7.35 (q, *J* = 7.1 Hz, 2H), 7.30 – 7.14 (m, 5H), 7.14 – 6.92 (m, 4H), 6.50 – 6.45 (m, 1H), 2.69 (d, *J* = 4.2 Hz, 1.6H), 2.64 (d, *J* = 4.2 Hz, 1.4H), 2.35 (s, 1.4H), 2.26 (s, 1.6H). **^{13}C NMR (101 MHz, CDCl$_3$) mixture of *Z*/*E* isomers:** δ 164.20, 164.06, 155.34, 141.29, 141.26, 139.79, 139.57, 139.27, 138.85, 138.58, 137.04, 136.42, 134.07, 133.16, 133.13, 129.41, 129.22, 129.19, 129.02, 128.54, 128.50, 128.44, 128.42, 128.30, 128.26, 128.14, 128.09, 127.57, 26.99, 26.91, 21.43, 21.32. **HRMS (m/z, ESI$^+$):** Calcd for $C_{23}H_{21}NO_3SNa^+$ [M+Na$^+$] 414.1134, found 414.1139.

3-([1,1'-biphenyl]-4-yl)-N-methyl-3-phenyl-2-(phenylsulfonyl)acrylamide (28)

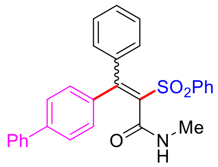

Standard conditions A was followed. Yellowish solid. Yield: 83.8 mg, 92%.

^{1}H NMR (400 MHz, CDCl$_3$) mixture of *Z*/*E* isomers: δ 7.68 (t, *J* = 8.9 Hz, 2H), 7.58 (d, *J* = 7.6 Hz, 1H), 7.50 – 7.42 (m, 5H), 7.40 – 7.22 (m, 9H), 7.15 (d, *J* = 8.0 Hz, 1H), 7.11 (d, *J* = 7.4 Hz, 1H), 6.62 – 6.53 (m, 1H), 2.71 (d, *J* = 4.6 Hz, 1.5H), 2.68 (d, *J* = 4.6 Hz, 1.5H). **^{13}C NMR (101 MHz, CDCl$_3$) mixture of *Z*/*E* isomers:** δ 164.12, 163.89, 154.69, 154.54, 142.16, 141.29, 141.23, 141.20, 140.32, 139.90, 139.84, 139.45, 139.25, 138.12, 136.84, 135.88, 133.25, 133.22, 129.80, 129.56, 129.30, 129.10, 128.97, 128.92, 128.63, 128.59, 128.49, 128.38, 128.15, 127.90, 127.78, 127.71, 127.08, 127.03, 126.88, 126.26, 27.06, 26.99. **HRMS (m/z, ESI$^+$):** Calcd for $C_{28}H_{23}NO_3SNa^+$ [M+Na$^+$] 476.1291, found 476.1292.

3-(4-methoxyphenyl)-N-methyl-3-phenyl-2-(phenylsulfonyl)acrylamide (29)

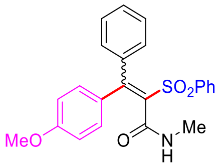

Standard conditions A was followed. White solid. Yield: 36.1 mg, 88%.

^{1}H NMR (400 MHz, CDCl$_3$) mixture of *Z*/*E* isomers: δ 7.69 – 7.61 (m, 2H), 7.58 – 7.44 (m, 1H), 7.39 – 7.19 (m, 7H), 7.07 – 6.97 (m, 2H), 6.79 – 6.70 (m, 2H), 6.51 (q, *J* = 4.9 Hz, 0.6H), 6.42 (q, *J* = 5.0 Hz, 0.4H), 3.80 (s, 1.6H), 3.72 (s, 1.8H), 2.73 (d, *J* = 4.9 Hz, 1.8H), 2.64 (d, *J* = 4.9 Hz, 1.2H). **^{13}C NMR (101 MHz, CDCl$_3$) mixture of *Z*/*E* isomers:** δ 164.48, 164.09, 160.68, 160.01, 155.27, 154.70, 141.39, 141.33, 139.80, 139.07, 137.92, 137.12, 133.12, 133.05, 131.53, 131.10, 130.50, 129.48, 129.43, 129.20, 128.61, 128.51, 128.49, 128.21, 128.03, 128.01, 127.53, 113.64, 113.00, 55.33, 55.28, 27.00, 26.91. **HRMS (m/z, ESI$^+$):** Calcd for $C_{23}H_{21}NO_4SNa^+$ [M+Na$^+$] 430.1083, found 430.1081.

3-(4-fluorophenyl)-N-methyl-3-phenyl-2-(phenylsulfonyl)acrylamide (30)

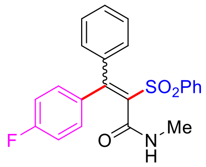

Standard conditions A was followed. Yellowish solid. Yield: 71.9 mg, 91%.

^{1}H NMR (400 MHz, CDCl$_3$) mixture of *Z*/*E* isomers: δ 7.69 (d, *J* = 7.6 Hz, 1H), 7.63 (d, *J* = 7.8 Hz, 1H), 7.52 (q, *J* = 7.7 Hz, 1H), 7.43 – 7.21 (m, 7H), 7.11 – 7.03 (m, 2H), 6.96 – 6.89 (m, 2H), 6.64 (q, *J* = 4.9 Hz, 0.5H), 6.53 (q, *J* = 4.9 Hz, 0.5H), 2.71 (d, *J* = 4.9 Hz, 1.5H), 2.66 (d, *J* = 4.8 Hz, 1.5H). **^{13}C NMR (101 MHz, CDCl$_3$) mixture of *Z*/*E* isomers:** δ 163.91, 163.80, 163.19 (d, *J* = 251.5 Hz), 162.80 (d, *J* = 250.5 Hz), 154.01, 153.82, 141.07 (d, *J* = 9.0 Hz), 139.96, 139.66, 139.12, 136.70, 135.28 (d, *J* = 3.3 Hz), 133.34 (d, *J* = 8.7 Hz), 132.75 (d, *J* = 3.3 Hz), 131.28 (d, *J* = 8.4 Hz), 130.64 (d, *J* = 8.5 Hz), 129.63, 129.15, 128.67, 128.61, 128.36, 128.08, 128.02, 127.71, 115.40 (d, *J* = 21.7 Hz), 114.73 (d, *J* = 21.7 Hz), 26.94, 26.90. **^{19}F NMR (376 MHz, CDCl$_3$) mixture of *Z*/*E* isomers:** δ -110.71, -112.26. **HRMS (m/z, ESI$^+$):** Calcd for $C_{22}H_{18}FNO_3SNa^+$ [M+Na$^+$] 418.0884, found 418.0880.

3-(4-chlorophenyl)-*N*-methyl-3-phenyl-2-(phenylsulfonyl)acrylamide (31)

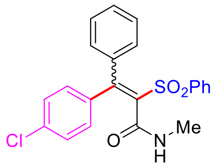

Standard conditions A was followed. Yellowish solid. Yield: 77.6 mg, 94%.

^{1}H NMR (400 MHz, CDCl$_3$) mixture of *Z*/*E* isomers: δ 7.69 (d, *J* = 7.8 Hz, 0.9H), 7.63 (d, *J* = 7.7 Hz, 1.1H), 7.56 – 7.49 (m, 1H), 7.43 – 7.29 (m, 3H), 7.29 – 7.18 (m, 6H), 7.04 (t, *J* = 8.0 Hz, 2H), 6.61 (q, *J* = 5.9 Hz, 0.6H), 6.47 (q, *J* = 5.6 Hz, 0.4H), 2.72 (d, *J* = 4.2 Hz, 1.6H), 2.67 (d, *J* = 4.1 Hz, 1.4H). **^{13}C NMR (101 MHz, CDCl$_3$) mixture of *Z*/*E* isomers:** δ 163.73, 163.71, 153.66, 153.57, 140.99, 140.89, 139.97, 138.81, 137.63, 136.46, 135.65, 135.27, 134.77, 133.47, 133.36, 130.61, 129.87, 129.71, 129.12, 128.74, 128.71, 128.64, 128.56, 128.42, 128.35, 128.12, 128.10, 127.91, 127.77, 27.01, 26.94. **HRMS (m/z, ESI$^+$):** Calcd for $C_{22}H_{18}ClNO_3SNa^+$ [M+Na$^+$] 434.0588, found 434.0591.

(*Z*)-3-(4-bromophenyl)-*N*-methyl-3-phenyl-2-(phenylsulfonyl)acrylamide (*Z*-32)

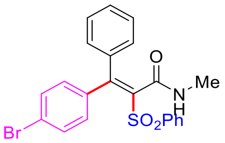

Standard conditions A was followed. White solid. Yield: 44.0 mg, 48%.

^{1}H NMR (400 MHz, CDCl$_3$): δ 7.69 (d, *J* = 7.1 Hz, 2H), 7.59 – 7.48 (m, 1H), 7.45 – 7.34 (m, 4H), 7.32 – 7.13 (m, 5H), 6.97 (d, *J* = 8.4 Hz, 2H), 6.58 (q, *J* = 4.9 Hz, 1H), 2.65 (d, *J* = 4.9 Hz, 3H).**^{13}C NMR (101 MHz, CDCl$_3$):** δ 163.71, 153.60, 140.99, 139.94, 138.70, 135.76, 133.47, 130.84, 129.71, 128.73, 128.42, 128.35, 128.09, 123.00, 26.92. **HRMS (m/z, ESI$^+$):** Calcd for $C_{22}H_{18}BrNO_3SNa^+$ [M+Na$^+$] 478.0083, found 478.0084.

(*E*)-3-(4-bromophenyl)-*N*-methyl-3-phenyl-2-(phenylsulfonyl)acrylamide (*E*-32)

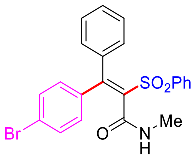

Standard conditions A was followed. White powder. Yield: 44.0 mg, 48%.

^{1}H NMR (400 MHz, CDCl$_3$): δ 7.62 (d, *J* = 7.1 Hz, 2H), 7.56 – 7.48 (m, 1H), 7.40 – 7.29 (m, 5H), 7.26 (d, *J* = 8.0 Hz, 2H), 7.16 (d, *J* = 8.6 Hz, 2H), 7.04 (d, *J* = 6.9 Hz, 2H), 6.49 (q, *J* = 4.9 Hz, 1H), 2.73 (d, *J* = 4.9 Hz, 3H). **^{13}C NMR (101 MHz, CDCl$_3$):** δ 163.68, 153.59, 140.80, 139.92, 138.08, 136.38, 133.38, 131.54, 130.07, 129.09, 128.77, 128.65, 128.12, 127.79, 124.10, 27.03. **HRMS (m/z, ESI$^+$):** Calcd for $C_{22}H_{18}BrNO_3SNa^+$ [M+Na$^+$]

478.0083, found 478.0087.

***N*-methyl-3-phenyl-2-(phenylsulfonyl)-3-(4-(trifluoromethyl)phenyl)acrylamide (33)**

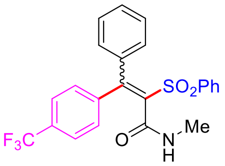

Standard conditions A was followed. White solid. Yield: 75.7 mg, 85%.

^{1}H NMR (400 MHz, CDCl$_3$) mixture of *Z*/*E* isomers: δ 7.68 – 7.65 (m, 2H), 7.58 – 7.36 (m, 6H), 7.33 (d, *J* = 7.5 Hz, 0.4H), 7.30 – 7.20 (m, 5H), 7.09 (d, *J* = 7.0 Hz, 0.6H), 6.70 (q, *J* = 4.9 Hz, 0.4H), 6.55 (q, *J* = 4.9 Hz, 0.6H), 2.69 (d, *J* = 4.8 Hz, 3H). **^{13}C NMR (101 MHz, CDCl$_3$) mixture of *Z*/*E* isomers:** δ 163.48, 163.36, 153.24, 153.08, 142.68, 141.00, 140.83, 140.74, 140.48, 140.40, 138.28, 136.15, 133.58, 133.52, 131.00 (q, *J* = 32.6 Hz), 130.45 (q, *J* = 32.7 Hz), 129.79, 129.43, 128.89, 128.83, 128.78, 128.72, 128.64, 128.53, 128.22, 128.17, 128.07, 127.90, 125.26 (q, *J* = 3.1 Hz), 124.61 (q, *J* = 4.0 Hz), 123.80 (q, *J* = 252.5 Hz), 123.60 (q, *J* = 253.5 Hz), 26.91. **^{19}F NMR (376 MHz, CDCl$_3$) mixture of *Z*/*E* isomers:** δ -62.68, -62.86. **HRMS (m/z, ESI$^+$):** Calcd for $C_{23}H_{18}F_3NO_3S\ Na^+$ [M+Na$^+$] 468.0852, found 468.0856.

methyl 4-(3-(methylamino)-3-oxo-1-phenyl-2-(phenylsulfonyl)prop-1-en-1-yl)benzoate (34)

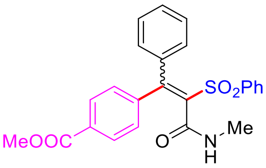

Standard conditions A was followed. White solid. Yield: 40.6 mg, 93%.

^{1}H NMR (400 MHz, CDCl$_3$) mixture of *Z*/*E* isomers: δ 7.95 (d, *J* = 8.0 Hz, 1.2H), 7.89 (d, *J* = 8.1 Hz, 0.8H), 7.70 (d, *J* = 7.5 Hz, 1.2H), 7.66 (d, *J* = 7.5 Hz, 0.8H), 7.63 – 7.45 (m, 1H), 7.44 – 7.22 (m, 7H), 7.19 (d, *J* = 8.0 Hz, 1.2H), 7.08 (d, *J* = 7.2 Hz, 0.8H), 6.67 (q, *J* = 4.8 Hz, 0.4H), 6.59 (q, *J* = 4.8 Hz, 0.6H), 3.94 (s, 1.8H), 3.87 (s, 1.2H), 2.69 – 2.66 (s, 3H). **^{13}C NMR (101 MHz, CDCl$_3$) mixture of *Z*/*E* isomers:** δ 166.63, 166.38, 163.66, 163.45, 153.67, 143.60, 141.56, 140.95, 140.82, 140.72, 139.92, 138.45, 136.17, 133.56, 133.43, 130.61, 129.96, 129.70, 129.48, 129.04, 128.92, 128.88, 128.75, 128.67, 128.46, 128.27, 128.18, 128.16, 127.83, 52.34, 52.32, 26.92, 26.91. **HRMS (m/z, ESI$^+$):** Calcd for $C_{24}H_{21}NO_5SNa^+$ [M+Na$^+$] 458.1033, found 458.1033.

3-([1,1'-biphenyl]-2-yl)-*N*-methyl-3-phenyl-2-(phenylsulfonyl)acrylamide (35)

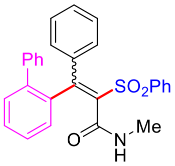

Standard conditions A was followed. White solid. Yield: 42.4 mg, 93%.

^{1}H NMR (400 MHz, CDCl$_3$) mixture of *Z*/*E* isomers: δ 7.83 (d, *J* = 7.6 Hz, 1H), 7.67 (m, 1H), 7.60 – 6.96 (m, 12H), 6.88 (t, *J* = 7.6 Hz, 2H), 6.82 (t, *J* = 7.6 Hz, 1H), 6.68 (d, *J* = 7.6 Hz, 1H), 6.60 (d, *J* = 7.4 Hz, 1H), 6.33 (d, *J* = 7.6 Hz, 0.6H), 6.24 (s, 0.4H), 2.76 (d, *J* = 4.3 Hz, 1.2H), 2.54 (d, *J* = 4.2 Hz, 1.8H). **^{13}C NMR (101 MHz, CDCl$_3$) mixture of *Z*/*E* isomers:** δ 164.01, 163.71, 157.65, 155.87, 141.49, 141.04, 140.80, 140.75, 140.53, 139.44, 138.93, 138.88, 136.34, 136.10, 133.86, 133.30, 130.98, 130.48, 130.02, 129.44, 129.26, 129.22, 128.98, 128.80, 128.78, 128.76, 128.66, 128.61, 128.57, 128.37, 128.10, 127.98, 127.78, 127.75, 127.38, 127.32, 127.06, 126.93, 126.70, 126.17, 26.97, 26.87. **HRMS (m/z, ESI$^+$):** Calcd for $C_{28}H_{23}NO_3SNa^+$ [M+Na$^+$] 476.1291, found 476.1287.

3-(2-chlorophenyl)-*N*-methyl-3-phenyl-2-(phenylsulfonyl)acrylamide (36)

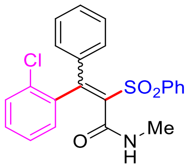

Standard conditions A was followed. White solid. Yield: 78.5 mg, 95%.

^{1}H NMR (400 MHz, $CDCl_3$) mixture of *Z*/*E* isomers: δ 7.73 (d, *J* = 7.7 Hz, 2H), 7.55 (t, *J* = 7.4 Hz, 1H), 7.47 (d, *J* = 7.3 Hz, 1H), 7.41 (t, *J* = 7.6 Hz, 2H), 7.37 – 7.17 (m, 8H), 6.45 – 6.26 (m, 1H), 2.66 (d, *J* = 4.3 Hz, 3H). **^{13}C NMR (101 MHz, $CDCl_3$) mixture of *Z*/*E* isomers:** δ 163.78, 162.71, 152.32, 151.35, 142.22, 140.65, 140.35, 140.29, 137.94, 137.14, 135.27, 134.83, 133.64, 133.42, 132.81, 131.40, 130.05, 129.78, 129.70, 129.48, 129.43, 129.09, 128.93, 128.70, 128.66, 128.60, 128.53, 128.23, 128.12, 127.57, 127.10, 127.02, 126.05, 26.95, 26.69. **HRMS (m/z, ESI^{+}):** Calcd for $C_{22}H_{18}ClNO_3SNa^+$ [M+Na$^+$] 434.0588, found 434.0590.

3-(3-bromophenyl)-*N*-methyl-3-phenyl-2-(phenylsulfonyl)acrylamide (37)

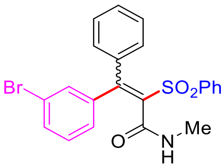

Standard conditions A was followed. Yellowish solid. Yield: 43.5 mg, 95%.

^{1}H NMR (400 MHz, $CDCl_3$) mixture of *Z*/*E* isomers: δ 7.69 (d, *J* = 7.7 Hz, 1.2H), 7.64 (d, *J* = 7.8 Hz, 0.8H), 7.58 – 7.50 (m, 1H), 7.46 – 7.09 (m, 10H), 7.06 (d, *J* = 7.2 Hz, 0.6H), 6.97 (s, 0.4H), 6.60 (q, *J* = 4.9 Hz, 0.4H), 6.53 (q, *J* = 4.9 Hz, 0.6H), 2.71 – 2.86 (m, 3H). **^{13}C NMR (101 MHz, $CDCl_3$) mixture of *Z*/*E* isomers:** δ 163.55, 163.44, 153.04, 152.95, 141.12, 140.91, 140.82, 140.59, 140.40, 138.67, 138.39, 136.18, 133.53, 133.40, 132.41, 131.49, 131.24, 131.10, 129.87, 129.71, 129.28, 128.99, 128.76, 128.67, 128.48, 128.24, 128.14, 128.11, 128.02, 127.85, 126.92, 122.33, 121.74, 26.96, 26.94. **HRMS (m/z, ESI^{+}):** Calcd for $C_{22}H_{18}BrNO_3SNa^+$ [M+Na$^+$] 478.0083, found 478.0083.

3-(3-iodophenyl)-*N*-methyl-3-phenyl-2-(phenylsulfonyl)acrylamide (38)

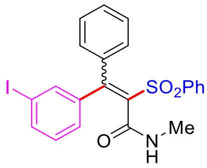

Standard conditions A was followed. Light brown solid. Yield: 44.6 mg, 89%.

^{1}H NMR (400 MHz, $CDCl_3$) mixture of *Z*/*E* isomers: δ 7.68 (d, *J* = 7.8 Hz, 0.8H), 7.64 (d, *J* = 7.7 Hz, 1.2H), 7.61 – 7.17 (m, 10H), 7.12 – 6.94 (m, 2H), 6.52 (d, *J* = 6.0 Hz, 0.4H), 6.45 – 6.42 (m, 0.6H), 2.72 – 2.69 (m, 3H).**^{13}C NMR (101 MHz, $CDCl_3$) mixture of *Z*/*E* isomers:** δ 163.54, 163.44, 152.97, 152.89, 141.15, 140.89, 140.80, 140.48, 140.34, 138.75, 138.37, 138.34, 137.39, 136.79, 136.72, 136.18, 133.58, 133.40, 129.96, 129.73, 129.39, 128.99, 128.81, 128.78, 128.68, 128.48, 128.25, 128.15, 128.12, 127.86, 127.54, 93.98, 93.41, 26.98. **HRMS (m/z, ESI^{+}):** Calcd for $C_{22}H_{18}INO_3SNa^+$ [M+Na$^+$] 525.9944, found 525.9947.

***N*-methyl-3-(naphthalen-1-yl)-3-phenyl-2-(phenylsulfonyl)acrylamide (39)**

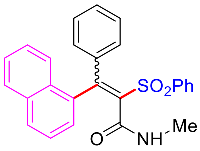

Standard conditions A was followed. White solid. Yield: 79.3 mg, 92%.

^{1}H NMR (400 MHz, $CDCl_3$) mixture of *Z*/*E* isomers: δ 7.96 (d, *J* = 9.0 Hz, 0.3H), 7.83 (d, *J* = 8.2 Hz, 0.7H),

7.76 – 7.68 (m, 2H), 7.59 – 7.10 (m, 12H), 6.99 (q, *J* = 8.0 Hz, 2H), 6.60 (q, *J* = 4.8 Hz, 0.7H), 6.26 (q, *J* = 4.9 Hz, 0.3H), 2.75 (d, *J* = 4.8 Hz, 2.1H), 2.35 (d, *J* = 4.8 Hz, 0.9H). **^{13}C NMR (101 MHz, $CDCl_3$) mixture of *Z*/*E* isomers:** δ 164.05, 163.21, 154.62, 153.26, 142.22, 141.81, 141.11, 140.03, 138.22, 136.58, 136.25, 133.55, 133.34, 133.21, 132.90, 132.85, 130.00, 129.97, 129.65, 129.30, 129.24, 128.94, 128.85, 128.51, 128.45, 128.33, 128.12, 128.08, 127.57, 126.94, 126.25, 126.21, 125.92, 125.68, 125.25, 125.12, 125.04, 124.60, 27.06, 26.55. **HRMS (m/z, ESI$^+$):** Calcd for $C_{26}H_{21}NO_3SNa^+$ [M+Na$^+$] 450.1134, found 450.1136.

3-(benzo[d][1,3]dioxol-5-yl)-*N*-methyl-3-phenyl-2-(phenylsulfonyl)acrylamide (40)

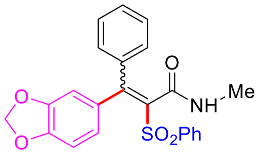

Standard conditions A was followed. Yellow solid. Yield: 30.8 mg, 70%.

^{1}H NMR (400 MHz, $CDCl_3$) mixture of *Z*/*E* isomers: δ 7.71 (dd, *J* = 8.4, 1.3 Hz, 1H), 7.59 (dd, *J* = 8.4, 1.3 Hz, 1H), 7.55 – 7.44 (m, 1H), 7.43 – 7.36 (m, 1H), 7.36 – 7.19 (m, 5H), 7.03 – 6.97 (m, 1H), 6.84 (dd, *J* = 8.2, 1.9 Hz, 0.5H), 6.73 – 6.58 (m, 2H), 6.40 (d, *J* = 1.7 Hz, 0.5H), 6.32 (q, *J* = 5.0 Hz, 0.5H), 6.27 – 6.21 (m, 0.5H), 5.94 (s, 1H), 5.89 (s, 1H), 2.75 (d, *J* = 4.9 Hz, 1.5H), 2.65 (d, *J* = 4.9 Hz, 1.5H). **^{13}C NMR (101 MHz, $CDCl_3$) mixture of *Z*/*E* isomers:** δ 164.26, 163.87, 154.77, 154.71, 148.98, 148.22, 147.74, 147.17, 141.20, 141.17, 139.45, 139.42, 138.50, 137.03, 133.33, 133.22, 133.16, 130.55, 129.70, 129.36, 128.73, 128.63, 128.59, 128.39, 128.18, 128.12, 127.72, 123.91, 123.46, 109.87, 109.18, 108.13, 107.63, 101.62, 101.39, 27.15, 27.00. **HRMS (m/z, ESI$^+$):** Calcd for $C_{23}H_{19}NO_5SNa^+$ [M+Na$^+$] 444.0876, found 444.0877.

3-(dibenzo[b,d]furan-3-yl)-*N*-methyl-3-phenyl-2-(phenylsulfonyl)acrylamide (41)

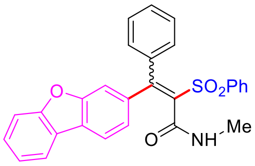

Standard conditions A was followed. Yellow solid. Yield: 27.9 mg, 50%.

^{1}H NMR (400 MHz, $CDCl_3$) mixture of *Z*/*E* isomers: δ 7.87 (d, *J* = 8.1 Hz, 0.5H), 7.80 (d, *J* = 8.3 Hz, 0.5H), 7.75 – 7.51 (m, 1H), 7.61 – 7.56 (m, 2H), 7.51 – 7.33 (m, 4H), 7.33 – 7.12 (m, 8H), 7.04 – 6.96 (m, 1H), 6.35-6.29 (m, 1H), 2.64 – 2.60 (m, 3H). **^{13}C NMR (101 MHz, $CDCl_3$) mixture of *Z*/*E* isomers:** δ 163.97, 163.83, 156.87, 156.73, 155.63, 155.28, 154.72, 154.59, 141.04, 140.98, 139.90, 139.74, 139.22, 138.20, 136.92, 135.77, 133.31, 133.28, 129.67, 129.26, 128.73, 128.63, 128.55, 128.47, 128.40, 128.16, 128.03, 127.77, 125.46, 124.51, 124.10, 123.67, 123.40, 123.30, 123.08, 123.03, 121.02, 120.93, 120.41, 119.82, 112.83, 112.13, 111.84, 111.83, 27.04, 26.99. **HRMS (m/z, ESI$^+$):** Calcd for $C_{28}H_{21}NO_4SNa^+$ [M+Na$^+$] 490.1083, found 490.1086.

methyl 3-(3-(methylamino)-3-oxo-1-phenyl-2-(phenylsulfonyl)prop-1-en-1-yl)thiophene-2-carboxylate (42)

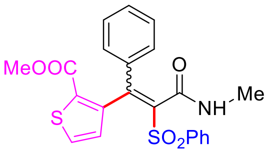

Standard conditions A was followed. White solid. Yield: 21.2 mg, 48%.

^{1}H NMR (400 MHz, $CDCl_3$) mixture of *Z*/*E* isomers: δ 7.73 (d, *J* = 8.5 Hz, 1.7H), 7.65 (d, *J* = 8.5 Hz, 0.3H), 7.54 (t, *J* = 7.4 Hz, 1H), 7.46 – 7.38 (m, 3H), 7.38 – 7.13 (m, 6H), 6.96 (d, *J* = 5.1 Hz, 0.1H), 6.71 (d, *J* = 5.5 Hz, 0.9H), 6.35 (s, 0.1H), 6.10 (d, *J* = 5.2 Hz, 0.9H), 3.84 (s, 0.4H), 3.69 (s, 2.6H), 2.67 (d, *J* = 5.0 Hz, 2.6H), 2.63 (d, *J* = 5.0 Hz, 0.4H). **^{13}C NMR (101 MHz, $CDCl_3$) mixture of *Z*/*E* isomers:** δ 163.94, 161.81, 149.33, 141.70,

139.61, 137.04, 133.42, 130.77, 130.00, 129.64, 128.72, 128.63, 128.47, 128.44, 128.35, 52.21, 27.02. **HRMS (m/z, ESI$^+$):** Calcd for $C_{22}H_{20}NO_5S_2^+$ [M+H$^+$] 442.0777, found 442.0775.

(*E*)-N-methyl-N-phenyl-3-(phenylsulfonyl)acrylamide (43)

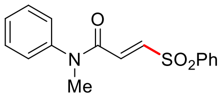

Standard conditions A was followed. White solid. Yield: 19.2 mg, 30%.

^{1}H NMR (400 MHz, CDCl$_3$)) δ 7.84 – 7.73 (m, 2H), 7.66 – 7.58 (m, 1H), 7.51 (dd, *J* = 8.5, 7.1 Hz, 2H), 7.47 – 7.34 (m, 3H), 7.28 (d, *J* = 14.7 Hz, 1H), 7.13 (dd, *J* = 8.2, 1.4 Hz, 2H), 6.81 (d, *J* = 14.7 Hz, 1H), 3.35 (s, 3H). **^{13}C NMR (101 MHz, CDCl$_3$)** δ 162.44, 142.10, 140.57, 139.00, 134.16, 131.81, 130.21, 129.56, 128.72, 128.18, 126.98, 37.94.

***N*-methyl-3,3-diphenyl-2-tosylacrylamide (44)**

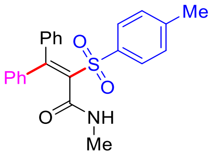

Standard conditions A was followed. White solid. Yield: 37.5 mg, 96%.

^{1}H NMR (400 MHz, CDCl$_3$) δ 7.53 (d, *J* = 8.0 Hz, 2H), 7.34 – 7.22 (m, 8H), 7.16 (d, *J* = 8.1 Hz, 2H), 7.09 (d, *J* = 7.2 Hz, 2H), 6.28 (q, *J* = 4.9 Hz, 1H), 2.66 (d, *J* = 4.9 Hz, 3H), 2.38 (s, 3H).**^{13}C NMR (101 MHz, CDCl$_3$)** δ 164.05, 154.53, 144.20, 139.71, 139.37, 138.18, 136.98, 129.39, 129.22, 129.07, 128.49, 128.32, 128.29, 128.26, 127.61, 26.90, 21.68. **HRMS (m/z, ESI$^+$):** Calcd for $C_{23}H_{21}NO_3SNa^+$ [M+Na$^+$] 414.1134, found 414.1135.

2-((4-isopropylphenyl)sulfonyl)-*N*-methyl-3,3-diphenylacrylamide (45)

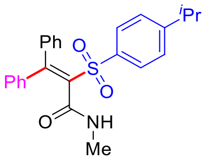

Standard conditions A was followed. White solid. Yield: 17.2 mg, 41%.

^{1}H NMR (400 MHz, CDCl$_3$) δ 7.54 (d, *J* = 8.5 Hz, 2H), 7.33 – 7.16 (m, 10H), 7.08 – 7.01 (m, 2H), 6.26 (q, *J* = 5.0 Hz, 1H), 2.92 (dq, *J* = 11.2, 5.8, 4.7 Hz, 1H), 2.70 (d, *J* = 4.9 Hz, 3H), 1.23 (d, *J* = 6.9 Hz, 6H). **^{13}C NMR (101 MHz, CDCl$_3$)** δ 163.96, 154.79, 154.46, 139.86, 139.32, 138.29, 136.97, 129.41, 129.06, 128.43, 128.34, 128.30, 127.61, 126.68, 34.24, 26.93, 23.66. **HRMS (m/z, ESI$^+$):** Calcd for $C_{25}H_{25}NO_3SNa^+$ [M+Na$^+$] 442.1447, found 442.1448.

2-((4-chlorophenyl)sulfonyl)-*N*-methyl-3,3-diphenylacrylamide (46)

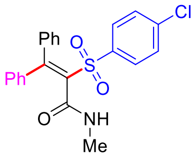

Standard conditions A was followed. White solid. Yield: 36.0 mg, 87%.

^{1}H NMR (400 MHz, CDCl$_3$) δ 7.57 (d, *J* = 8.6 Hz, 2H), 7.37 – 7.31 (m, 3H), 7.31 – 7.23 (m, *J* = 2.2, 1.8 Hz, 7H), 7.07 (d, *J* = 7.2 Hz, 2H), 6.33 – 6.28 (m, 1H), 2.67 (d, *J* = 4.9 Hz, 3H). **^{13}C NMR (101 MHz, CDCl$_3$)** δ 163.89, 155.29, 139.89, 139.64, 139.22, 139.04, 136.60, 129.67, 129.12, 128.85, 128.74, 128.39, 128.31, 127.70, 26.95. **HRMS (m/z, ESI$^+$):** Calcd for $C_{22}H_{18}ClNO_3SNa^+$ [M+Na$^+$] 434.0588, found 434.0588.

***N*-methyl-3,3-diphenyl-2-((4-(trifluoromethyl)phenyl)sulfonyl)acrylamide (47)**

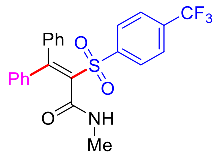

Standard conditions A was followed. White solid. Yield: 39.1 mg, 88%.

^{1}H NMR (400 MHz, CDCl$_3$) δ 7.77 (d, *J* = 8.1 Hz, 2H), 7.62 (d, *J* = 8.2 Hz, 2H), 7.36 – 7.24 (m, 8H), 7.04 (d, *J* = 7.6 Hz, 2H), 6.22 (q, *J* = 5.0 Hz, 1H), 2.70 (d, *J* = 4.9 Hz, 3H). **^{13}C NMR (101 MHz, CDCl$_3$)** δ 163.69, 155.94, 144.68, 138.90, 138.82, 136.44, 134.63 (q, *J* = 33.0 Hz), 129.86, 129.17, 128.86, 128.69, 128.43, 128.36, 127.71, 125.62 (q, *J* = 3.7 Hz), 123.22 (q, *J* = 273.2 Hz), 26.99. **^{19}F NMR (376 MHz, CDCl$_3$)** δ -63.15. **HRMS (m/z, ESI$^+$):** Calcd for $C_{23}H_{18}F_3NO_3SNa^+$ [M+Na$^+$] 468.0852, found 468.0852.

2-((4-cyanophenyl)sulfonyl)-*N*-methyl-3,3-diphenylacrylamide (48)

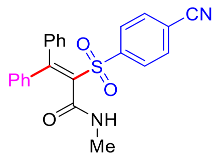

Standard conditions A was followed. Yellowish solid. Yield: 20.6 mg, 51%.

^{1}H NMR (400 MHz, CDCl$_3$) δ 7.76 (d, *J* = 8.0 Hz, 2H), 7.66 (d, *J* = 8.0 Hz, 2H), 7.40 – 7.23 (m, 8H), 7.03 (d, *J* = 7.5 Hz, 2H), 6.13 (q, *J* = 5.0 Hz, 1H), 2.69 (d, *J* = 4.9 Hz, 3H). **^{13}C NMR (101 MHz, CDCl$_3$)** δ 163.63, 156.35, 145.35, 138.78, 138.38, 136.27, 132.29, 130.05, 129.24, 129.06, 128.77, 128.50, 128.38, 127.77, 117.42, 116.66, 27.01. **HRMS (m/z, ESI$^+$):** Calcd for $C_{23}H_{18}N_2O_3SNa^+$ [M+Na$^+$] 425.0930, found 425.0931.

2-((4-acetylphenyl)sulfonyl)-*N*-methyl-3,3-diphenylacrylamide (49)

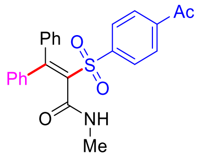

Standard conditions A was followed. White powder. Yield: 26.5 mg, 63%.

^{1}H NMR (400 MHz, CDCl$_3$) δ 7.93 (d, *J* = 8.5 Hz, 2H), 7.75 (d, *J* = 8.7 Hz, 2H), 7.36 – 7.24 (m, 8H), 7.06 (d, *J* = 7.4 Hz, 2H), 6.27 (q, *J* = 5.3 Hz, 1H), 2.68 (d, *J* = 4.8 Hz, 3H), 2.63 (s, 3H). **^{13}C NMR (101 MHz, CDCl$_3$)** δ 197.09, 163.81, 155.85, 145.02, 140.16, 139.04, 138.81, 136.52, 129.79, 129.19, 128.86, 128.51, 128.40, 128.36, 128.32, 127.69, 27.00, 26.97. **HRMS (m/z, ESI$^+$):** Calcd for $C_{24}H_{21}NO_4SNa^+$ [M+Na$^+$] 442.1083, found 442.1087.

2-((2-methoxyphenyl)sulfonyl)-*N*-methyl-3,3-diphenylacrylamide (50)

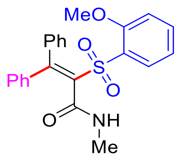

Standard conditions A was followed. White solid. Yield: 30.9 mg, 76%.

^{1}H NMR (400 MHz, CDCl$_3$) δ 7.43 – 7.36 (m, 1H), 7.31 – 7.21 (m, 6H), 7.16 (t, *J* = 7.4 Hz, 1H), 7.08 (t, *J* = 7.6 Hz, 2H), 6.97 (d, *J* = 7.1 Hz, 2H), 6.92 (d, *J* = 8.3 Hz, 1H), 6.70 (t, *J* = 7.6 Hz, 1H), 6.57 (q, *J* = 5.0 Hz, 1H), 4.05 (s, 3H), 2.77 (d, *J* = 4.9 Hz, 3H). **^{13}C NMR (101 MHz, CDCl$_3$)** δ 163.47, 156.85, 154.77, 140.04, 139.06, 137.28, 134.96, 129.90, 129.39, 129.27, 128.70, 128.55, 128.50, 128.27, 127.48, 120.41, 111.84, 56.43, 27.01. **HRMS (m/z, ESI$^+$):** Calcd for $C_{23}H_{21}NO_4SNa^+$ [M+Na$^+$] 430.1083, found 430.1085.

2-((2-fluorophenyl)sulfonyl)-*N*-methyl-3,3-diphenylacrylamide (51)

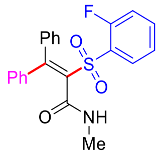

Standard conditions A was followed. White solid. Yield: 36.0 mg, 91%.

^{1}H NMR (400 MHz, CDCl$_3$) δ 7.48 (q, *J* = 7.3 Hz, 1H), 7.41 (t, *J* = 7.5 Hz, 1H), 7.27 (d, *J* = 5.6 Hz, 6H), 7.18 (t, *J* = 7.5 Hz, 2H), 7.11 (d, *J* = 7.3 Hz, 3H), 7.01 (t, *J* = 7.7 Hz, 1H), 6.26 (q, *J* = 5.2 Hz, 1H), 2.67 (d, *J* = 4.9 Hz, 3H). **^{13}C NMR (101 MHz, CDCl$_3$)** δ 163.14, 159.32 (d, *J* = 255.8 Hz), 156.09, 139.52, 138.12, 136.96, 135.52 (d, *J* = 8.7 Hz), 130.07, 129.77, 129.37, 128.95 (d, *J* = 13.1 Hz), 128.93, 128.64, 128.37, 127.66, 124.18 (d, *J* = 3.7 Hz), 116.64 (d, *J* = 20.9 Hz), 26.99. **^{19}F NMR (376 MHz, CDCl$_3$)** δ -108.91. **HRMS (m/z, ESI$^+$):** Calcd for $C_{22}H_{18}NO_3SNa^+$ [M+Na$^+$] 418.0884, found 418.0887.

methyl 2-((3-(methylamino)-3-oxo-1,1-diphenylprop-1-en-2-yl)sulfonyl)benzoate (52)

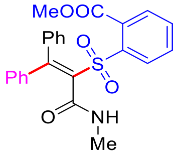

Standard conditions A was followed. White solid. Yield: 27.0 mg, 62%.

^{1}H NMR (400 MHz, CDCl$_3$) δ 7.69 (d, *J* = 7.6 Hz, 1H), 7.46 – 7.41 (m, 2H), 7.36 – 7.33 (m, 2H), 7.27 – 7.25 (m, 3H), 7.20 (t, *J* = 7.8 Hz, 1H), 7.16 – 7.12 (m, 1H), 7.10 – 7.04 (m, 4H), 4.00 (s, 3H), 2.72 (d, *J* = 4.5 Hz, 3H). **^{13}C NMR (101 MHz, CDCl$_3$)** δ 167.52, 162.97, 150.30, 141.18, 139.88, 139.32, 137.20, 132.39, 131.16, 130.94, 130.69, 129.85, 129.09, 128.96, 128.51, 128.30, 128.16, 127.88, 53.46, 26.80. **HRMS (m/z, ESI$^+$):** Calcd for $C_{24}H_{21}NO_5SNa^+$ [M+Na$^+$] 458.1033, found 458.1035.

2-((3-bromophenyl)sulfonyl)-*N*-methyl-3,3-diphenylacrylamide (53)

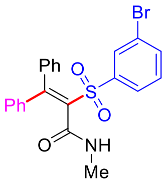

Standard conditions A was followed. White solid. Yield: 43.3 mg, 95%.

^{1}H NMR (400 MHz, CDCl$_3$) δ 7.75 (d, *J* = 7.9 Hz, 1H), 7.62 (d, *J* = 8.0 Hz, 1H), 7.51 (s, 1H), 7.36 (d, *J* = 7.4 Hz, 1H), 7.27 (td, *J* = 8.6, 4.4 Hz, 8H), 7.04 (d, *J* = 7.6 Hz, 2H), 6.25 (q, *J* = 5.0 Hz, 1H), 2.70 (d, *J* = 4.9 Hz, 3H). **^{13}C NMR (101 MHz, CDCl$_3$)** δ 163.64, 155.54, 142.89, 139.24, 138.91, 136.28, 136.21, 131.00, 130.26, 129.73, 129.09, 128.94, 128.40, 128.35, 127.72, 126.90, 122.31, 26.99. **HRMS (m/z, ESI$^+$):** Calcd for $C_{22}H_{18}NO_3SNa^+$ [M+Na$^+$] 478.0083, found 478.0083.

***N*-methyl-3,3-diphenyl-2-((3-(trifluoromethyl)phenyl)sulfonyl)acrylamide (54)**

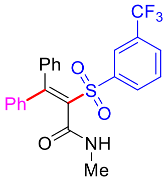

Standard conditions A was followed. White solid. Yield: 36.7 mg, 82%.

^{1}H NMR (400 MHz, CDCl$_3$) δ 8.09 (d, *J* = 7.9 Hz, 1H), 7.76 (d, *J* = 7.8 Hz, 1H), 7.65 (s, 1H), 7.59 (t, *J* = 7.8 Hz, 1H), 7.34 (t, *J* = 7.4 Hz, 1H), 7.30 – 7.23 (m, 7H), 7.03 (d, *J* = 7.5 Hz, 2H), 6.20 (q, *J* = 5.0 Hz, 1H), 2.71 (d, *J* =

4.9 Hz, 3H). **^{13}C NMR (101 MHz, $CDCl_3$)** δ 163.67, 155.88, 142.35, 138.98, 138.84, 136.27, 131.71, 130.94 (q, *J* = 33.5 Hz), 129.88 (q, *J* = 3.5 Hz), 129.83, 129.59, 129.02, 128.43, 128.33, 127.75, 125.10 (q, *J* = 4.0 Hz), 123.10 (q, *J* = 272.9 Hz), 26.98. **^{19}F NMR (376 MHz, $CDCl_3$)** δ -62.62. **HRMS (m/z, ESI$^+$):** Calcd for $C_{23}H_{18}F_3NO_3SNa^+$ [M+Na$^+$] 468.0852, found 468.0854.

2-((3,5-dimethylphenyl)sulfonyl)-*N*-methyl-3,3-diphenylacrylamide (55)

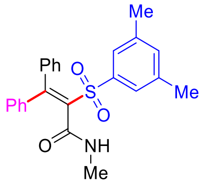

Standard conditions A was followed. White solid. Yield: 30.4 mg, 75%.

^{1}H NMR (400 MHz, $CDCl_3$) δ 7.33 – 7.22 (m, 8H), 7.18 (s, 2H), 7.11 (s, 1H), 7.09 – 7.03 (m, 2H), 6.25 (q, *J* = 4.9 Hz, 1H), 2.70 (d, *J* = 4.9 Hz, 3H), 2.27 (s, 6H). **^{13}C NMR (101 MHz, $CDCl_3$)** δ 163.90, 154.37, 140.64, 140.14, 139.25, 138.44, 136.78, 134.98, 129.40, 129.21, 128.36, 128.32, 128.30, 127.50, 125.82, 26.93, 21.10. **HRMS (m/z, ESI$^+$):** Calcd for $C_{24}H_{23}NO_3SNa^+$ [M+Na$^+$] 428.1291, found 428.1289.

***N*-methyl-2-(naphthalen-1-ylsulfonyl)-3,3-diphenylacrylamide (56)**

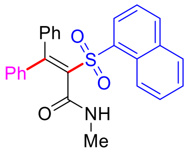

Standard conditions A was followed. Yellowish solid. Yield: 29.0 mg, 68%.

^{1}H NMR (400 MHz, $CDCl_3$) δ 9.26 (d, *J* = 8.8 Hz, 1H), 7.89 – 7.79 (m, 3H), 7.58 (t, *J* = 7.5 Hz, 1H), 7.46 (d, *J* = 7.4 Hz, 1H), 7.28 – 7.20 (m, 5H), 7.12 (t, *J* = 7.5 Hz, 1H), 7.04 (t, *J* = 7.8 Hz, 1H), 6.95 (t, *J* = 7.7 Hz, 2H), 6.65 (d, *J* = 7.2 Hz, 2H), 6.37 – 6.31 (m, 1H), 2.78 (d, *J* = 4.8 Hz, 3H). **^{13}C NMR (101 MHz, $CDCl_3$)** δ 163.23, 153.76, 139.89, 138.85, 136.53, 134.63, 134.28, 133.62, 130.07, 129.51, 128.94, 128.74, 128.55, 128.53, 128.48, 128.32, 128.23, 127.52, 126.85, 125.03, 123.85, 27.12. **HRMS (m/z, ESI$^+$):** Calcd for $C_{26}H_{21}NO_3S\ Na^+$ [M+Na$^+$] 450.1134, found 450.1136.

***N*-methyl-2-(naphthalen-2-ylsulfonyl)-3,3-diphenylacrylamide (57)**

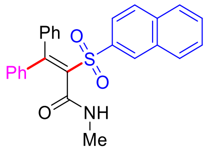

Standard conditions A was followed. White solid. Yield: 32.0 mg, 72%.

^{1}H NMR (400 MHz, $CDCl_3$) δ 7.94 – 7.82 (m, 4H), 7.75 (d, *J* = 8.1 Hz, 1H), 7.61 (t, *J* = 7.4 Hz, 1H), 7.54 (t, *J* = 7.5 Hz, 1H), 7.30 – 7.22 (m, 6H), 7.15 (t, *J* = 7.8 Hz, 2H), 7.02 (d, *J* = 7.4 Hz, 2H), 6.31 – 6.26 (m, 1H), 2.72 (d, *J* = 5.0 Hz, 3H). **^{13}C NMR (101 MHz, $CDCl_3$)** δ 163.99, 155.01, 139.78, 139.18, 137.80, 136.62, 135.08, 131.73, 130.08, 129.53, 129.51, 129.14, 129.06, 128.93, 128.64, 128.34, 127.85, 127.56, 127.23, 123.10, 27.00. **HRMS (m/z, ESI$^+$):** Calcd for $C_{26}H_{21}NO_3SNa^+$ [M+Na$^+$] 450.1134, found 450.1134.

***N*-methyl-3,3-diphenyl-2-(pyridin-3-ylsulfonyl)acrylamide (58)**

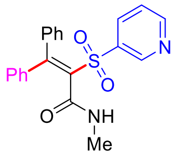

Standard conditions A was followed. White powder. Yield: 32.3 mg, 85%.
^{1}H NMR (400 MHz, CDCl$_3$) δ 8.74 (s, 1H), 8.65 (s, 1H), 8.07 (d, *J* = 8.1 Hz, 1H), 7.43 – 7.34 (m, 2H), 7.34 – 7.19 (m, 7H), 7.07 (dd, *J* = 7.7, 2.4 Hz, 2H), 6.37 (d, *J* = 5.0 Hz, 1H), 2.69 (dd, *J* = 5.2, 2.4 Hz, 3H). **^{13}C NMR (101 MHz, CDCl$_3$)** δ163.83, 155.96, 153.59, 148.83, 138.92, 138.85, 136.34, 136.23, 129.99, 129.20, 129.17, 128.53, 128.46, 127.94, 123.54, 27.05. **HRMS (m/z, ESI+):** Calcd for $C_{21}H_{19}N_2O_3S^+$ [M+H$^+$] 379.1111, found 379.1113.

_N_-methyl-3,3-diphenyl-2-(quinolin-8-ylsulfonyl)acrylamide (59)

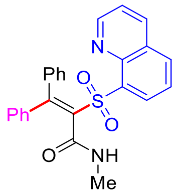

Standard conditions A was followed. White solid. Yield: 19.0 mg, 44%.
^{1}H NMR (400 MHz, CDCl$_3$) δ 9.17 (d, *J* = 4.3 Hz, 1H), 9.06 (q, *J* = 5.1 Hz, 1H), 8.31 (d, *J* = 8.3 Hz, 1H), 7.89 (d, *J* = 8.1 Hz, 1H), 7.66 (dd, *J* = 8.5, 4.3 Hz, 1H), 7.55 (d, *J* = 7.3 Hz, 1H), 7.25 – 7.12 (m, 6H), 7.05 (t, *J* = 7.5 Hz, 1H), 6.87 (t, *J* = 7.5 Hz, 2H), 6.54 (d, *J* = 7.5 Hz, 2H), 3.08 (d, *J* = 4.9 Hz, 3H). **^{13}C NMR (101 MHz, CDCl$_3$)** δ 163.91, 153.40, 150.29, 142.94, 140.76, 139.55, 137.63, 136.92, 136.89, 133.37, 132.15, 129.16, 128.99, 128.60, 128.22, 128.17, 127.98, 127.26, 125.75, 122.27, 27.13. Anal. Calcd for $C_{25}H_{20}N_2O_3S$: N, 6.54; C, 70.07; H, 4.70. Found: N, 6.33; C, 70.04; H, 4.91.

_N_-methyl-3,3-diphenyl-2-(thiophen-2-ylsulfonyl)acrylamide (60)

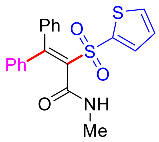

Standard conditions A was followed. Yellowish solid. Yield: 17.6 mg, 46%.
^{1}H NMR (400 MHz, CDCl$_3$) δ 7.64 (d, *J* = 5.7 Hz, 1H), 7.44 – 7.24 (m, 9H), 7.19 (d, *J* = 5.9 Hz, 2H), 6.99 (p, *J* = 4.3 Hz, 1H), 6.21 (s, 1H), 2.68 (d, *J* = 5.4 Hz, 3H). **^{13}C NMR (101 MHz, CDCl$_3$)** δ 163.84, 155.08, 141.87, 139.42, 139.10, 137.12, 134.90, 134.41, 129.62, 128.95, 128.77, 128.44, 128.36, 127.78, 127.36, 26.94. **HRMS (m/z, ESI$^+$):** Calcd for $C_{20}H_{17}NO_3S_2Na^+$ [M+Na$^+$] 406.0542, found 406.0540.

_N_-methyl-2-(methylsulfonyl)-3,3-diphenylacrylamide (61)

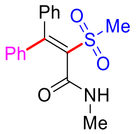

Standard conditions A was followed. White powder. Yield: 14.1 mg, 45%.
^{1}H NMR (400 MHz, CDCl$_3$) δ 7.41 – 7.26 (m, 10H), 6.20 – 6.06 (m, 1H), 3.11 (s, 3H), 2.63 (d, *J* = 4.9 Hz, 3H). **^{13}C NMR (101 MHz, CDCl$_3$)** δ 164.73, 154.88, 139.28, 137.18, 136.96, 129.75, 129.02, 128.90, 128.49, 128.43, 127.87, 44.52, 26.91. **HRMS (m/z, ESI$^+$):** Calcd for $C_{17}H_{17}NO_3SNa^+$ [M+Na$^+$] 338.0821, found 338.0821.

_N_-methyl-2-((4-(5-methyl-3-phenylisoxazol-4-yl)phenyl)sulfonyl)-3,3-diphenylacrylamide (62)

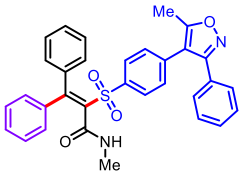

Standard conditions A was followed. White solid. Yield: 47.5 mg, 89%.

^{1}H NMR (600 MHz, CDCl$_3$) δ 7.61 (d, *J* = 8.7 Hz, 2H), 7.39 – 7.33 (m, 3H), 7.31 (t, *J* = 7.7 Hz, 2H), 7.29 – 7.22 (m, 6H), 7.19 (t, *J* = 7.6 Hz, 2H), 7.14 (d, *J* = 8.5 Hz, 2H), 7.04 (d, *J* = 7.5 Hz, 2H), 6.27 (t, *J* = 4.9 Hz, 1H), 2.67 (d, *J* = 4.8 Hz, 3H), 2.45 (s, 3H). **^{13}C NMR (101 MHz, CDCl$_3$)** δ 167.41, 163.90, 161.18, 155.08, 140.33, 139.65, 139.18, 136.79, 135.75, 129.85, 129.80, 129.73, 129.27, 128.77, 128.62, 128.58, 128.53, 128.47, 128.44, 127.72, 27.02, 11.86. **HRMS (m/z, ESI$^+$):** Calcd for $C_{32}H_{26}N_2O_4SNa^+$ [M+Na$^+$] 557.1505, found 557.1505.

2-((4-ethoxy-3-(1-methyl-7-oxo-3-propyl-6,7-dihydro-1H-pyrazolo[4,3-d]pyrimidin-5-yl)phenyl)sulfonyl)-*N*-methyl-3,3-diphenylacrylamide (63)

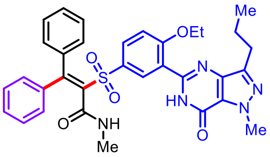

Standard conditions A was followed. White solid. Yield: 28.8 mg, 48%.

^{1}H NMR (600 MHz, CDCl$_3$) δ 10.78 (s, 1H), 8.50 (d, *J* = 2.5 Hz, 1H), 7.90 (dd, *J* = 8.8, 2.5 Hz, 1H), 7.28 – 7.16 (m, 8H), 7.12 (d, *J* = 7.0 Hz, 2H), 7.05 (d, *J* = 8.8 Hz, 1H), 6.22 (q, *J* = 5.0 Hz, 1H), 4.32 (q, *J* = 6.9 Hz, 2H), 4.25 (s, 3H), 2.92 (t, *J* = 7.6 Hz, 2H), 2.67 (d, *J* = 4.9 Hz, 3H), 1.84 (h, *J* = 7.5 Hz, 2H), 1.59 (t, *J* = 6.9 Hz, 3H), 1.00 (t, *J* = 7.3 Hz, 3H). **^{13}C NMR (101 MHz, CDCl$_3$)** δ 163.03, 158.77, 153.92, 152.82, 146.03, 145.58, 138.35, 138.14, 137.52, 136.07, 133.14, 131.61, 131.02, 128.70, 128.08, 127.78, 127.52, 127.44, 126.75, 123.54, 119.63, 112.02, 65.15, 37.35, 26.77, 26.02, 21.51, 13.62, 13.16. **HRMS (m/z, ESI$^+$):** Calcd for $C_{33}H_{33}N_5O_5SNa^+$ [M+Na$^+$] 634.2095, found 634.2094.

***N*-ethyl-3,3-diphenylacrylamide (64)**[3]

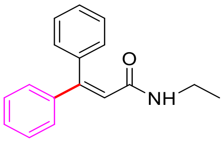

Standard conditions B was followed. White solid. Yield: 18.0 mg, 72%.

^{1}H NMR (400 MHz, CDCl$_3$) δ 7.41 (dd, *J* = 4.4, 2.4 Hz, 3H), 7.34 – 7.24 (m, 7H), 6.39 (s, 1H), 5.16 (s, 1H), 3.11 (p, *J* = 6.9 Hz, 2H), 0.81 (t, *J* = 7.2 Hz, 3H). **^{13}C NMR (101 MHz, CDCl$_3$)** δ 166.59, 149.17, 140.66, 138.54, 133.17, 129.39, 129.06, 128.89, 128.69, 128.56, 128.52, 128.41, 128.33, 128.17, 127.95, 127.66, 122.91, 35.03, 34.13, 14.21, 13.91.

***N*-benzyl-3,3-diphenylacrylamide (65)**[4]

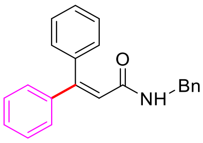

Standard conditions B was followed. White powder. Yield: 20.0 mg, 64%.

^{1}H NMR (400 MHz, CDCl$_3$) δ 7.39 – 7.27 (m, 6H), 7.27 – 7.16 (m, 7H), 6.94 (t, *J* = 3.7 Hz, 2H), 6.42 (s, 1H), 5.52 (t, *J* = 5.8 Hz, 1H), 4.27 (d, *J* = 5.6 Hz, 2H). **^{13}C NMR (101 MHz, CDCl$_3$)** δ 166.56, 149.72, 140.67, 138.44, 137.66, 129.34, 128.98, 128.75, 128.55, 128.43, 127.98, 127.74, 127.36, 122.49, 43.64.

3,3-diphenyl-*N*-(1-phenylethyl)acrylamide (66)

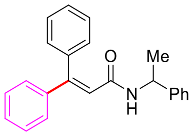

Standard conditions B was followed. White powder. Yield: 27.1 mg, 83%.

^{1}H NMR (400 MHz, CDCl$_3$) δ 7.38 (d, *J* = 5.1 Hz, 3H), 7.31 – 7.16 (m, 10H), 6.97 (d, *J* = 6.9 Hz, 2H), 6.41 (s, 1H), 5.42 (d, *J* = 8.0 Hz, 1H), 4.98 (p, *J* = 7.1 Hz, 1H), 1.17 (d, *J* = 6.8 Hz, 3H). **^{13}C NMR (101 MHz, CDCl$_3$)** δ 165.68, 149.15, 142.83, 140.53, 138.48, 129.38, 128.95, 128.87, 128.59, 128.55, 128.43, 127.90, 127.21, 126.06, 123.01, 48.66, 21.44. **HRMS (m/z, ESI$^+$):** Calcd for $C_{23}H_{21}NONa^+$ [M+Na$^+$] 350.1515, found 350.1513.

***N*-methyl-3-phenyl-3-(p-tolyl)acrylamide (67)**

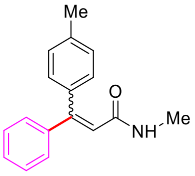

Standard conditions B was followed. White solid. Yield: 21.4 mg, 85%.

^{1}H NMR (400 MHz, CDCl$_3$) mixture of *Z*/*E* isomers: δ 7.36 – 7.11 (m, 6H), 7.05 (q, *J* = 7.9 Hz, 3H), 6.27 (s, 0.5H), 6.25 (s, 0.5H), 5.25 (s, 0.5H), 5.18 (s, 0.5H), 2.57 (d, *J* = 5.0 Hz, 1.5H), 2.54 (d, *J* = 4.9 Hz, 1.5H), 2.32 (s, 1.5H), 2.27 (s, 1.5H). **^{13}C NMR (101 MHz, CDCl$_3$) mixture of *Z*/*E* isomers:** δ 167.51, 167.48, 149.68, 149.53, 141.17, 139.04, 138.68, 138.50, 137.98, 135.44, 129.34, 129.32, 129.12, 128.83, 128.59, 128.50, 128.34, 128.08, 127.93, 122.24, 121.63, 26.24, 21.38, 21.23. **HRMS (m/z, ESI$^+$):** Calcd for $C_{17}H_{17}NONa^+$ [M+Na$^+$] 274.1202, found 274.1206.

3-(4-methoxyphenyl)-*N*-methyl-3-phenylacrylamide (68)

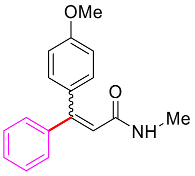

Standard conditions B was followed. White solid. Yield: 20.0 mg, 75%.

^{1}H NMR (400 MHz, CDCl$_3$) mixture of *Z*/*E* isomers: δ 7.47 – 7.22 (m, 5H), 7.19 (d, *J* = 8.8 Hz, 2H), 6.92 (d, *J* = 8.3 Hz, 1H), 6.83 (d, *J* = 8.4 Hz, 1H), 6.31 (s, 0.5H), 6.28 (s, 0.5H), 5.37 (s, 0.5H), 5.23 (s, 0.5H), 3.84 (s, 1.5H), 3.80 (s, 1.5H), 2.67 (d, *J* = 4.9 Hz, 1.5H), 2.61 (d, *J* = 4.8 Hz, 1.5H). **^{13}C NMR (101 MHz, CDCl$_3$) mixture of *Z*/*E* isomers:** δ 167.65, 167.52, 160.28, 159.80, 149.43, 149.24, 141.42, 138.74, 133.19, 130.93, 130.53, 129.35, 129.33, 128.84, 128.61, 128.51, 128.33, 128.19, 122.01, 120.64, 113.92, 113.76, 55.34, 55.30, 26.26, 26.23. **HRMS (m/z, ESI$^+$):** Calcd for $C_{17}H_{17}NO_2Na^+$ [M+Na$^+$] 290.1151, found 290.1152.

***N*-methyl-3-phenyl-3-(4-(trifluoromethoxy)phenyl)acrylamide (69)**

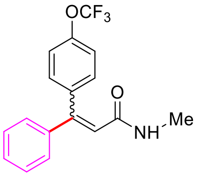

Standard conditions B was followed. Yellowish solid. Yield: 21.1 mg, 66%.

^{1}H NMR (400 MHz, CDCl$_3$) mixture of *Z*/*E* isomers: δ 7.43 – 7.40 (m, 1H), 7.39 – 7.21 (m, 7H), 7.15 (d, *J* = 8.4

Hz, 1H), 6.34 (d, J = 2.0 Hz, 1H), 5.46 (s, 0.5H), 5.29 (s, 0.5H), 2.70 (d, J = 4.9 Hz, 1.5H), 2.63 (d, J = 4.9 Hz, 1.5H). **^{13}C NMR (101 MHz, $CDCl_3$) mixture of *Z*/*E* isomers:** δ 167.02, 166.86, 149.57 (q, J = 2.0 Hz), 149.16 (q, J = 2.0 Hz), 149.04, 148.17, 140.69, 139.45, 137.97, 137.23, 131.01, 129.45, 129.26, 129.13, 128.86, 128.77, 128.52, 128.04, 123.10, 122.49, 120.70, 120.64, 120.45 (q, J = 258.6 Hz), 120.39 (q, J = 258.6 Hz), 26.23. **^{19}F NMR (376 MHz, $CDCl_3$) mixture of *Z*/*E* isomers:** δ -57.69, -57.77. **HRMS (m/z, ESI$^+$):** Calcd for $C_{17}H_{14}F_3NO_2Na^+$ [M+Na$^+$] 344.0869, found 344.0871.

(*Z*)-*N*-methyl-3-phenyl-3-(4-(trifluoromethyl)phenyl)acrylamide (*Z*-70)

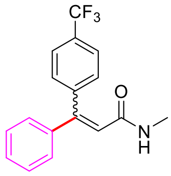

Standard conditions B was followed. Yellowish solid. Yield: 9.1 mg, 35%.
^{1}H NMR (400 MHz, $CDCl_3$) δ 7.65 (d, J = 8.0 Hz, 2H), 7.36 (m, 5H), 7.21 (d, J = 7.1 Hz, 2H), 6.38 (s, 1H), 5.42 (s, 1H), 2.72 (d, J = 3.7 Hz, 3H). **^{13}C NMR (101 MHz, $CDCl_3$)** δ 166.55, 149.55, 142.64, 140.40, 130.21 (q, J = 32.6 Hz), 129.76, 129.26, 128.59, 128.00, 125.23 (q, J = 3.8 Hz), 124.07 (q, J = 272.7 Hz), 122.40, 26.30. **^{19}F NMR (376 MHz, $CDCl_3$)** δ -62.54. **HRMS (m/z, ESI$^+$):** Calcd for $C_{17}H_{14}F_3NONa^+$ [M+Na$^+$] 328.0920, found 328.0920.

(*E*)-*N*-methyl-3-phenyl-3-(4-(trifluoromethyl)phenyl)acrylamide (*E*-70)

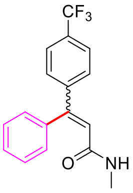

Standard conditions B was followed. Yellowish solid. Yield: 8.1 mg, 31%.
^{1}H NMR (400 MHz, $CDCl_3$) δ 7.57 (d, J = 8.1 Hz, 2H), 7.46 – 7.40 (m, 3H), 7.37 (d, J = 8.0 Hz, 2H), 7.27 – 7.24 (m, 2H), 6.41 (s, 1H), 5.25 (s, 1H), 2.65 (d, J = 4.2 Hz, 3H). **^{13}C NMR (101 MHz, $CDCl_3$)**δ 166.89, 148.14, 144.43, 137.70, 130.69 (q, J = 32.5 Hz), 129.27, 128.96, 128.80, 128.32, 125.38 (q, J = 3.8 Hz), 124.18, 123.97 (q, J = 273.7 Hz), 26.25. **^{19}F NMR (376 MHz, $CDCl_3$)** δ -62.65. **HRMS (m/z, ESI$^+$):** Calcd for $C_{17}H_{14}F_3NONa^+$ [M+Na$^+$] 328.0920, found 328.0919.

***N*-methyl-3-phenyl-3-(*m*-tolyl)acrylamide (71)**

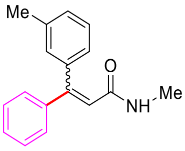

Standard conditions B was followed. White solid. Yield: 21.1 mg, 84%.
^{1}H NMR (400 MHz, $CDCl_3$) mixture of *Z*/*E* isomers: δ 7.44 – 7.11 (m, 7H), 7.08 – 7.03 (m, 2H), 6.35 (d, J = 1.7 Hz, 1H), 5.28 (s, 1H), 2.62 (d, J = 5.0 Hz, 3H), 2.35 (s, 1.5H), 2.31 (s, 1.5H). **^{13}C NMR (101 MHz, $CDCl_3$) mixture of *Z*/*E* isomers:** δ 167.45, 167.41, 149.74, 149.60, 140.90, 140.86, 138.61, 138.42, 138.40, 138.01, 129.79, 129.69, 129.38, 129.33, 128.85, 128.62, 128.58, 128.52, 128.37, 128.27, 127.98, 126.42, 125.26, 122.46, 122.37, 26.24, 26.23, 21.44. **HRMS (m/z, ESI+):** Calcd for $C_{17}H_{17}NONa^+$ [M+Na$^+$] 274.1202, found 274.1204.

***N*-methyl-3-(naphthalen-1-yl)-3-phenylacrylamide (72)**

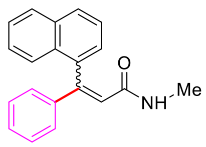

Standard conditions B was followed. Yellowish solid. Yield: 14.3 mg, 50%.

^{1}H NMR (400 MHz, CDCl$_3$) mixture of *Z*/*E* isomers: δ 7.67 – 7.52 (m, 3H), 7.49 – 7.27 (m, 9H), 6.44 (s, 0.5H), 6.37 (s, 0.5H), 5.39 (q, *J* = 5.0 Hz 0.5H), 5.27 (q, *J* = 5.0 Hz, 0.5H), 2.67 (d, *J* = 4.9 Hz, 1.5H), 2.63 (d, *J* = 4.9 Hz, 1.5H). **^{13}C NMR (101 MHz, CDCl$_3$) mixture of *Z*/*E* isomers:** δ 167.42, 167.35, 149.48, 149.17, 141.71, 141.23, 141.03, 140.28, 139.66, 138.45, 137.44, 129.99, 129.38, 128.96, 128.91, 128.88, 128.71, 128.65, 128.44, 128.17, 127.69, 127.67, 127.12, 127.08, 127.07, 127.05, 122.49, 122.28, 26.31, 26.28. Anal. Calcd for $C_{20}H_{17}NO$: N, 4.87; C, 83.59; H, 5.96. Found: N, 4.31; C, 83.57; H, 6.34.

_N_-methyl-3-(1-methyl-1*H*-indol-5-yl)-3-phenylacrylamide (73)[5]

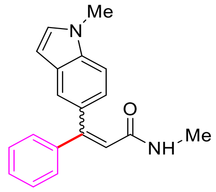

Standard conditions B was followed. Yellow solid. Yield: 13.0 mg, 50%.

^{1}H NMR (400 MHz, CDCl$_3$) mixture of *Z*/*E* isomers: δ 7.51 (d, *J* = 1.6 Hz, 0.5H), 7.42 – 7.39 (m, 2H), 7.39 – 7.14 (m, 5H), 7.14 – 7.09 (m, 1H), 7.04 (d, *J* = 3.1 Hz, 0.5H), 6.48 (d, *J* = 3.1 Hz, 0.5H), 6.42 (d, *J* = 3.2 Hz, 0.5H), 6.40 (s, 0.5H), 6.38 (s, 0.5H), 5.24 (s, 0.5H), 5.17 (s, 0.5H), 3.84 (s, 1.5H), 3.78 (s, 1.5H), 2.62 (d, *J* = 4.9 Hz, 1.5H), 2.56 (d, *J* = 4.9 Hz, 1.5H). **^{13}C NMR (101 MHz, CDCl$_3$) mixture of *Z*/*E* isomers:** δ 168.03, 167.83, 151.03, 150.58, 142.00, 139.52, 136.98, 136.65, 132.30, 129.85, 129.76, 129.52, 129.15, 128.68, 128.54, 128.50, 128.38, 128.30, 128.23, 123.19, 122.19, 122.12, 121.68, 121.50, 120.70, 109.54, 109.13, 101.74, 101.48, 33.04, 32.99, 26.25.

3-(dibenzo[b,d]thiophen-4-yl)-*N*-methyl-3-phenylacrylamide (74)

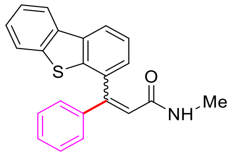

Standard conditions B was followed. Yellow solid. Yield: 23.7 mg, 69%.

^{1}H NMR (400 MHz, CDCl$_3$) mixture of *Z*/*E* isomers: δ 8.90 (s, 0.3H), 8.76 (s, 0.7H), 8.13 – 8.08 (m, 1H), 8.03 (s, 0.7H), 7.88 (s, 0.3H), 7.82 – 7.67 (m, 2H), 7.55 (t, *J* = 7.6 Hz, 1H), 7.45 (d, *J* = 4.0 Hz, 1H), 7.40 – 7.18 (m, 5H), 6.54 (s, 0.3H), 6.46 (s, 0.7H), 5.82 (s, 0.7H), 5.51 (s, 0.3H), 2.77 – 2.59 (m, 3H). **^{13}C NMR (101 MHz, CDCl$_3$) mixture of *Z*/*E* isomers:** δ 166.79, 166.42, 151.16, 149.43, 147.89, 147.58, 146.61, 140.76, 137.54, 136.31, 135.43, 133.73, 132.02, 130.25, 129.95, 129.35, 129.29, 129.18, 129.09, 128.92, 128.67, 128.32, 128.22, 128.19, 127.57, 127.45, 127.39, 127.26, 126.97, 123.97, 123.01, 26.32. **HRMS (m/z, ESI$^+$):** Calcd for $C_{22}H_{17}NOSNa^+$ [M+Na$^+$] 366.0923, found 366.0923.

4-(3-(methylamino)-3-oxo-1-phenylprop-1-en-1-yl)benzyl 2-(3-benzoylphenyl)propanoate (75)

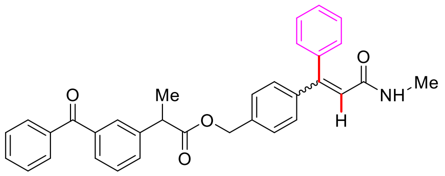

Standard conditions B was followed. Yellowish solid. Yield: 60.8 mg, 81%.

^{1}H NMR (400 MHz, CDCl$_3$) mixture of *Z*/*E* isomers: δ 7.79 – 7.74 (m, 3H), 7.66 (d, *J* = 7.6 Hz, 1H), 7.59 – 7.52 (m, 2H), 7.48 – 7.28 (m, 6H), 7.26 – 7.13 (m, 6H), 6.33 (s, 1H), 5.66 (q, *J* = 4.9 Hz, 0.5H), 5.48 (q, *J* = 5.0 Hz, 0.5H), 5.16 (s, 1H), 5.10 (s, 1H), 3.87 – 3.83 (m, 1H), 2.62 (t, *J* = 4.6 Hz, 3H), 1.58 – 1.54 (m, 3H). **^{13}C NMR (101 MHz, CDCl$_3$) mixture of *Z*/*E* isomers:** δ 196.54, 196.52, 173.84, 173.80, 167.25, 167.18, 149.57, 149.15, 140.90, 140.81, 140.72, 140.61, 138.53, 138.31, 137.88, 137.39, 136.51, 135.96, 132.63, 132.61, 131.61, 130.07, 129.59, 129.31, 129.25, 129.22, 129.14, 129.10, 128.92, 128.63, 128.60, 128.58, 128.39, 128.37, 128.17, 128.06, 127.83, 127.72, 122.58, 122.34, 66.22, 66.10, 45.40, 45.38, 18.45, 18.36. **HRMS (m/z, ESI$^+$):** Calcd for $C_{33}H_{29}NO_4Na^+$ [M+Na$^+$] 526.1989, found 526.1989.

4-(3-(methylamino)-3-oxo-1-phenylprop-1-en-1-yl)phenyl 4-(*N*,*N*-dipropylsulfamoyl)benzoate (76)

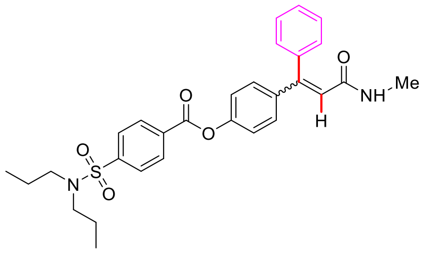

Standard conditions B was followed. Yellowish solid. Yield: 17.5 mg, 34%.

^{1}H NMR (400 MHz, CDCl$_3$) mixture of *Z*/*E* isomers: δ 8.32 (t, *J* = 7.6 Hz, 2H), 8.04 – 7.89 (m, 2H), 7.46 – 7.31 (m, 5H), 7.30 – 7.27 (m, 3H), 7.18 (d, *J* = 8.4 Hz, 1H), 6.39 (s, 0.5H), 6.37 (s, 0.5H), 5.39 (q, *J* = 5.3 Hz, 0.5H), 5.26 (q, *J* = 5.0 Hz, 0.5H), 3.25 – 3.03 (m, 4H), 2.71 (d, *J* = 4.9 Hz, 1.5H), 2.64 (d, *J* = 5.0 Hz, 1.5H), 1.62 – 1.52 (m, 4H), 0.91 – 0.87 (m, 6H). **^{13}C NMR (101 MHz, CDCl$_3$) mixture of *Z*/*E* isomers:** δ 167.15, 163.71, 151.05, 150.68, 148.99, 148.51, 145.05, 140.71, 138.91, 138.16, 136.57, 132.65, 132.58, 130.85, 130.83, 130.78, 129.34, 129.29, 129.08, 128.79, 128.74, 128.49, 128.13, 127.24, 127.21, 122.83, 122.64, 121.60, 121.51, 49.96, 49.94, 26.29, 26.26, 21.97, 21.94, 11.19. **HRMS (m/z, ESI$^+$):** Calcd for $C_{29}H_{32}N_2O_5SNa^+$ [M+Na$^+$] 543.1924, found 543.1927.

(8S,9R,13R,14R)-13-methyl-17-oxo-7,8,9,11,12,13,14,15,16,17-decahydro-6H-cyclopenta[a]phenanthren-3-yl 4-(3-(methylamino)-3-oxo-1-phenylprop-1-en-1-yl)benzoate (77)

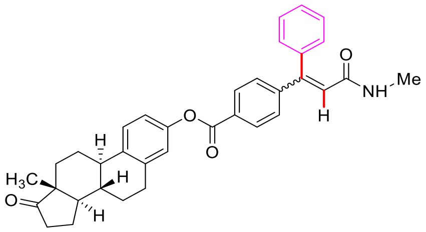

Standard conditions B was followed. Brown solid. Yield: 22.0 mg, 41%.

^{1}H NMR (400 MHz, CDCl$_3$) mixture of *Z*/*E* isomers: δ 8.14 (d, *J* = 8.0 Hz, 1.2H), 8.05 (d, *J* = 8.2 Hz, 0.8H), 7.40 – 7.23 (m, 6H), 7.22 – 7.16 (m, 2H), 6.97 – 6.78 (m, 2H), 6.38 (s, 0.4H), 6.33 (s, 0.6H), 5.41 (q, *J* = 5.0 Hz, 0.6H), 5.22 (q, *J* = 4.8 Hz, 0.4H), 2.91 – 2.83 (m, 2H), 2.65 (d, *J* = 4.9 Hz, 1.8H), 2.58 (d, *J* = 4.9 Hz, 1.2H), 2.44 (dd, *J* = 18.9, 8.6 Hz, 1H), 2.38 – 2.21 (m, 2H), 2.16 – 1.85 (m, 4H), 1.69 – 1.32 (m, 6H), 0.85 (s, 3H). **^{13}C NMR**

(101 MHz, $CDCl_3$) mixture of *Z*/*E* isomers: δ 166.91, 166.50, 165.11, 165.00, 149.66, 148.79, 148.74, 148.30, 145.92, 144.43, 140.25, 138.15, 137.76, 137.53, 130.24, 130.17, 129.74, 129.55, 129.36, 129.30, 129.24, 128.94, 128.82, 128.58, 128.15, 127.96, 126.54, 124.39, 122.44, 121.70, 121.66, 118.86, 118.82, 50.44, 47.99, 44.20, 38.02, 35.90, 31.57, 29.47, 26.37, 26.34, 25.80, 21.62, 13.86. **HRMS (m/z, ESI^+):** Calcd for $C_{35}H_{35}NO_4Na^+$ [$M+Na^+$] 556.2458, found 556.2461.

***N*-methyl-3-phenyl-3-(*p*-tolyl)acrylamide (78)**

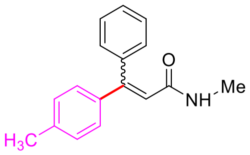

Standard conditions B was followed. White solid. Yield: 21.4 mg, 85%.

^{1}H NMR (400 MHz, $CDCl_3$) mixture of *Z*/*E* isomers: δ 7.40 (d, *J* = 5.7 Hz, 2H), 7.31 – 7.20 (m, 4H), 7.13 (q, *J* = 7.7 Hz, 3H), 6.35 (s, 0.5H), 6.33 (s, 0.5H), 5.35 (s, 0.5H), 5.28 (s, 0.5H), 2.64 (d, *J* = 4.5 Hz, 1.5H), 2.61 (d, *J* = 4.5 Hz, 1.5H), 2.39 (s, 1.5H), 2.34 (s, 1.5H). **^{13}C NMR (101 MHz, $CDCl_3$) mixture of *Z*/*E* isomers:** δ 167.50, 167.47, 149.68, 149.54, 141.17, 139.03, 138.68, 138.48, 137.99, 135.45, 129.34, 129.31, 129.12, 128.82, 128.58, 128.49, 128.34, 128.07, 127.92, 122.24, 121.62, 26.23, 21.37, 21.23. **HRMS (m/z, ESI^+):** Calcd for $C_{17}H_{17}NONa^+$ [$M+Na^+$] 274.1202, found 274.1203.

3-([1,1'-biphenyl]-4-yl)-*N*-methyl-3-phenylacrylamide (79)

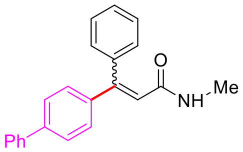

Standard conditions B was followed. Yellowish solid. Yield: 25.7 mg, 82%.

^{1}H NMR (400 MHz, $CDCl_3$) mixture of *Z*/*E* isomers: δ 7.67 – 7.59 (m, 2H), 7.57 (d, *J* = 7.4 Hz, 1H), 7.53 (d, *J* = 8.3 Hz, 1H), 7.48 – 7.26 (m, 10H), 6.43 (s, 0.5H), 6.36 (s, 0.5H), 5.45 (s, 0.5H), 5.34 (s, 0.5H), 2.66 (d, *J* = 4.9 Hz, 1.5H), 2.63 (d, *J* = 4.9 Hz, 1.5H). **^{13}C NMR (101 MHz, $CDCl_3$) mixture of *Z*/*E* isomers:** δ 167.40, 167.34, 149.52, 149.20, 141.69, 141.20, 141.07, 140.30, 140.27, 139.70, 138.47, 137.47, 130.00, 129.38, 128.96, 128.92, 128.89, 128.69, 128.64, 128.46, 128.44, 128.18, 127.68, 127.11, 127.07, 127.05, 122.47, 122.27, 26.31, 26.28. **HRMS (m/z, ESI^+):** Calcd for $C_{22}H_{19}NONa^+$ [$M+Na^+$] 336.1359, found 336.1363.

3-(4-methoxyphenyl)-*N*-methyl-3-phenylacrylamide (80)

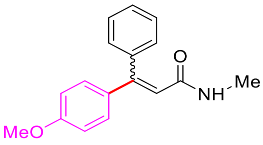

Standard conditions B was followed. Yellowish solid. Yield: 13.5 mg, 51%.

^{1}H NMR (400 MHz, $CDCl_3$) mixture of *Z*/*E* isomers: δ 7.44 – 7.37 (m, 1.5H), 7.34 – 7.28 (m, 1.5H), 7.33 – 7.28 (m, 2H), 7.19 (d, *J* = 9.0 Hz, 2H), 6.92 (d, *J* = 7.6 Hz, 1H), 6.83 (d, *J* = 7.8 Hz, 1H), 6.31 (s, 0.5H), 6.29 (s, 0.5H), 5.33 (s, 0.5H), 5.19 (s, 0.5H), 3.85 (s, 1.5H), 3.80 (s, 1.5H), 2.67 (d, *J* = 4.9 Hz, 1.5H), 2.61 (d, *J* = 4.9 Hz, 1.5H). **^{13}C NMR (101 MHz, $CDCl_3$) mixture of *Z*/*E* isomers:** δ 167.66, 167.53, 160.29, 159.82, 149.41, 149.22, 141.41, 138.73, 133.18, 130.93, 130.53, 129.35, 129.33, 128.85, 128.63, 128.53, 128.34, 128.19, 122.03, 120.66, 113.94, 113.76, 55.35, 55.31, 26.27, 26.24. **HRMS (m/z, ESI^+):** Calcd for $C_{17}H_{17}NO_2Na^+$ [$M+Na^+$] 290.1151, found 290.1150.

3-(4-fluorophenyl)-*N*-methyl-3-phenylacrylamide (81)

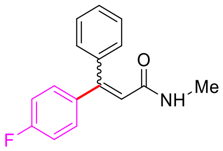

Standard conditions B was followed. White solid. Yield: 18.2 mg, 71%.

^{1}H NMR (400 MHz, $CDCl_3$) mixture of *Z*/*E* isomers: δ 7.43 – 7.39 (m, 1.4H), 7.36 – 7.29 (m, 1.6H), 7.28 – 7.19 (m, 4H), 7.12 – 7.04 (m, 1H), 7.01 – 6.97 (m, 1H), 6.32 (s, 0.5H), 6.31 (s, 0.5H), 5.44 (s, 0.5H), 5.28 (s, 0.5H), 2.69 (d, *J* = 4.9 Hz, 1.5H), 2.62 (d, *J* = 4.9 Hz, 1.5H). **^{13}C NMR (101 MHz, $CDCl_3$) mixture of *Z*/*E* isomers:** δ 167.22, 167.13, 163.16 (d, *J* = 250.5 Hz), 162.74 (d, *J* = 249.5 Hz), 149.14, 148.56, 140.94, 138.29, 136.97 (d, *J* = 3.2 Hz), 134.50 (d, *J* = 3.6 Hz), 131.30 (d, *J* = 8.1 Hz), 129.80 (d, *J* = 8.4 Hz), 129.29, 129.04, 128.75, 128.71, 128.46, 128.06, 122.33, 122.31, 115.55 (d, *J* = 9.9 Hz), 115.34 (d, *J* = 10.0 Hz), 26.25. **^{19}F NMR (376 MHz, $CDCl_3$) mixture of *Z*/*E* isomers:** δ -112.52, -112.83. **HRMS (m/z, ESI$^+$):** Calcd for $C_{16}H_{14}FNONa^+$ [M+Na$^+$] 278.0952, found 278.0951.

3-(4-chlorophenyl)-*N*-methyl-3-phenylacrylamide (82)

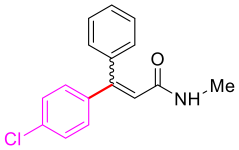

Standard conditions B was followed. Yellowish solid. Yield: 22.3 mg, 82%.

^{1}H NMR (400 MHz, $CDCl_3$) mixture of *Z*/*E* isomers: δ 7.44 – 7.30 (m, 4H), 7.22 (m, 5H), 6.33 (s, 0.4H), 6.32 (s, 0.6H), 5.53 (s, 0.6H), 5.32 (s, 0.4H), 2.69 (d, *J* = 4.9 Hz, 1.6H), 2.62 (d, *J* = 4.9 Hz, 1.4H). **^{13}C NMR (101 MHz, $CDCl_3$) mixture of *Z*/*E* isomers:** δ 167.10, 166.92, 149.15, 148.42, 140.72, 139.35, 138.02, 137.10, 134.94, 134.42, 130.83, 129.28, 129.09, 128.80, 128.72, 128.62, 128.48, 128.06, 122.73, 122.33, 26.28, 26.24. **HRMS (m/z, ESI$^+$):** Calcd for $C_{16}H_{14}ClNO\ Na^+$ [M+Na$^+$] 294.0656, found 294.0657.

3-(4-bromophenyl)-*N*-methyl-3-phenylacrylamide (83)

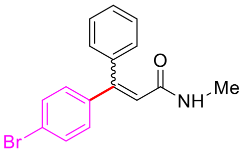

Standard conditions B was followed. White solid. Yield: 22.2 mg, 70%.

^{1}H NMR (400 MHz, $CDCl_3$) mixture of *Z*/*E* isomers: δ 7.55 – 7.48 (m, 1H), 7.46 – 7.38 (m, 2H), 7.38 – 7.25 (m, 2H), 7.24 – 7.20 (m, 2H), 7.15 – 7.10 (m, 2H), 6.34 (s, 0.4H), 6.32 (s, 0.6H), 5.52 (s, 0.6H), 5.33 (s, 0.4H), 2.70 (d, *J* = 4.9 Hz, 1.8H), 2.62 (d, *J* = 4.9 Hz, 1.2H). **^{13}C NMR (101 MHz, $CDCl_3$) mixture of *Z*/*E* isomers:** δ 167.11, 166.92, 149.20, 148.50, 140.63, 139.82, 137.93, 137.58, 131.59, 131.57, 131.12, 129.58, 129.30, 129.11, 128.83, 128.73, 128.49, 128.06, 123.25, 122.76, 122.69, 122.28, 26.30, 26.25. **HRMS (m/z, ESI$^+$):** Calcd for $C_{16}H_{14}BrNONa^+$ [M+Na$^+$] 338.0151, found 338.0153.

***N*-methyl-3-phenyl-3-(4-(trifluoromethyl)phenyl)acrylamide (84)**

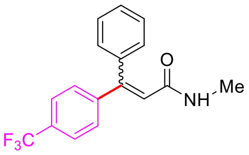

Standard conditions B was followed. Yellowish solid. Yield: 24.6 mg, 81%.

^{1}H NMR (400 MHz, $CDCl_3$) mixture of *Z*/*E* isomers: δ 7.63 (d, *J* = 8.0 Hz, 1H), 7.56 (d, *J* = 8.4 Hz, 1H), 7.41 (s,

1H), 7.39 – 7.29 (m, 4H), 7.27 – 7.20 (m, 2H), 6.40 (s, 0.5H), 6.38 (s, 0.5H), 5.67 (s, 0.5H), 5.40 (s, 0.5H), 2.70 (d, *J* = 8.2 Hz, 1.5H), 2.64 (d, *J* = 7.5 Hz, 1.5H). **^{13}C NMR (101 MHz, $CDCl_3$) mixture of *Z*/*E* isomers:** δ 166.92, 166.53, 149.54, 148.32, 144.52, 142.73, 140.45, 137.72, 130.65 (q, *J* = 32.6 Hz), 130.08 (q, *J* = 32.6 Hz), 129.75, 129.27, 129.20, 128.91, 128.74, 128.55, 128.34, 127.98, 125.36 (q, *J* = 3.7 Hz), 125.13 (q, *J* = 3.8 Hz), 124.11 (q, *J* = 273.7 Hz), 124.03, 123.98 (q, *J* = 273.7 Hz), 122.37, 26.23. **^{19}F NMR (376 MHz, $CDCl_3$) mixture of *Z*/*E* isomers:** δ -62.54, -62.66. **HRMS (m/z, ESI$^+$):** Calcd for $C_{17}H_{14}F_3NONa^+$ [M+Na$^+$] 328.0920, found 328.0922.

methyl 4-(3-(methylamino)-3-oxo-1-phenylprop-1-en-1-yl)benzoate (85)

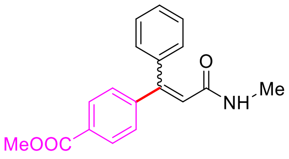

Standard conditions B was followed. White solid. Yield: 28.6 mg, 97%.

^{1}H NMR (400 MHz, $CDCl_3$) mixture of *Z*/*E* isomers: δ 8.06 (d, *J* = 8.0 Hz, 1.2H), 7.97 (d, *J* = 8.1 Hz, 0.8H), 7.43 – 7.40 (m, 1H), 7.37 – 7.27 (m, 4H), 7.23 (m, 2H), 6.42 (s, 0.4H), 6.39 (s, 0.6H), 5.55 (s, 0.6H), 5.41 (s, 0.4H), 3.93 (s, 1.8H), 3.91 (s, 1.2H), 2.67 (d, *J* = 4.9 Hz, 1.8H), 2.64 (d, *J* = 4.9 Hz, 1.2H). **^{13}C NMR (101 MHz, $CDCl_3$) mixture of *Z*/*E* isomers:** δ 166.97, 166.76, 166.74, 166.66, 149.35, 148.51, 145.32, 143.67, 140.32, 137.84, 130.24, 129.91, 129.64, 129.62, 129.42, 129.29, 129.14, 128.84, 128.73, 128.53, 128.00, 127.96, 124.04, 122.60, 52.26, 26.27, 26.25. **HRMS (m/z, ESI$^+$):** Calcd for $C_{18}H_{17}NO_3Na^+$ [M+Na$^+$] 318.1101, found 318.1102.

3-(3-bromophenyl)-*N*-methyl-3-phenylacrylamide (86)

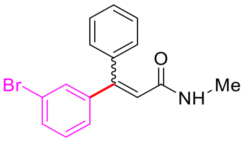

Standard conditions B was followed. White solid. Yield: 23.5 mg, 74%.

^{1}H NMR (400 MHz, $CDCl_3$) mixture of *Z*/*E* isomers: δ 7.52-7.44 (m, 1H), 7.43 – 7.28 (m, 4H), 7.27-7.17 (m, 4H), 6.34 (s, 0.5H), 6.33 (s, 0.5H), 5.50 (s, 0.5H), 5.35 (s, 0.5H), 2.69 (d, *J* = 5.0 Hz, 1.5H), 2.63 (d, *J* = 4.9 Hz, 1.5H). **^{13}C NMR (101 MHz, $CDCl_3$) mixture of *Z*/*E* isomers:** δ 166.95, 166.79, 148.66, 148.22, 143.09, 140.77, 140.38, 137.76, 132.05, 131.81, 131.47, 130.89, 129.93, 129.28, 129.14, 128.86, 128.74, 128.54, 128.15, 128.00, 126.71, 123.47, 122.71, 122.62, 122.48, 26.30, 26.26. **HRMS (m/z, ESI$^+$):** Calcd for $C_{16}H_{14}BrNONa^+$ [M+Na$^+$] 338.0151, found 338.0154.

3-(3-iodophenyl)-*N*-methyl-3-phenylacrylamide (87)

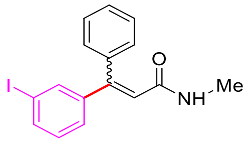

Standard conditions B was followed. Yellowish solid. Yield: 23.7 mg, 65%.

^{1}H NMR (400 MHz, $CDCl_3$) mixture of *Z*/*E* isomers: δ 7.71 (dt, *J* = 7.9, 1.4 Hz, 0.5H), 7.65 (dt, *J* = 7.8, 1.5 Hz, 0.5H), 7.61 (t, *J* = 1.7 Hz, 0.5H), 7.57 (t, *J* = 1.7 Hz, 0.5H), 7.42 – 7.39 (m, 1H), 7.35 – 7.28 (m, 2H), 7.28 – 7.16 (m, 3H), 7.13 (t, *J* = 7.8 Hz, 0.5H), 7.04 (t, *J* = 7.8 Hz, 0.5H), 6.33 (s, 0.5H), 6.31 (s, 0.5H), 5.46 (s, 0.5H), 5.32 (s, 0.5H), 2.69 (d, *J* = 4.9 Hz, 1.6H), 2.63 (d, *J* = 4.9 Hz, 1.4H). **^{13}C NMR (101 MHz, $CDCl_3$) mixture of *Z*/*E* isomers:** δ 166.95, 166.81, 148.51, 148.13, 143.18, 140.81, 140.39, 137.81, 137.75, 137.42, 136.72, 130.06, 129.28, 129.13, 128.86, 128.76, 128.74, 128.54, 128.01, 127.41, 123.43, 122.71, 94.43, 94.22, 26.31, 26.27. **HRMS (m/z, ESI$^+$):** Calcd for $C_{16}H_{14}INONa^+$ [M+Na$^+$] 386.0012, found 386.0012.

3-(4-chlorophenyl)-*N*-methyl-3-phenylacrylamide (88)

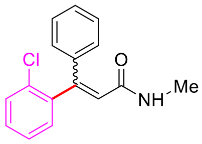

Standard conditions B was followed. White solid. Yield: 21.9 mg, 81%.

^{1}H NMR (400 MHz, $CDCl_3$) mixture of *Z*/*E* isomers: δ 7.47 – 7.37 (m, 1H), 7.35 – 7.29 (m, 4H), 7.28 – 7.22 (m, 4H), 6.51 (s, 0.6H), 6.10 (s, 0.4H), 5.61 (s, 0.6H), 5.49 (s, 0.4H), 2.70 (d, *J* = 5.0 Hz, 1.2H), 2.67 (d, *J* = 4.9 Hz, 1.8H). **^{13}C NMR (101 MHz, $CDCl_3$) mixture of *Z*/*E* isomers:** δ 167.44, 166.28, 147.44, 146.71, 140.60, 138.90, 137.82, 137.73, 132.93, 132.76, 131.05, 130.73, 130.11, 129.74, 129.47, 129.41, 129.05, 128.95, 128.63, 128.58, 128.29, 127.05, 126.99, 126.75, 126.35, 122.80, 26.31, 26.26. **HRMS (m/z, ESI$^+$):** Calcd for $C_{16}H_{14}ClNONa^+$ [M+Na$^+$] 294.0656, found 294.0660.

3-(dibenzo[b,d]furan-3-yl)-*N*-methyl-3-phenylacrylamide (89)

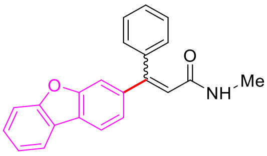

Standard conditions B was followed. Yellow solid. Yield: 17.8 mg, 54%.

^{1}H NMR (400 MHz, $CDCl_3$) mixture of *Z*/*E* isomers: δ 7.97 (d, *J* = 7.9 Hz, 1.1H), 7.92 (d, *J* = 7.8 Hz, 0.5H), 7.85 (d, *J* = 8.1 Hz, 0.4H), 7.60 – 7.39 (m, 4H), 7.31 (m, 6H), 6.48 (s, 0.4H), 6.43 (s, 0.6H), 5.38 (s, 0.6H), 5.32 (s, 0.4H), 2.65 (d, *J* = 4.9 Hz, 1.2H), 2.62 (d, *J* = 4.9 Hz, 1.8H). **^{13}C NMR (101 MHz, $CDCl_3$) mixture of *Z*/*E* isomers:** δ 167.28, 156.84, 156.69, 156.19, 156.10, 149.51, 140.94, 140.24, 138.50, 137.68, 129.44, 129.04, 128.76, 128.73, 128.46, 128.13, 127.66, 127.64, 124.86, 124.54, 124.29, 123.80, 123.73, 123.02, 122.97, 122.88, 122.83, 122.78, 120.88, 120.84, 120.68, 120.35, 112.68, 111.83, 111.76, 111.44, 26.29. **HRMS (m/z, ESI$^+$):** Calcd for $C_{22}H_{17}NO_2Na^+$ [M+Na$^+$] 350.1151, found 350.1152.

3-(4-bromophenyl)-*N*-methyl-3-(pyridin-3-yl)acrylamide (90)

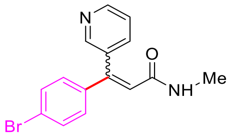

Standard conditions B was followed. Brown solid. Yield: 15.0 mg, 47%.

^{1}H NMR (400 MHz, $CDCl_3$) mixture of *Z*/*E* isomers: δ 8.61 (d, *J* = 4.3 Hz, 0.5H), 8.57 (d, *J* = 4.6 Hz, 0.5H), 8.54 (s, 0.5H), 8.45 (s, 0.5H), 7.61 – 7.41 (m, 3H), 7.33 (dd, *J* = 7.9, 4.9 Hz, 0.5H), 7.27 – 7.23 (m, 0.5H), 7.13 (d, *J* = 8.1 Hz, 1H), 7.10 (d, *J* = 8.4 Hz, 1H), 6.37 (s, 0.5H), 6.35 (s, 0.5H), 5.66 (s, 0.5H), 5.59 (s, 0.5H), 2.73 (m, 3H). **^{13}C NMR (101 MHz, $CDCl_3$) mixture of *Z*/*E* isomers:** δ 166.24, 166.04, 150.00, 149.81, 149.49, 148.67, 146.58, 146.02, 139.34, 136.97, 136.51, 136.44, 135.48, 134.21, 131.85, 130.96, 129.53, 123.72, 123.65, 123.32, 123.21, 123.16, 123.13, 26.33, 26.31. **HRMS (m/z, ESI$^+$):** Calcd for $C_{15}H_{14}BrN_2ONa^+$ [M+Na$^+$] 317.0284, found 317.0286.

***N*-methyl-3,3-diphenyl-2-(trifluoromethyl) acrylamide (91)**

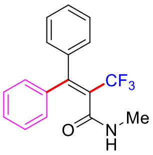

Standard conditions C was followed. White solid. Yield: 27.4 mg, 45% yield.

^{1}H NMR (400 MHz, CDCl$_3$) δ 7.34 (m, 3H), 7.29 (s, 5H), 7.21 (m, 2H), 5.63 (d, *J* = 6.9 Hz, 1H), 2.61 (d, *J* = 4.9 Hz, 3H). **^{13}C NMR (101 MHz, CDCl$_3$)** δ 164.7, 151.9 (q, *J* = 3.7 Hz), 139.3, 138.3, 129.3, 128.6, 128.6, 128.4, 128.3, 128.3, 128.2, 126.8 (q, *J* = 30.3 Hz), 122.1 (q, *J* = 275.5 Hz), 26.8. **^{19}F NMR (376 MHz, CDCl$_3$)** δ -55.30. **HRMS (m/z, ESI$^+$):** Calculated for $C_{17}H_{14}F_3NONa^+$ [M+Na$^+$] 328.0920, found 328.0922.

(*E*)-*N*-methyl-3-phenyl-3-(*p*-tolyl)-2-(trifluoromethyl) acrylamide (92)

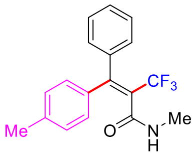

Standard conditions C was followed. White solid. Yield: 30.0 mg, 47% yield.

^{1}H NMR (400 MHz, CDCl$_3$) δ 7.29 (s, 5H), 7.19 – 7.07 (m, 4H), 5.69 (q, *J* = 5.2 Hz, 1H), 2.60 (d, *J* = 5.0 Hz, 3H), 2.35 (s, 3H). **^{13}C NMR (101 MHz, CDCl$_3$)** δ 164.9, 151.9 (q, *J* = 3.6 Hz), 139.5, 138.5, 136.4, 129.3, 128.9, 128.6, 128.5, 128.5, 128.3 (q, *J* = 1.9 Hz), 128.2, 126.1 (q, *J* = 30.2 Hz), 122.1 (q, *J* = 275.4 Hz), 26.9, 21.4. **^{19}F NMR (376 MHz, CDCl$_3$)** δ -55.20. **HRMS (m/z, ESI$^+$):** Calculated for $C_{18}H_{16}F_3NONa^+$ [M+Na$^+$] 342.1076, found 342.1079.

(*E*)-3-(3-iodophenyl)-*N*-methyl-3-phenyl-2-(trifluoromethyl)acrylamide (93)

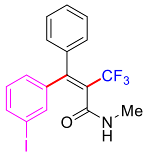

Standard conditions C was followed. White solid. Yield: 37.8 mg, 45% yield.

^{1}H NMR (400 MHz, CDCl$_3$) δ 7.67 (d, *J* = 7.9 Hz, 1H), 7.60 (t, *J* = 1.8 Hz, 1H), 7.42 – 7.33 (m, 4H), 7.21 (m, 2H), 7.08 (t, *J* = 7.8 Hz, 1H), 5.71 (q, *J* = 5.2 Hz, 1H), 2.70 (d, *J* = 4.9 Hz, 3H). **^{19}F NMR (376 MHz, CDCl$_3$)** δ -55.47. **^{13}C NMR (101 MHz, CDCl$_3$)** δ 164.1 (d, *J* = 1.8 Hz), 150.1 (q, *J* = 3.7 Hz), 141.1, 138.2, 137.4, 136.9, 130.1, 128.8, 128.3, 128.1 (q, *J* = 1.9 Hz), 127.5, 121.5 (q, *J* = 37.3 Hz), 121.7 (q, *J* = 275.7 Hz), 94.1, 29.7, 26.8. **HRMS (m/z, ESI$^+$):** Calculated for $C_{17}H_{13}F_3INONa^+$ [M+Na$^+$] 453.9886, found 453.9883.

(*Z*)-*N*-methyl-3-phenyl-3-(p-tolyl)-2-(trifluoromethyl)acrylamide (94)

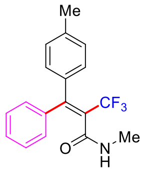

Standard conditions C was followed. White solid. Yield: 35.0 mg, 55% yield.

^{1}H NMR (400 MHz, CDCl$_3$) δ 7.39 – 7.34 (m, 2.7H), 7.31 (d, *J* = 1.5 Hz, 0.6H), 7.24 – 7.16 (m, 3.7H), 7.12 (d, *J* = 8.1 Hz, 2H), 5.71 (q, *J* = 6.5, 5.8 Hz, 1H), 2.67 (d, *J* = 5.0 Hz, 2.5H), 2.62 (d, *J* = 4.8 Hz, 0.5H), 2.37 (s, 0.5H), 2.33 (s, 2.5H). **^{13}C NMR (101 MHz, CDCl$_3$)** δ 164.9, 152.1 (q, *J* = 3.6 Hz), 139.6, 138.7, 135.5, 132.0, 129.6, 129.2, 128.9, 128.7, 128.5, 128.4, 128.3 (q, *J* = 2.0 Hz), 128.1, 126.4 (q, *J* = 30.1 Hz), 122.1 (q, *J* = 275.5 Hz), 29.8, 26.8, 21.4, 19.3. **^{19}F NMR (376 MHz, CDCl$_3$)** δ -55.14. **HRMS (m/z, ESI$^+$):** Calculated for $C_{18}H_{16}F_3NONa^+$ [M+Na$^+$] 342.1076, found 342.1077.

methyl (*Z*)-4-(3,3,3-trifluoro-2-(methylcarbamoyl)-1-phenylprop-1-en-1-yl)benzoate (95)

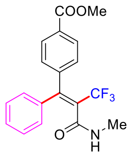

Standard conditions C was followed. White solid. Yield: 29.0 mg, 40% yield.

^{1}H NMR (400 MHz, CDCl$_3$) δ 8.04 (d, *J* = 8.4 Hz, 2H), 7.30 (m, 7H), 5.68 (q, *J* = 5.2 Hz, 1H), 3.92 (s, 3H), 2.64 (d, *J* = 5.0 Hz, 3H). **^{13}C NMR (101 MHz, CDCl$_3$)** δ 166.7, 164.3 (q, *J* = 1.8 Hz), 150.6 (q, *J* = 3.7 Hz), 142.8, 138.4, 130.3, 129.6, 129.5, 128.7, 128.3, 128.3 (q, *J* = 1.4 Hz), 127.4 (q, *J* = 30.5 Hz), 121.9 (q, *J* = 275.6 Hz), 52.4, 26.8. **^{19}F NMR (376 MHz, CDCl$_3$)** δ -55.41. **HRMS (m/z, ESI$^+$):** Calculated for $C_{19}H_{16}F_3NO_3Na^+$ [M+Na$^+$] 386.0974, found 386.0972.

2.4 One-pot two-steps synthesis of *tetra*- and *tri*-substituted olefins

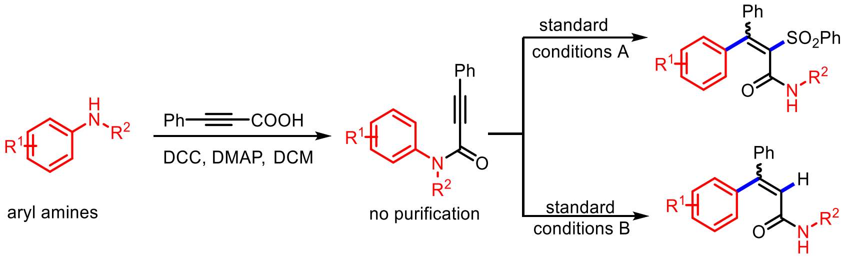

The oven-dried Schlenk tube (38 mL) containing a stirring bar was charged with 3-phenylpropiolic acid (0.2 mmol, 1.0 equiv), aryl amines (0.24 mmol, 1.2 equiv), DCC (0.3 mmol, 1.5 equiv), DMAP (0.02 mmol, 10 mol%) and DCM (4 mL). The tube was then stirred at ambient temperature overnight. Upon completion of the reaction, all the solvents were removed under reduced pressure at high temperature. The crude reaction mixture was diluted with EtOAc (5 mL) and filtered through a short pad of Celite. The sealed tube and Celite pad were washed with an additional 25 mL of EtOAc. The filtrate was concentrated in vacuo. Then the resulting residue was conducted under standard conditions A or B to get the desired products.

2.5 Gram-scale experiments

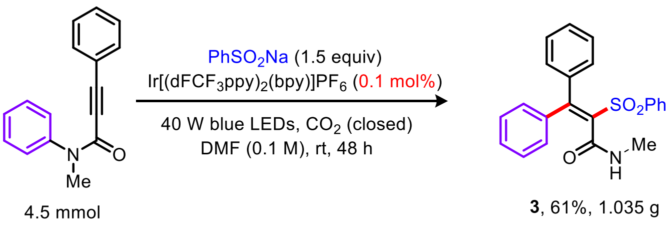

The oven-dried Schlenk tube (250 mL) containing a stirring bar was charged with propiolamide **1a** (4.5 mmol), $PhSO_2Na$ (6.75 mmol, 1.5 equiv), Ir[(dFCF$_3$ppy)$_2$(bpy)]PF$_6$ (0.1 mmol%) and DMF (45 mL). The tube was then evacuated and back-filled with CO_2 for 3 times. The mixture was placed under a 40 W blue LED (λ_{max} = 465 nm, 2.0 cm away from the LEDs, with cooling fan to keep the reaction temperature at 25~30 ^{o}C) light source and stirred at ambient temperature for 48 h. Upon completion of the reaction, all the solvents were removed under reduced pressure at high temperature. The crude reaction mixture was diluted with EtOAc (25 mL) and filtered through a short pad of Celite. The sealed tube and Celite pad were washed with an additional 100 mL of EtOAc.

The filtrate was concentrated in vacuo. The resulting residue was purified by flash silica gel chromatography using petroleum ether/EtOAc (4:1) as the eluent to give the desired product **3** in 61% (1.035 g) yield.

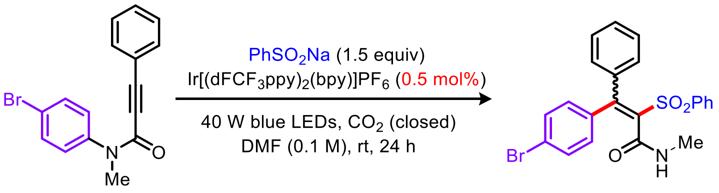

3.5 mmol

32, 79%, 1.261 g

The oven-dried Schlenk tube (250 mL) containing a stirring bar was charged with bromide propiolamide (3.5 mmol), $PhSO_2Na$ (5.25 mmol, 1.5 equiv), Ir[(dFCF$_3$ppy)$_2$(bpy)]PF$_6$ (0.05 mmol%) and anhydrous DMF (35 mL). Tube was then evacuated and back-filled with CO_2 for 3 times. The mixture was placed under a 30 W blue LED (λ_{max} = 465 nm, 2.0 cm away from the LEDs, with cooling fan to keep the reaction temperature at 25~30 ^{o}C) light source and stirred at ambient temperature for 24 h. Upon completion of the reaction, all the solvents were removed under reduced pressure at high temperature. The crude reaction mixture was diluted with EtOAc (25 mL) and filtered through a short pad of Celite. The sealed tube and Celite pad were washed with an additional 100 mL of EtOAc. The filtrate was concentrated in vacuo. The resulting residue was purified by flash silica gel chromatography using petroleum ether/EtOAc (4:1) as the eluent to give the desired product **31** in 79% (1.261 g) yield. The pure *Z*-**32** and *E*-**32** were separated by recrystallization.

2.6 Unsuccessful substrates

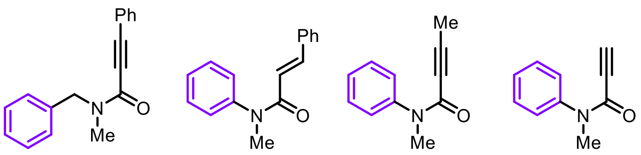

2.7 General photoredox reaction setup

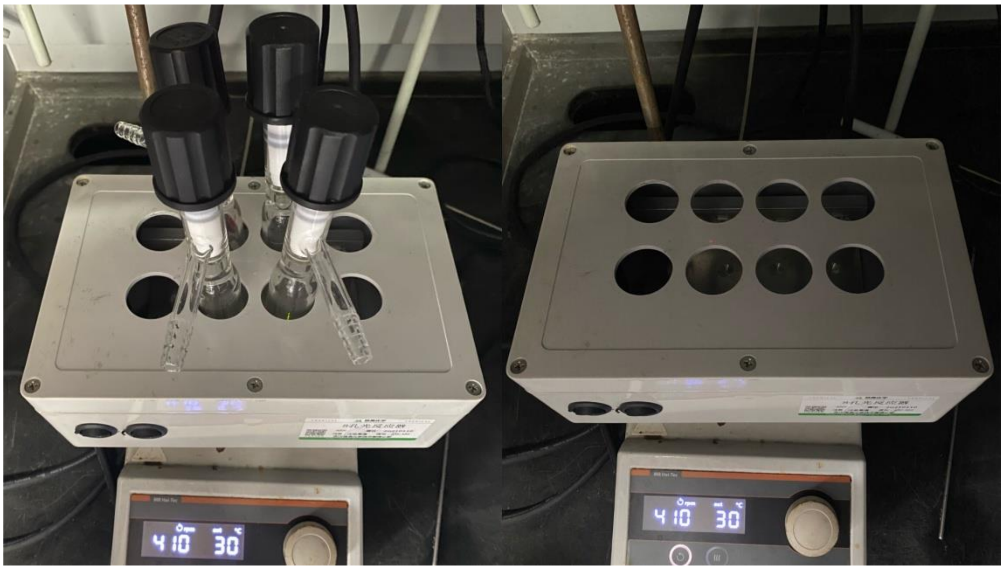

Figure S1. General photoredox reaction setup (before light irradiation).

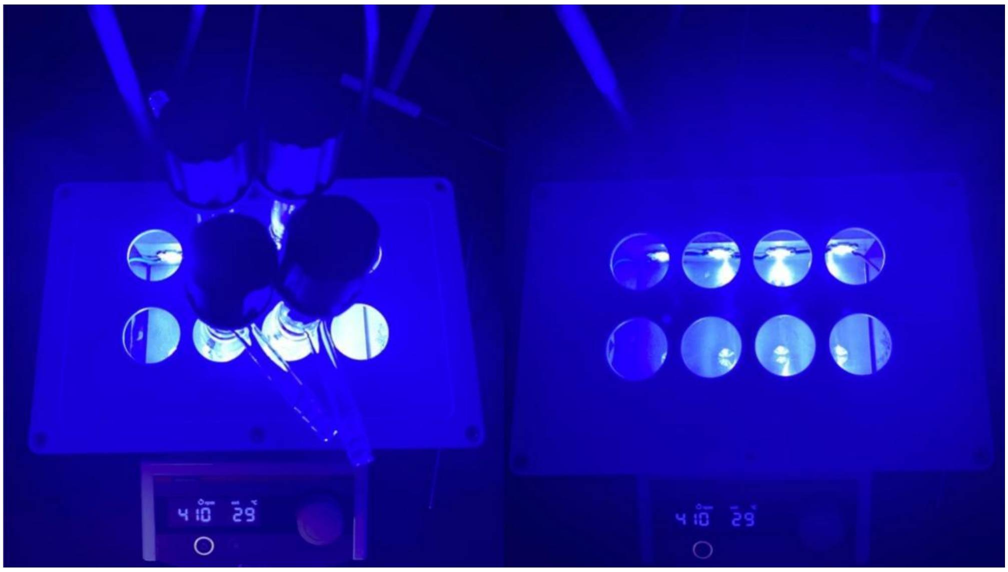

Figure S2. General photoredox reaction setup (during light irradiation).

3. Mechanistic studies

3.1 Stern-Volmer fluorescence quenching experiments

Fluorescence quenching experiments were measured on a Hitachi F7000 Spectrofluorophotometer. DMF was degassed by N_2 bubbling for 30 minutes before using. The complex [Ir(dFCF$_3$ppy)$_2$(bpy)]PF$_6$ was excited at 320 nm and the emission spectrum λmax = 500 nm was recorded. In a typical experiment, 2.0 mL (5.0 × 10^{-5} M) solution of [Ir(dFCF$_3$ppy)$_2$(bpy)]PF$_6$ in DMF was added into the 4.0 mL quartz cuvette (d = 1 cm) and covered with Teflon cap. Then the emission spectrum of the solution was collected at each addition. A stock solution of **1a** (0.25 mmol) or PhSO$_2$Na (0.25 mmol) or **3** (0.25 mmol) in 1 mL of DMF was prepared (0.25 M). Then, different amounts of these stock solutions were added to a solution of the photocatalyst [Ir(dFCF$_3$ppy)$_2$(bpy)]PF$_6$ in DMF (5.0 x 10^{-5} M), respectively.

As shown, a significant decrease of [Ir(dFCF$_3$ppy)$_2$(bpy)]PF$_6$ luminescence was observed, **suggesting that the mechanism might operate via a photo-redox cycle consisting of a reductive quenching with PhSO$_2$Na**.

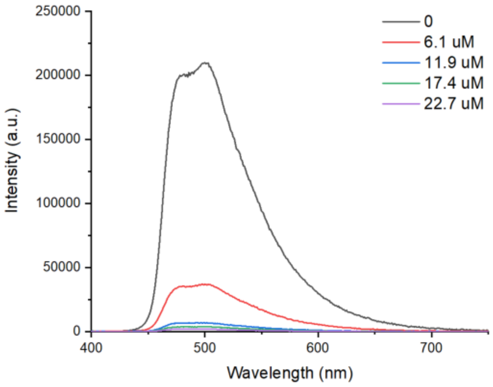

Figure S3. Fluorescence of [Ir(dFCF$_3$ppy)$_2$(bpy)]PF$_6$ with **PhSO$_2$Na** in DMF.

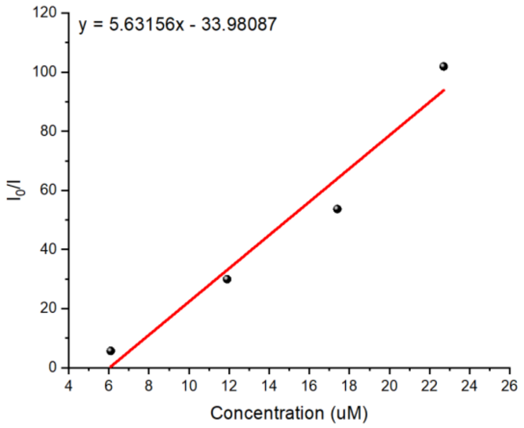

Figure S4. Stern-Volmer quenching plot of **$PhSO_2Na$** .

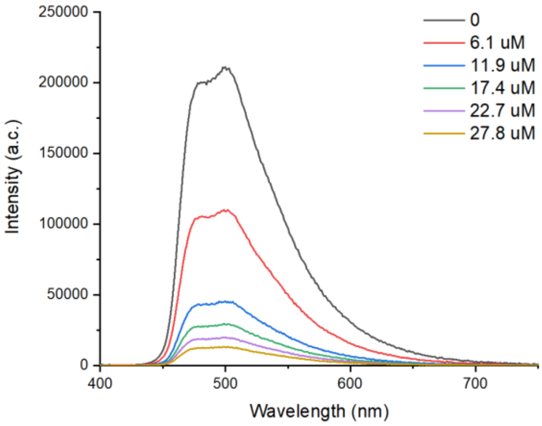

Figure S5. Fluorescence of $[Ir(dFCF_3ppy)_2(bpy)]PF_6$ with **1a** in DMF.

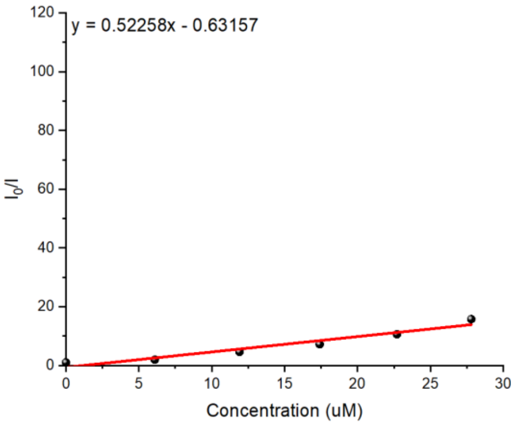

Figure S6. Stern-Volmerquenching plot of **1a**.

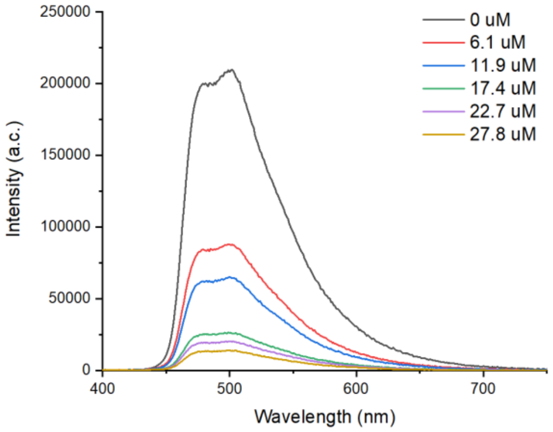

Figure S7. Fluorescence of [Ir(dFCF$_3$ppy)$_2$(bpy)]PF$_6$ with **3** in DMF.

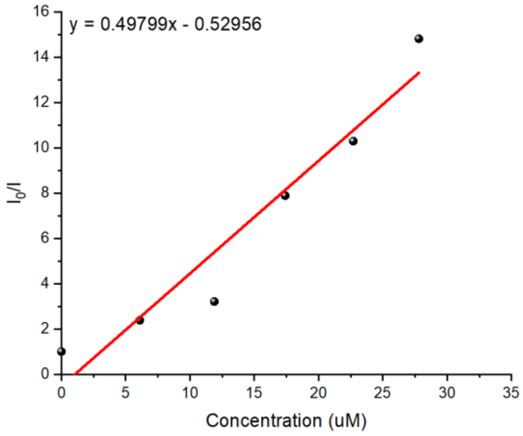

Figure S8. Stern-Volmerquenching plot of **3**.

3.2 Control experiments with radical scavengers

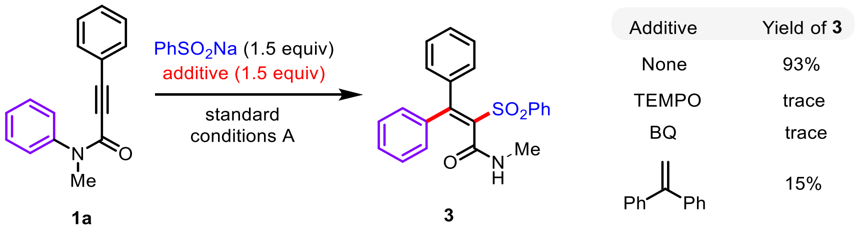

The oven-dried Schlenk tube (38 mL) containing a stirring bar was charged with **1a** (0.1 mmol), PhSO$_2$Na (0.15 mmol, 1.5 equiv), Ir[(dFCF$_3$ppy)$_2$(bpy)]PF$_6$ (1 mmol%), TEMPO (0.15 mmol, 1.5 equiv) or BQ (0.15 mmol, 1.5 equiv) or ethene-1,1-diyldibenzene (0.15 mmol, 1.5 equiv) and DMF (2 mL). The tube was then evacuated and back-filled with CO_2 for 3 times. The mixture was placed under a 40 W blue LED (λ_{max}=465 nm, 1.0 cm away from the LEDs, with cooling fan to keep the reaction temperature at 25~30 ^{o}C) light source and stirred at ambient temperature for 18 h. Upon completion of the reaction, all the solvents were removed under reduced pressure at high temperature. The crude reaction mixture was diluted with EtOAc (5 mL) and filtered through a short pad of

Celite. The sealed tube and Celite pad were washed with an additional 25 mL of EtOAc. The filtrate was concentrated in vacuo, and the yield of product was determined by crude ^{1}H NMR spectrum using $CHCl_2CHCl_2$ as internal standard.

3.3 Reaction with radical clock

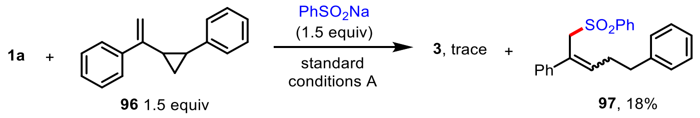

The oven-dried Schlenk tube (38 mL) containing a stirring bar was charged with **1a** (0.1 mmol), $PhSO_2Na$ (0.15 mmol, 1.5 equiv), Ir[(dFCF$_3$ppy)$_2$(bpy)]PF$_6$ (1 mmol%), **96** (0.15 mmol, 1.5 equiv) and DMF (2 mL). The tube was then evacuated and back-filled with CO_2 for 3 times. The mixture was placed under a 30 W blue LED (λ_{max}=465 nm, 1.0 cm away from the LEDs, with cooling fan to keep the reaction temperature at 25~30 ^{o}C) light source and stirred at ambient temperature for 18 h. Upon completion of the reaction, all the solvents were removed under reduced pressure at high temperature. The crude reaction mixture was diluted with EtOAc (5 mL) and filtered through a short pad of Celite. The sealed tube and Celite pad were washed with an additional 25 mL of EtOAc. The filtrate was concentrated in vacuo, and the resulting residue was purified by flash silica gel chromatography using petroleum ether/EtOAc (15:1) as the eluent to give the desired product **97** in 18% yield (9.6 mg).

(5-(phenylsulfonyl)pent-3-ene-1,4-diyl)dibenzene (97)

^{1}H NMR (400 MHz, CDCl$_3$) mixture of *Z*/*E* isomers: δ 7.78 – 7.68 (m, 2H), 7.52 (dt, *J* = 19.3, 7.5 Hz, 1H), 7.39 (dt, *J* = 21.4, 7.7 Hz, 2H), 7.31 – 7.10 (m, 8.5), 7.08 – 7.01 (m, 0.8H), 6.96 – 6.91 (m, 0.7H), 6.01 (t, *J* = 7.4 Hz, 0.6H), 5.71 (t, *J* = 8.0 Hz, 0.4H), 4.24 (s, 1.2H), 4.11 (s, 0.7H), 2.69 (t, *J* = 7.6 Hz, 1.1H), 2.62 – 2.55 (m, 0.9H), 2.42 (q, *J* = 7.5 Hz, 1.2H), 2.31 (q, *J* = 7.6 Hz, 0.8H). **^{13}C NMR (101 MHz, CDCl$_3$) mixture of *Z*/*E* isomers:** δ 141.14, 140.81, 139.03, 138.91, 138.15, 137.66, 137.52, 133.54, 133.45, 128.91, 128.89, 128.82, 128.70, 128.56, 128.54, 128.48, 128.46, 128.44, 128.38, 128.32, 128.18, 127.30, 127.25, 126.42, 126.14, 126.01, 64.98, 57.64, 35.52, 35.19, 31.25, 31.21. **HRMS (ESI+):** Calcd for $C_{23}H_{22}O_2SNa^+$ [M+Na$^+$] 385.1233, found 385.1232.

3.4 Desulfonylated exploration

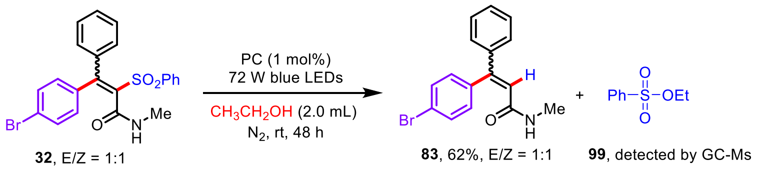

Under a nitrogen atmosphere, the oven-dried Schlenk tube (38 mL) containing a stirring bar was charged with **3** (0.2 mmol), Ir[(dFCF$_3$ppy)$_2$(bpy)]PF$_6$ (1 mmol%) and CH_3CH_2OH (2 mL). Then the mixture was placed under a 72 W blue LED (λ_{max} = 465 nm, 1.0 cm away from the LEDs, with cooling fan to keep the reaction temperature at 25~30 ^{o}C) light source and stirred at ambient temperature for 48 h. Upon completion of the reaction, all the solvents were removed under reduced pressure. The crude reaction mixture was diluted with EtOAc (5 mL) and filtered through a short pad of Celite. The sealed tube and Celite pad were washed with an additional 25 mL of EtOAc. The filtrate was concentrated in vacuo and then analyzed by GC-Ms. The yield of product was determined by crude ^{1}H NMR spectrum using $CHCl_2CHCl_2$ as internal standard.

3.5 *Z*/*E* isomerization of product 32

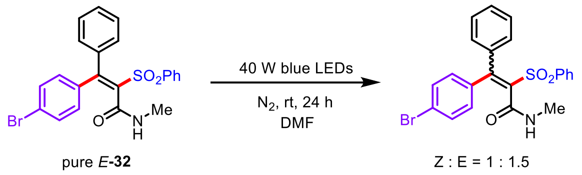

Under a nitrogen atmosphere, the oven-dried Schlenk tube (38 mL) containing a stirring bar was charged with pure ***E*-32** (0.2 mmol) and DMF (2 mL). Then the mixture was placed under a 40 W blue LED (λ_{max} = 465 nm, 1.0 cm away from the LEDs, with cooling fan to keep the reaction temperature at 25~30 ^{o}C) light source and stirred at ambient temperature for 24 h. Upon completion of the reaction, all the solvents were removed under reduced pressure at high temperature. The resulting residue was analyzed by ^{1}H NMR with $CHCl_2CHCl_2$ as internal standard, and the ratio of *E* and *Z* was determined by the crude ^{1}H NMR spectrum.

3.6 Isotope-labelling experiments

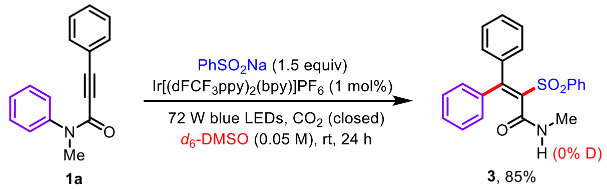

The oven-dried Schlenk tube (38 mL) containing a stirring bar was charged with **1a** (0.1 mmol), $PhSO_2Na$ (0.15 mmol, 1.5 equiv), Ir[(dFCF$_3$ppy)$_2$(bpy)]PF$_6$ (1 mmol%), *d*$_6$-DMSO (2 mL). The tube was then evacuated and back-filled with CO_2 for 3 times. The mixture was placed under a 40 W blue LED (λ_{max}=465 nm, 1.0 cm away from the LEDs, with cooling fan to keep the reaction temperature at 25~30 ^{o}C) light source and stirred at ambient temperature for 24 h. Upon completion of the reaction, the internal standard $CHCl_2CHCl_2$ was added to the crude reaction mixture in the glove box and then was analyzed by ^{1}H NMR directly. (NOTE: The hydrogen atom bonded to nitrogen (O=C−N−H) was high activity, leading a H-D exchange process might occur during the isolation. Therefore, the separation of the product was avoided.)

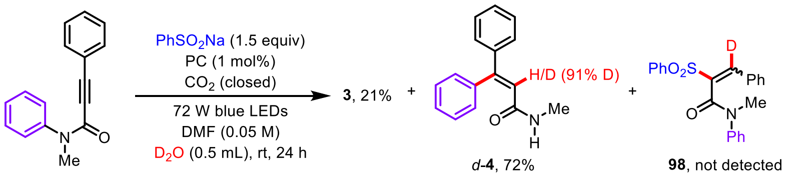

The oven-dried Schlenk tube (38 mL) containing a stirring bar was charged with **1a** (0.1 mmol), $PhSO_2Na$ (0.15 mmol, 1.5 equiv), Ir[(dFCF$_3$ppy)$_2$(bpy)]PF$_6$ (1 mmol%), DMF (2 mL) and D_2O (0.5 mL). The tube was then evacuated and back-filled with CO_2 for 3 times. The mixture was placed under a 72 W blue LED (λ_{max}=465 nm, 1.0 cm away from the LEDs, with cooling fan to keep the reaction temperature at 25~30 ^{o}C) light source and stirred at ambient temperature for 24 h. Upon completion of the reaction, all the solvents were removed under reduced pressure at high temperature. The crude reaction mixture was diluted with EtOAc (5 mL) and filtered through a short pad of Celite. The sealed tube and Celite pad were washed with an additional 25 mL of EtOAc. The filtrate was concentrated in vacuo, then the resulting residue was purified by preparative thin layer chromatography using petroleum ether/EtOAc (3:1) as the eluent to give the desired product ***d*-4** in 72% (16.9 mg) yield.

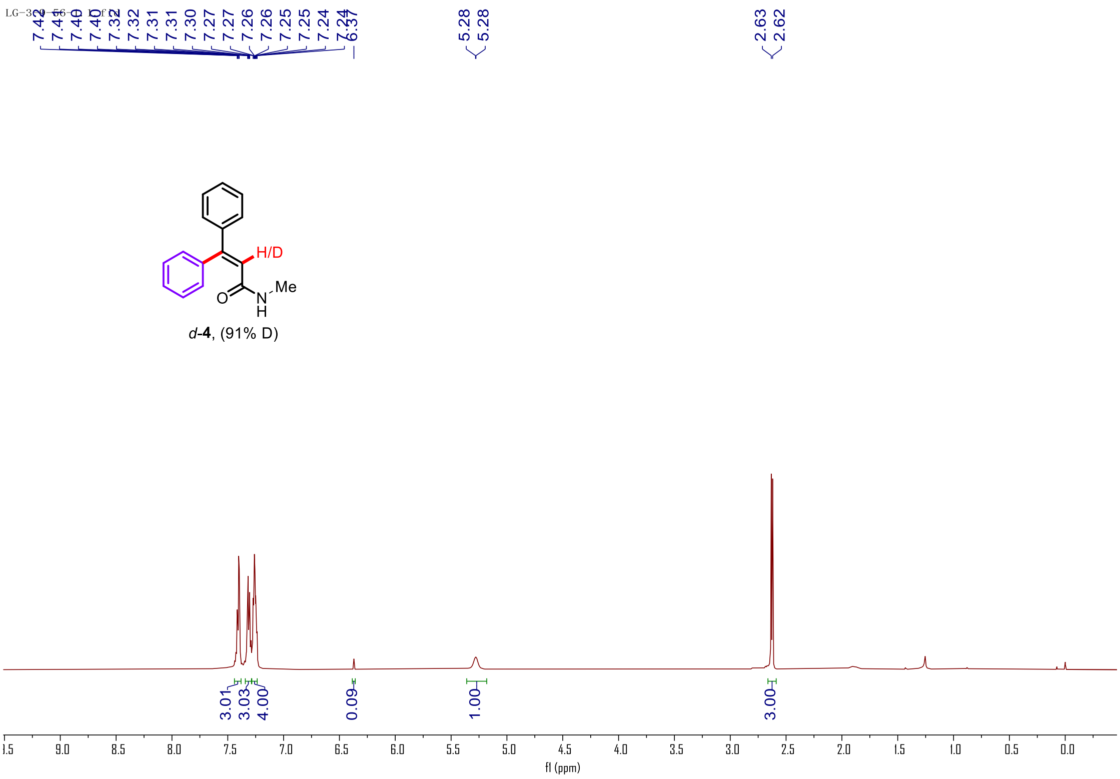

Figure S9. Result of the isotope-labelling experiment by adding D_2O

3.7 Computational details

All DFT calculations were conducted using the Gaussian 16 software package[6] at the unrestricted (U)B3LYP[7-10] level of theory, in combination with the def2-TZVP basis set[11] and the DFT-D3BJ dispersion correction.[12] The PCM solvation model for the Self-Consistent Reaction Field (SCRF) was utilized to account for solvation in DMF, with the solvation parameters (ε=37.2) as specified in Gaussian. Geometry structures were visualized using CYLview.[13]

Reduction potentials were determined using a modified procedure as described by Nicewicz and coworkers.[14] Geometry optimizations were conducted for both the reduced and neutral forms of each molecule, followed by frequency calculations were performed on the minimized structures to confirm no imaginary frequencies existed. Gibbs free energies (G_{298}) were derived from the DFT calculation and applied in the following equation:

$$E_{1/2}^{0,calc} = -\frac{(G_{298}[reduced] - G_{298}[oxidized])}{n_e \mathrm{F}} - E_{1/2}^{0,SHE} + E_{1/2}^{0,SCE}$$

Where n_e represents the number of electrons transferred ($n_e = 1$ for all calculations here), $\mathscr{F}$ is the Faraday constant valued at 23.061 kcal mol^{-1} V^{-1}, $E_{1/2}^{0,SCE}$ is the absolute value for the standard hydrogen electrode (SHE) at 4.281 V, and $E_{1/2}^{0,SCE}$ is the potential of the saturated calomel electrode (SCE) relative to the SHE in DMF, which is –0.099V.[15] $G_{298[oxidized]}$ and $G_{298[reduced]}$ are the Gibbs free energies in DMF obtained from DFT calculations.

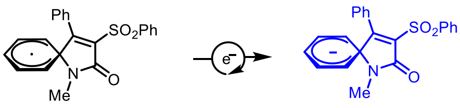

$E_{1/2}{}^{o}$ = -1.17 V vs SCE

Molecular coordinates of optimized structures:

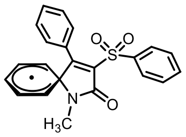

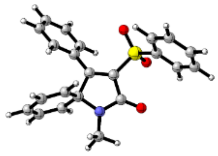

Charge: 0

Multiplicity: 2

Number of Imaginary Frequencies: 0

Solvation: DMF

G298: -1528.008385 Hartree

C	-3.13922200	-2.14477400	1.69405500
C	-1.89768100	-1.73232600	1.32799700
C	-1.56186600	-1.34549400	-0.08891700
C	-2.75193600	-1.41190900	-1.00444900
C	-3.97565500	-1.81313900	-0.57660100
C	-4.20192000	-2.18662800	0.76683600
H	-3.31822300	-2.44580300	2.71906700
H	-1.07733000	-1.70532500	2.03364800
H	-2.57740600	-1.13110500	-2.03486400
H	-4.79745100	-1.85183800	-1.28070900
H	-5.18349700	-2.51162600	1.08124300
C	-0.90887400	0.05399200	-0.15199000
C	-1.70943500	1.26452400	0.04171300
C	-1.63907600	2.29032900	-0.90740600
C	-2.59178200	1.40066100	1.11735900
C	-2.42383400	3.42444700	-0.77747600
H	-0.97289600	2.19018800	-1.75239200
C	-3.36538800	2.54390900	1.25161600
H	-2.65947700	0.61836200	1.85801100
C	-3.28601000	3.55782100	0.30458800
H	-2.35732500	4.20768900	-1.52090300
H	-4.03210000	2.64222200	2.09840300
H	-3.89400600	4.44715800	0.40851500
C	0.37339700	-0.08719500	-0.52282900
C	0.68287300	-1.51813100	-0.83129000
N	-0.47371600	-2.20159400	-0.57976400
C	-0.60185800	-3.63345800	-0.70964700
H	-0.82228900	-4.09697100	0.25455700
H	-1.39920200	-3.89020200	-1.40957100
H	0.34521800	-4.01367200	-1.08508700
O	1.72547500	-1.99575400	-1.23016000
S	1.60354300	1.18111000	-0.69481000
O	1.16532900	2.33284900	0.05448500
O	1.90715900	1.29885900	-2.09969900

C	3.00759400	0.50090900	0.14746700
C	3.99405900	-0.15486200	-0.57709300
C	3.09837000	0.66023500	1.52644200
C	5.09701300	-0.65824300	0.09816000
H	3.88397200	-0.27031300	-1.64413800
C	4.20152200	0.14490500	2.19175900
H	2.32405000	1.19653000	2.05683100
C	5.19819000	-0.51276900	1.47739100
H	5.87482000	-1.16998200	-0.45271100
H	4.28850900	0.26437300	3.26362700
H	6.05938200	-0.91075900	1.99865900

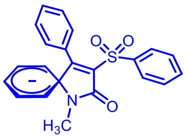

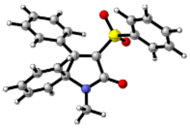

Charge: -1

Multiplicity: 1

Number of Imaginary Frequencies: 0

Solvation: DMF

G298: -1528.126328 Hartree

C	-2.74053700	-2.32051500	1.83363600
C	-1.61256200	-1.77482300	1.24261400
C	-1.56732500	-1.35003800	-0.20540500
C	-2.90953100	-1.53596000	-0.85454200
C	-3.98167200	-2.11293800	-0.21220900
C	-3.95060600	-2.51567900	1.14519600
H	-2.66949700	-2.61033700	2.89018900
H	-0.69431800	-1.63056400	1.81926400
H	-2.99657500	-1.22071500	-1.89912200
H	-4.90999100	-2.23937900	-0.78412800
H	-4.82417700	-2.95529400	1.63115700
C	-0.98525200	0.10163900	-0.23626300
C	-1.83772400	1.26822500	-0.00507800
C	-1.59057000	2.47380400	-0.70107800
C	-2.99910200	1.19060200	0.79517700
C	-2.45557600	3.55977200	-0.58563100
H	-0.72271100	2.54792400	-1.35673000
C	-3.85869600	2.28543700	0.90982800
H	-3.21065400	0.27339700	1.34392700
C	-3.59358400	3.47492300	0.22582300
H	-2.23988300	4.47921100	-1.13737100
H	-4.74719200	2.20270700	1.54258800

H	-4.27002500	4.32992900	0.31919600
C	0.33441300	0.02379100	-0.57551600
C	0.66120300	-1.33235100	-1.11433800
N	-0.46483600	-2.07516100	-0.94912000
C	-0.49803700	-3.49869000	-1.14354400
H	-0.67627500	-4.01343300	-0.18078000
H	-1.30524000	-3.79271000	-1.83525100
H	0.47168900	-3.81038600	-1.55715200
O	1.72774500	-1.70676600	-1.59841800
S	1.58435300	1.28322300	-0.47836900
O	1.16824100	2.29740400	0.51005700
O	1.95167600	1.71352800	-1.84197200
C	3.04245700	0.48279600	0.23743800
C	3.99720100	-0.10738300	-0.59443700
C	3.21439500	0.53769000	1.62330500
C	5.14413000	-0.65789000	-0.01846400
H	3.82086400	-0.13887200	-1.66871500
C	4.36236100	-0.02440600	2.18914900
H	2.45625400	1.03022300	2.23473600
C	5.32629400	-0.62076500	1.36928700
H	5.89869600	-1.12528500	-0.65706700
H	4.50688500	0.00990200	3.27256400
H	6.22497600	-1.05852800	1.81341300

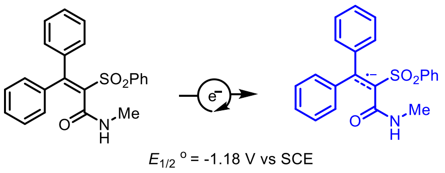

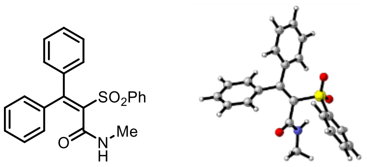

Charge: 0

Multiplicity: 1

Number of Imaginary Frequencies: 0

Solvation: DMF

G_{298}: -1528.648957 Hartree

C	-2.73806900	-3.30372200	1.32113900
C	-1.84837200	-2.24080800	1.14249300
C	-2.12619200	-1.23210100	0.20238700
C	-3.31847400	-1.31385600	-0.54499300
C	-4.19148300	-2.38888300	-0.37990400

C	-3.90565800	-3.38632700	0.55870400
H	-2.51092300	-4.07480400	2.06164100
H	-0.92897500	-2.19326900	1.72368100
H	-3.55535100	-0.52870500	-1.26632600
H	-5.10311300	-2.44385100	-0.98032600
H	-4.59509500	-4.22318100	0.69761900
C	-1.22933800	-0.04746100	0.01385200
C	-1.93907500	1.26764200	0.04389300
C	-1.83652400	2.17798300	-1.02227300
C	-2.79493700	1.57428300	1.11730400
C	-2.56250600	3.37033800	-1.00728300
H	-1.18131800	1.95327100	-1.86603100
C	-3.50060400	2.77727100	1.14119300
H	-2.89670200	0.86657900	1.94313900
C	-3.39007300	3.67716200	0.07590300
H	-2.47449500	4.06562900	-1.84573900
H	-4.14595800	3.01051800	1.99186500
H	-3.95217600	4.61449800	0.08911200
C	0.09970100	-0.18753900	-0.24005100
C	0.80230400	-1.50493900	-0.52700700
N	1.27234100	-1.58682400	-1.80224100
C	2.04390800	-2.71479900	-2.28040700
H	1.60174900	-3.13508100	-3.19842600
H	3.08893100	-2.43035500	-2.49360600
H	2.04105400	-3.48442100	-1.49802700
O	0.94117600	-2.38372500	0.30923600
S	1.21792500	1.23755800	-0.40356600
O	0.76848800	2.35941600	0.42760200
O	1.45444100	1.42926600	-1.85535000
C	2.75962300	0.64368200	0.32039900
C	3.86790500	0.44588800	-0.50474400
C	2.83247500	0.46878600	1.70567700
C	5.07630700	0.04579300	0.07345800
H	3.77576500	0.61763200	-1.57841100
C	4.04193900	0.06028300	2.26960600
H	1.95658600	0.65570300	2.32985400
C	5.16080200	-0.15113600	1.45513600
H	5.95471800	-0.10898700	-0.55783800
H	4.11377400	-0.08820900	3.34961900
H	6.10623700	-0.46770100	1.90272200
H	1.17730200	-0.76331100	-2.39059400

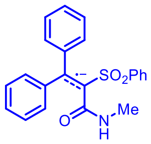

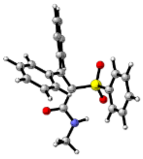

Charge: -1

Multiplicity: 2

Number of Imaginary Frequencies: 0

Solvation: DMF

G_{298}: -1528.766635 Hartree

C	-0.03088200	3.73867600	-0.67474800
C	0.01221300	2.38511100	-0.35520800
C	-1.16528300	1.64945600	-0.06315600
C	-2.38580500	2.37275500	-0.08792500
C	-2.42643300	3.73039200	-0.40182700
C	-1.25095700	4.42876200	-0.70467100
H	0.90028400	4.26572300	-0.90590900
H	0.96922200	1.86059000	-0.34177300
H	-3.31189100	1.85754100	0.17380400
H	-3.38790400	4.25431200	-0.39807800
H	-1.28334200	5.49387600	-0.95236500
C	-1.09813200	0.21964500	0.25851100
C	-2.22833900	-0.65451200	-0.06610600
C	-2.54292000	-1.76861200	0.75466100
C	-3.06198600	-0.44286700	-1.19497300
C	-3.62661300	-2.59624800	0.47939600
H	-1.91225300	-1.95976300	1.62474500
C	-4.14264300	-1.27839900	-1.47376600
H	-2.83394400	0.38097800	-1.87436400
C	-4.44139000	-2.36107500	-0.63738100
H	-3.84214600	-3.43886500	1.14406300
H	-4.75417700	-1.08877200	-2.36200700
H	-5.29048100	-3.01556100	-0.85546700
C	0.06554200	-0.30414600	0.95387400
C	0.62078500	0.41295800	2.11953600
N	1.77680100	-0.08310000	2.71479600
C	2.19135900	0.44639400	3.98970800
H	1.57929200	0.08295600	4.84199400
H	3.24172400	0.17274200	4.18454400
H	2.10030800	1.54185700	3.98010400
O	0.08237500	1.42178600	2.58157900
S	0.90923600	-1.64855400	0.22402500
O	0.02462200	-2.44688300	-0.64931400
O	1.72857800	-2.38260800	1.23313800
C	2.14408000	-0.94421800	-0.92776100

C	3.33762900	-0.39563600	-0.44047200
C	1.86967900	-0.94478300	-2.29928400
C	4.25345000	0.16214300	-1.33533400
H	3.52971400	-0.39672100	0.63359000
C	2.79257700	-0.38418800	-3.18798100
H	0.93807400	-1.39559300	-2.64608700
C	3.98482800	0.16952600	-2.71031700
H	5.18568200	0.59265300	-0.95778700
H	2.57999700	-0.38378300	-4.26121200
H	4.70545700	0.60676800	-3.40743000
H	1.99185000	-1.05755100	2.49976700

4. NMR Spectra of Compounds

^{1}H NMR Spectrum of *N*-methyl-3,3-diphenyl-2-(phenylsulfonyl)acrylamide (3, $CDCl_3$ as solvent, 400 MHz)

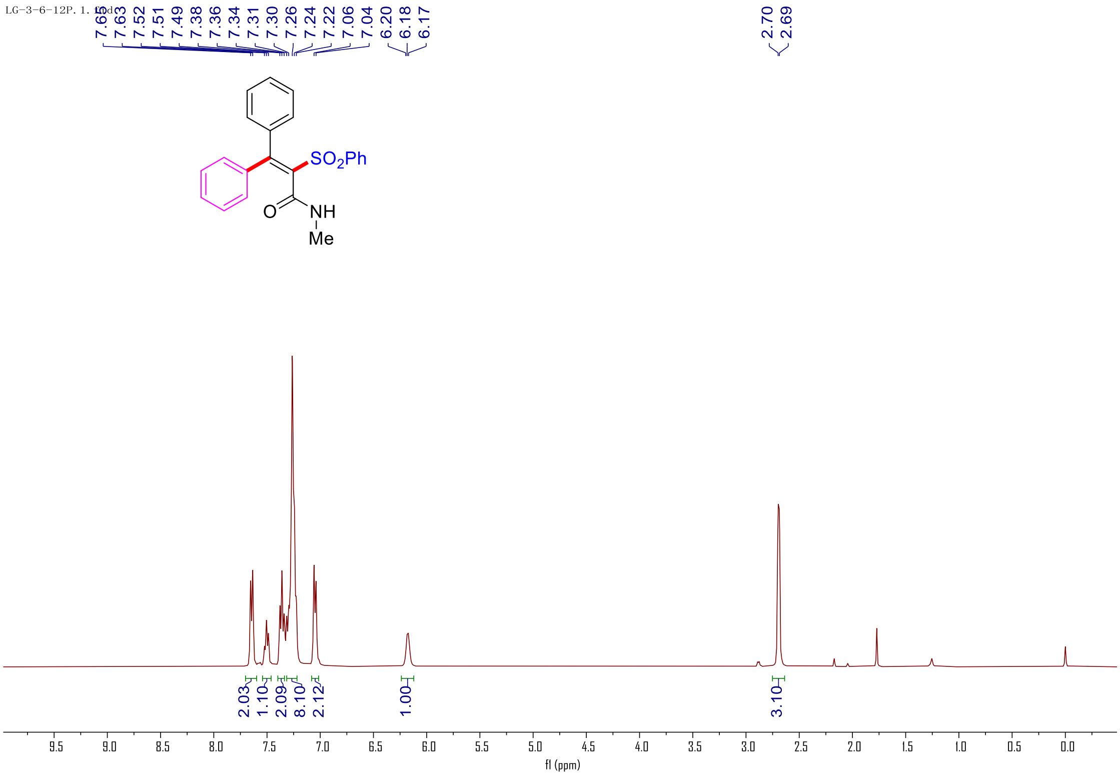

^{13}C {^{1}H} NMR Spectrum of *N*-methyl-3,3-diphenyl-2-(phenylsulfonyl)acrylamide (3, $CDCl_3$ as solvent, 101 MHz)

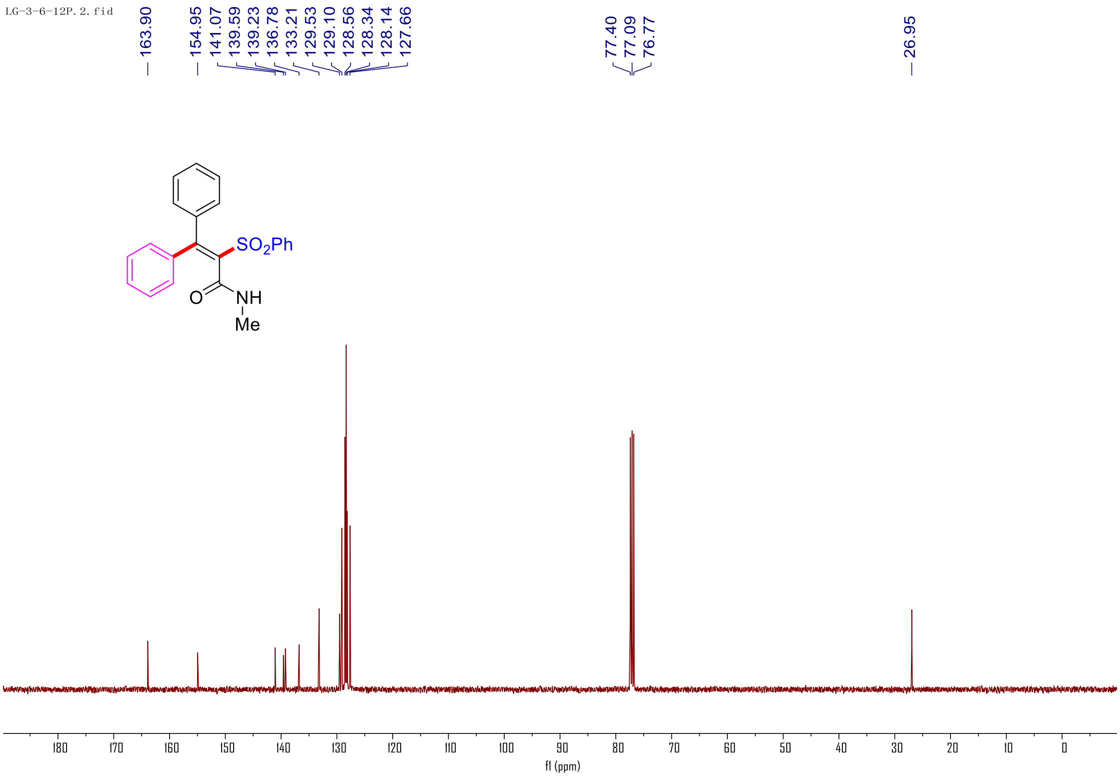

^{1}H NMR Spectrum of *N*-methyl-3,3-diphenylacrylamide (4, $CDCl_3$ as solvent, 400 MHz)

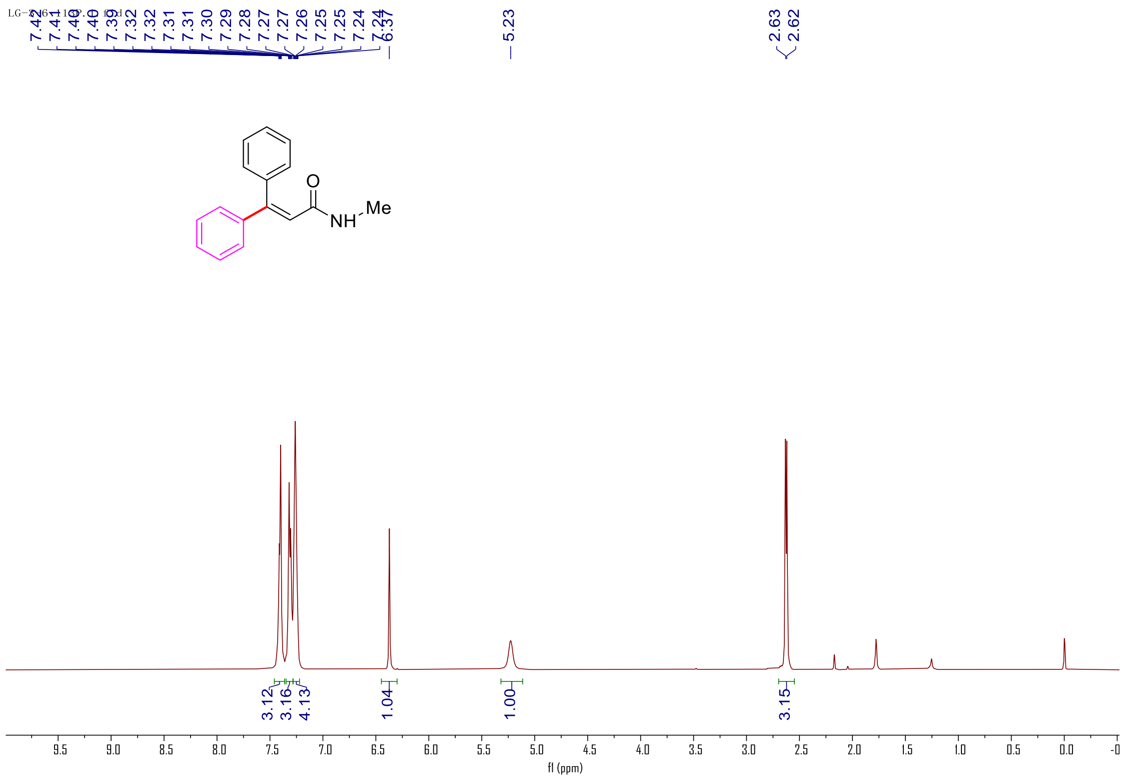

^{13}C {^{1}H} NMR Spectrum of *N*-methyl-3,3-diphenylacrylamide (4, $CDCl_3$ as solvent, 101 MHz)

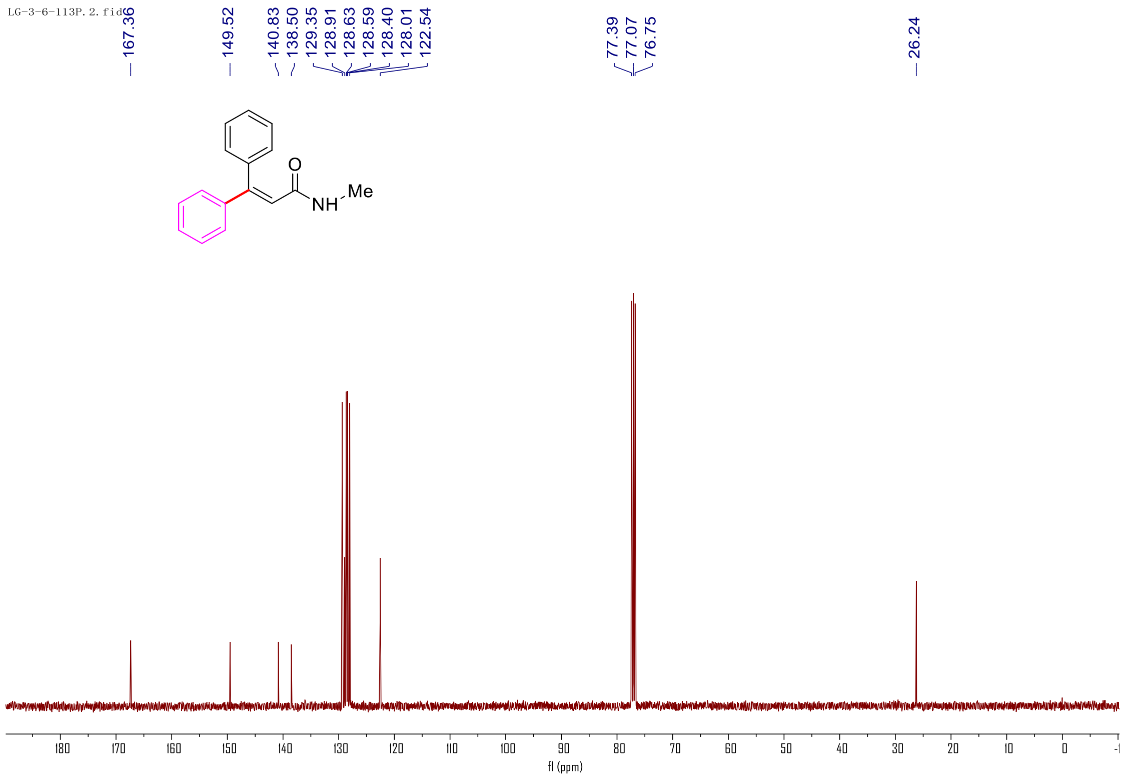

^{1}H NMR Spectrum of *N*-ethyl-3,3-diphenyl-2-(phenylsulfonyl)acrylamide (5, $CDCl_3$ as solvent, 400 MHz)

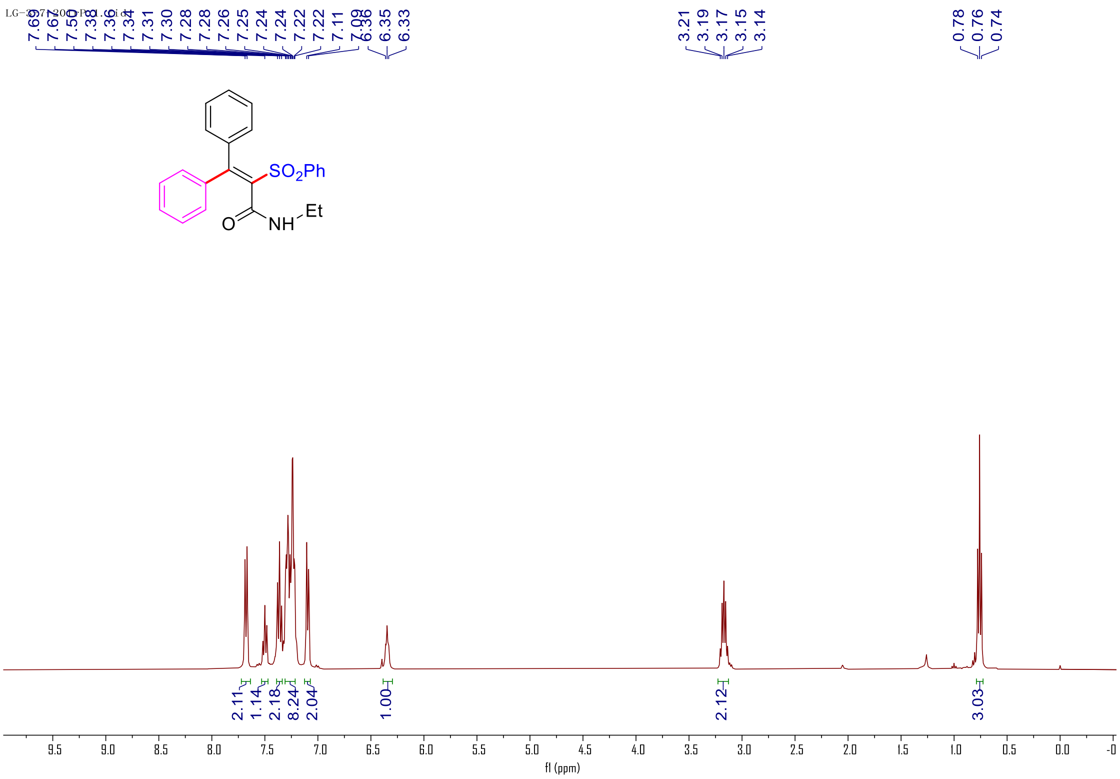

^{13}C {^{1}H} NMR Spectrum of *N*-ethyl-3,3-diphenyl-2-(phenylsulfonyl)acrylamide (5, $CDCl_3$ as solvent, 101 MHz)

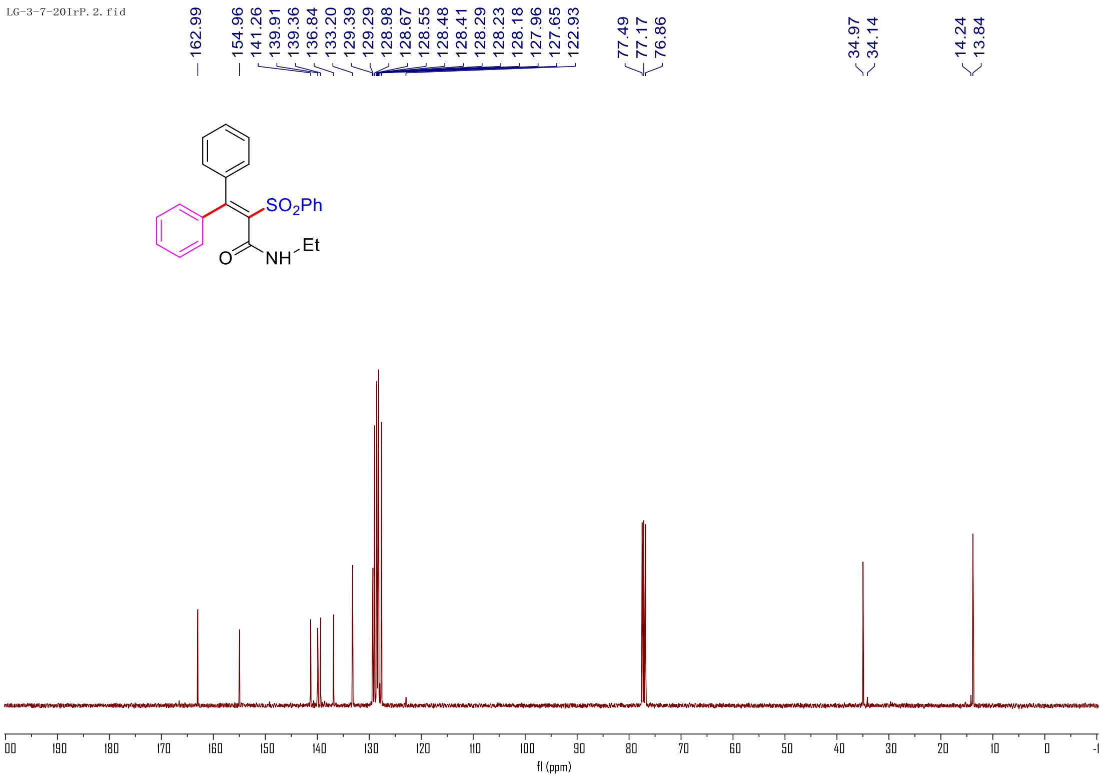

^{1}H NMR Spectrum of *N*-allyl-3,3-diphenyl-2-(phenylsulfonyl)acrylamide (6, $CDCl_3$ as solvent, 400 MHz)

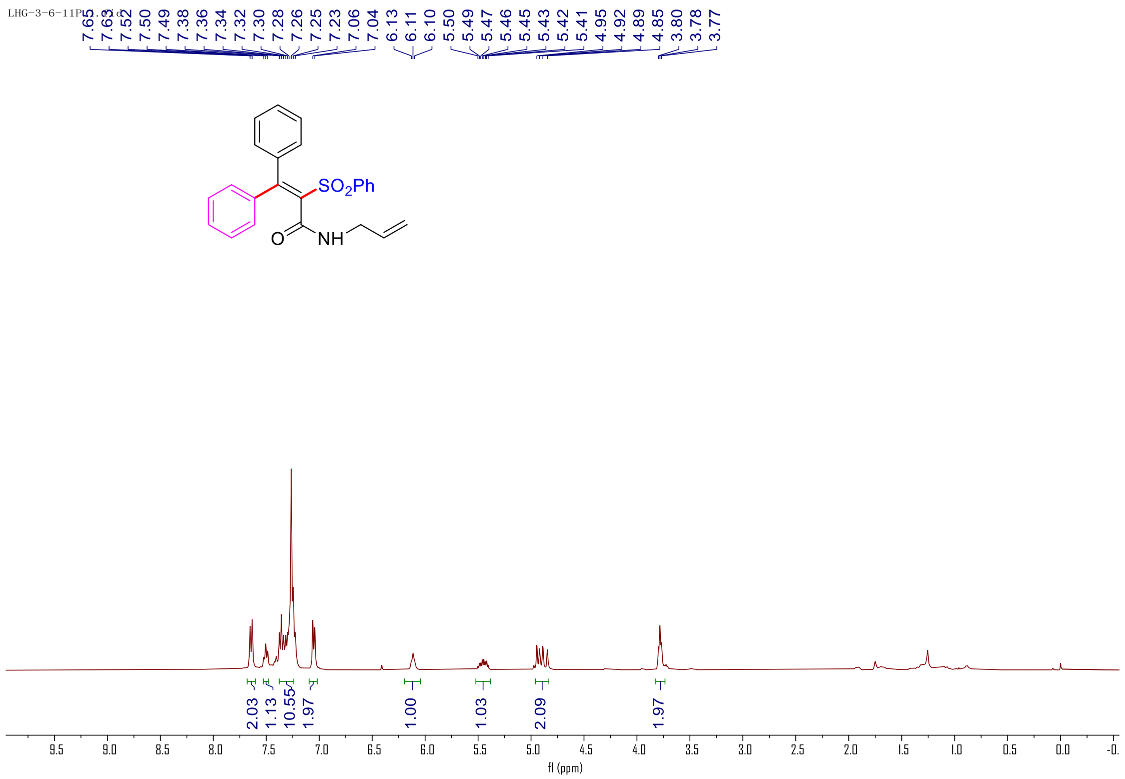

^{13}C {^{1}H} NMR Spectrum of *N*-allyl-3,3-diphenyl-2-(phenylsulfonyl)acrylamide (6, $CDCl_3$ as solvent, 101 MHz)

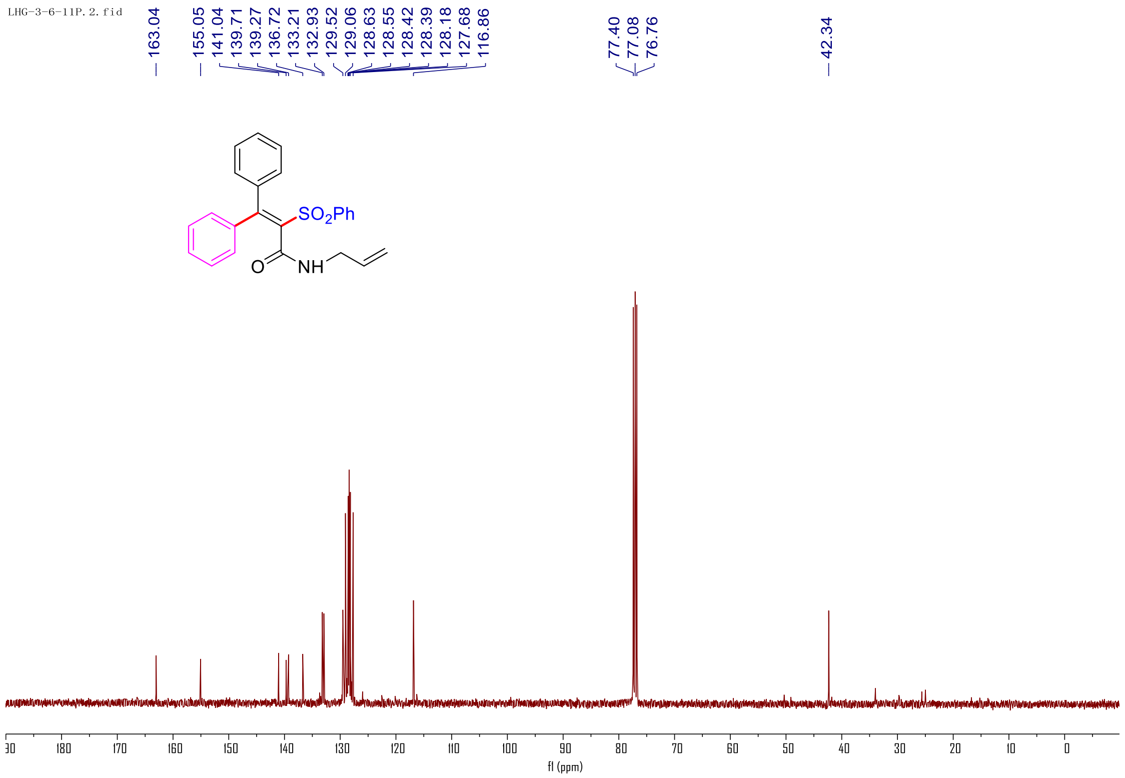

^{1}H NMR Spectrum of *N*-benzyl-3,3-diphenyl-2-(phenylsulfonyl)acrylamide (7, $CDCl_3$ as solvent, 400 MHz)

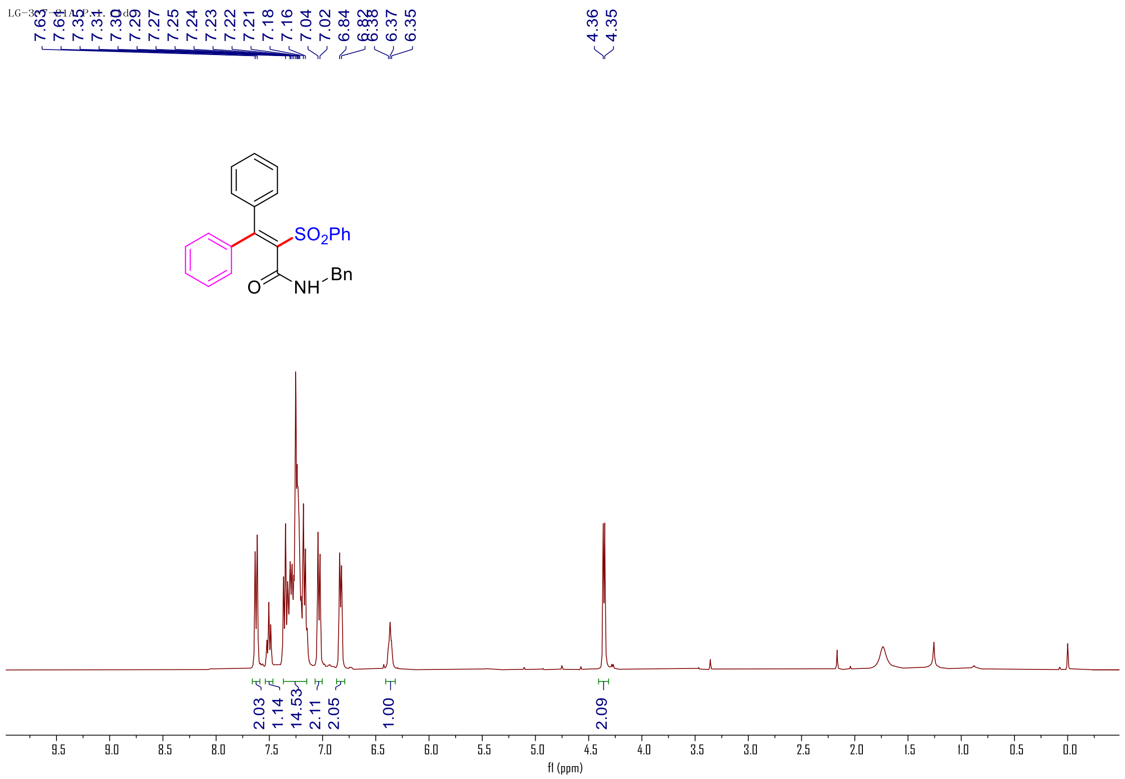

^{13}C {^{1}H} NMR Spectrum of *N*-benzyl-3,3-diphenyl-2-(phenylsulfonyl)acrylamide (7, $CDCl_3$ as solvent, 101 MHz)

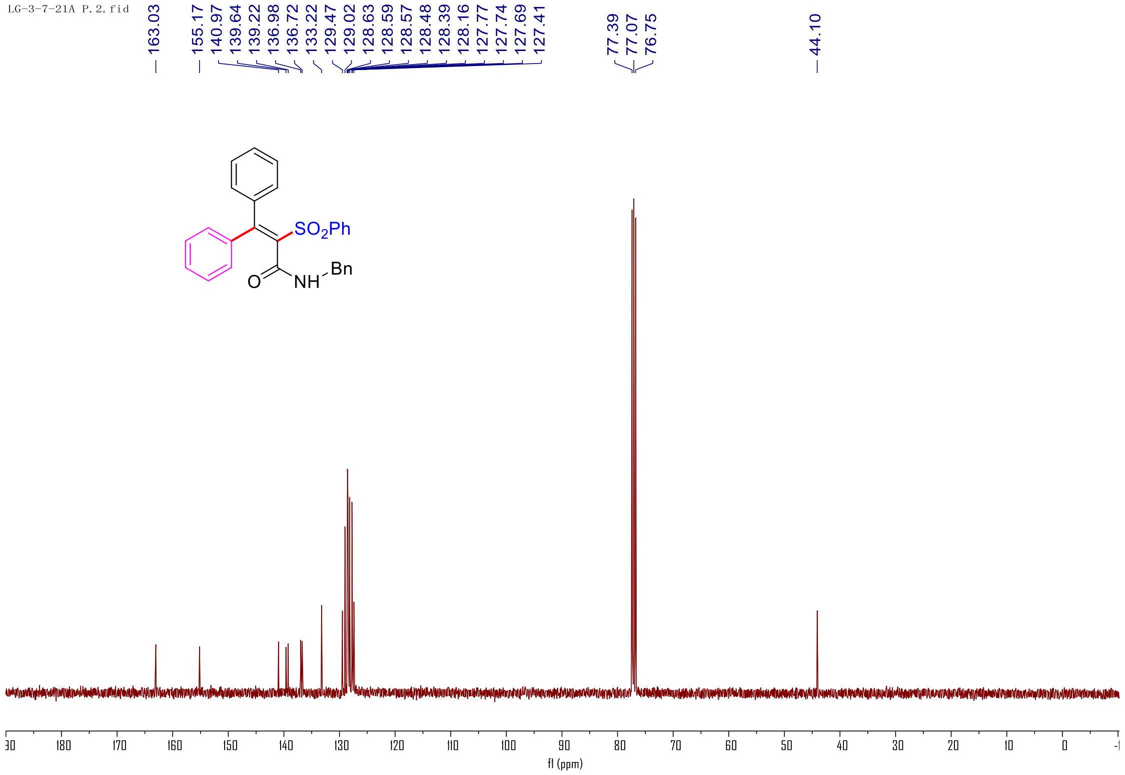

^{1}H NMR Spectrum of 3,3-diphenyl-*N*-(1-phenylethyl)-2-(phenylsulfonyl)acrylamide (8, $CDCl_3$ as solvent, 400 MHz)

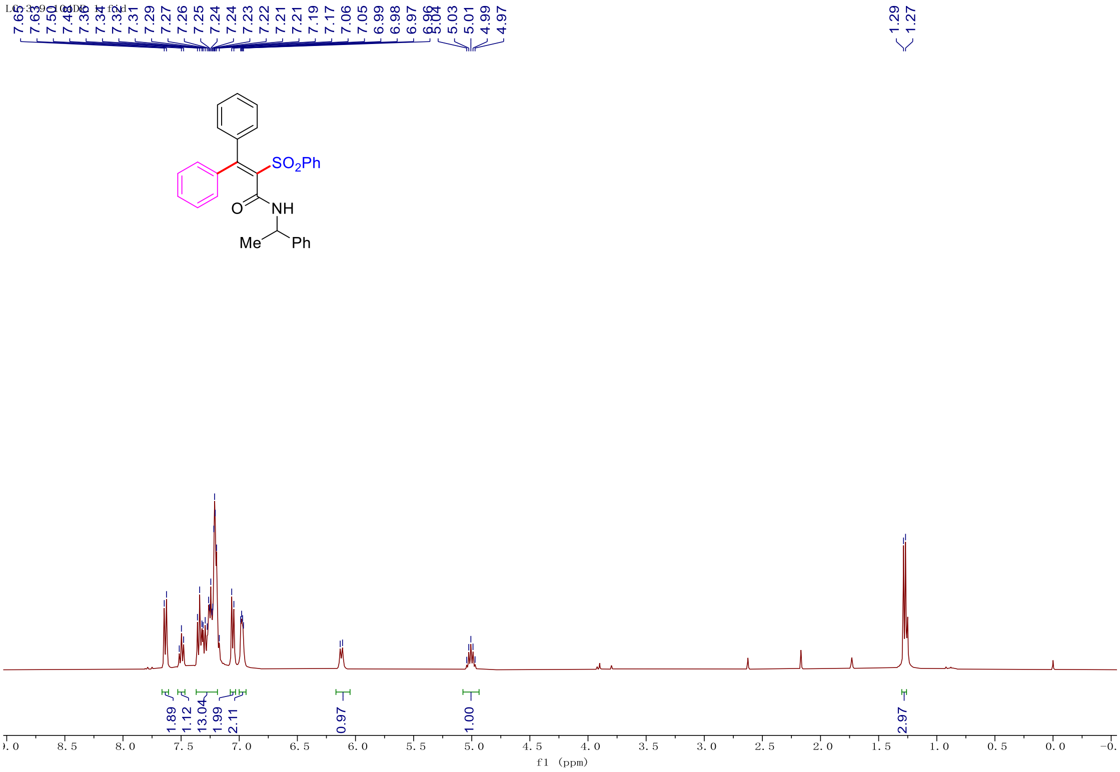

^{13}C {^{1}H} NMR Spectrum of 3,3-diphenyl-*N*-(1-phenylethyl)-2-(phenylsulfonyl)acrylamide (8, $CDCl_3$ as solvent, 101 MHz)

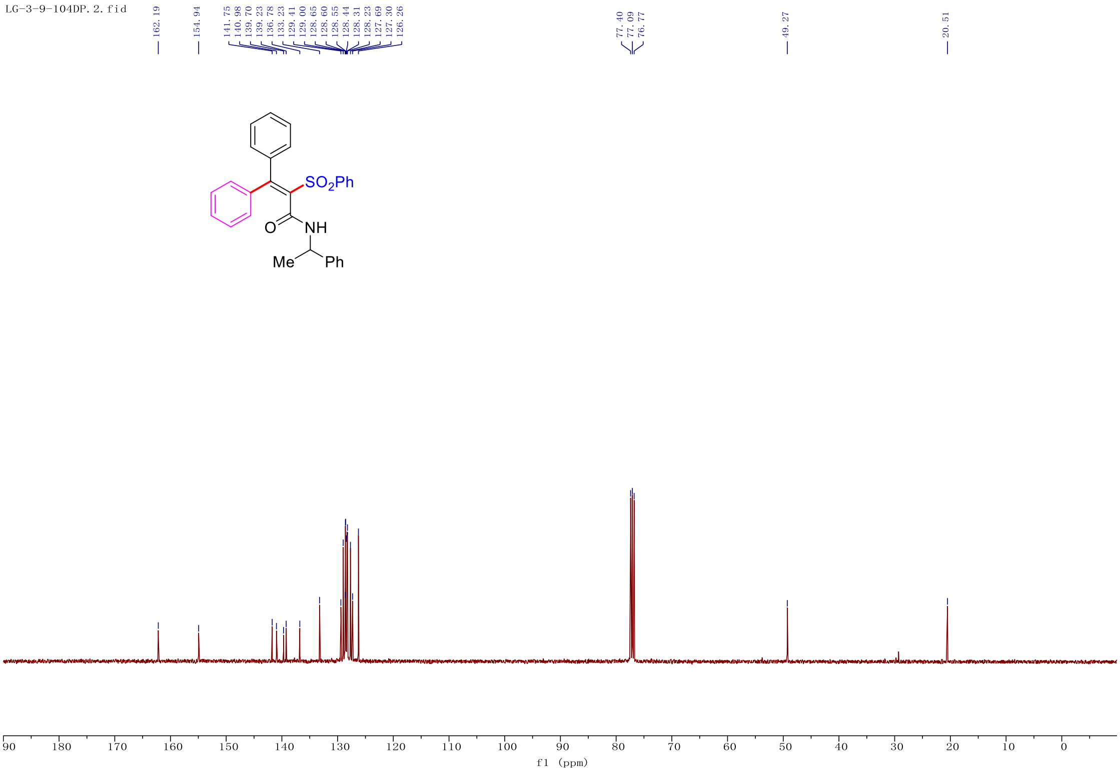

^{1}H NMR Spectrum of 3,3-diphenyl-2-(phenylsulfonyl)acrylamide (9, $CDCl_3$ as solvent, 400 MHz)

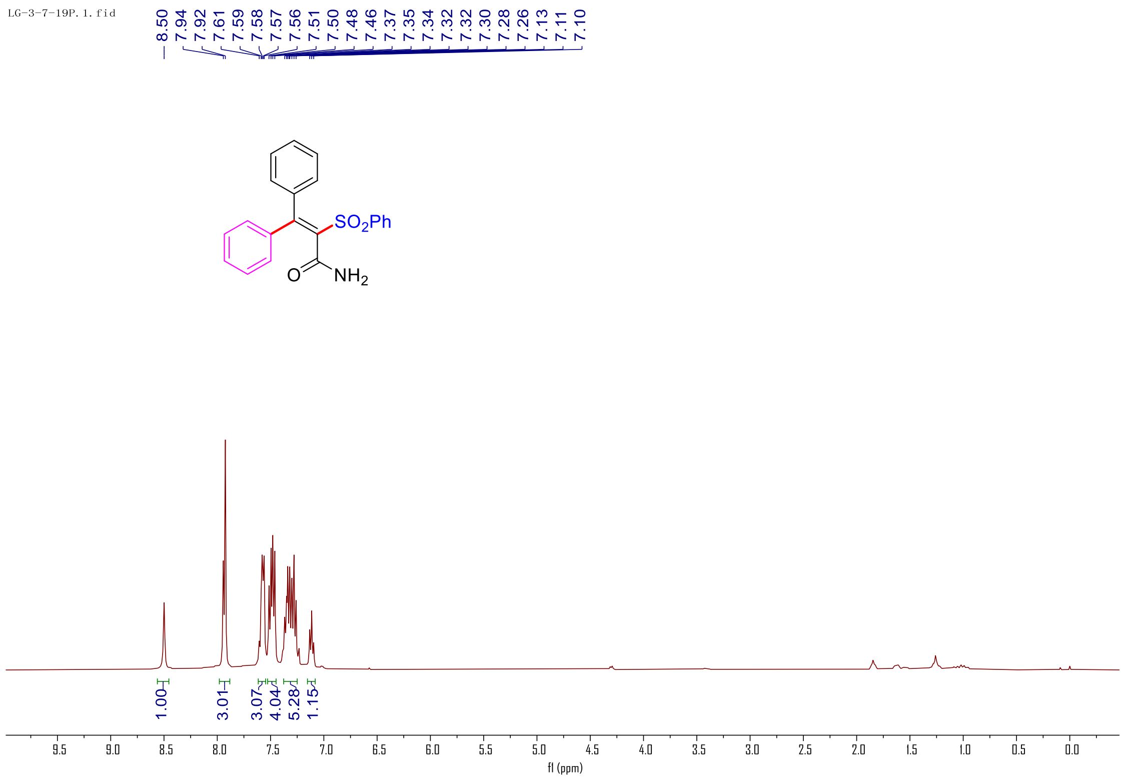

^{13}C {^{1}H} NMR Spectrum of 3,3-diphenyl-2-(phenylsulfonyl)acrylamide (9, $CDCl_3$ as solvent, 101 MHz)

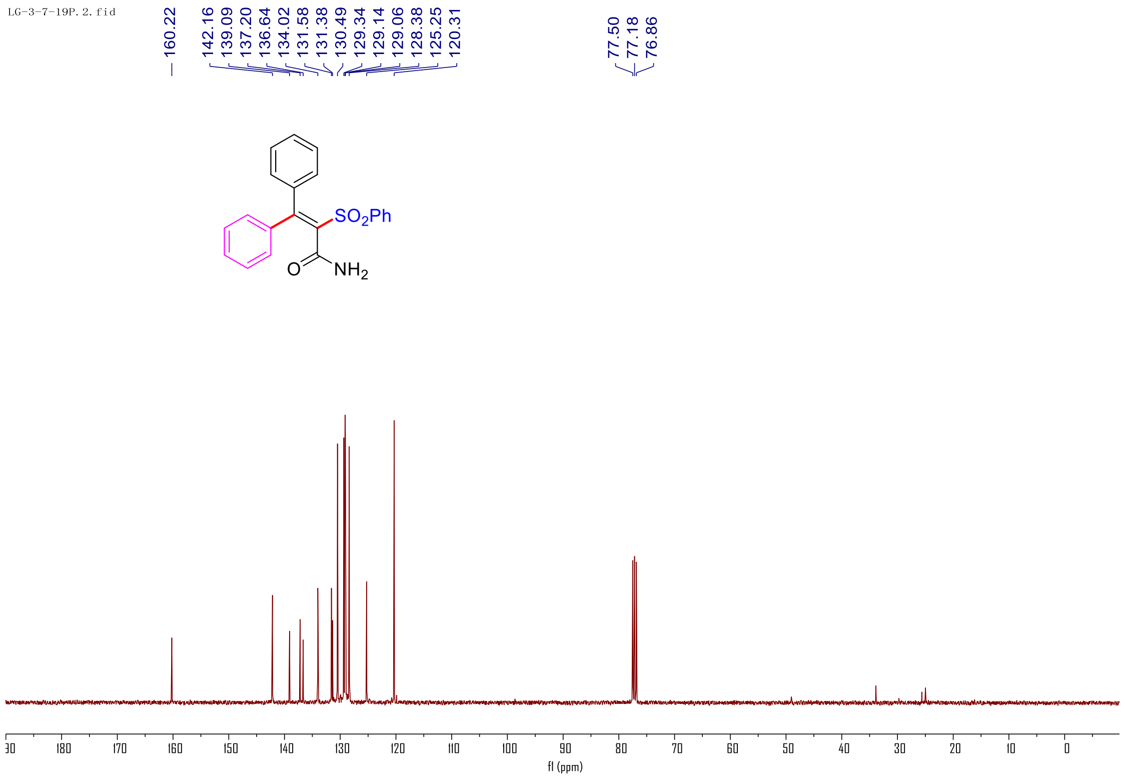

^{1}H NMR Spectrum of *N*,3,3-triphenyl-2-(phenylsulfonyl)acrylamide (10, $CDCl_3$ as solvent, 400 MHz)

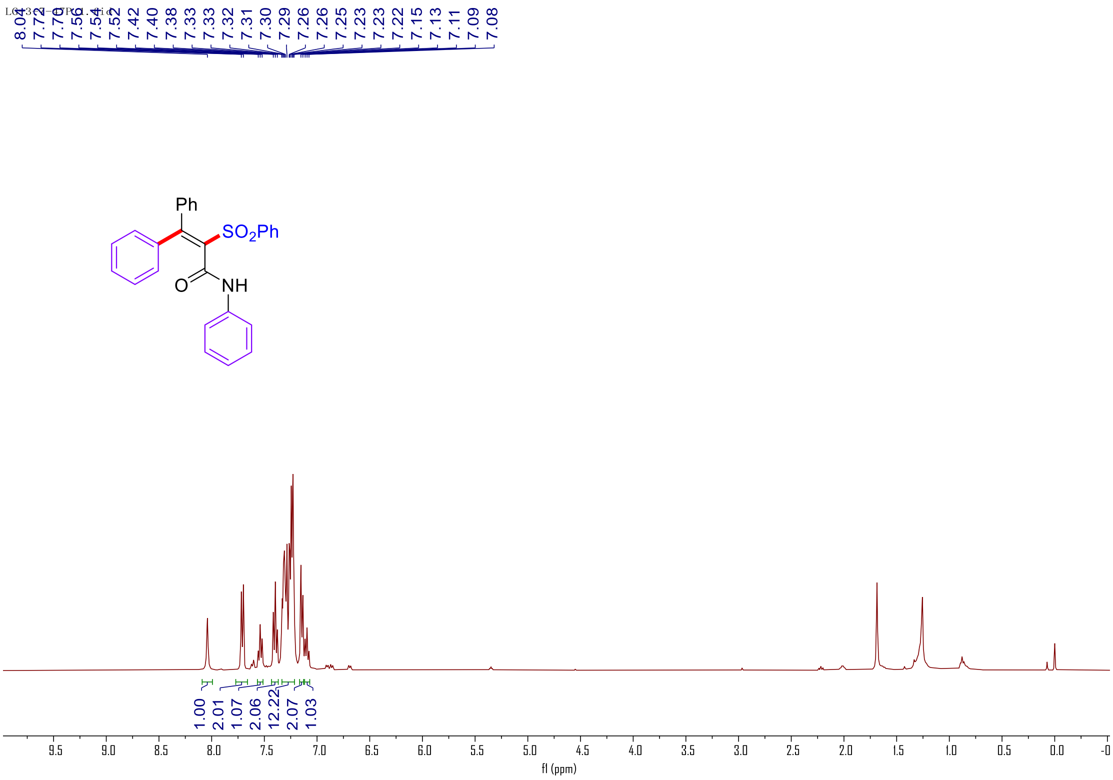

^{13}C {^{1}H} NMR Spectrum of *N*,3,3-triphenyl-2-(phenylsulfonyl)acrylamide (10, $CDCl_3$ as solvent, 101 MHz)

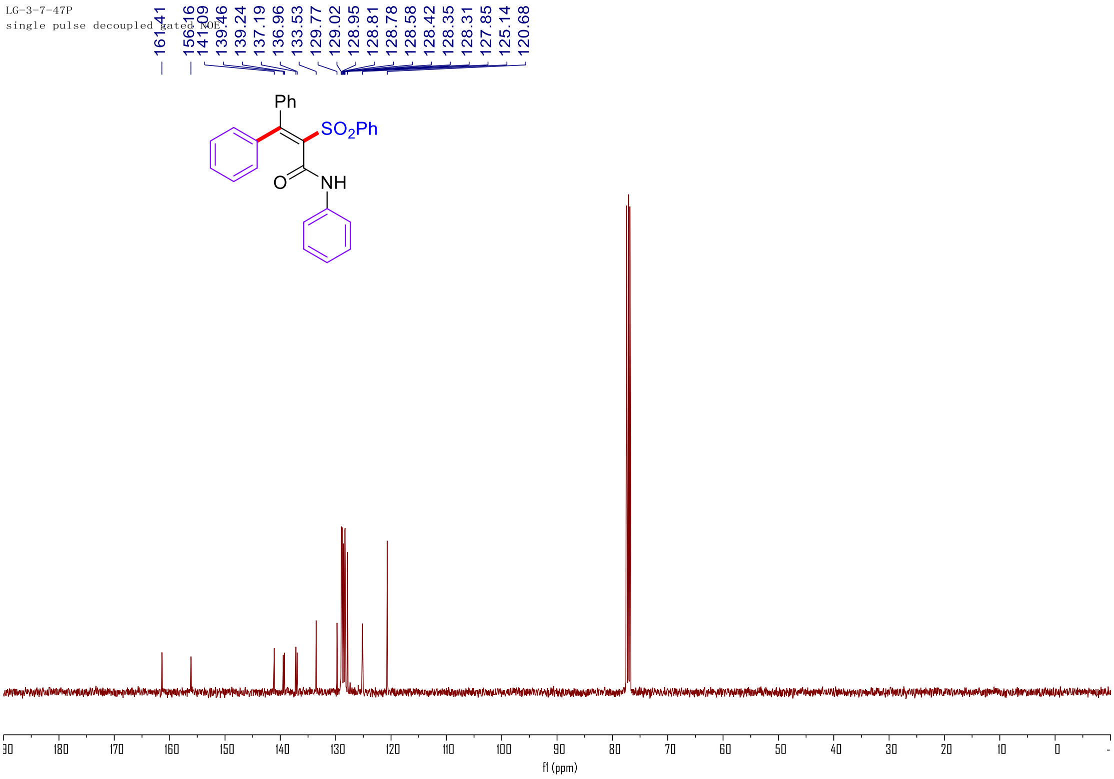

¹H NMR Spectrum of *N*-(4-fluorophenyl)-3,3-diphenyl-2-(phenylsulfonyl)acrylamide and 3-(4-fluorophenyl)-*N*,3-diphenyl-2-(phenylsulfonyl)acrylamide (11a and 11b, $CDCl_3$ as solvent, 400 MHz)

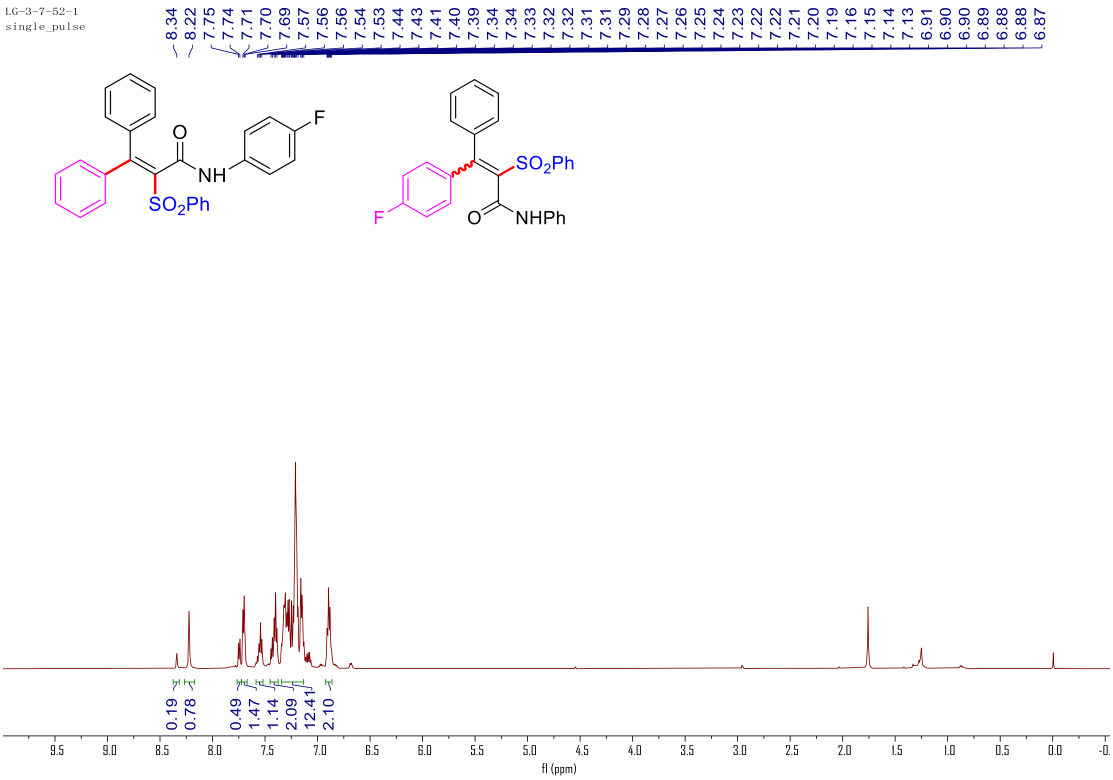

¹³C {¹H} NMR Spectrum of *N*-(4-fluorophenyl)-3,3-diphenyl-2-(phenylsulfonyl)acrylamide and 3-(4-fluorophenyl)-*N*,3-diphenyl-2-(phenylsulfonyl)acrylamide (11a and 11b, $CDCl_3$ as solvent, 101 MHz)

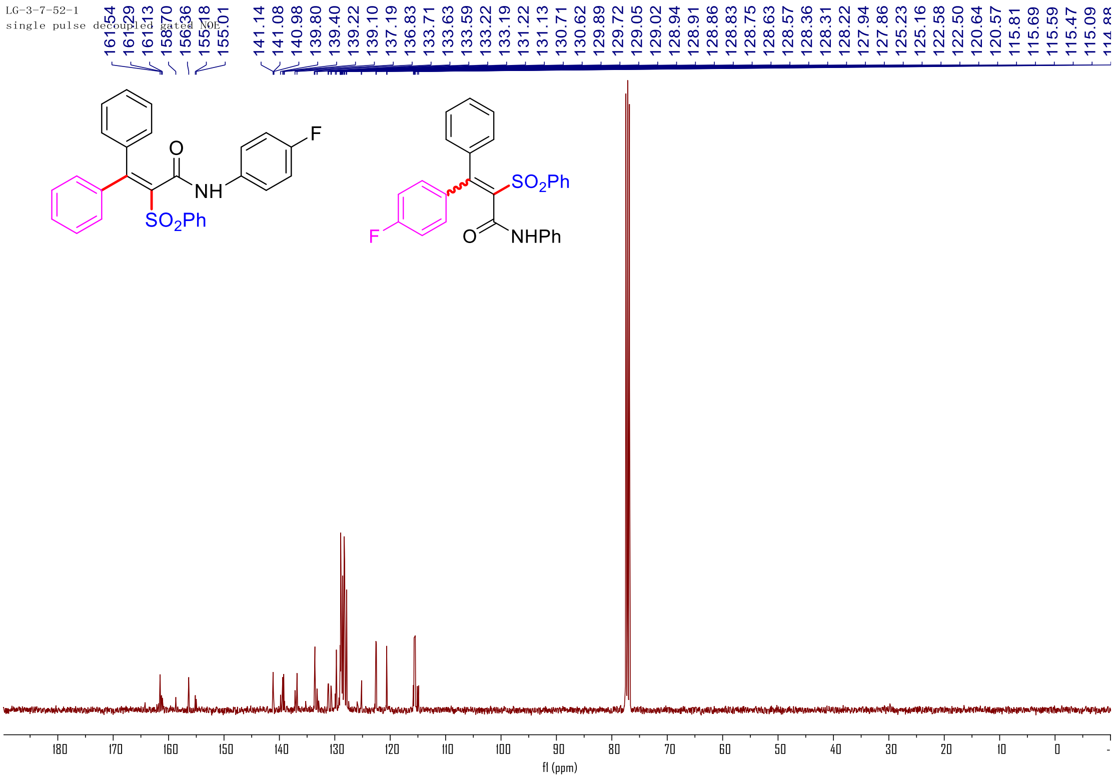

^{19}F NMR Spectrum of *N*-(4-fluorophenyl)-3,3-diphenyl-2-(phenylsulfonyl)acrylamide and 3-(4-fluorophenyl)-*N*,3-diphenyl-2-(phenylsulfonyl)acrylamide (11a and 11b, $CDCl_3$ as solvent, 376 MHz)

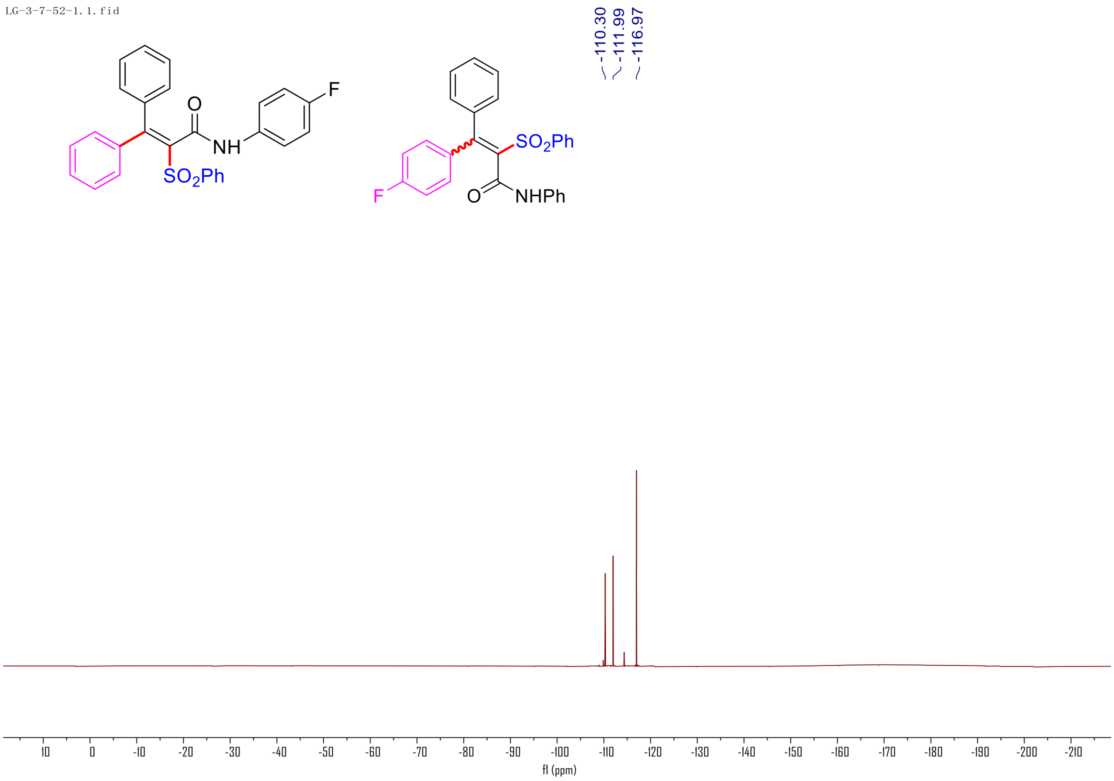

^{1}H NMR Spectrum of *N*-(4-methoxyphenyl)-3,3-diphenyl-2-(phenylsulfonyl)acrylamide (12a, $CDCl_3$ as solvent, 400 MHz)

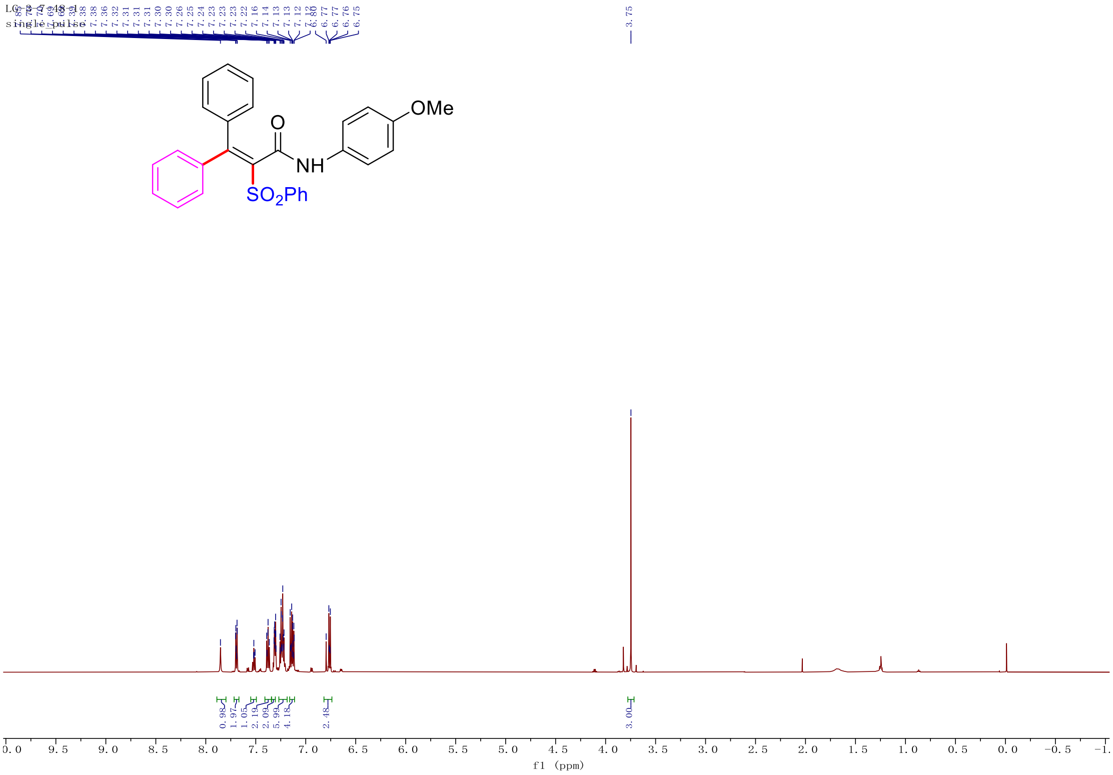

^{13}C {^{1}H} NMR Spectrum of *N*-(4-methoxyphenyl)-3,3-diphenyl-2-(phenylsulfonyl)acrylamide (12a, $CDCl_3$ as solvent, 101 MHz)

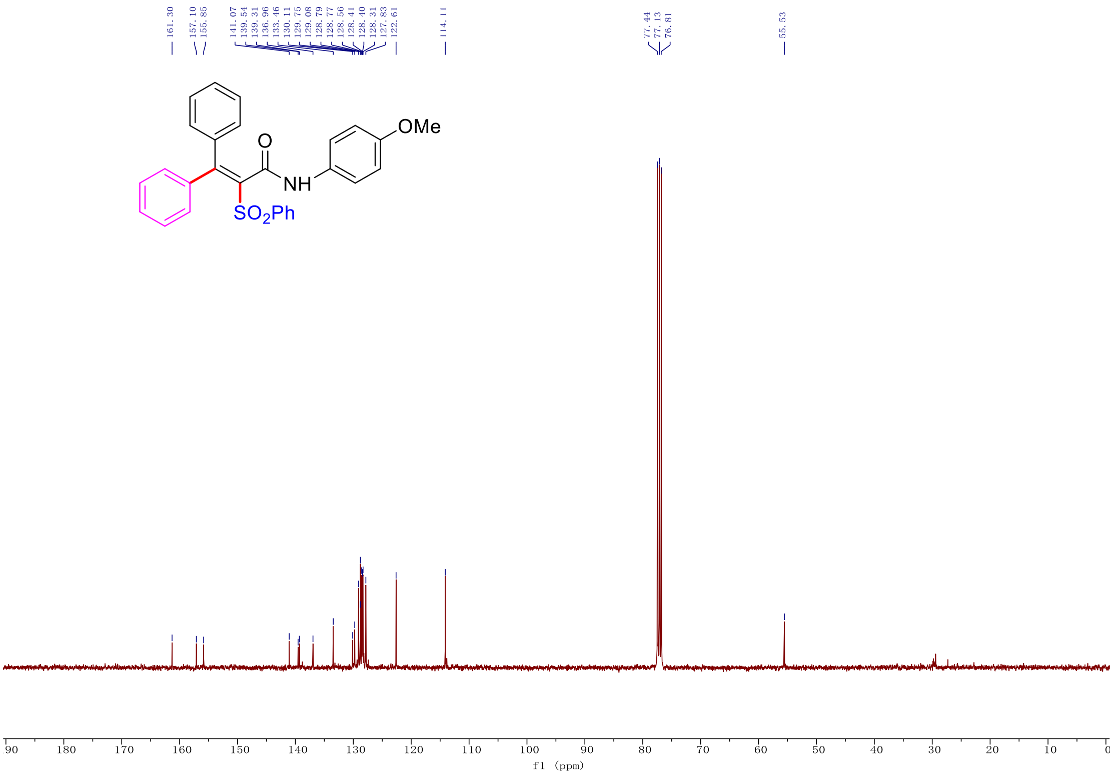

^{1}H NMR Spectrum of 3-(4-methoxyphenyl)-*N*,3-diphenyl-2-(phenylsulfonyl)acrylamide (12b, $CDCl_3$ as solvent, 400 MHz)

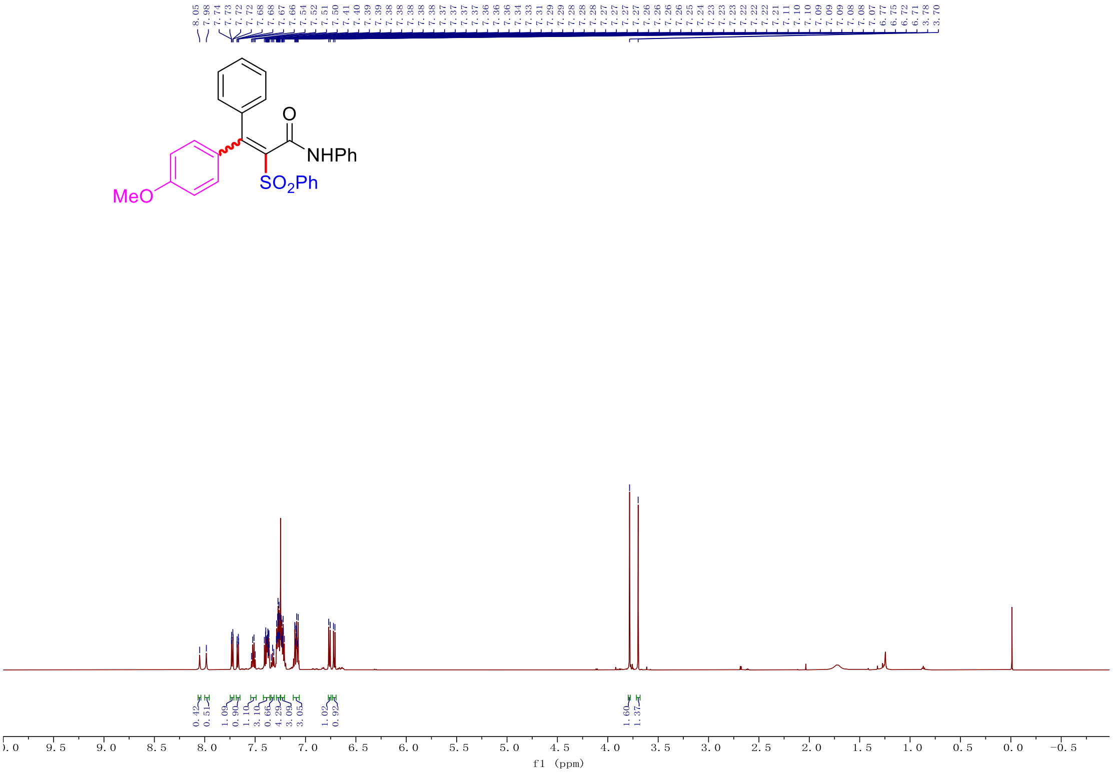

^{13}C {^{1}H} NMR Spectrum of 3-(4-methoxyphenyl)-*N*,3-diphenyl-2-(phenylsulfonyl)acrylamide (12b, $CDCl_3$ as solvent, 101 MHz)

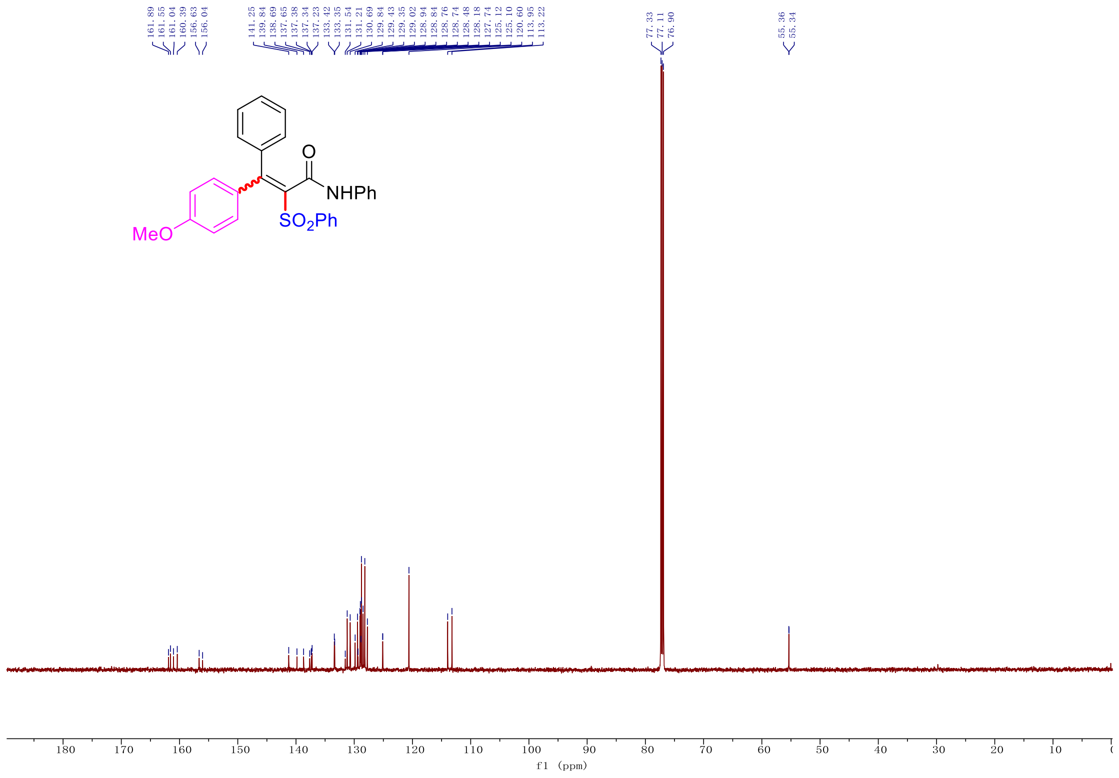

^{1}H NMR Spectrum of *N*-methyl-3-phenyl-2-(phenylsulfonyl)-3-(*p*-tolyl)acrylamide (13, $CDCl_3$ as solvent, 400 MHz)

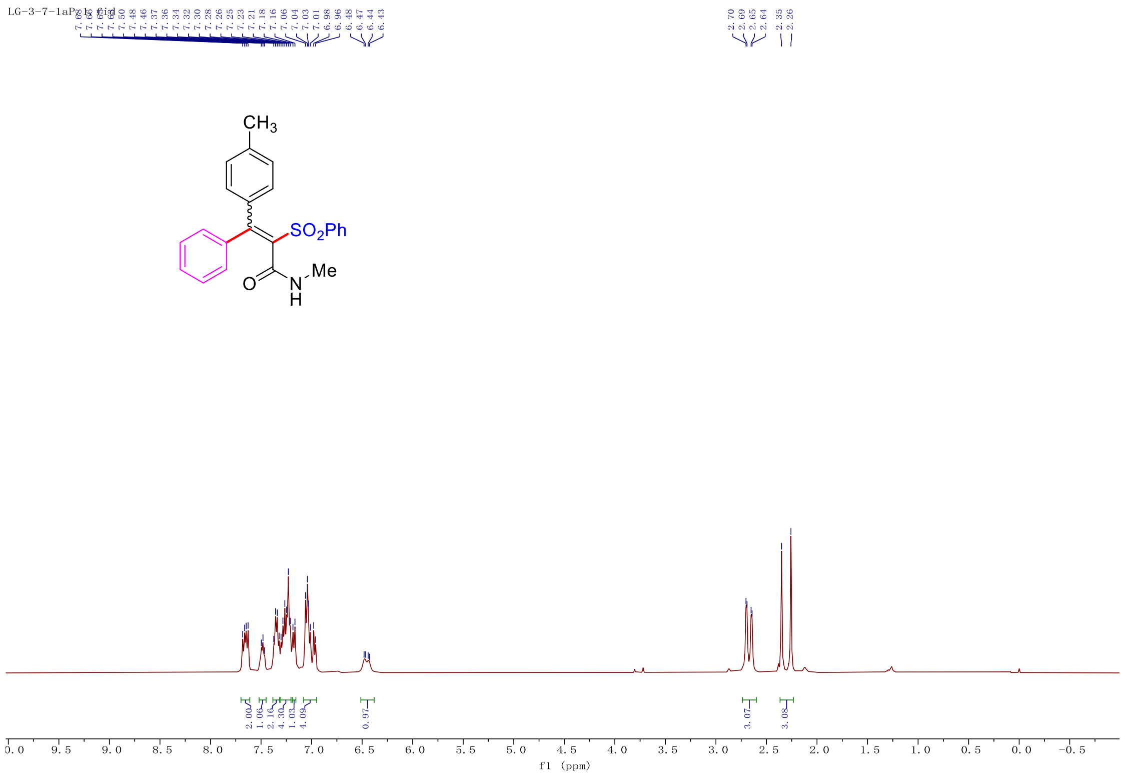

^{13}C {^{1}H} NMR Spectrum of *N*-methyl-3-phenyl-2-(phenylsulfonyl)-3-(*p*-tolyl)acrylamide (13, $CDCl_3$ as solvent, 101 MHz)

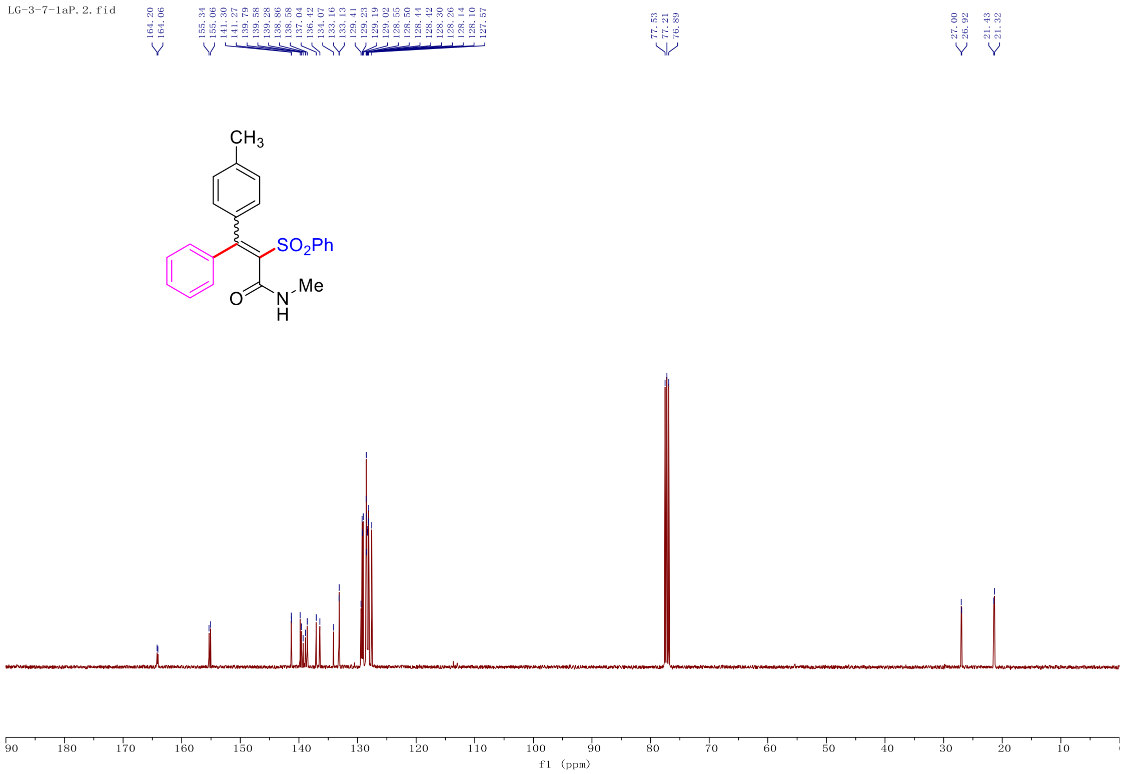

^{1}H NMR Spectrum of 3-([1,1'-biphenyl]-4-yl)-*N*-methyl-3-phenyl-2-(phenylsulfonyl)acrylamide (14, $CDCl_3$ as solvent, 400 MHz)

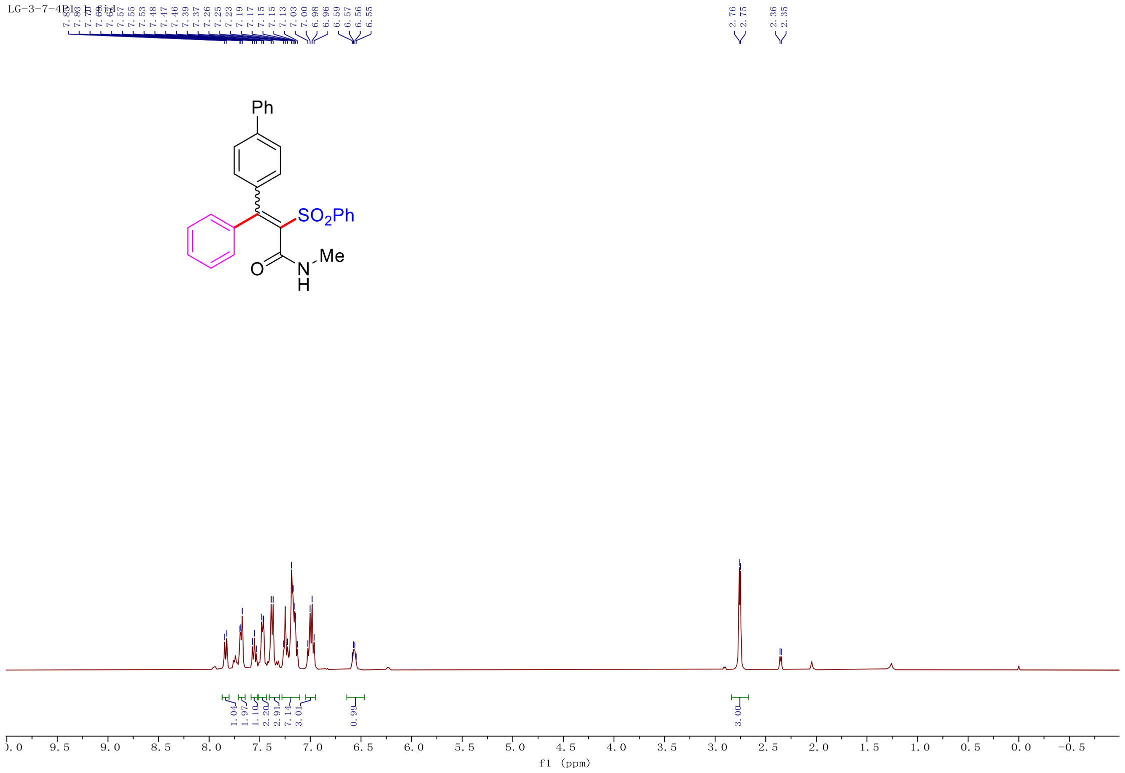

^{13}C {^{1}H} NMR Spectrum of 3-([1,1'-biphenyl]-4-yl)-*N*-methyl-3-phenyl-2-(phenylsulfonyl)acrylamide (14, $CDCl_3$ as solvent, 101 MHz)

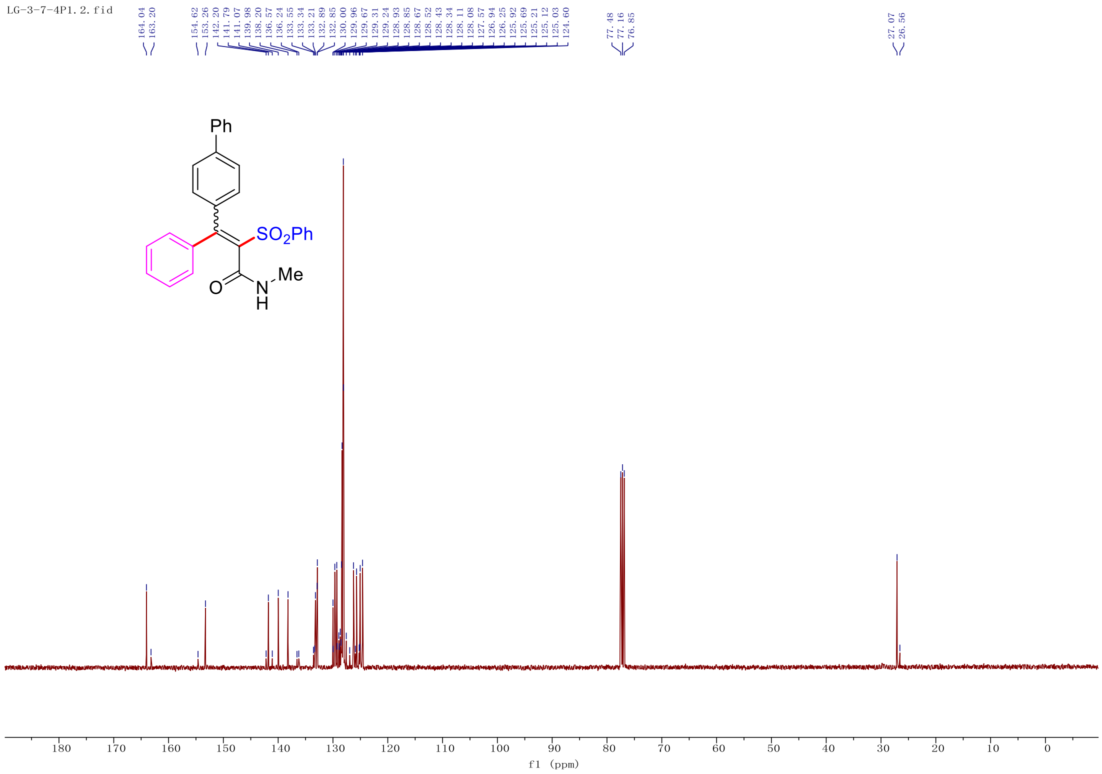

^{1}H NMR Spectrum of 3-(4-hydroxyphenyl)-*N*-methyl-3-phenyl-2-(phenylsulfonyl)acrylamide (15, DMSO-d_6 as solvent, 400 MHz)

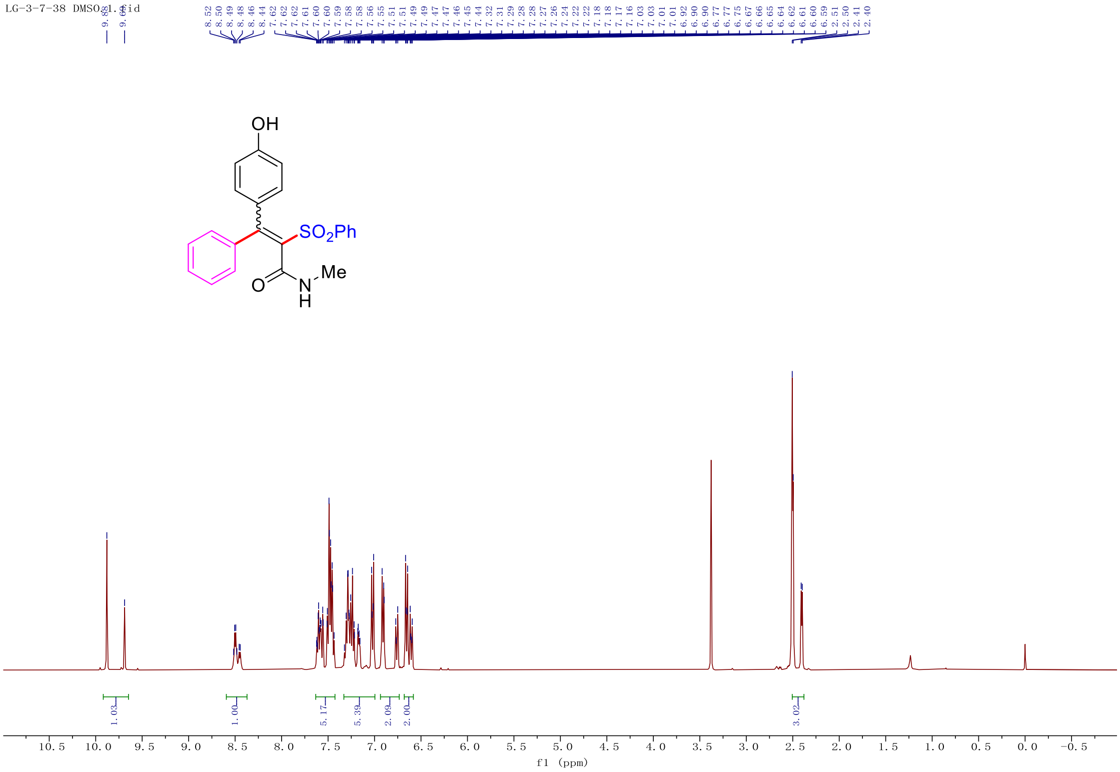

^{13}C {^{1}H} NMR Spectrum of 3-(4-hydroxyphenyl)-*N*-methyl-3-phenyl-2-(phenylsulfonyl)acrylamide (15, DMSO-d_6 as solvent, 101 MHz)

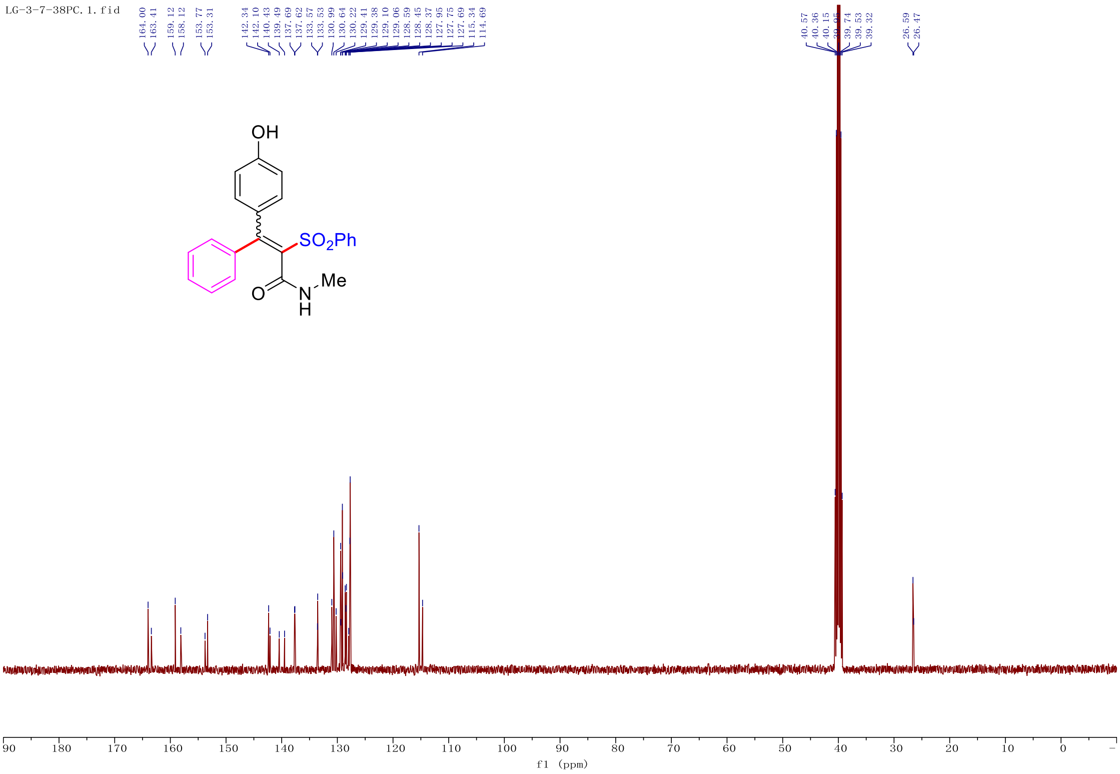

^{1}H NMR Spectrum of 3-(4-methoxyphenyl)-*N*-methyl-3-phenyl-2-(phenylsulfonyl)acrylamide (16, $CDCl_3$ as solvent, 400 MHz)

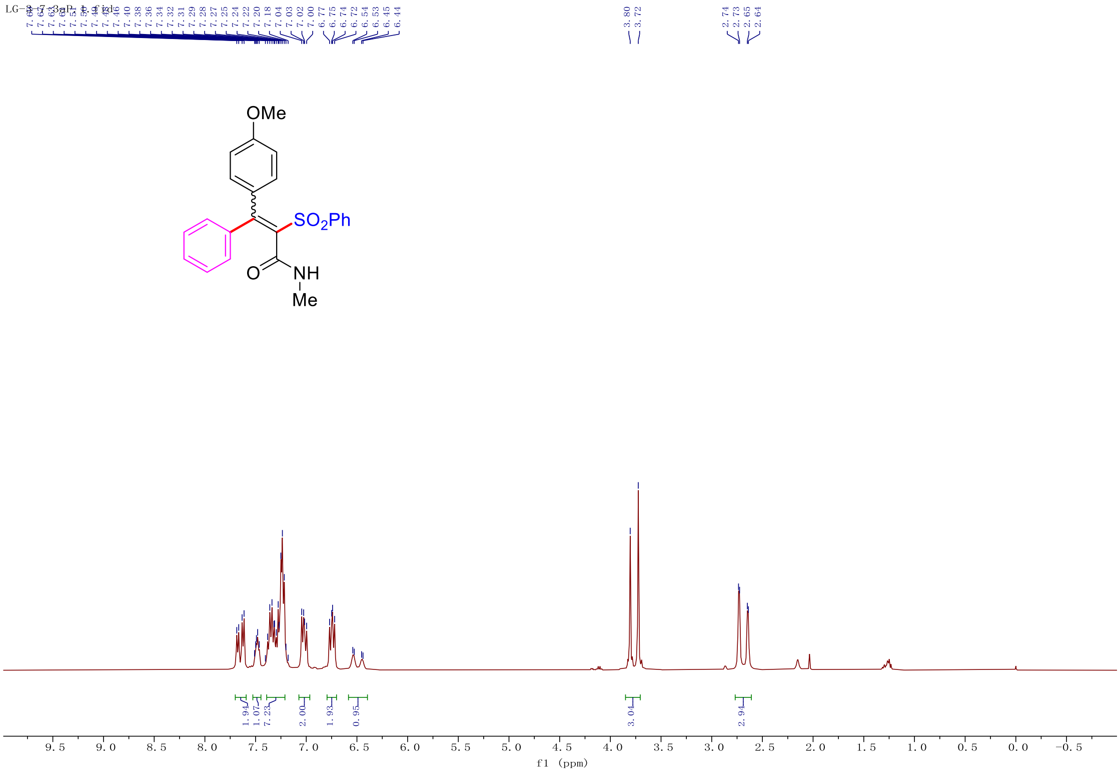

$^{13}C\ \{^{1}H\}$ NMR Spectrum of 3-(4-methoxyphenyl)-*N*-methyl-3-phenyl-2-(phenylsulfonyl)acrylamide (16, $CDCl_3$ as solvent, 101 MHz)

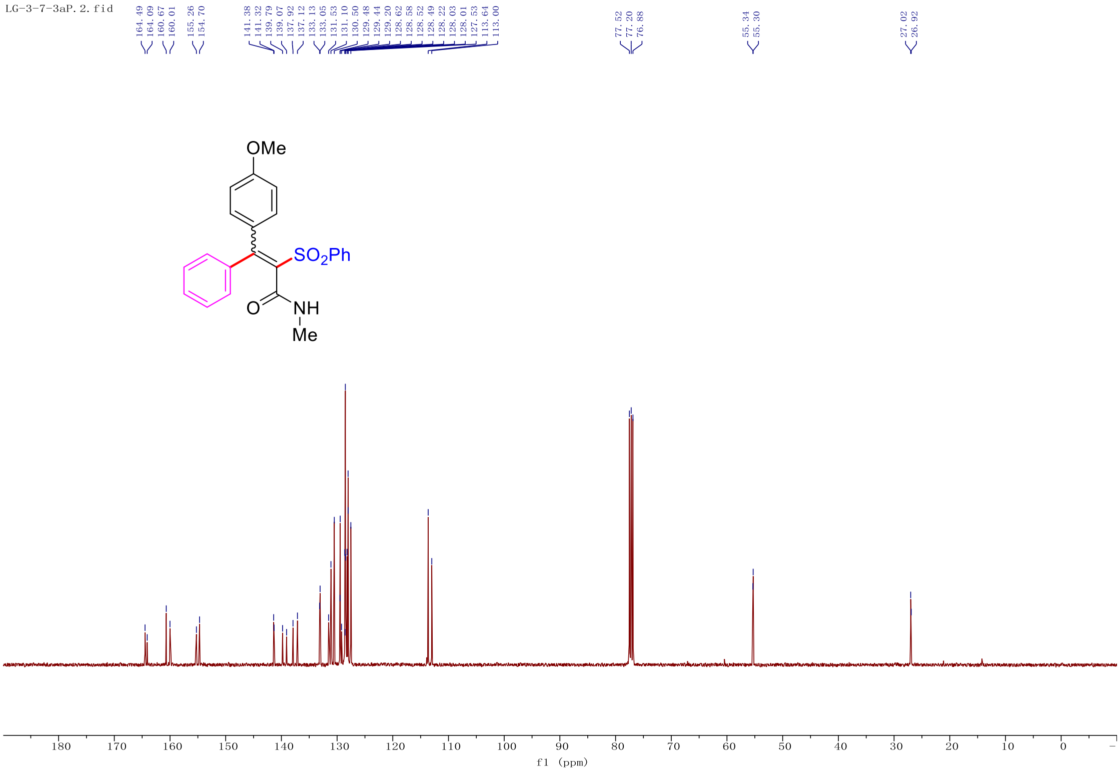

^{1}H NMR Spectrum of *N*-methyl-3-phenyl-2-(phenylsulfonyl)-3-(4-(trifluoromethoxy)phenyl)acrylamide (17, $CDCl_3$ as solvent, 400 MHz)

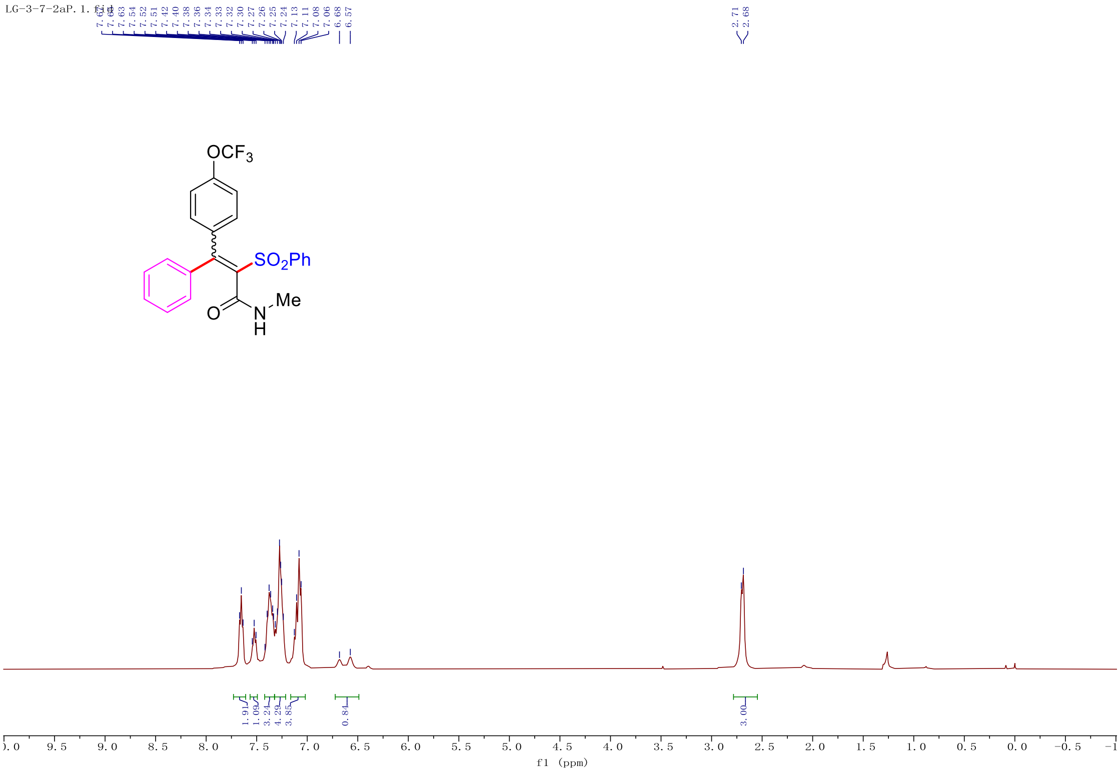

[13] C {[1] H} NMR Spectrum of *N*-methyl-3-phenyl-2-(phenylsulfonyl)-3-(4-(trifluoromethoxy)phenyl)acrylamide (17, $CDCl_3$ as solvent, 101 MHz)

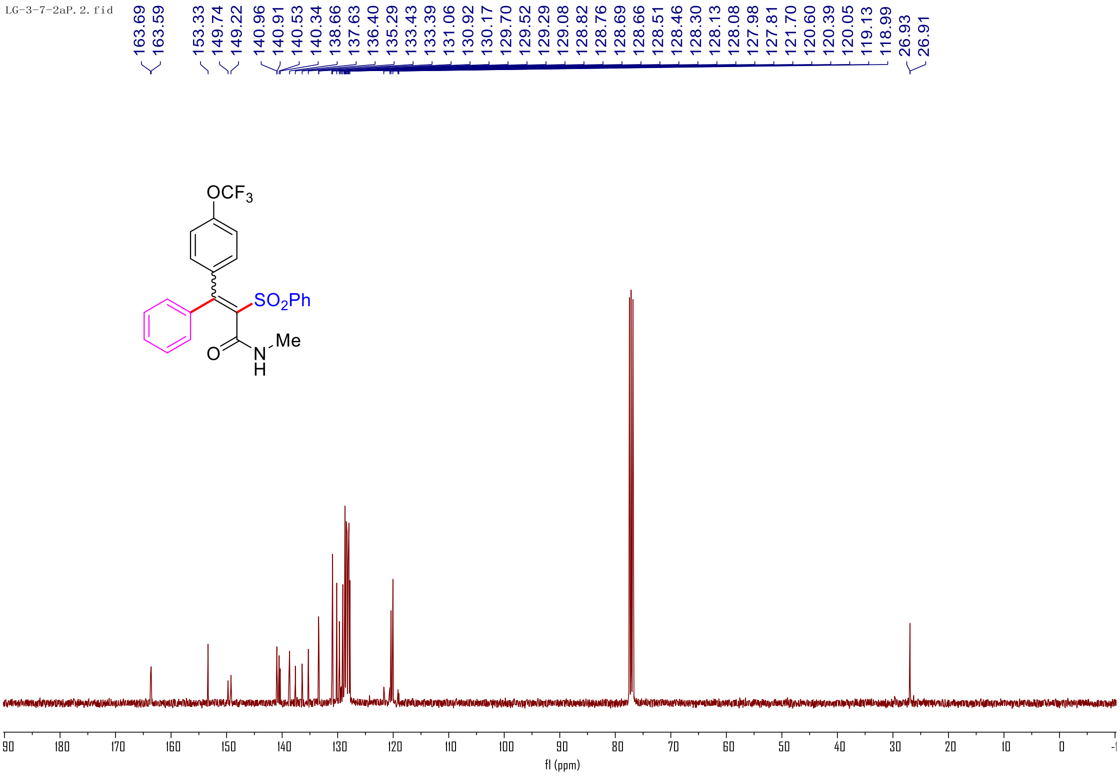

^{19}F NMR Spectrum of *N*-methyl-3-phenyl-2-(phenylsulfonyl)-3-(4-(trifluoromethoxy)phenyl)acrylamide (17, $CDCl_3$ as solvent, 376 MHz)

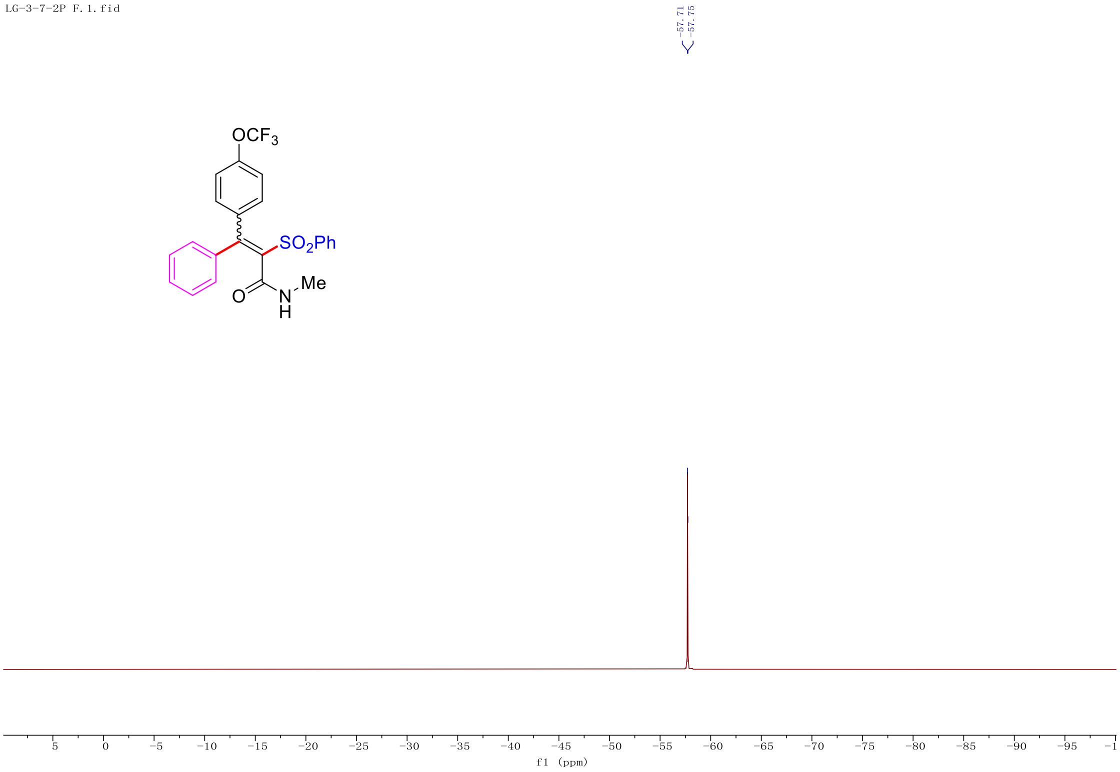

^{1}H NMR Spectrum of *N*-methyl-3-phenyl-2-(phenylsulfonyl)-3-(4-(trifluoromethyl)phenyl)acrylamide (18, $CDCl_3$ as solvent, 400 MHz)

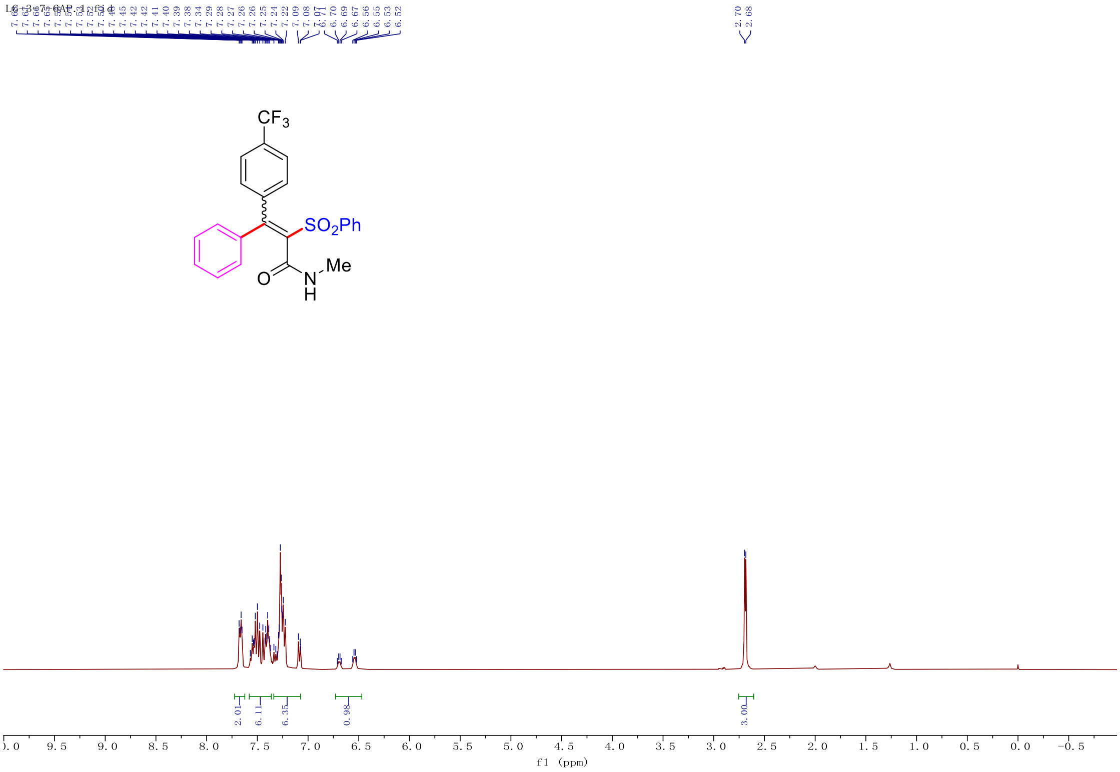

^{13}C {^{1}H} NMR Spectrum of *N*-methyl-3-phenyl-2-(phenylsulfonyl)-3-(4-(trifluoromethyl)phenyl)acrylamide (18, $CDCl_3$ as solvent, 101 MHz)

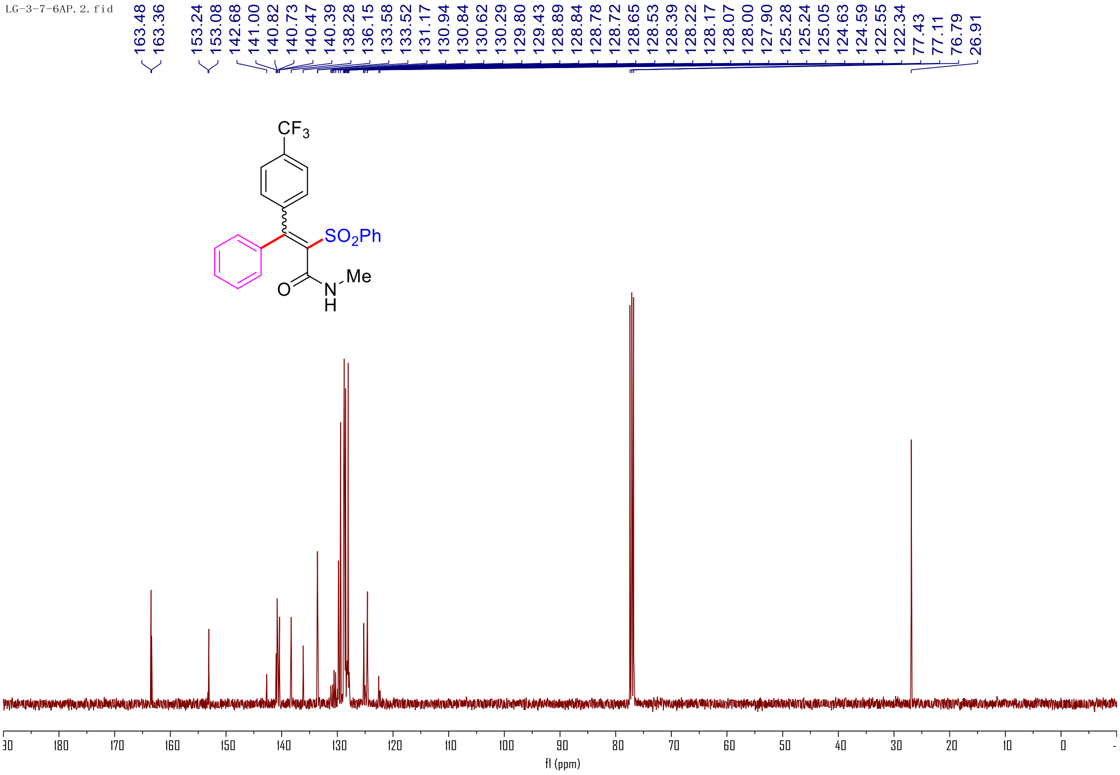

^{19}F NMR Spectrum of *N*-methyl-3-phenyl-2-(phenylsulfonyl)-3-(4-(trifluoromethyl)phenyl)acrylamide (18, $CDCl_3$ as solvent, 376 MHz)

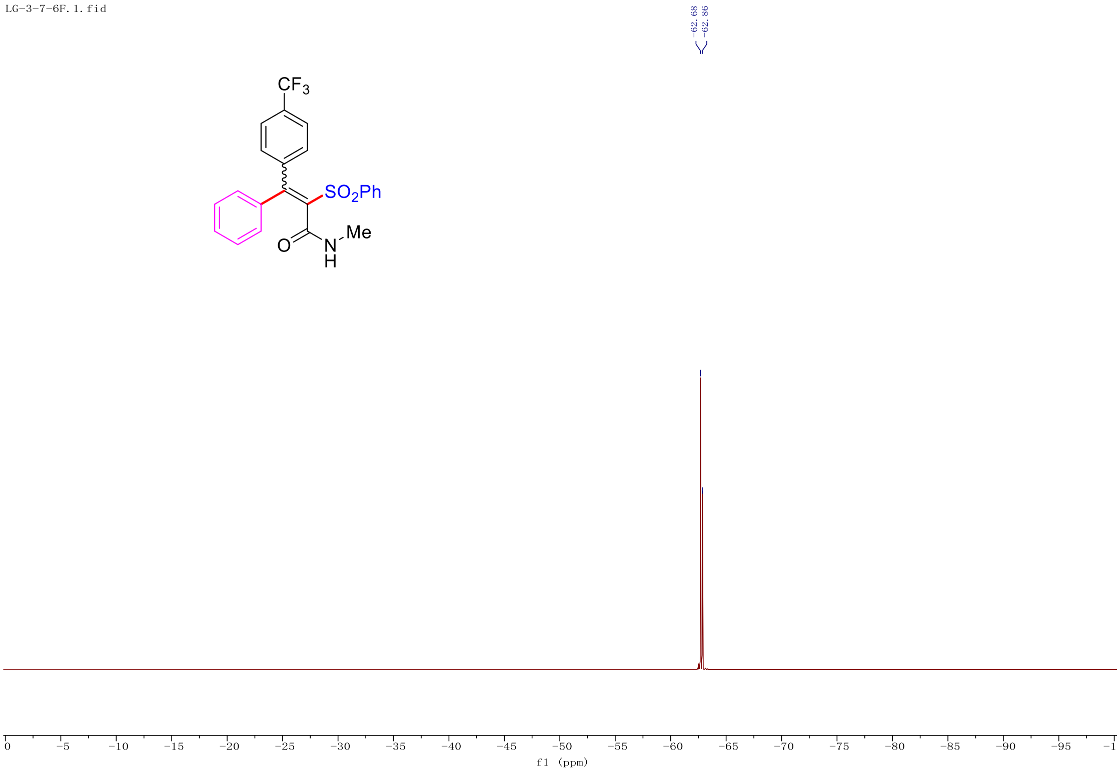

^{1}H NMR Spectrum of *N*-methyl-3-phenyl-2-(phenylsulfonyl)-3-(*m*-tolyl)acrylamide (19, $CDCl_3$ as solvent, 400 MHz)

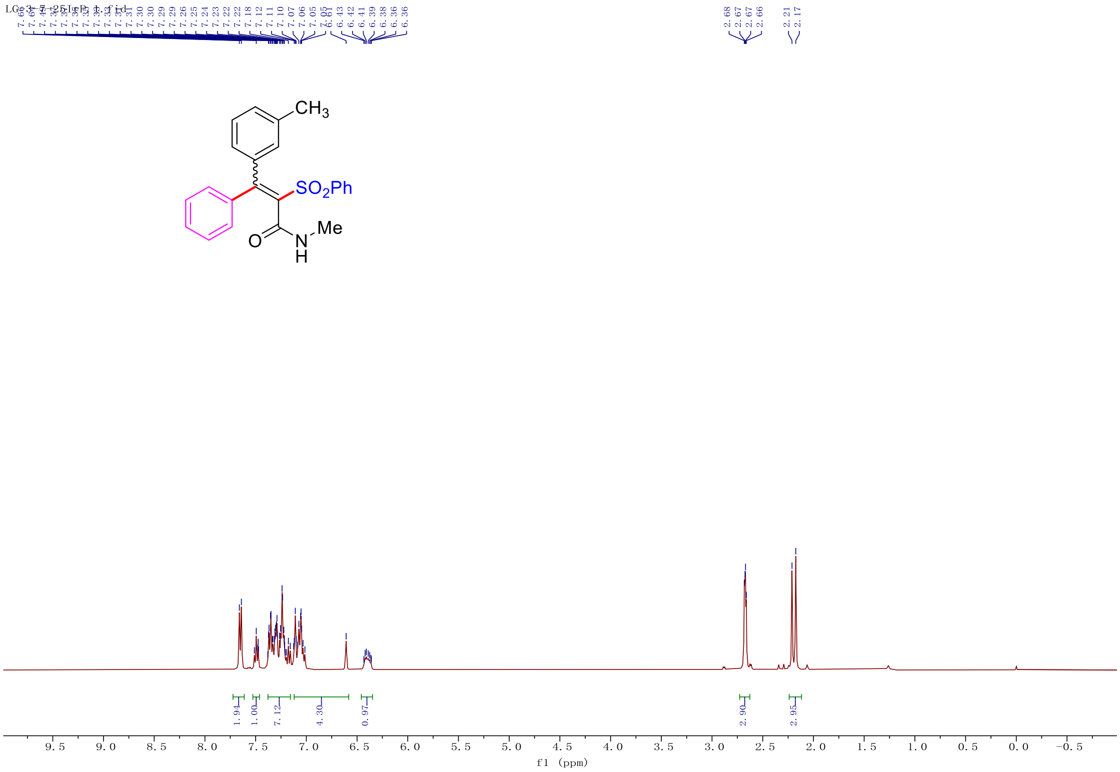

^{13}C {^{1}H} NMR Spectrum of *N*-methyl-3-phenyl-2-(phenylsulfonyl)-3-(*m*-tolyl)acrylamide (19, CDCl$_3$ as solvent, 101 MHz)

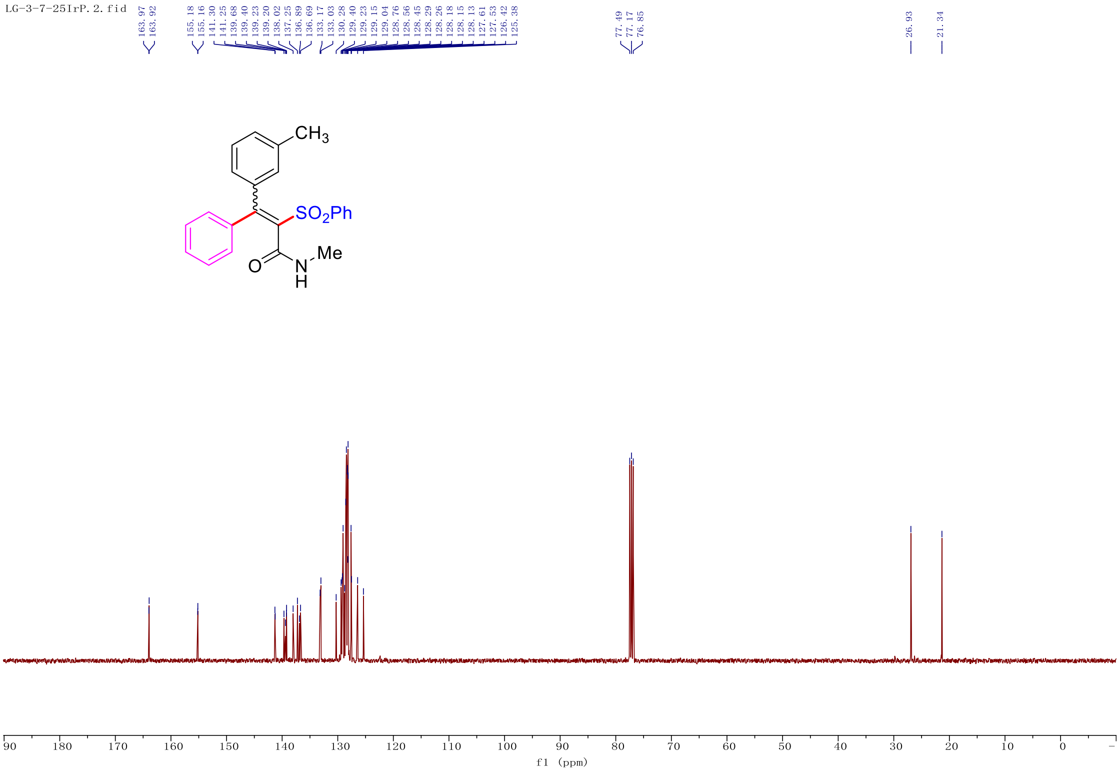

^{1}H NMR Spectrum of *N*-methyl-3-(naphthalen-1-yl)-3-phenyl-2-(phenylsulfonyl)acrylamide (20, CDCl$_3$ as solvent, 400 MHz)

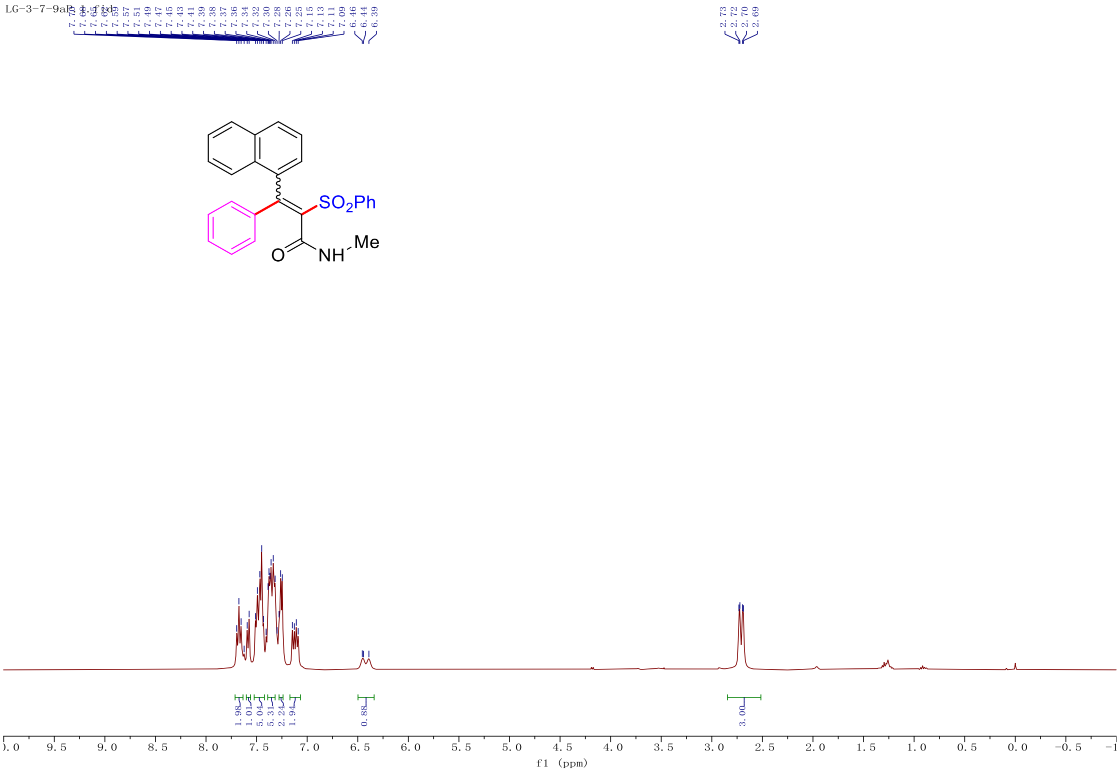

^{13}C {^{1}H} NMR Spectrum of *N*-methyl-3-(naphthalen-1-yl)-3-phenyl-2-(phenylsulfonyl)acrylamide (20, $CDCl_3$ as solvent, 101 MHz)

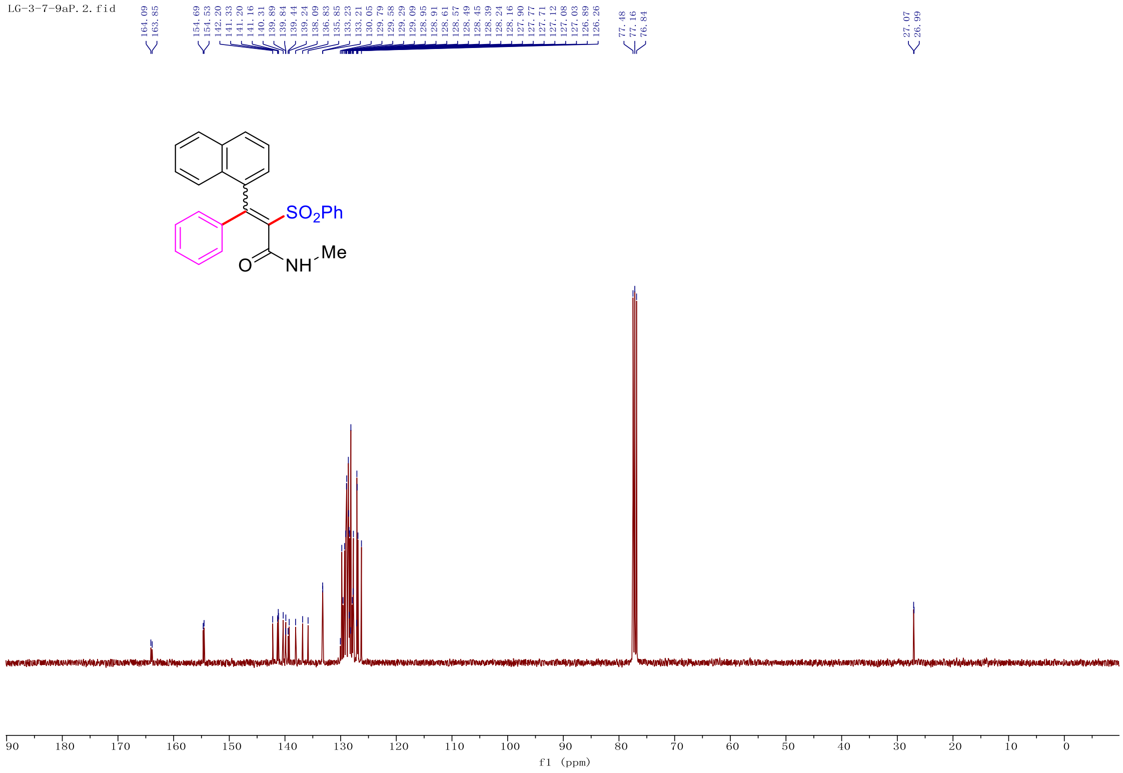

^{1}H NMR Spectrum of 3-(furan-3-yl)-*N*-methyl-3-phenyl-2-(phenylsulfonyl)acrylamide (21, $CDCl_3$ as solvent, 400 MHz)

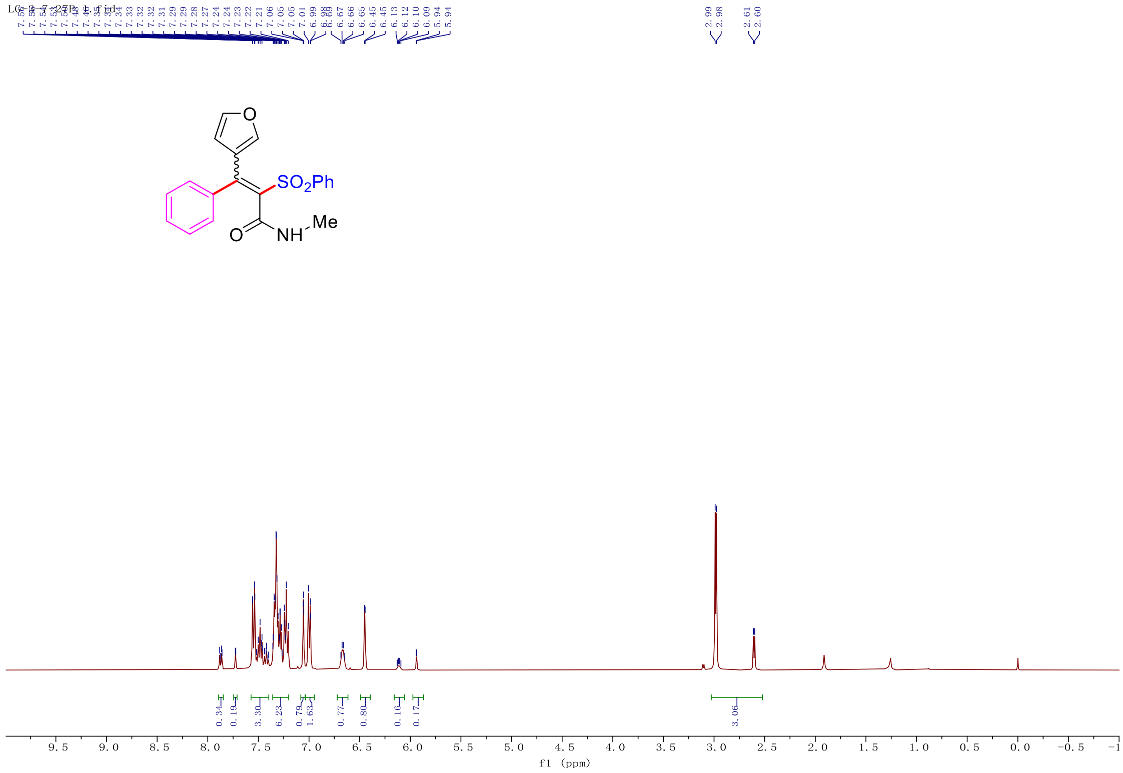

^{13}C {^{1}H} NMR Spectrum of 3-(furan-3-yl)-*N*-methyl-3-phenyl-2-(phenylsulfonyl)acrylamide (21, $CDCl_3$ as solvent, 101 MHz)

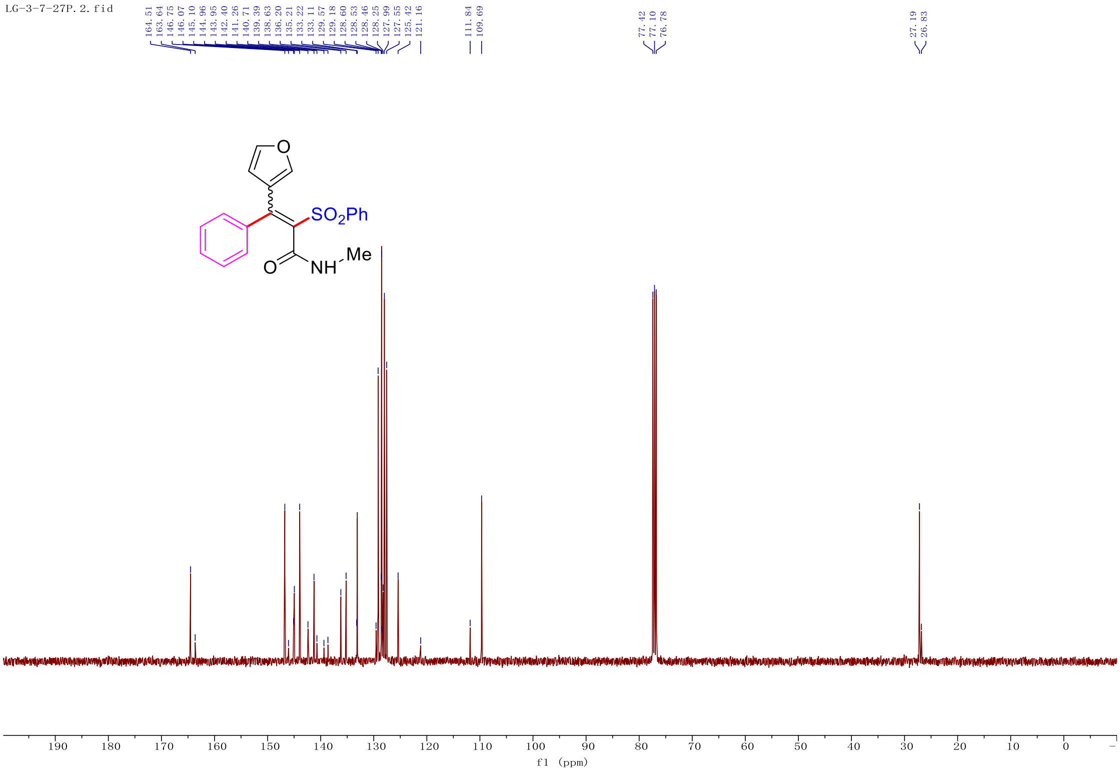

^{1}H NMR Spectrum of *N*-methyl-3-(1-methyl-1*H*-indol-5-yl)-3-phenyl-2-(phenylsulfonyl)acrylamide (22, $CDCl_3$ as solvent, 400 MHz)

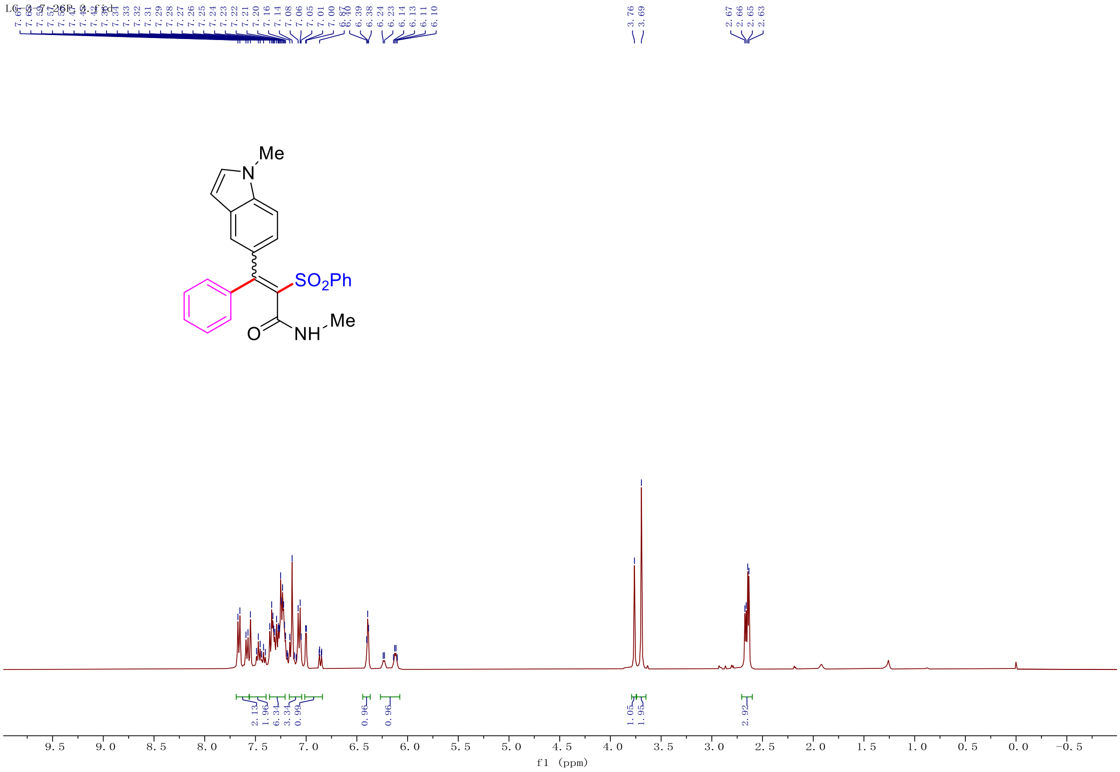

^{13}C {^{1}H} NMR Spectrum of *N*-methyl-3-(1-methyl-1*H*-indol-5-yl)-3-phenyl-2-(phenylsulfonyl)acrylamide (22, $CDCl_3$ as solvent, 101 MHz)

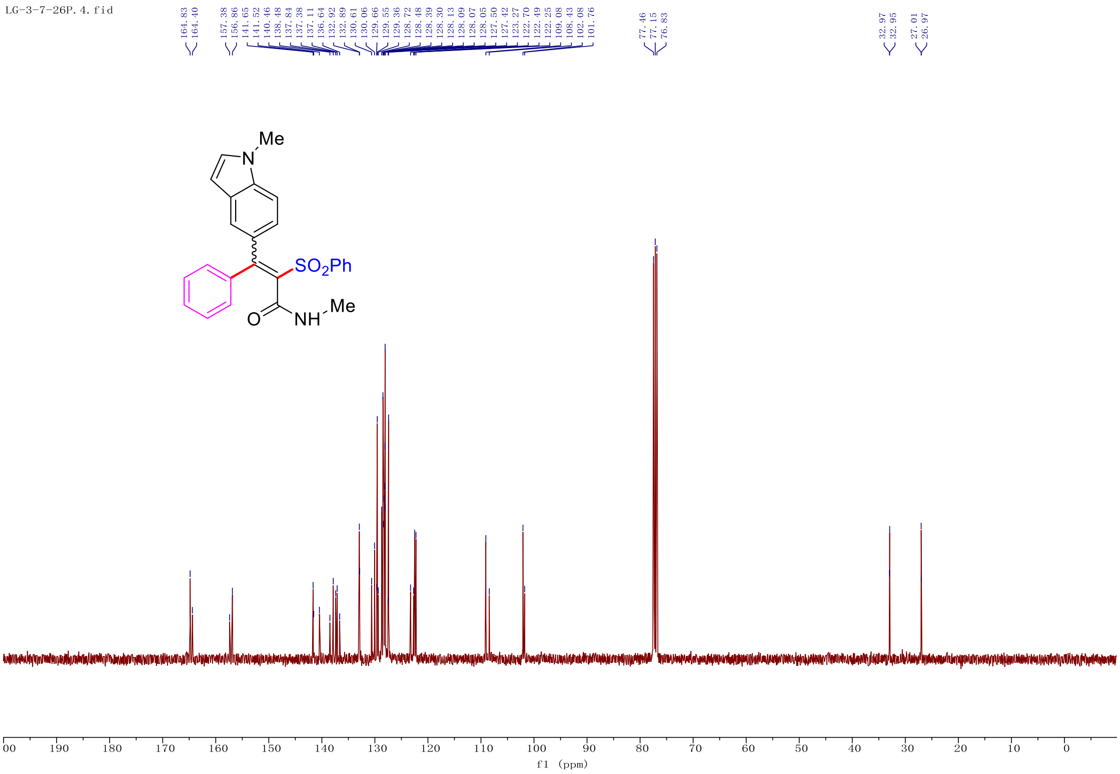

^{1}H NMR Spectrum of 3-(1*H*-indazol-6-yl)-*N*-methyl-3-phenyl-2-(phenylsulfonyl)acrylamide (23, $CDCl_3$ as solvent, 400 MHz)

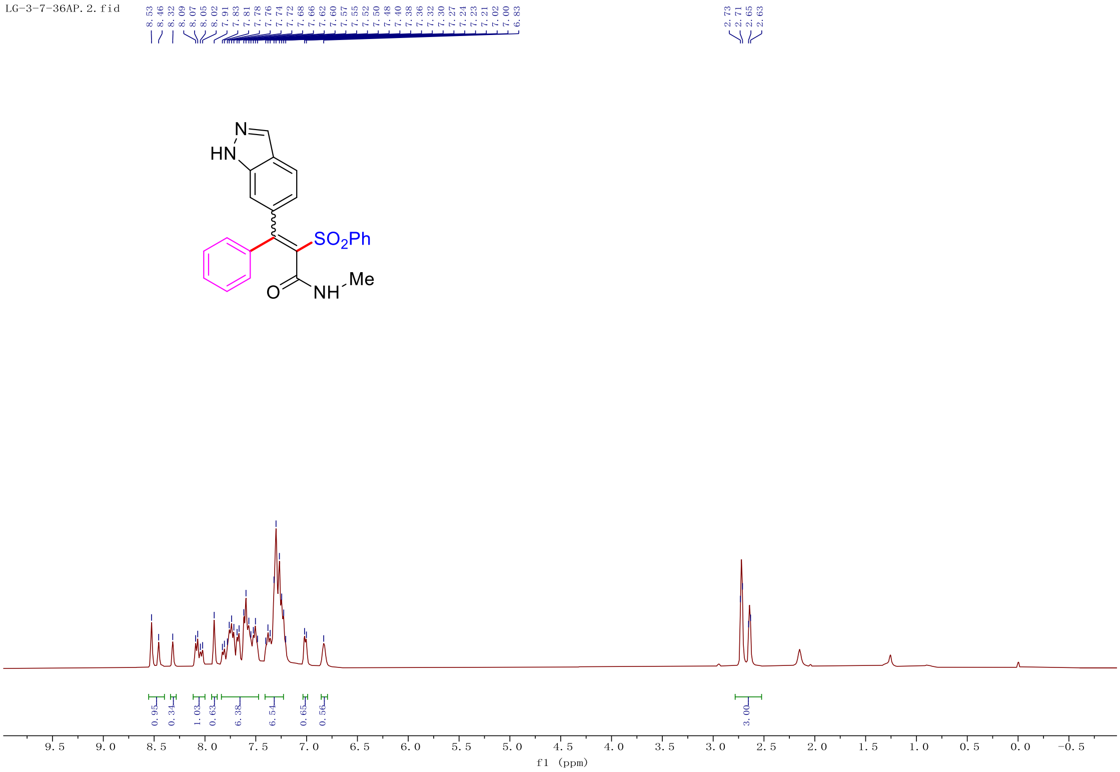

^{13}C {^{1}H} NMR Spectrum of 3-(1*H*-indazol-6-yl)-*N*-methyl-3-phenyl-2-(phenylsulfonyl)acrylamide (23, $CDCl_3$ as solvent, 101 MHz)

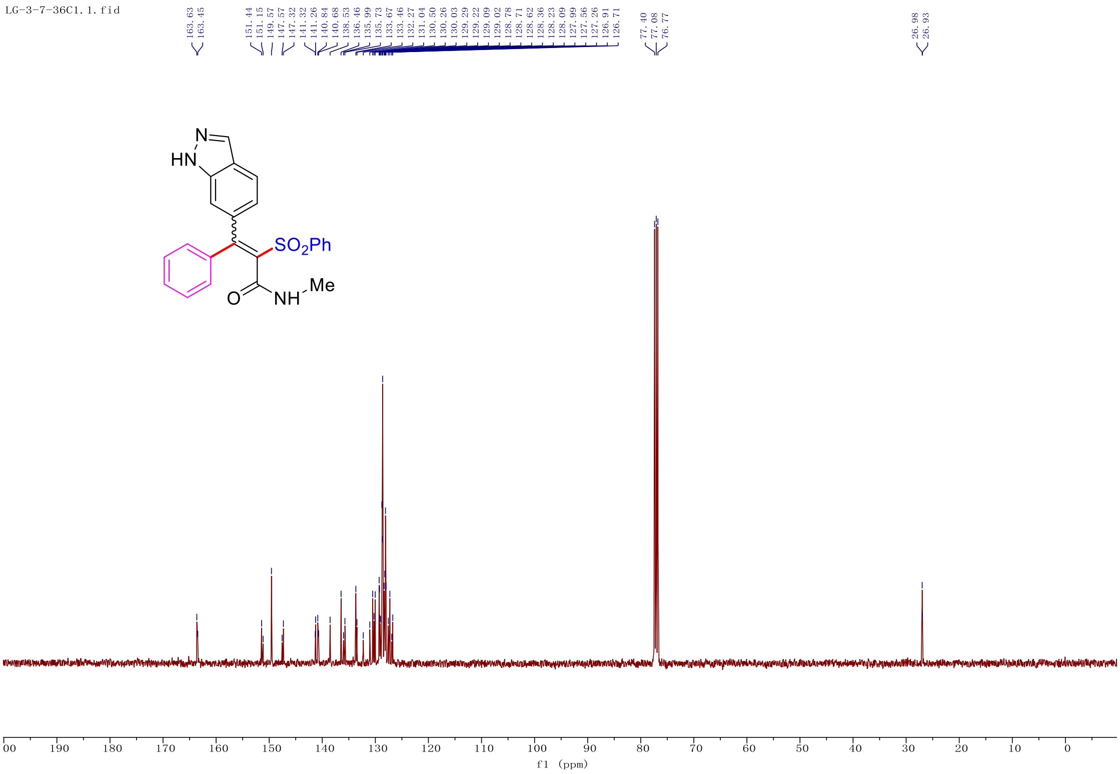

^{1}H NMR Spectrum of 3-(dibenzo[b,d]furan-4-yl)-*N*-methyl-3-phenyl-2-(phenylsulfonyl)acrylamide (24, $CDCl_3$ as solvent, 400 MHz)

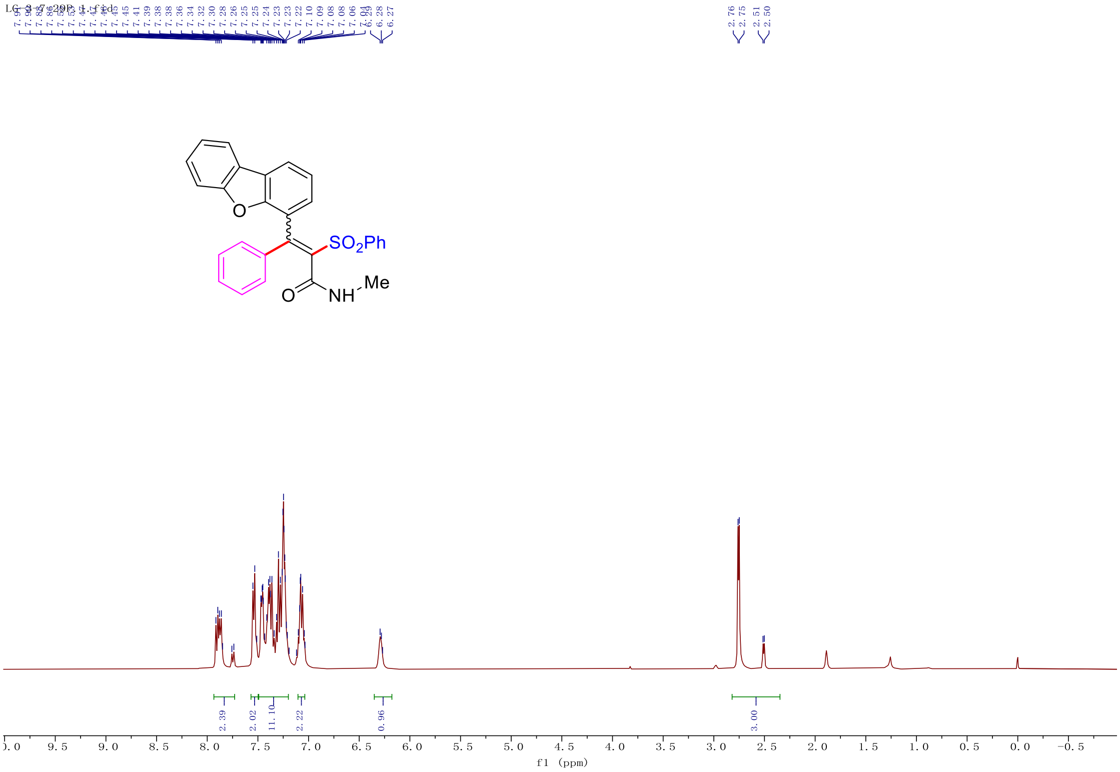

^{13}C {^{1}H} NMR Spectrum of 3-(dibenzo[b,d]furan-4-yl)-*N*-methyl-3-phenyl-2-(phenylsulfonyl)acrylamide (24, $CDCl_3$ as solvent, 101 MHz)

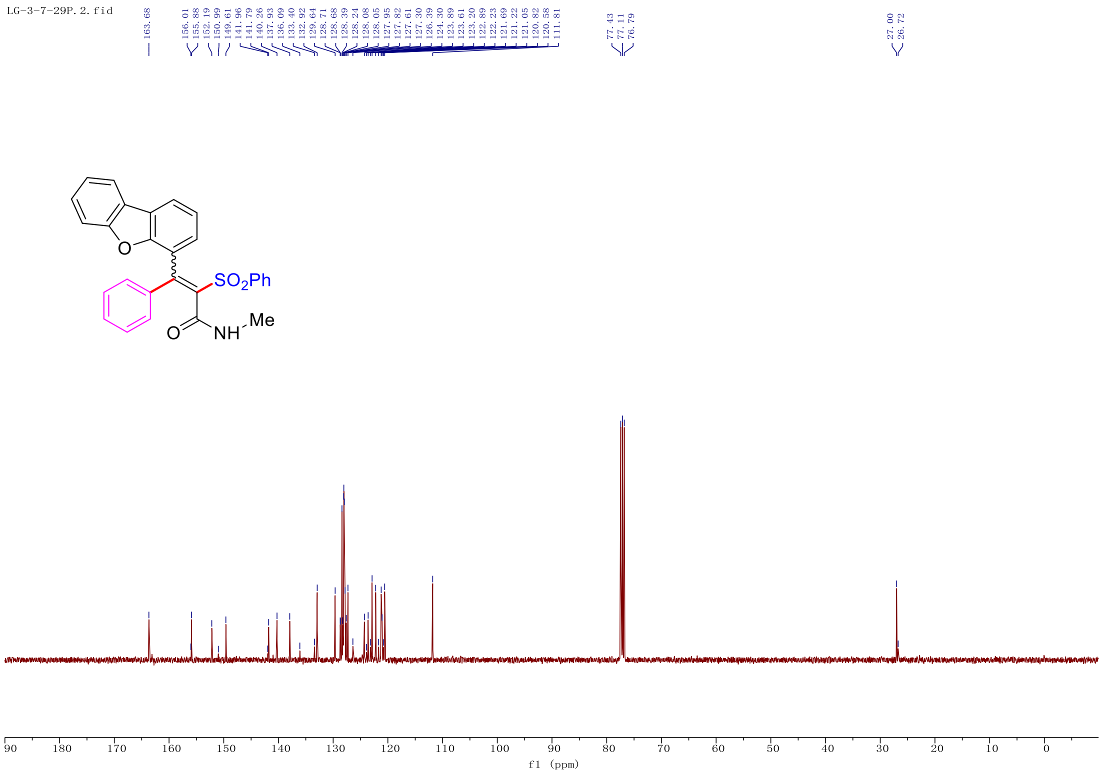

^{1}H NMR Spectrum of 3-(dibenzo[b,d]thiophen-4-yl)-*N*-methyl-3-phenyl-2-(phenylsulfonyl)acrylamide (25, $CDCl_3$ as solvent, 400 MHz)

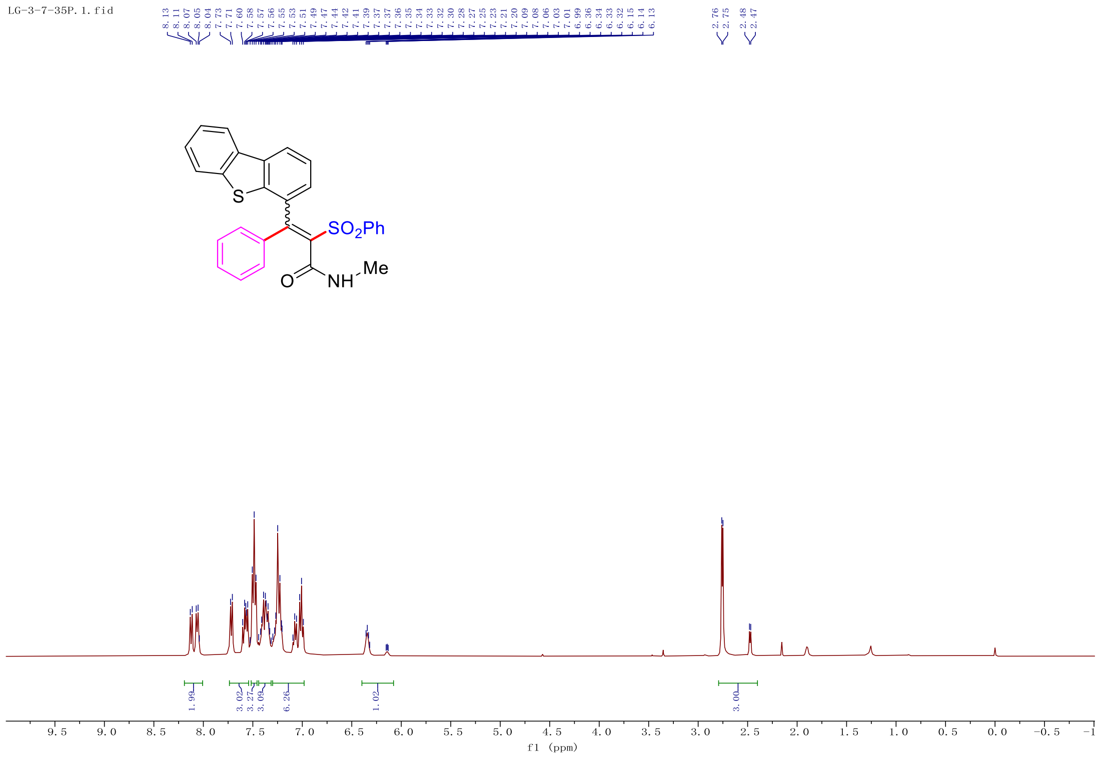

^{13}C {^{1}H} NMR Spectrum of 3-(dibenzo[b,d]thiophen-4-yl)-*N*-methyl-3-phenyl-2-(phenylsulfonyl)acrylamide (25, $CDCl_3$ as solvent, 101 MHz)

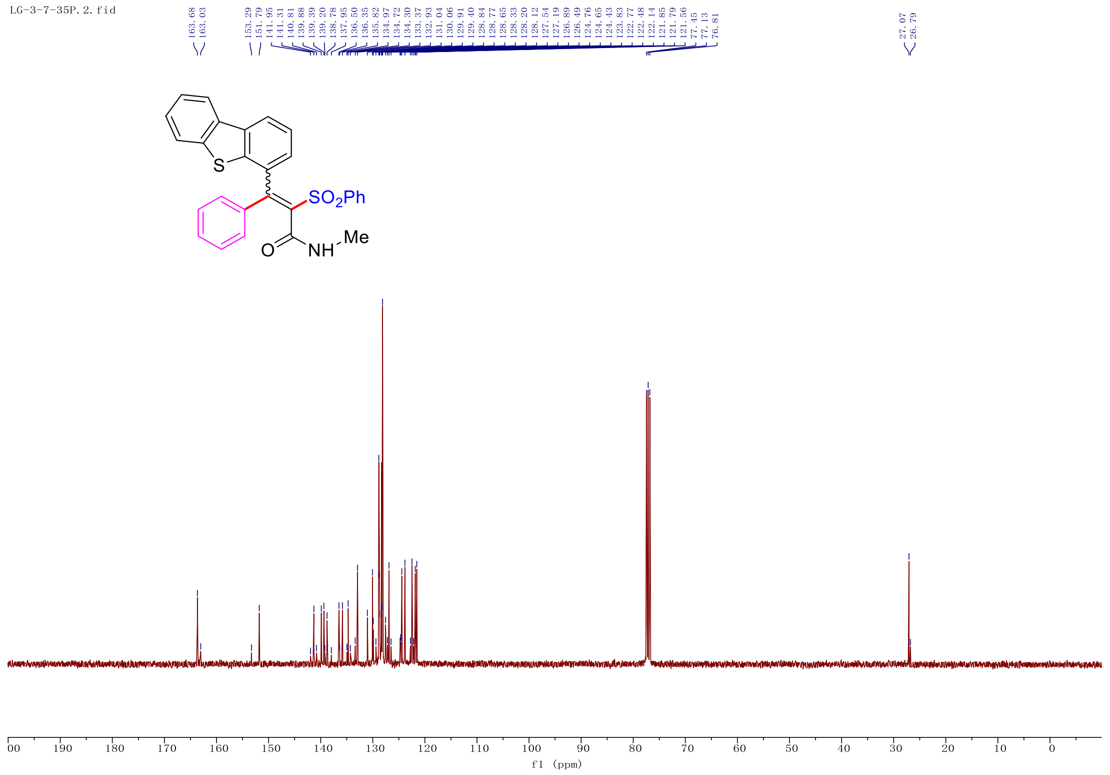

^{1}H NMR Spectrum of 4-(3-(methylamino)-3-oxo-1-phenyl-2-(phenylsulfonyl)prop-1-en-1-yl)benzyl 2-(3-benzoylphenyl)propanoate (26, $CDCl_3$ as solvent, 400 MHz)

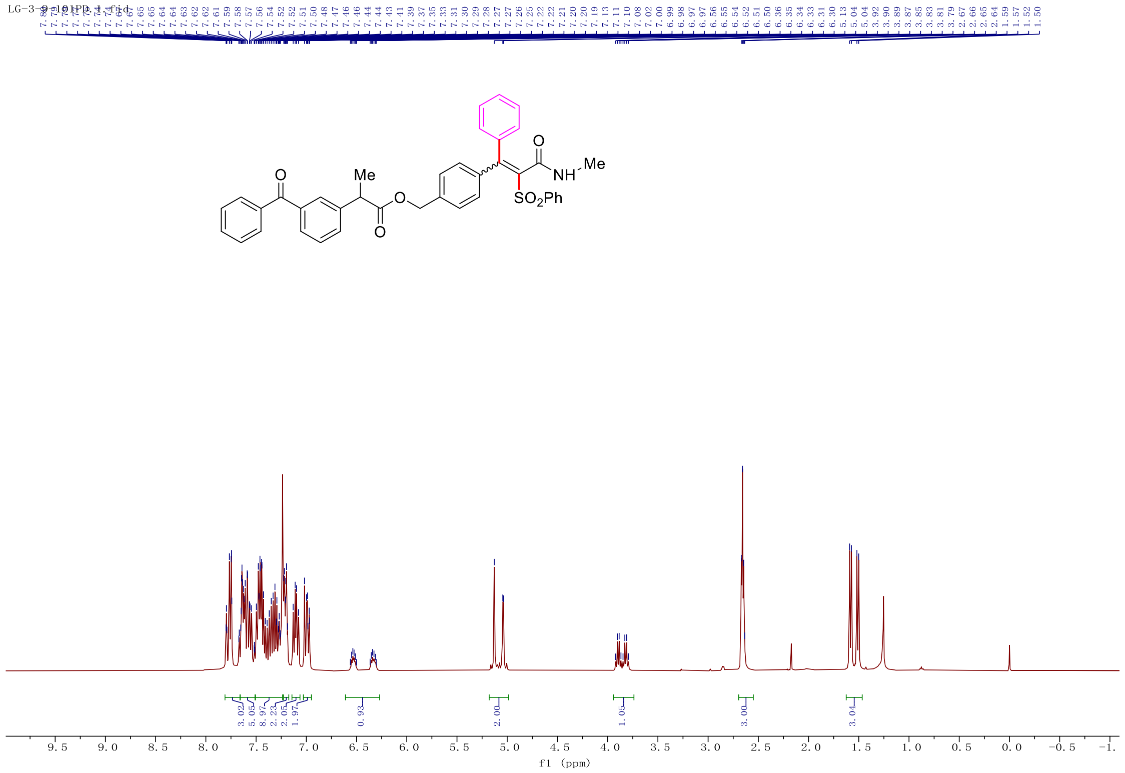

^{13}C {^{1}H} NMR Spectrum of 4-(3-(methylamino)-3-oxo-1-phenyl-2-(phenylsulfonyl)prop-1-en-1-yl)benzyl 2-(3-benzoylphenyl)propanoate (26, $CDCl_3$ as solvent, 101 MHz)

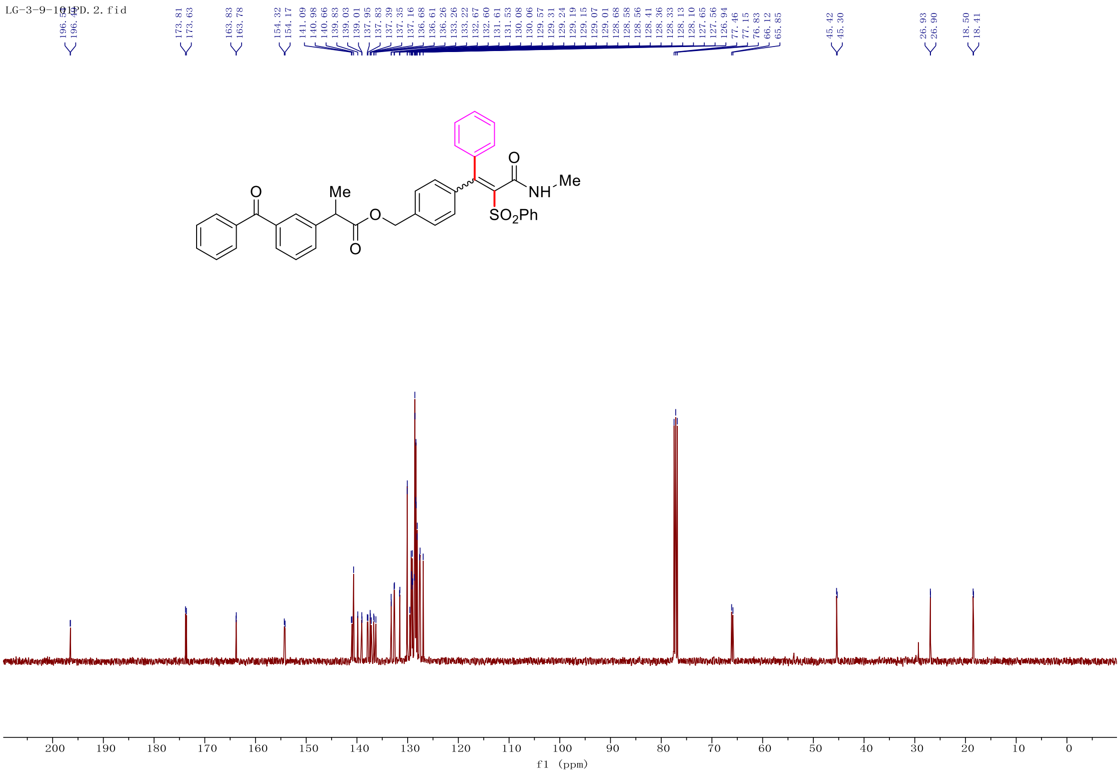

^{1}H NMR Spectrum of *N*-methyl-3-phenyl-2-(phenylsulfonyl)-3-(p-tolyl)acrylamide (27, $CDCl_3$ as solvent, 400 MHz)

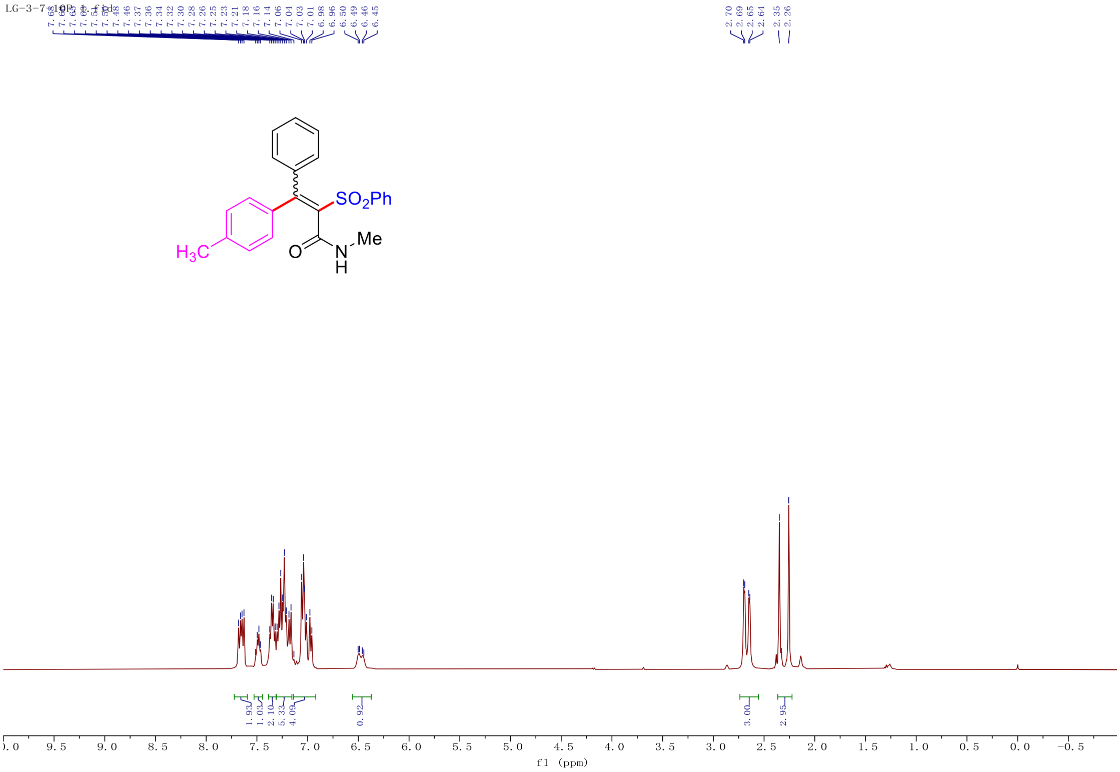

^{13}C {^{1}H} NMR Spectrum of *N*-methyl-3-phenyl-2-(phenylsulfonyl)-3-(p-tolyl)acrylamide (27, $CDCl_3$ as solvent, 101 MHz)

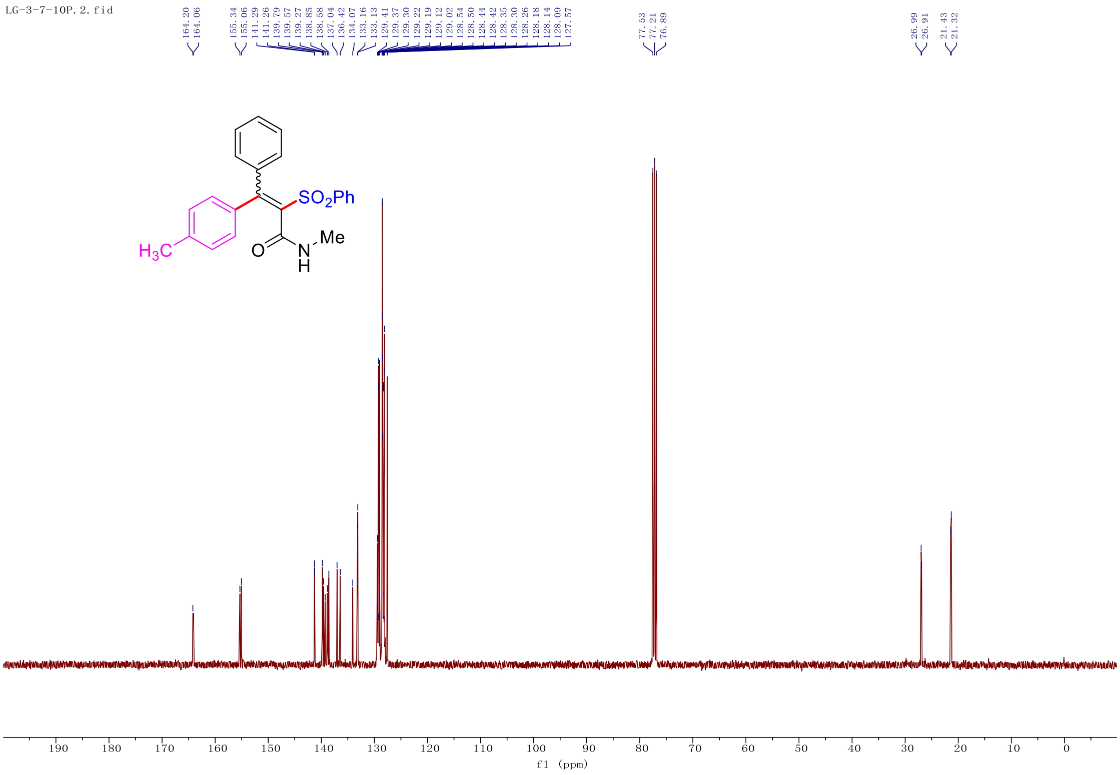

^{1}H NMR Spectrum of 3-([1,1'-biphenyl]-4-yl)-*N*-methyl-3-phenyl-2-(phenylsulfonyl)acrylamide (28, $CDCl_3$ as solvent, 400 MHz)

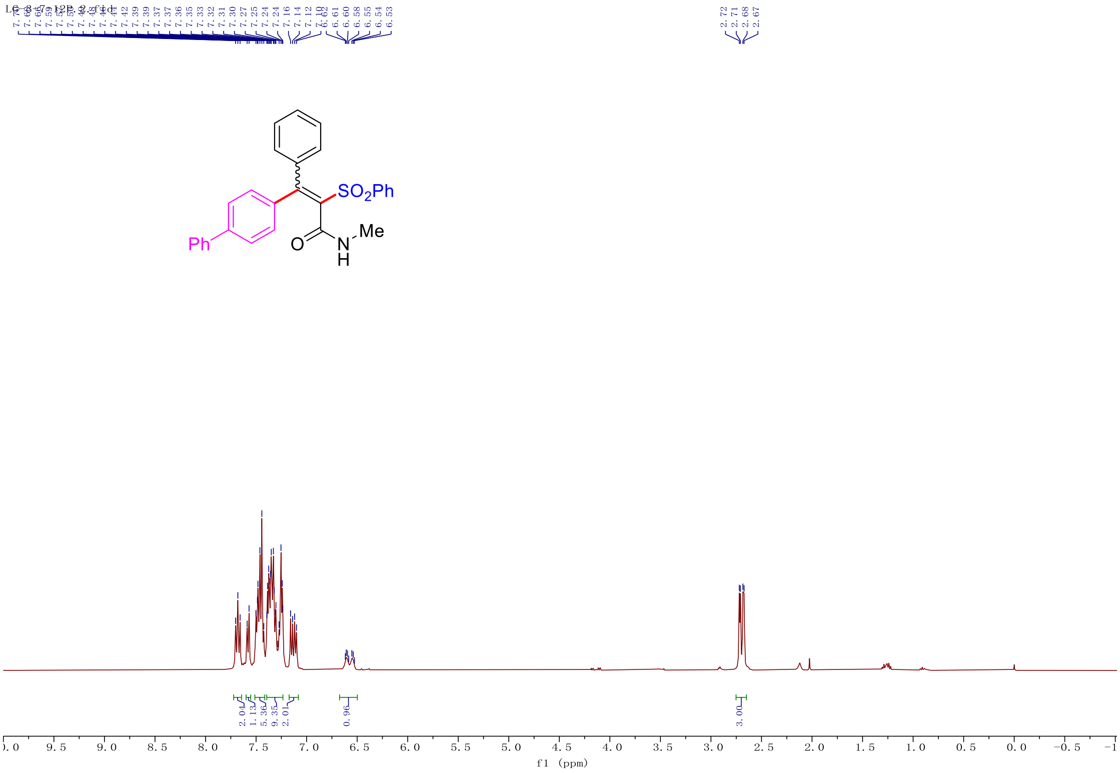

^{13}C {^{1}H} NMR Spectrum of 3-([1,1'-biphenyl]-4-yl)-*N*-methyl-3-phenyl-2-(phenylsulfonyl)acrylamide (28, $CDCl_3$ as solvent, 101 MHz)

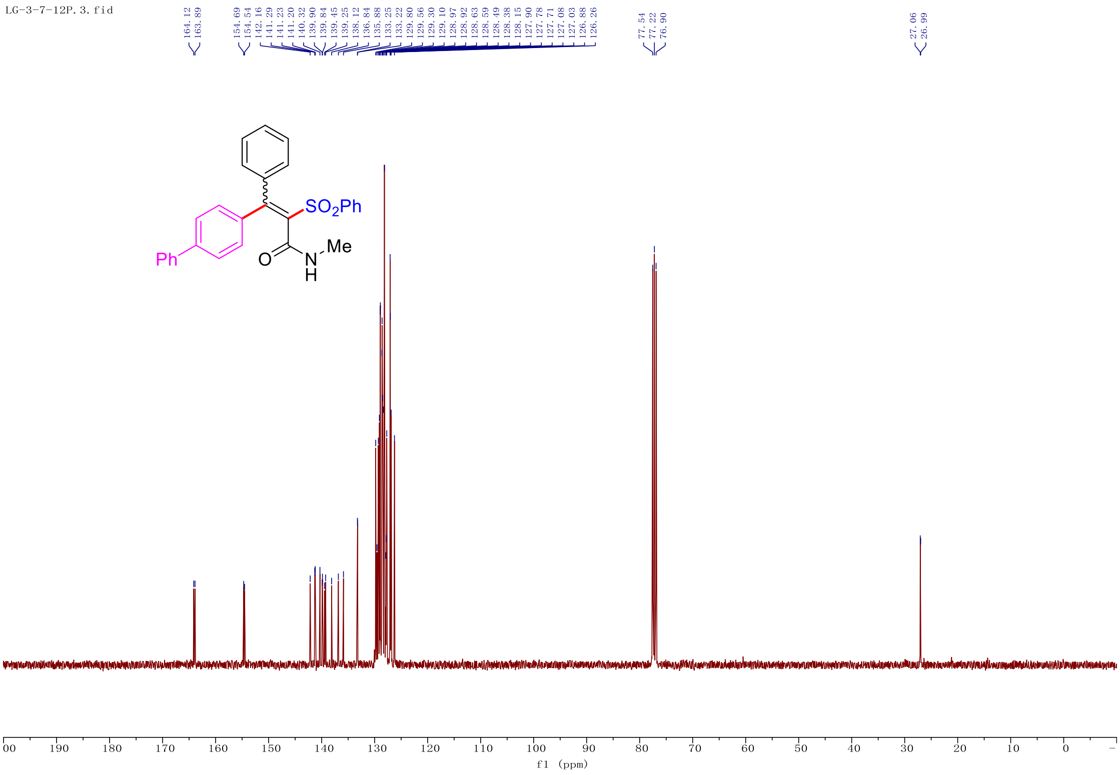

^{1}H NMR Spectrum of 3-(4-methoxyphenyl)-*N*-methyl-3-phenyl-2-(phenylsulfonyl)acrylamide (29, $CDCl_3$ as solvent, 400 MHz)

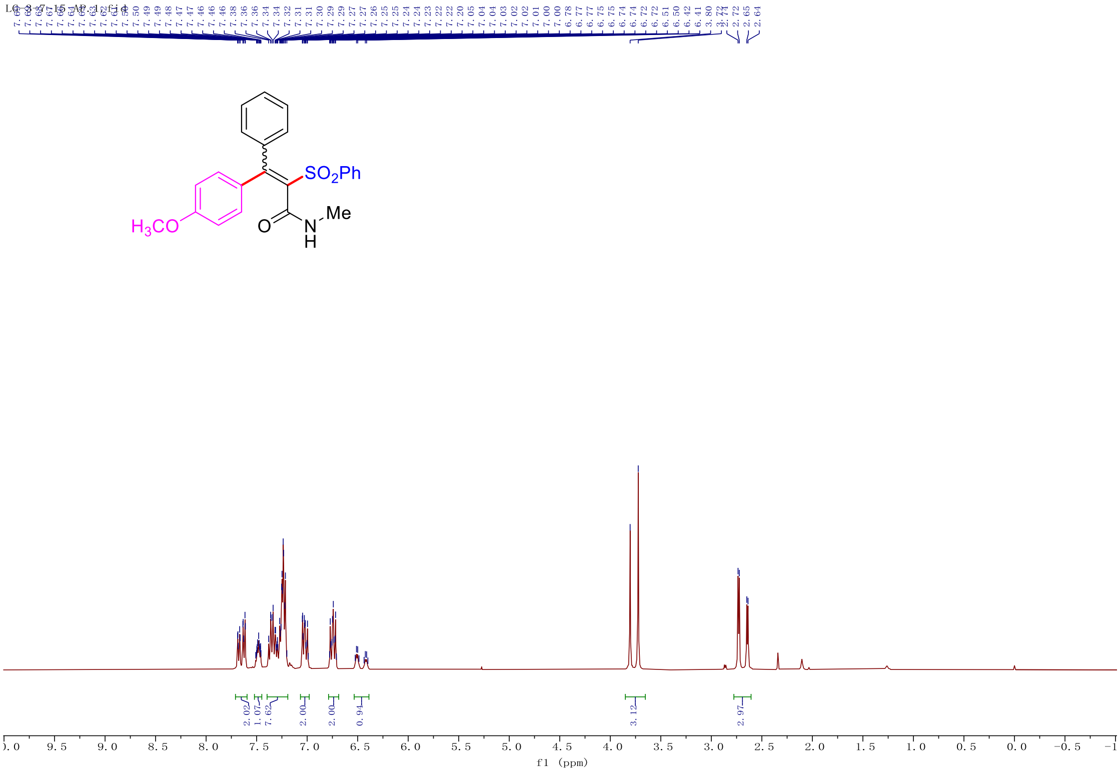

^{13}C {^{1}H} NMR Spectrum of 3-(4-methoxyphenyl)-*N*-methyl-3-phenyl-2-(phenylsulfonyl)acrylamide (29, $CDCl_3$ as solvent, 101 MHz)

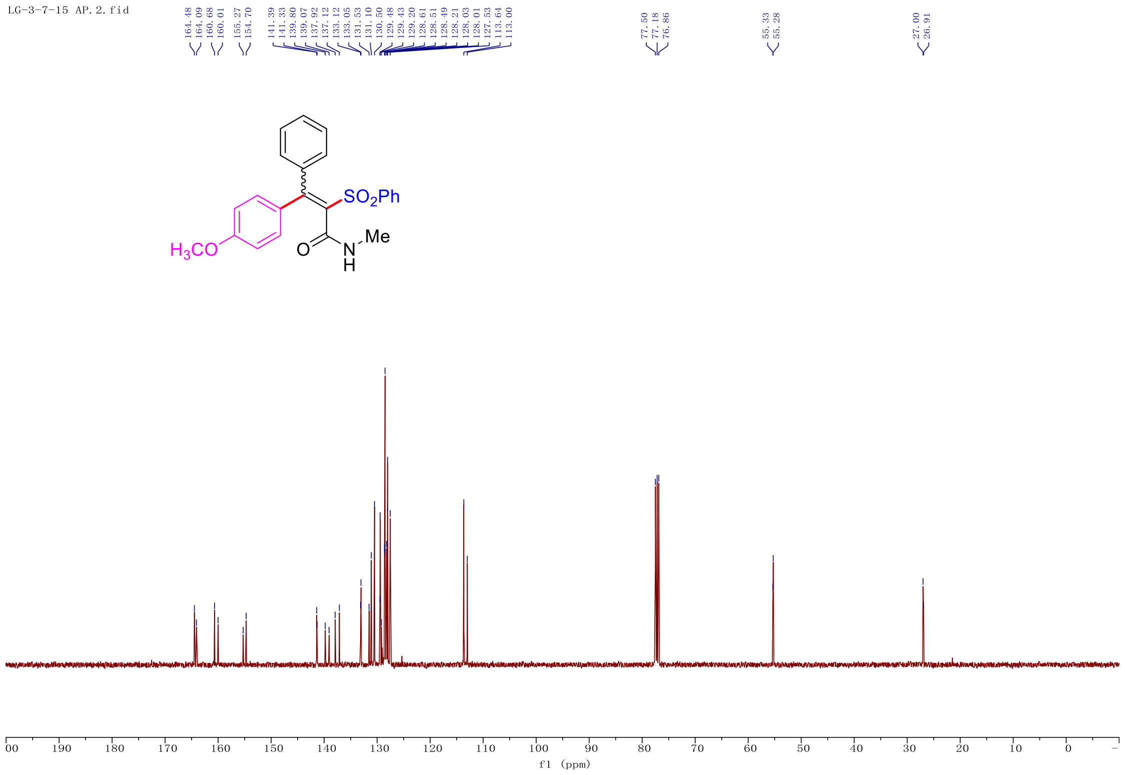

^{1}H NMR Spectrum of 3-(4-fluorophenyl)-*N*-methyl-3-phenyl-2-(phenylsulfonyl)acrylamide (30, $CDCl_3$ as solvent, 400 MHz)

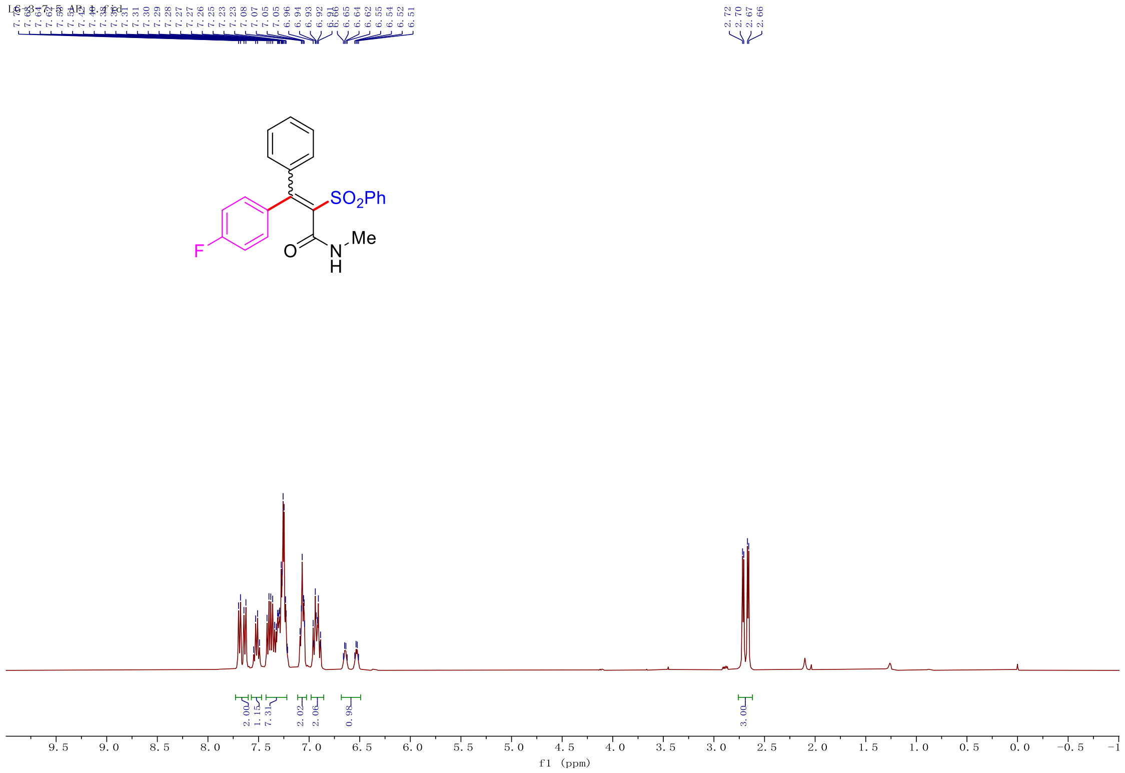

^{13}C {^{1}H} NMR Spectrum of 3-(4-fluorophenyl)-*N*-methyl-3-phenyl-2-(phenylsulfonyl)acrylamide (30, $CDCl_3$ as solvent, 101 MHz)

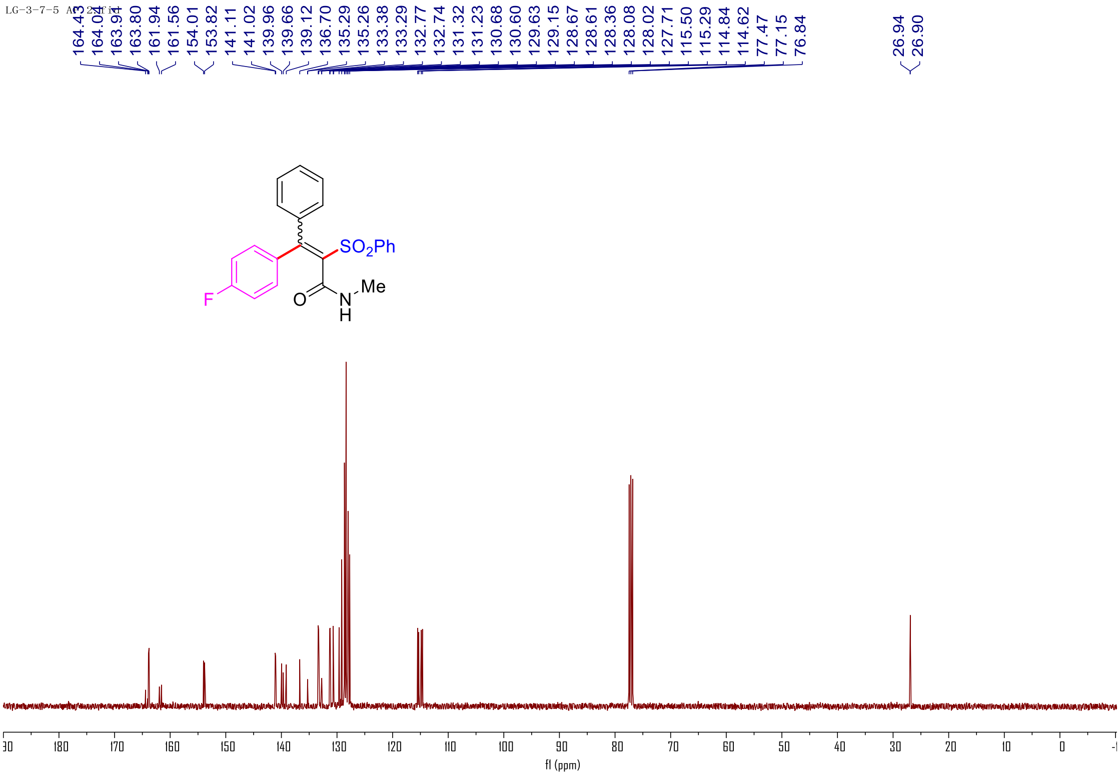

^{19}F NMR Spectrum of 3-(4-fluorophenyl)-*N*-methyl-3-phenyl-2-(phenylsulfonyl)acrylamide (30, $CDCl_3$ as solvent, 376 MHz)

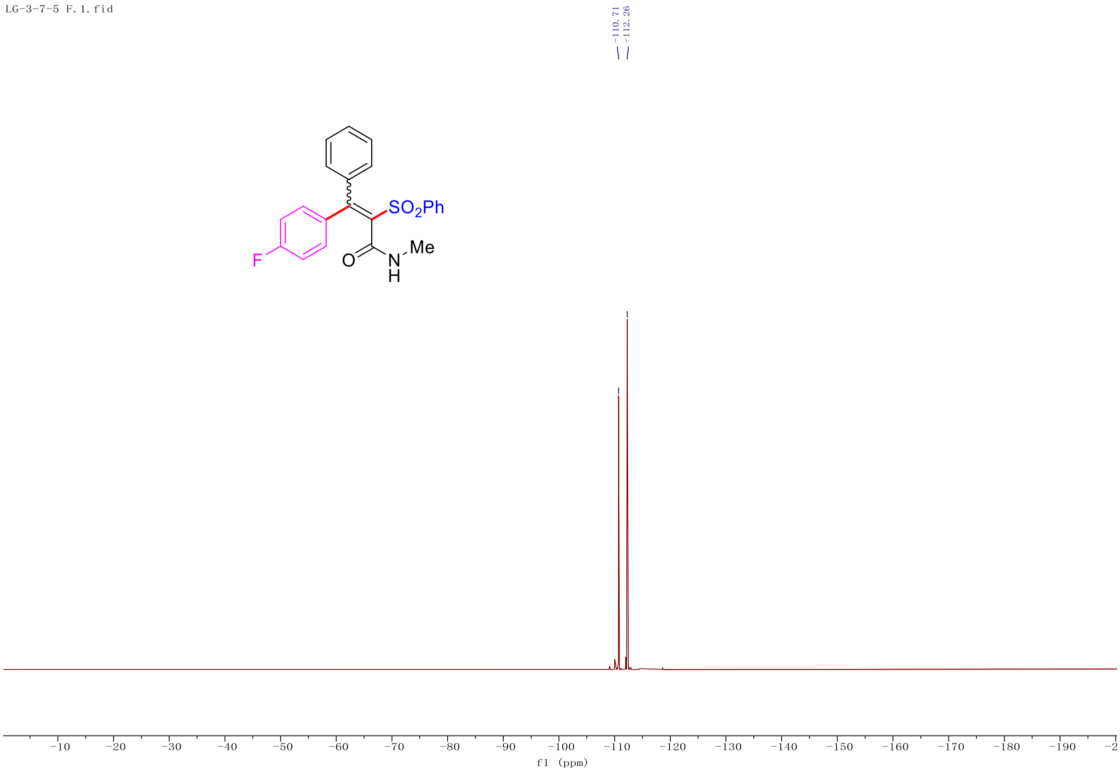

^{1}H NMR Spectrum of 3-(4-chlorophenyl)-*N*-methyl-3-phenyl-2-(phenylsulfonyl)acrylamide (31, $CDCl_3$ as solvent, 400 MHz)

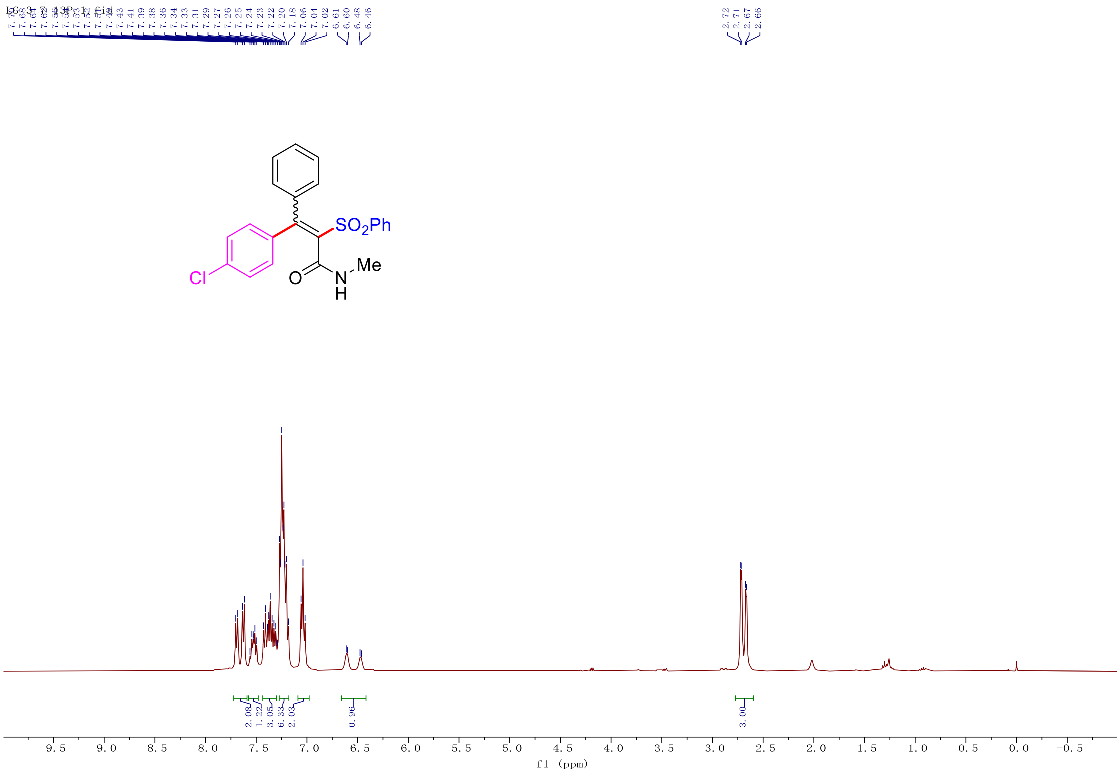

^{13}C {^{1}H} NMR Spectrum of 3-(4-chlorophenyl)-*N*-methyl-3-phenyl-2-(phenylsulfonyl)acrylamide (31, $CDCl_3$ as solvent, 101 MHz)

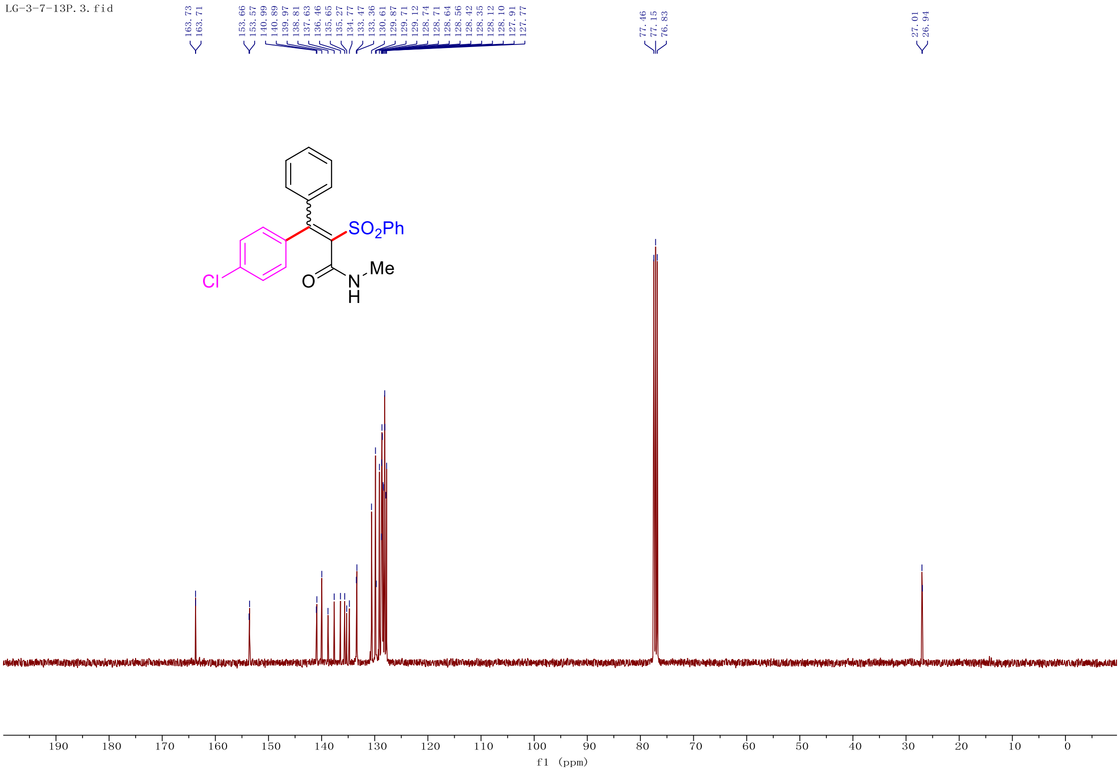

^{1}H NMR Spectrum of (*Z*)-3-(4-bromophenyl)-*N*-methyl-3-phenyl-2-(phenylsulfonyl)acrylamide (*Z*-32, $CDCl_3$ as solvent, 400 MHz)

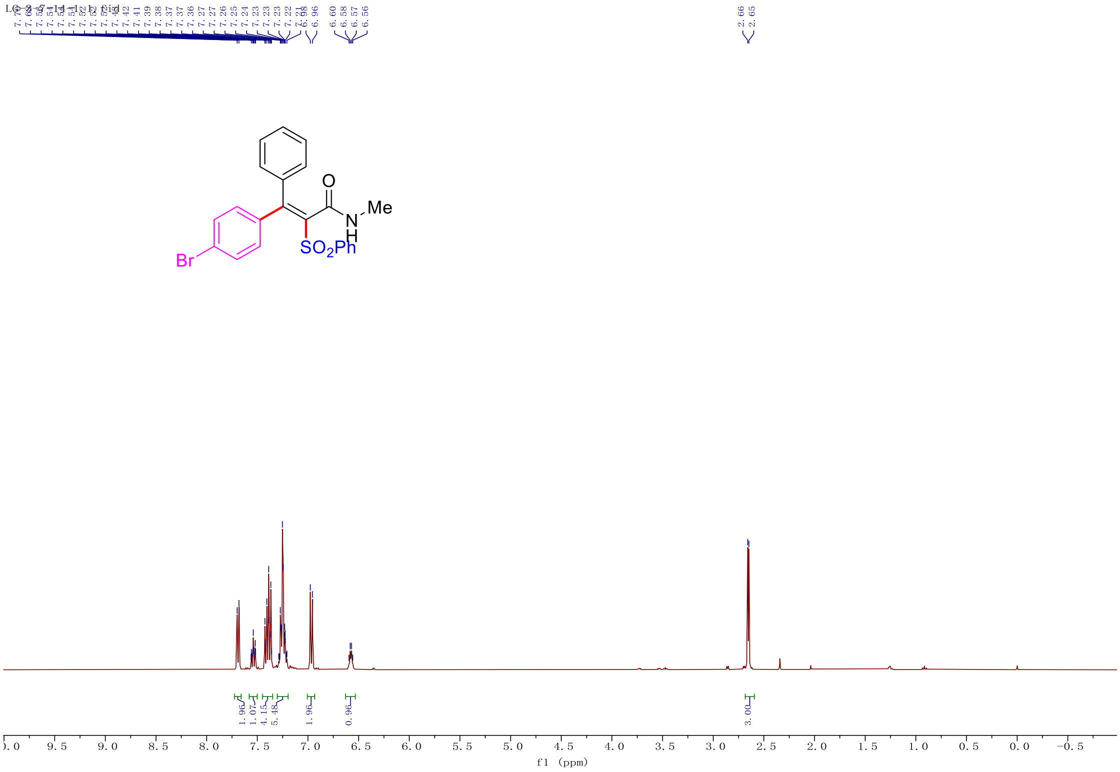

^{13}C {^{1}H} NMR Spectrum of (*Z*)-3-(4-bromophenyl)-*N*-methyl-3-phenyl-2-(phenylsulfonyl)acrylamide (*Z*-32, $CDCl_3$ as solvent, 101 MHz)

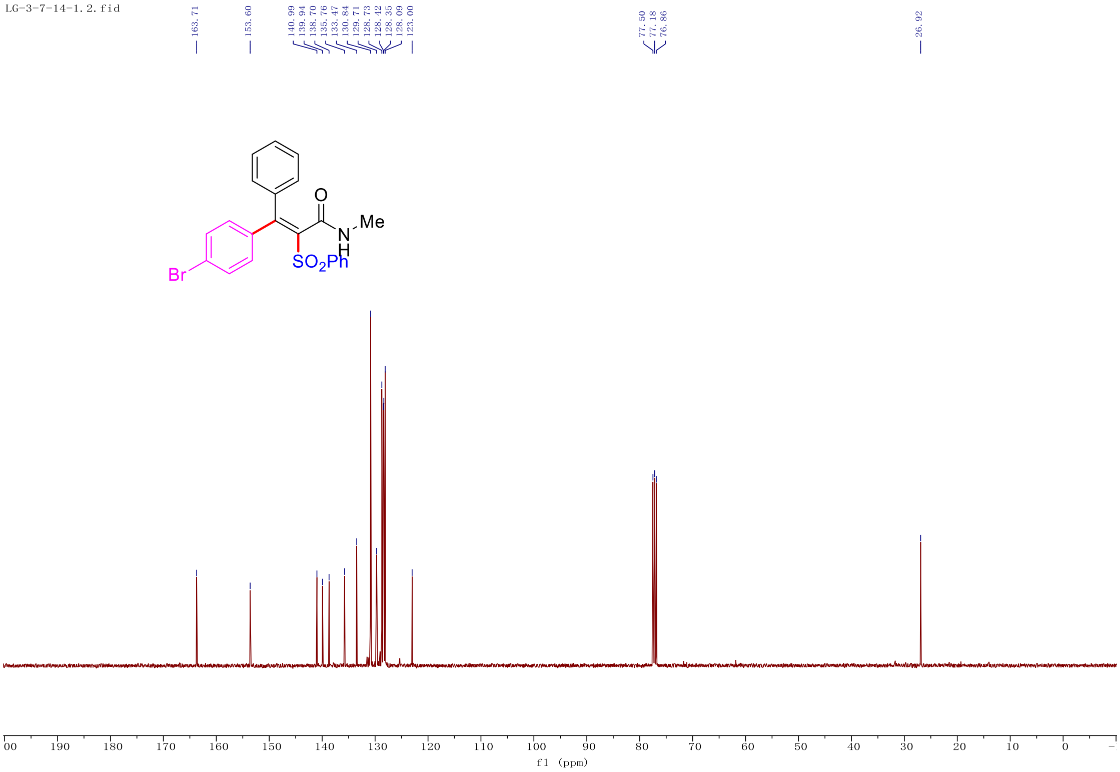

^{1}H NMR Spectrum of (*E*)-3-(4-bromophenyl)-*N*-methyl-3-phenyl-2-(phenylsulfonyl)acrylamide (*E*-32, $CDCl_3$ as solvent, 400 MHz)

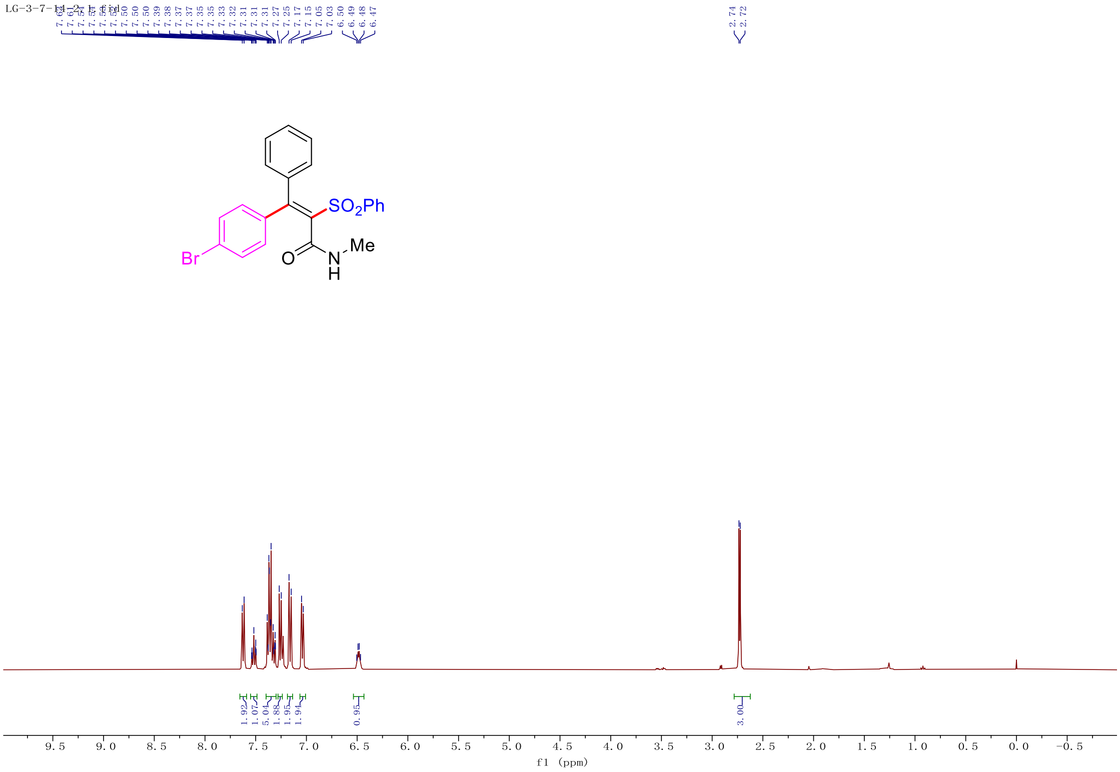

^{13}C {^{1}H} NMR Spectrum of (*E*)-3-(4-bromophenyl)-*N*-methyl-3-phenyl-2-(phenylsulfonyl)acrylamide (*E*-32, $CDCl_3$ as solvent, 101 MHz)

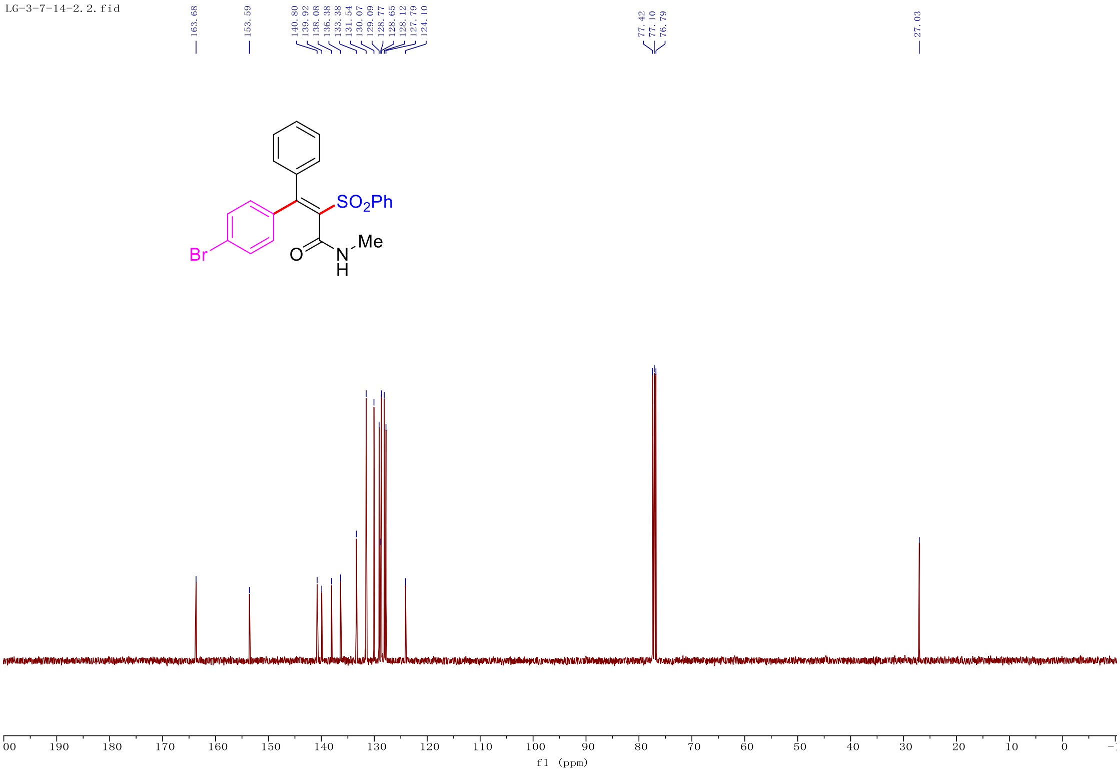

^{1}H NMR Spectrum for *Z/E* isomerization of product 32 ($CDCl_3$ as solvent, 400 MHz)

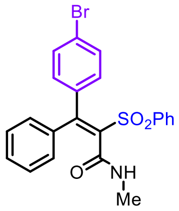

32, 97%, Z/E = 81:19

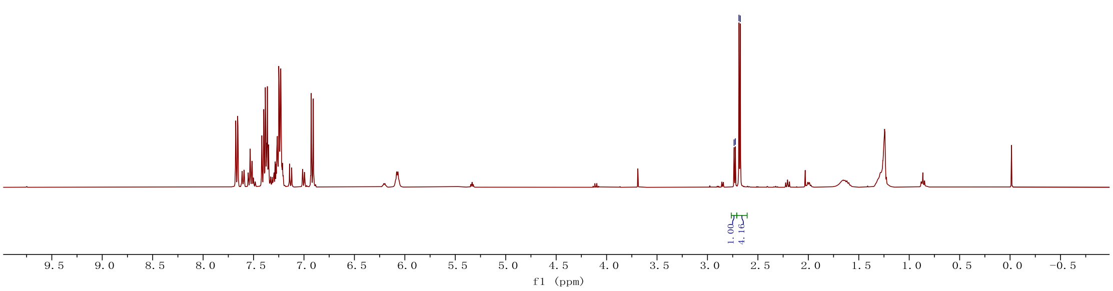

^{1}H NMR Spectrum of *N*-methyl-3-phenyl-2-(phenylsulfonyl)-3-(4-(trifluoromethyl)phenyl)acrylamide (33, $CDCl_3$ as solvent, 400 MHz)

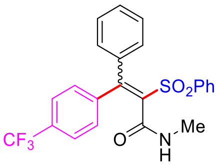

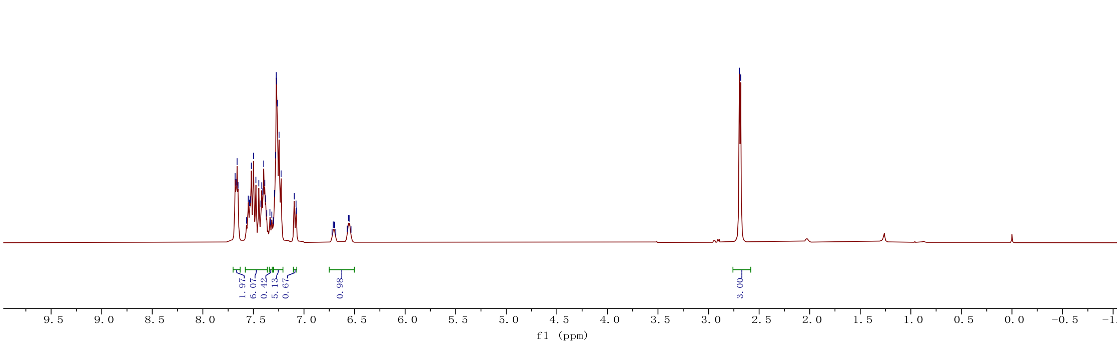

^{13}C {^{1}H} NMR Spectrum of *N*-methyl-3-phenyl-2-(phenylsulfonyl)-3-(4-(trifluoromethyl)phenyl)acrylamide (33, $CDCl_3$ as solvent, 101 MHz)

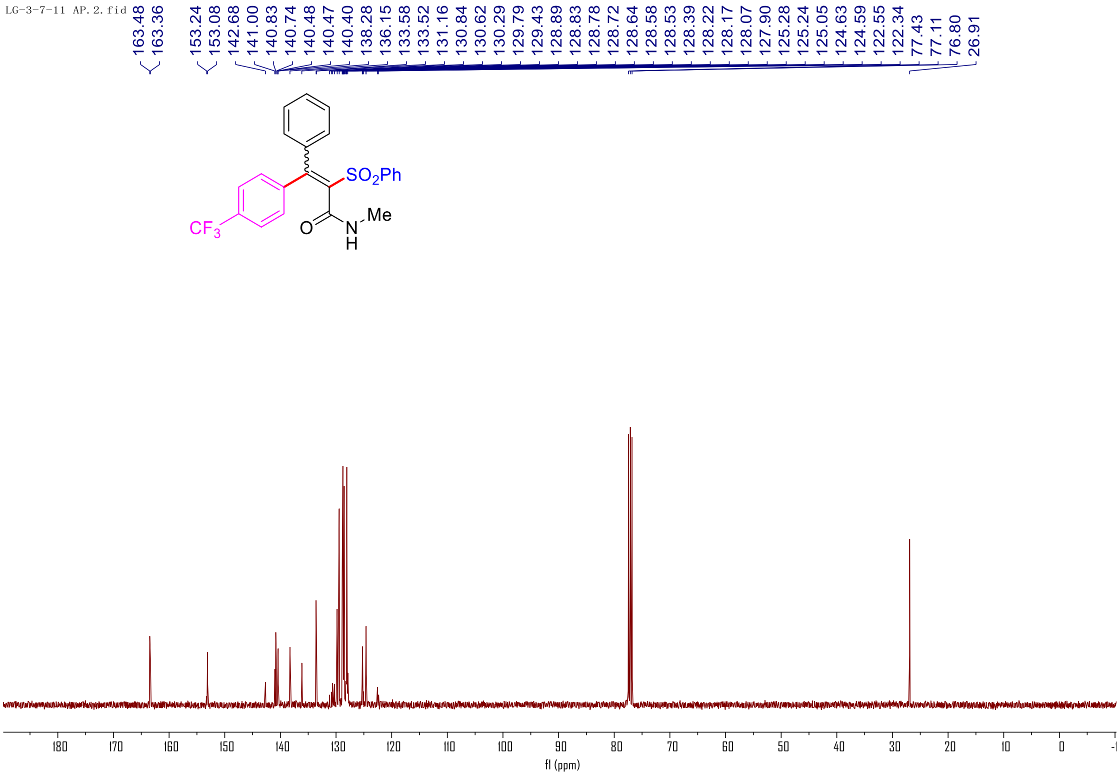

^{19}F NMR Spectrum of *N*-methyl-3-phenyl-2-(phenylsulfonyl)-3-(4-(trifluoromethyl)phenyl)acrylamide (33, $CDCl_3$ as solvent, 376 MHz)

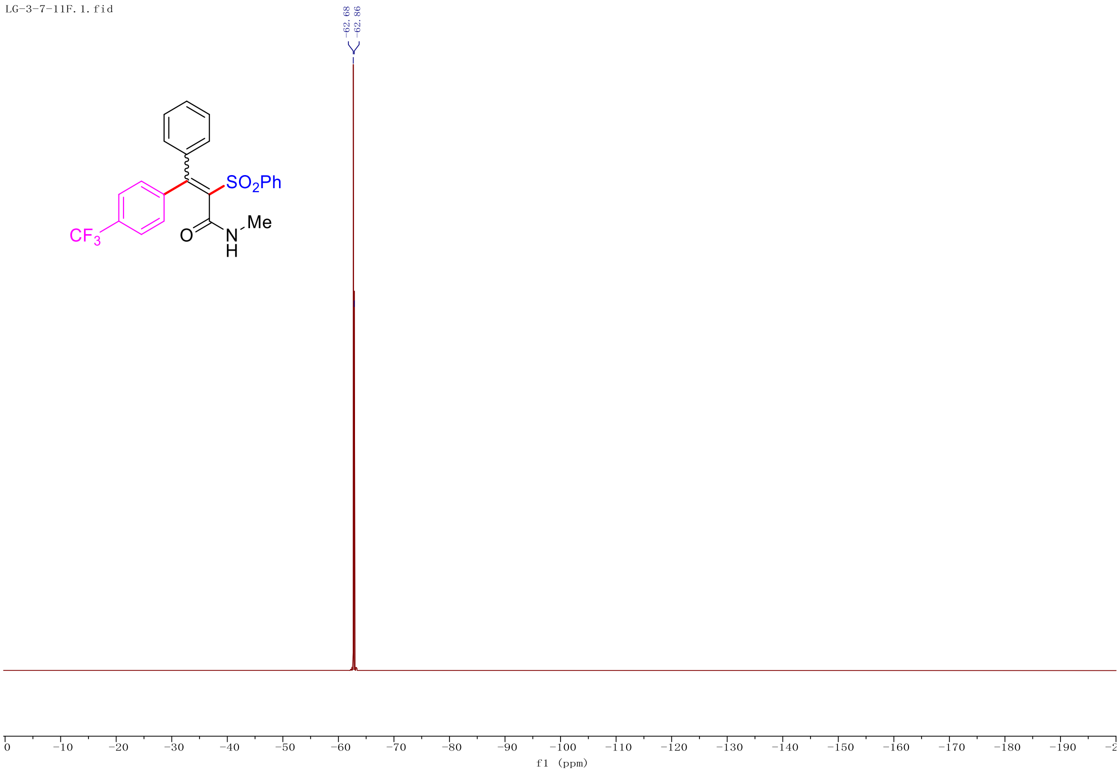

^{1}H NMR Spectrum of methyl 4-(3-(methylamino)-3-oxo-1-phenyl-2-(phenylsulfonyl)prop-1-en-1-yl)benzoate (34, $CDCl_3$ as solvent, 400 MHz)

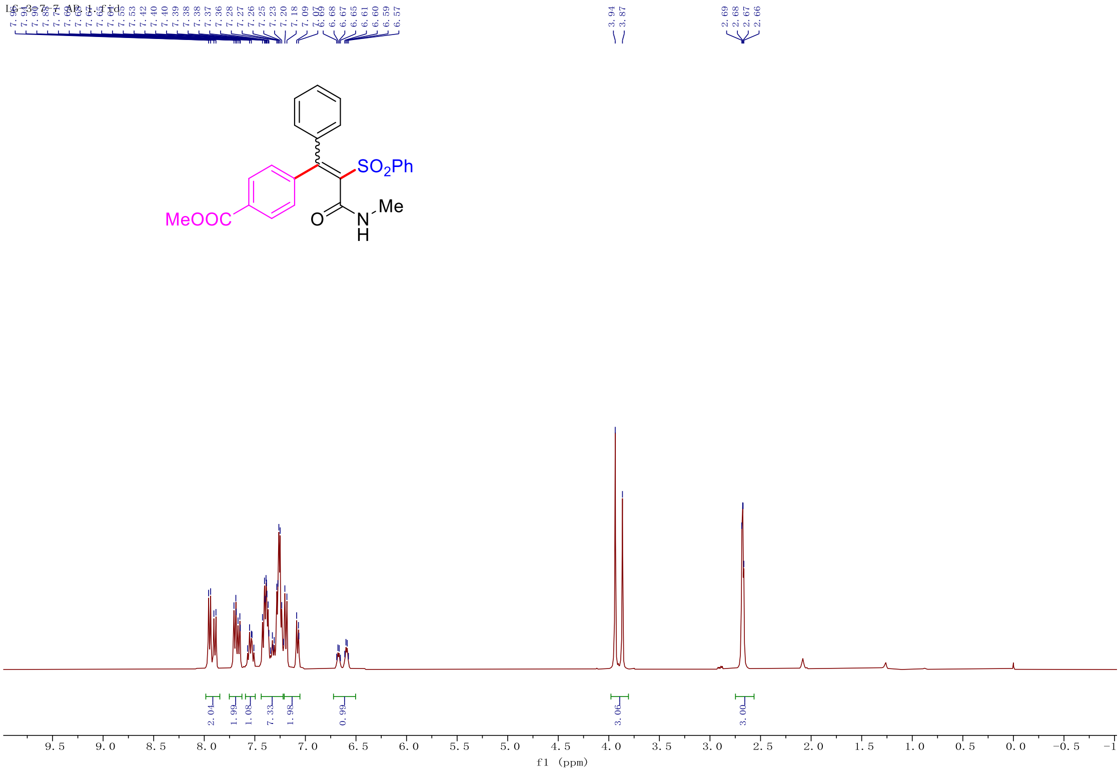

^{13}C {^{1}H} NMR Spectrum of methyl 4-(3-(methylamino)-3-oxo-1-phenyl-2-(phenylsulfonyl)prop-1-en-1-yl)benzoate (34, $CDCl_3$ as solvent, 101 MHz)

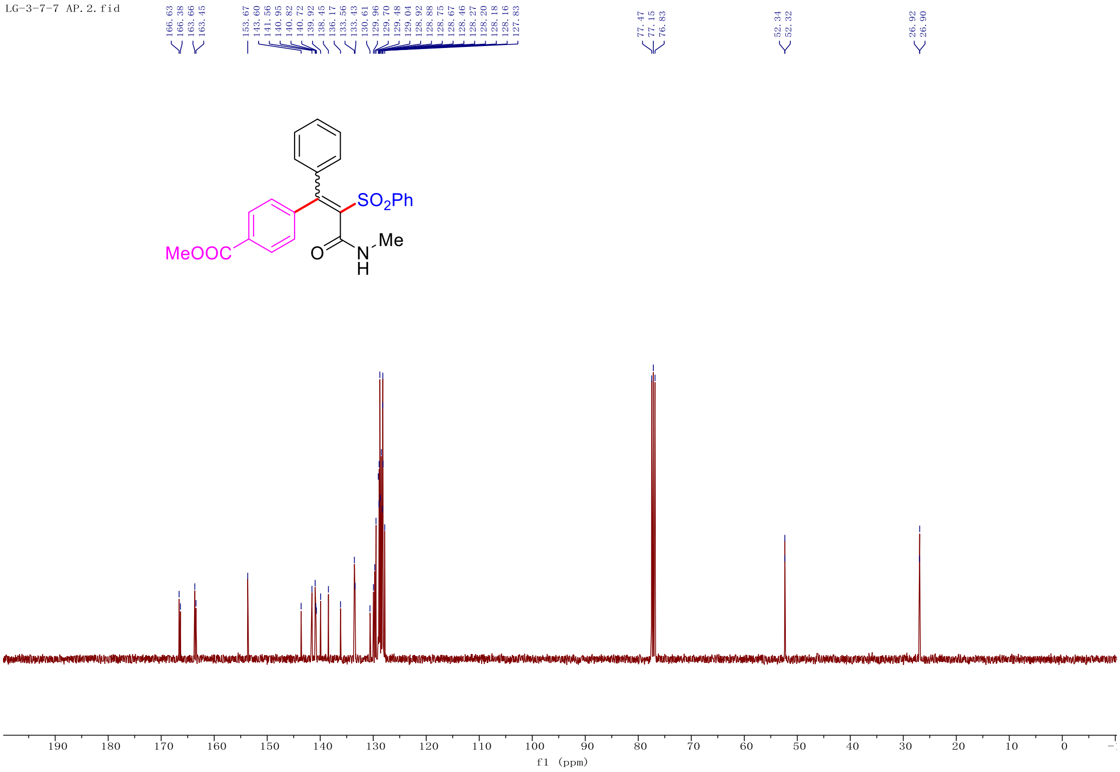

^{1}H NMR Spectrum of 3-([1,1'-biphenyl]-2-yl)-*N*-methyl-3-phenyl-2-(phenylsulfonyl)acrylamide (35, $CDCl_3$ as solvent, 400 MHz)

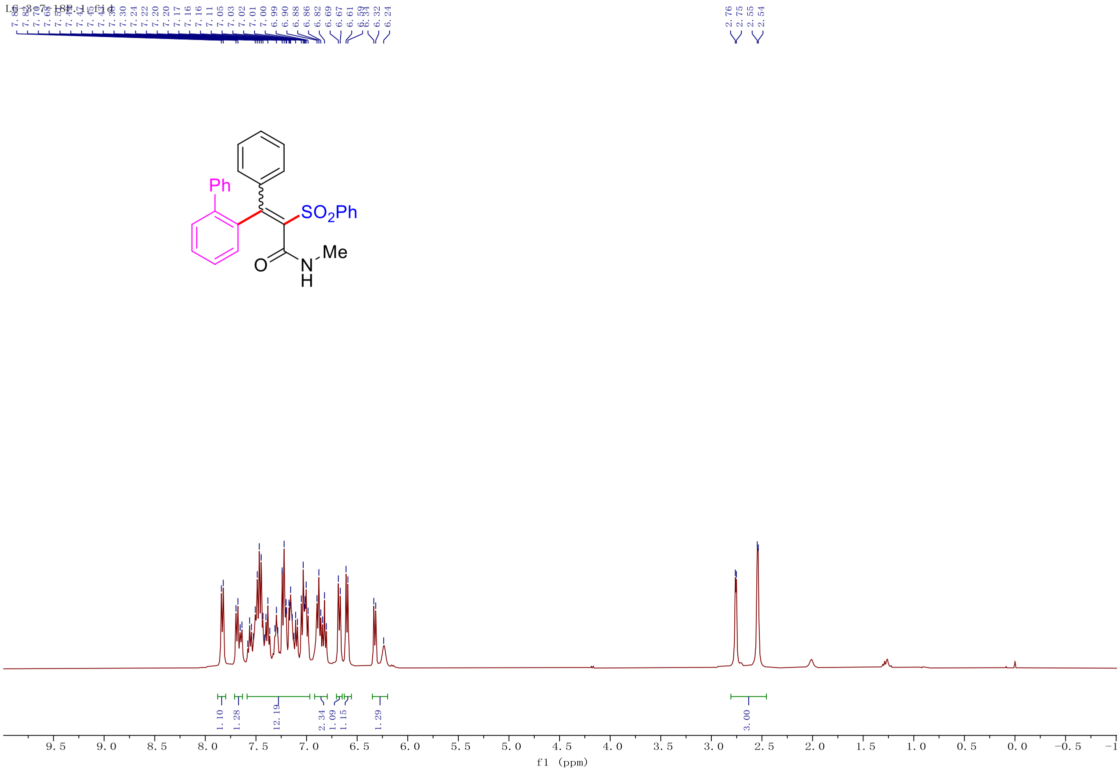

^{13}C {^{1}H} NMR Spectrum of 3-([1,1'-biphenyl]-2-yl)-*N*-methyl-3-phenyl-2-(phenylsulfonyl)acrylamide (35, $CDCl_3$ as solvent, 101 MHz)

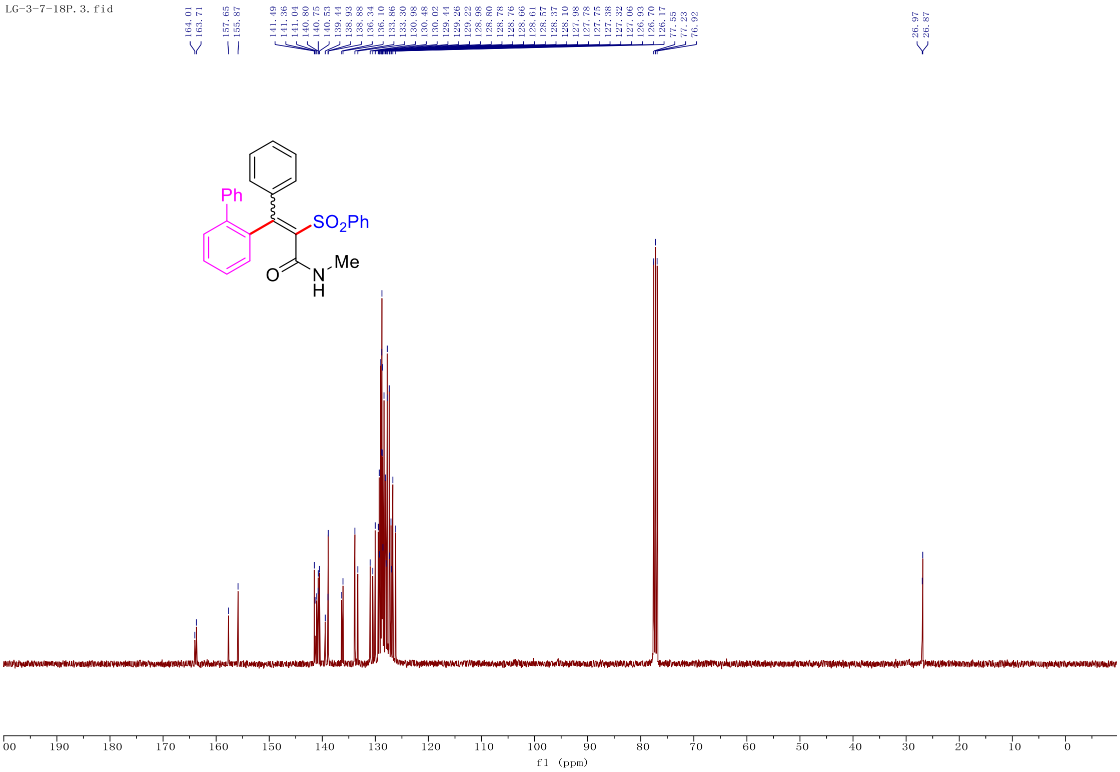

^{1}H NMR Spectrum of 3-(2-chlorophenyl)-*N*-methyl-3-phenyl-2-(phenylsulfonyl)acrylamide (36, $CDCl_3$ as solvent, 400 MHz)

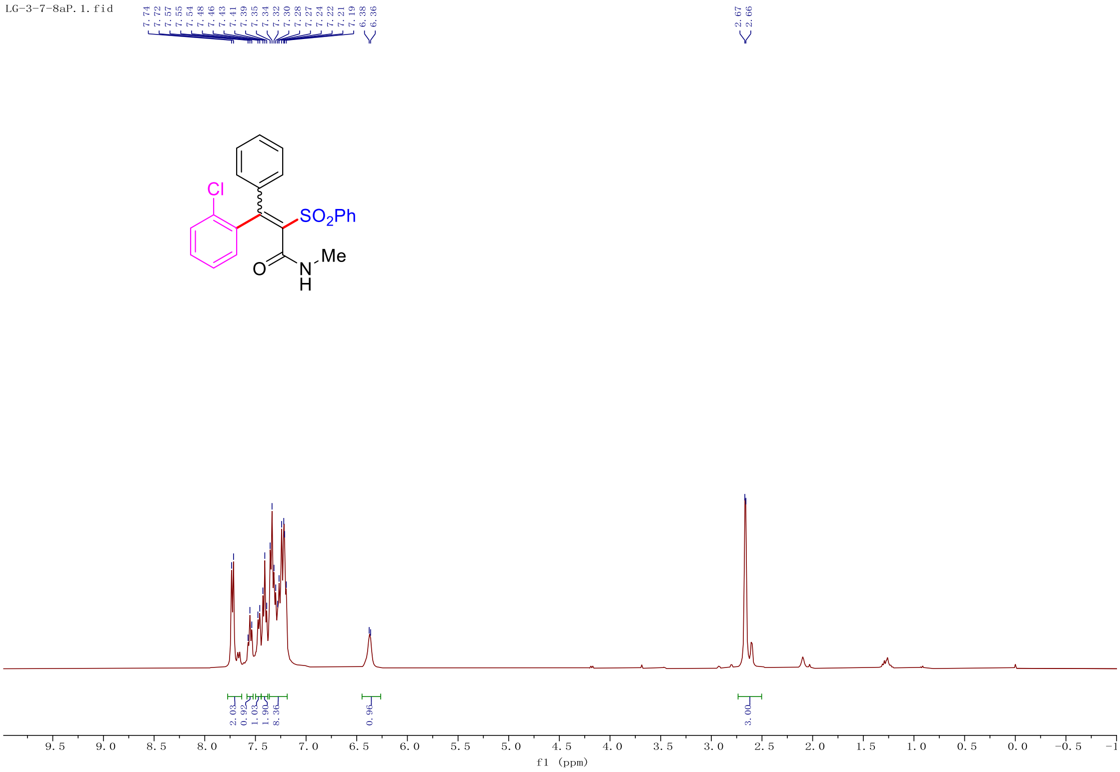

^{13}C {^{1}H} NMR Spectrum of 3-(2-chlorophenyl)-*N*-methyl-3-phenyl-2-(phenylsulfonyl)acrylamide (36, $CDCl_3$ as solvent, 101 MHz)

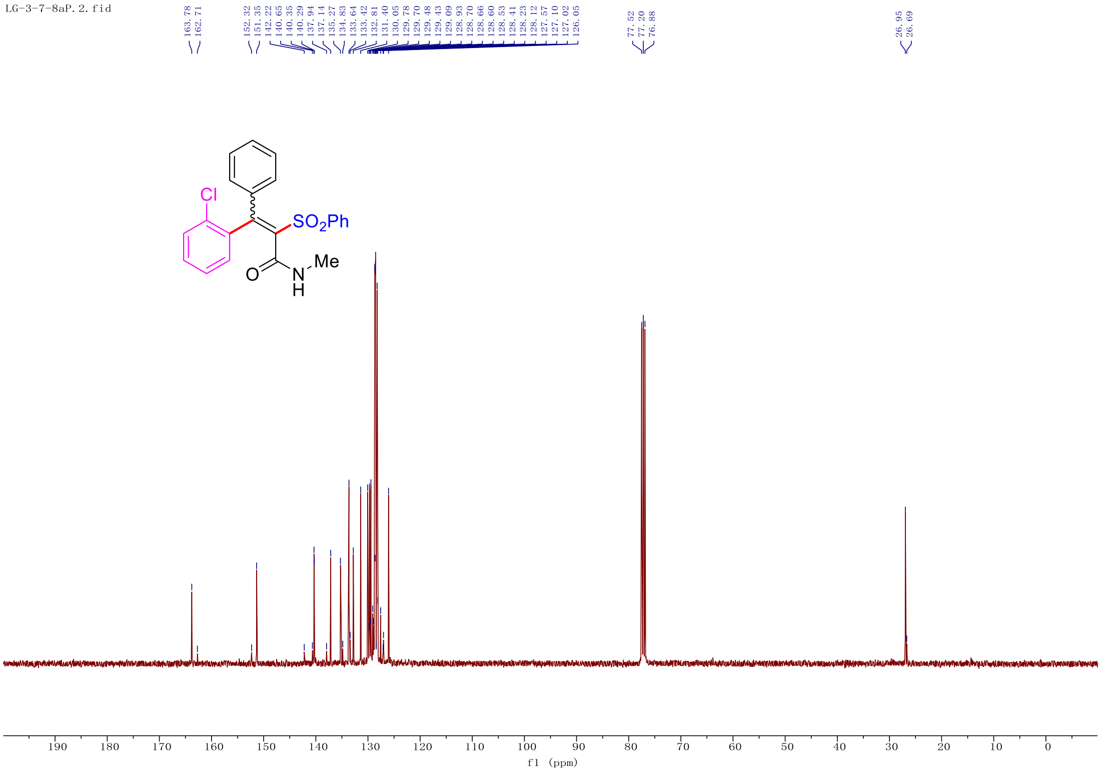

^{1}H NMR Spectrum of 3-(3-bromophenyl)-*N*-methyl-3-phenyl-2-(phenylsulfonyl)acrylamide (37, $CDCl_3$ as solvent, 400 MHz)

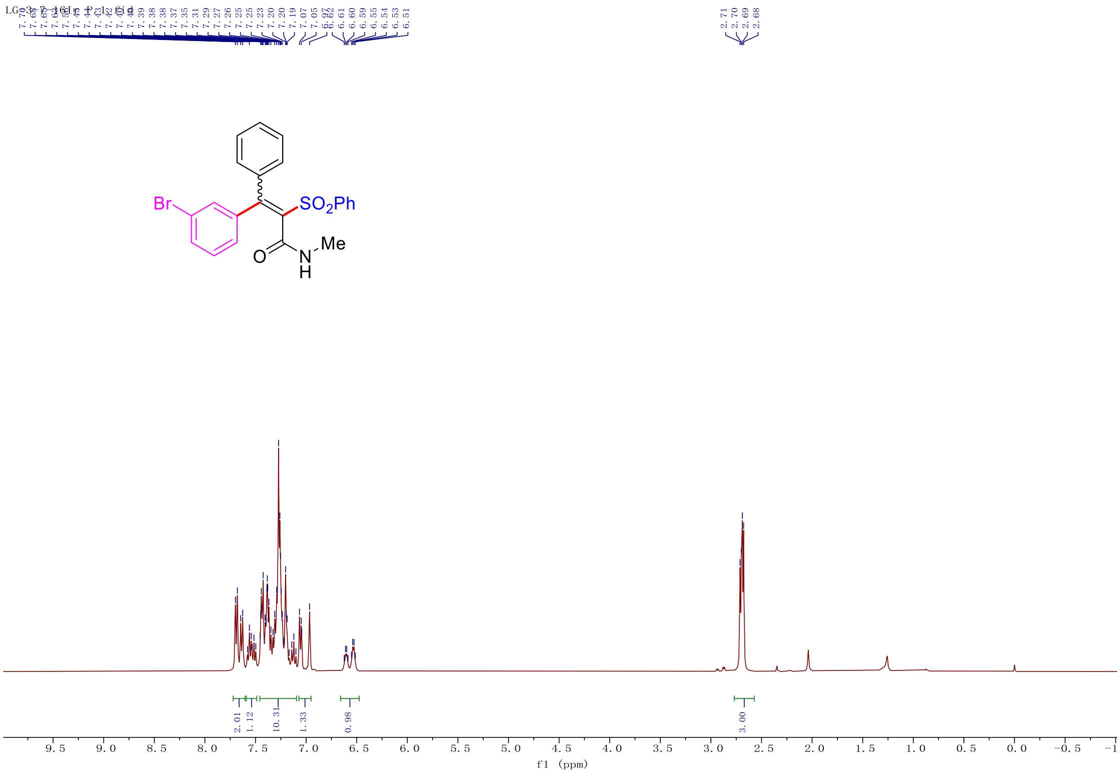

^{13}C {^{1}H} NMR Spectrum of 3-(3-bromophenyl)-*N*-methyl-3-phenyl-2-(phenylsulfonyl)acrylamide (37, $CDCl_3$ as solvent, 101 MHz)

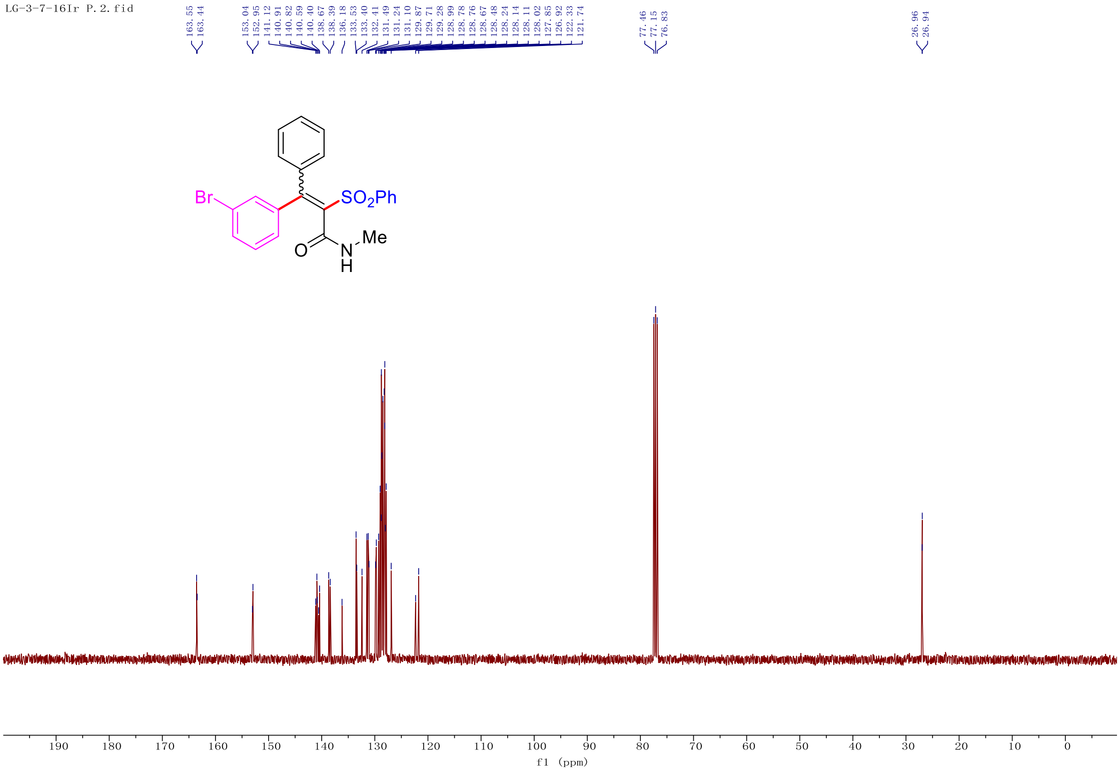

^{1}H NMR Spectrum of 3-(3-iodophenyl)-*N*-methyl-3-phenyl-2-(phenylsulfonyl)acrylamide (38, $CDCl_3$ as solvent, 400 MHz)

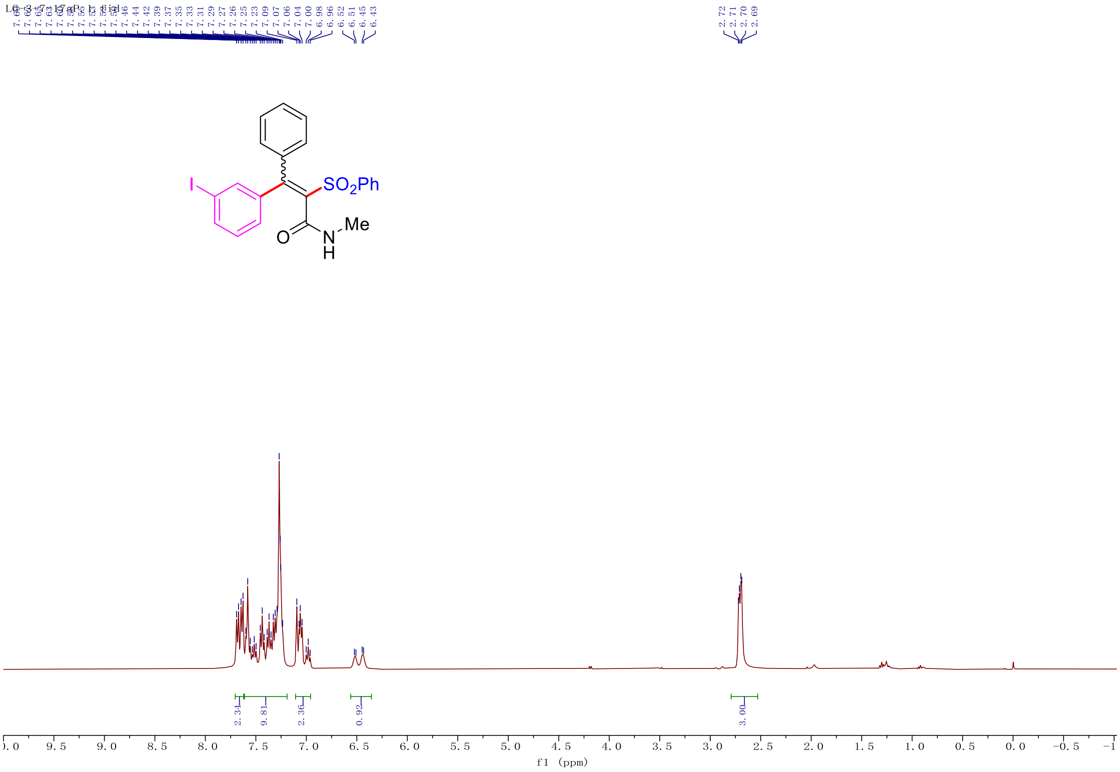

^{1}H NMR Spectrum for *Z/E* isomerization of product 38 ($CDCl_3$ as solvent, 400 MHz)

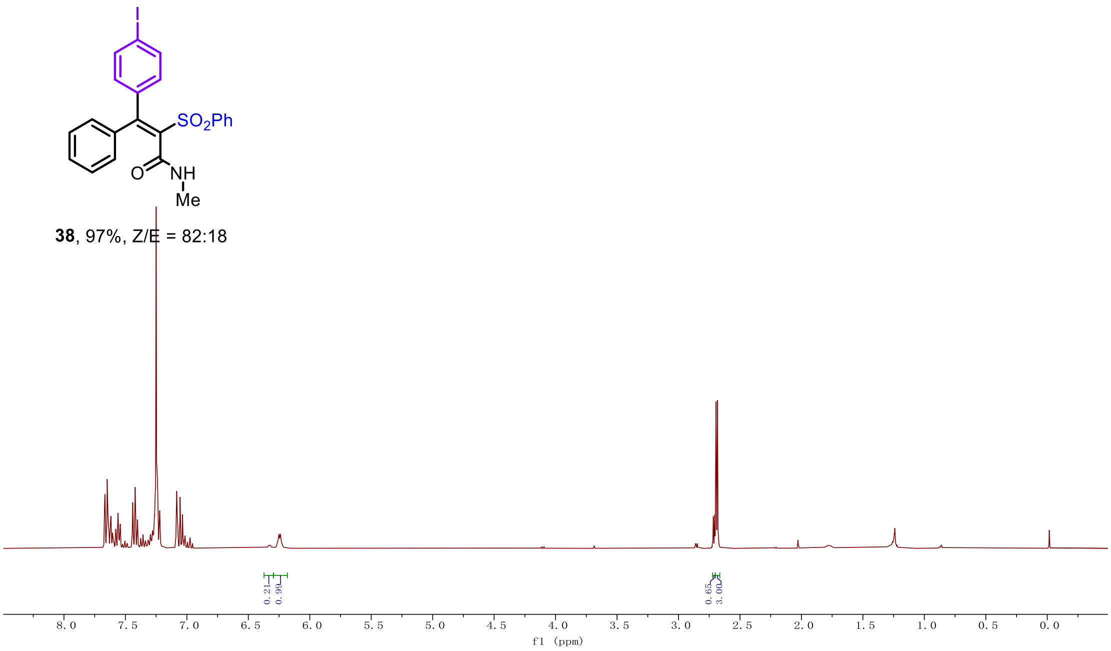

^{13}C {^{1}H} NMR Spectrum of 3-(3-iodophenyl)-*N*-methyl-3-phenyl-2-(phenylsulfonyl)acrylamide (38, $CDCl_3$ as solvent, 101 MHz)

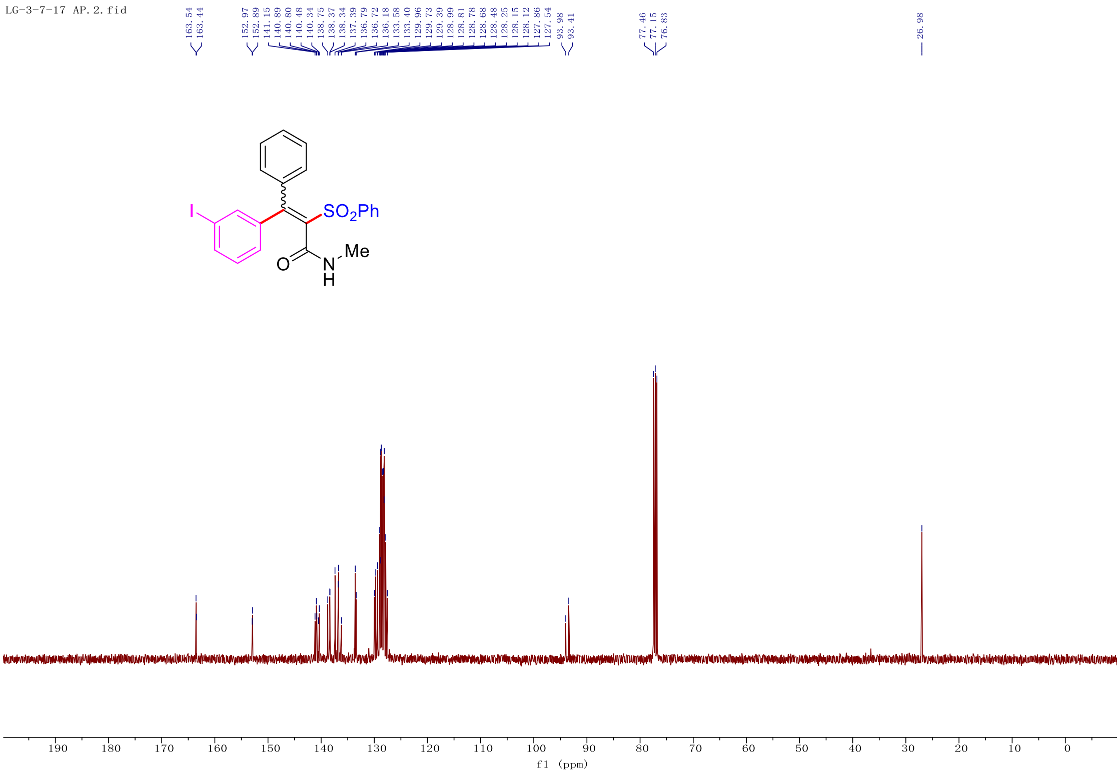

^{1}H NMR Spectrum of *N*-methyl-3-(naphthalen-1-yl)-3-phenyl-2-(phenylsulfonyl)acrylamide (39, $CDCl_3$ as solvent, 400 MHz)

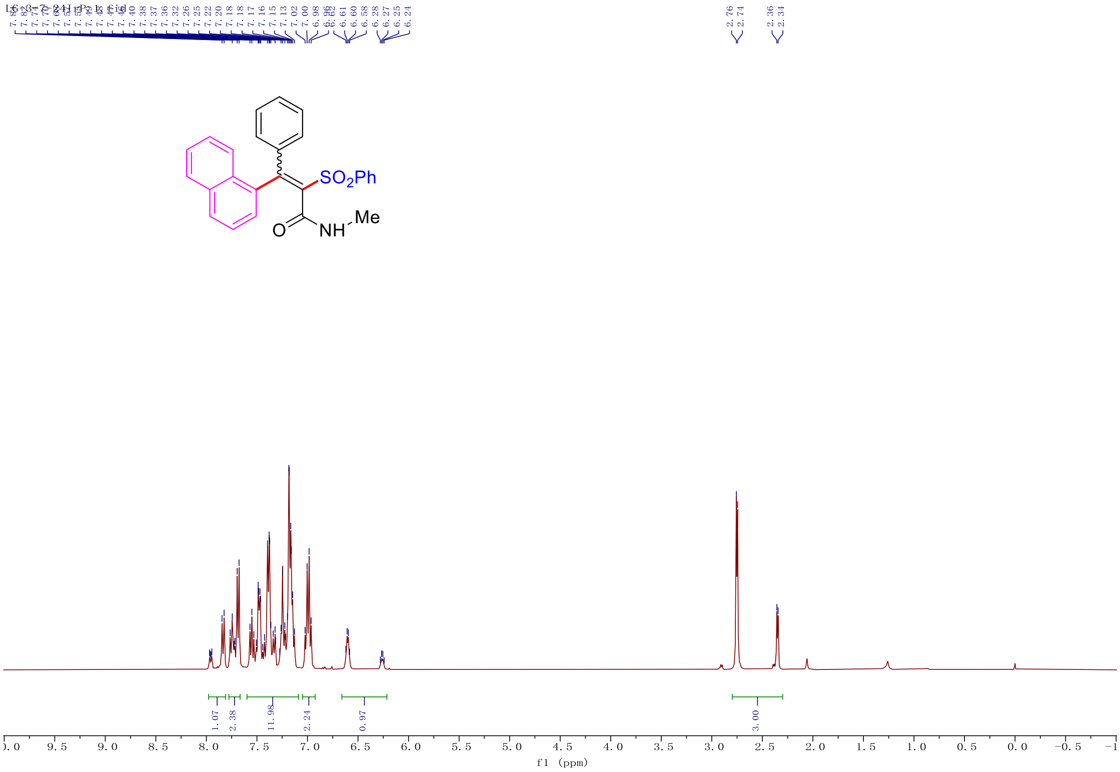

^{13}C {^{1}H} NMR Spectrum of *N*-methyl-3-(naphthalen-1-yl)-3-phenyl-2-(phenylsulfonyl)acrylamide (39, $CDCl_3$ as solvent, 101 MHz)

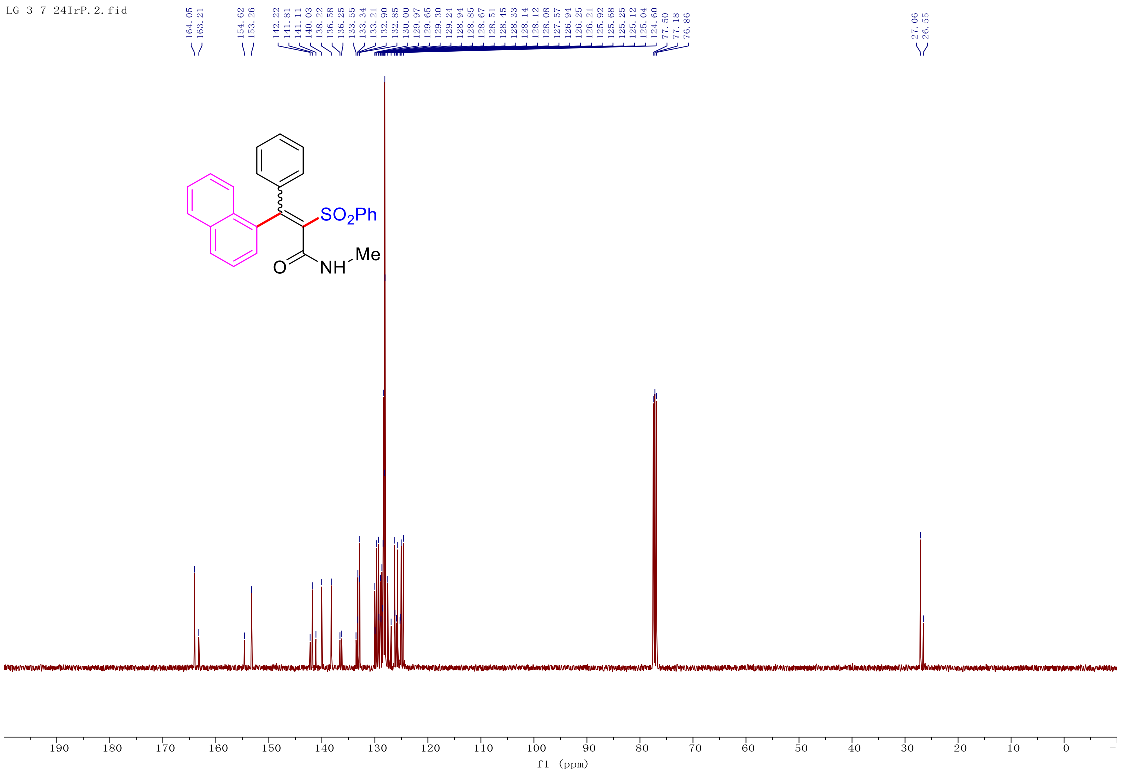

^{1}H NMR Spectrum of 3-(benzo[d][1,3]dioxol-5-yl)-*N*-methyl-3-phenyl-2-(phenylsulfonyl)acrylamide (40, $CDCl_3$ as solvent, 400 MHz)

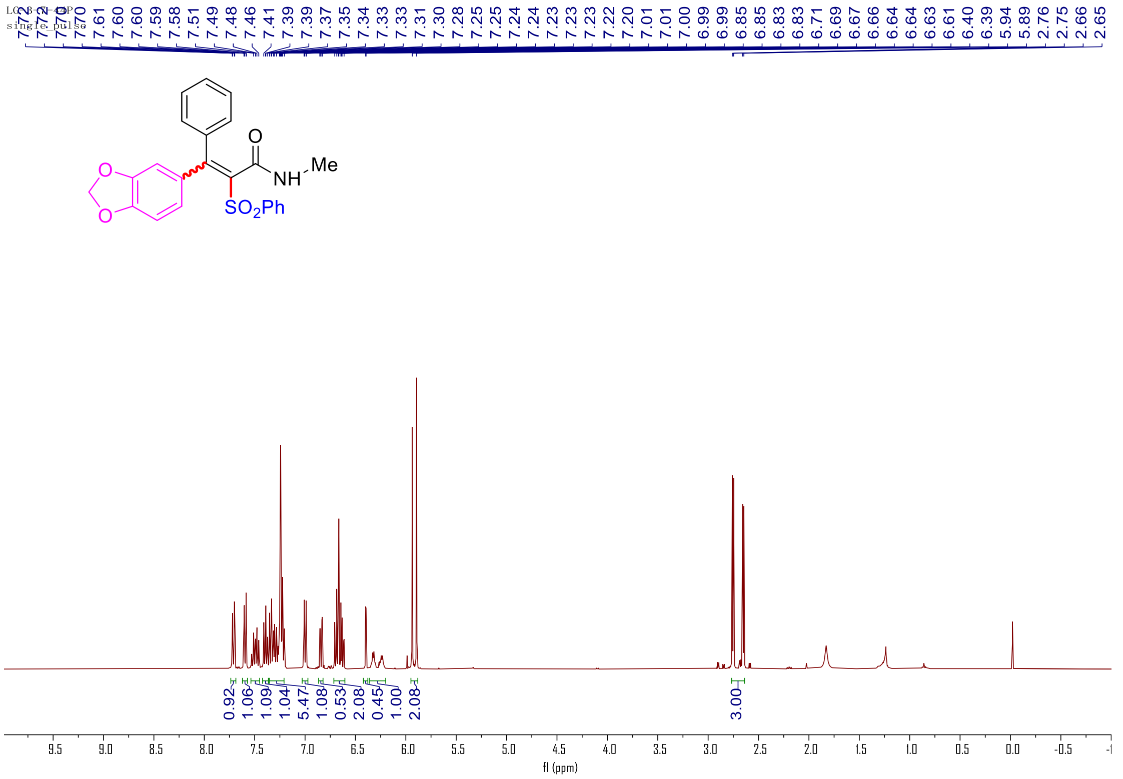

^{13}C {^{1}H} NMR Spectrum of 3-(benzo[d][1,3]dioxol-5-yl)-*N*-methyl-3-phenyl-2-(phenylsulfonyl)acrylamide (40, $CDCl_3$ as solvent, 101 MHz)

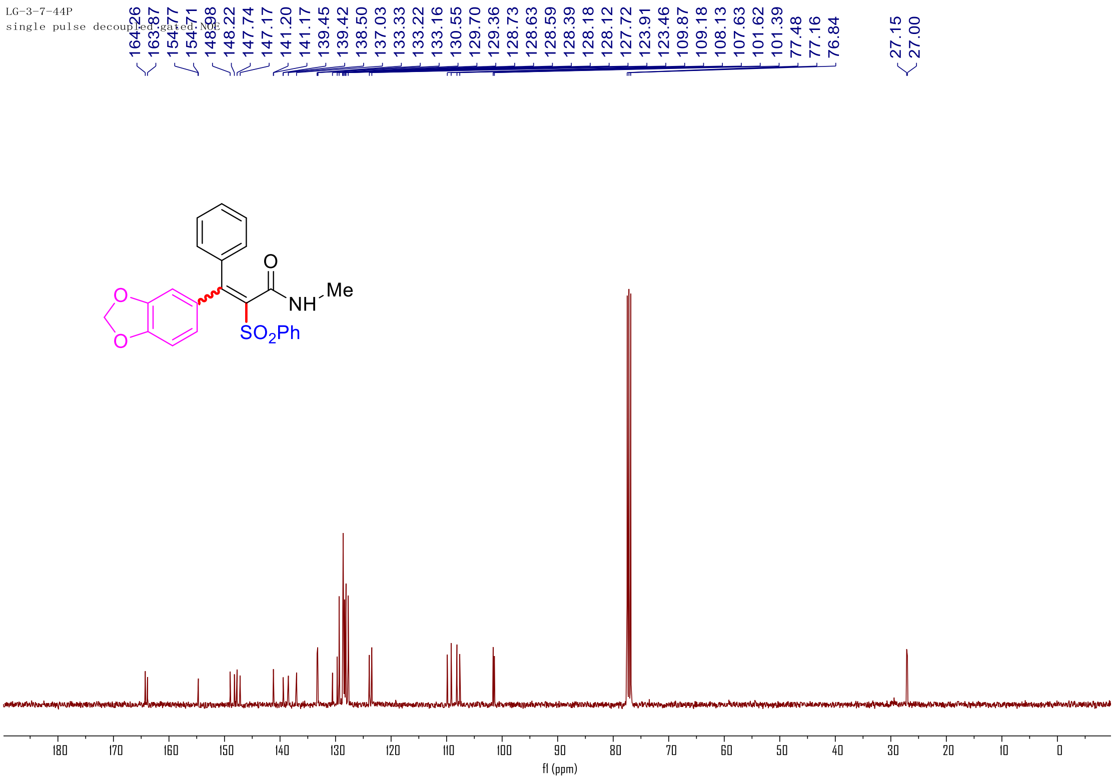

^{1}H NMR Spectrum of 3-(dibenzo[b,d]furan-3-yl)-*N*-methyl-3-phenyl-2-(phenylsulfonyl)acrylamide (41, $CDCl_3$ as solvent, 400 MHz)

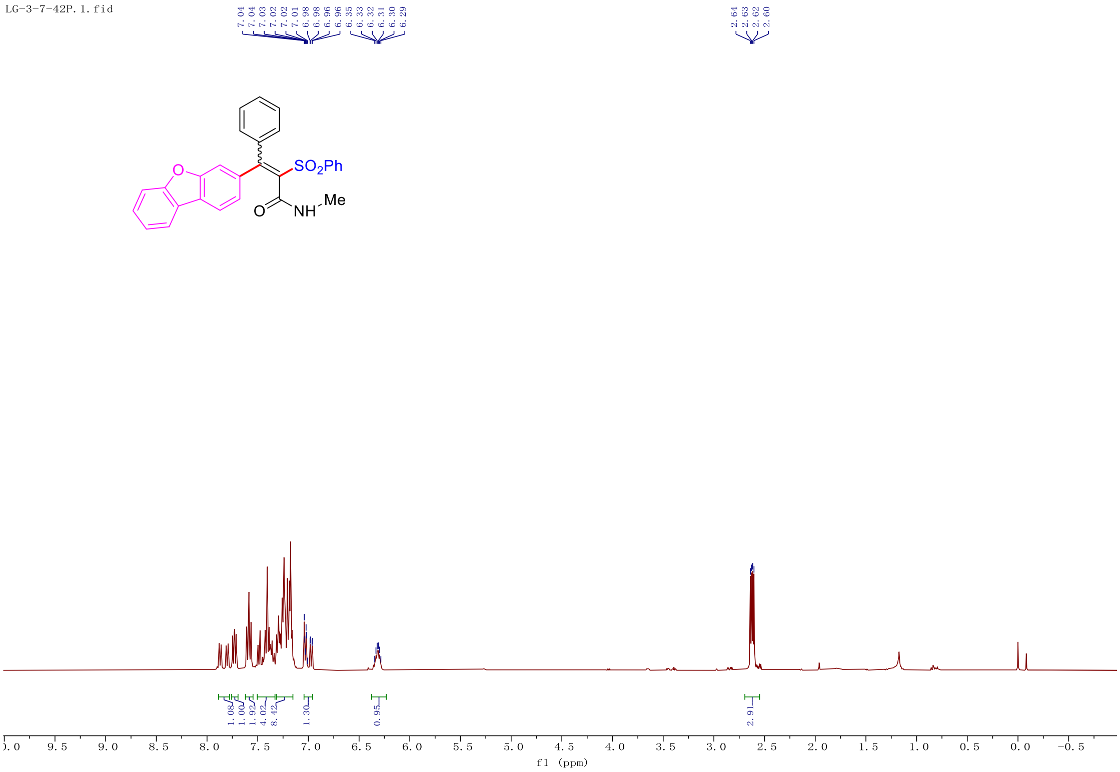

^{13}C {^{1}H} NMR Spectrum of 3-(dibenzo[b,d]furan-3-yl)-*N*-methyl-3-phenyl-2-(phenylsulfonyl)acrylamide (41, $CDCl_3$ as solvent, 101 MHz)

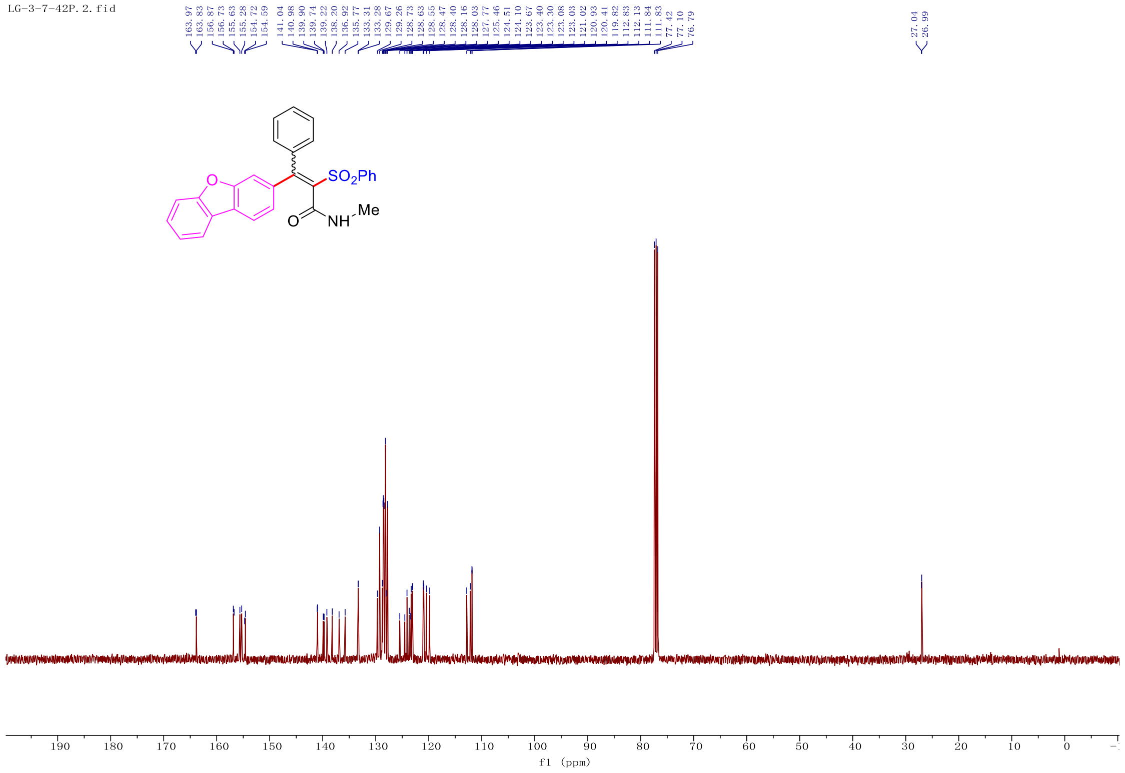

^{1}H NMR Spectrum of methyl 3-(3-(methylamino)-3-oxo-1-phenyl-2-(phenylsulfonyl)prop-1-en-1-yl)thiophene-2-carboxylate (42, $CDCl_3$ as solvent, 400 MHz)

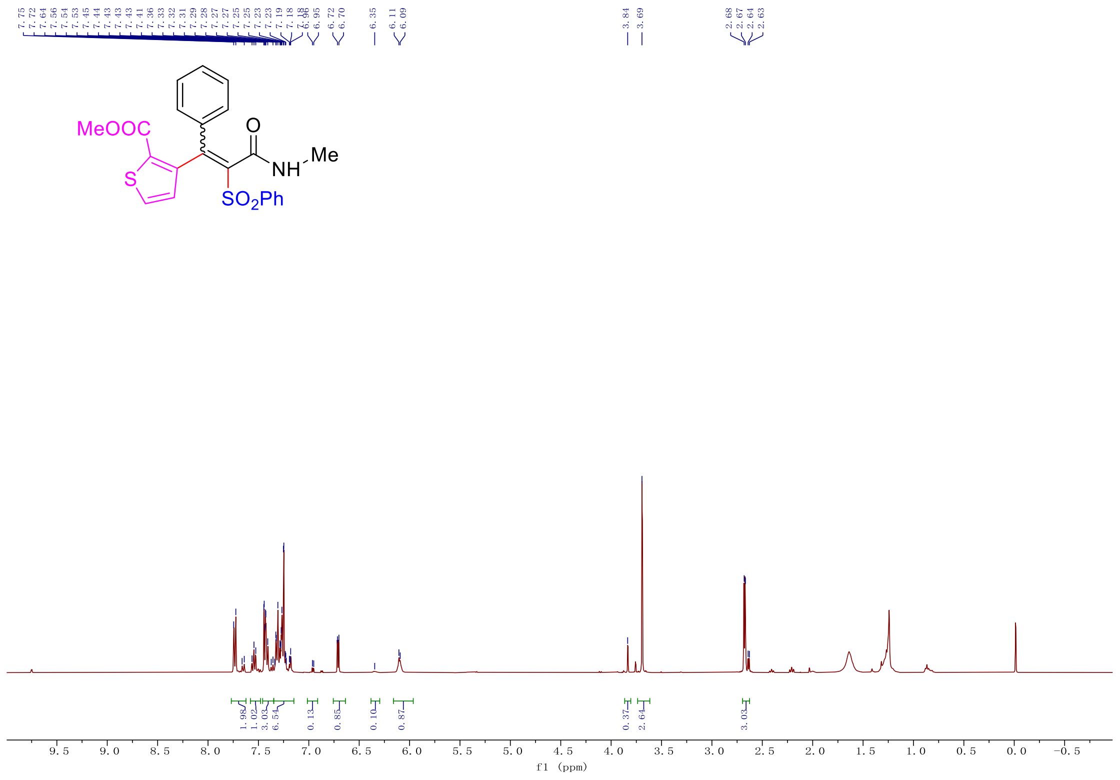

^{13}C {^{1}H} NMR Spectrum of methyl 3-(3-(methylamino)-3-oxo-1-phenyl-2-(phenylsulfonyl)prop-1-en-1-yl)thiophene-2-carboxylate (42, $CDCl_3$ as solvent, 101 MHz)

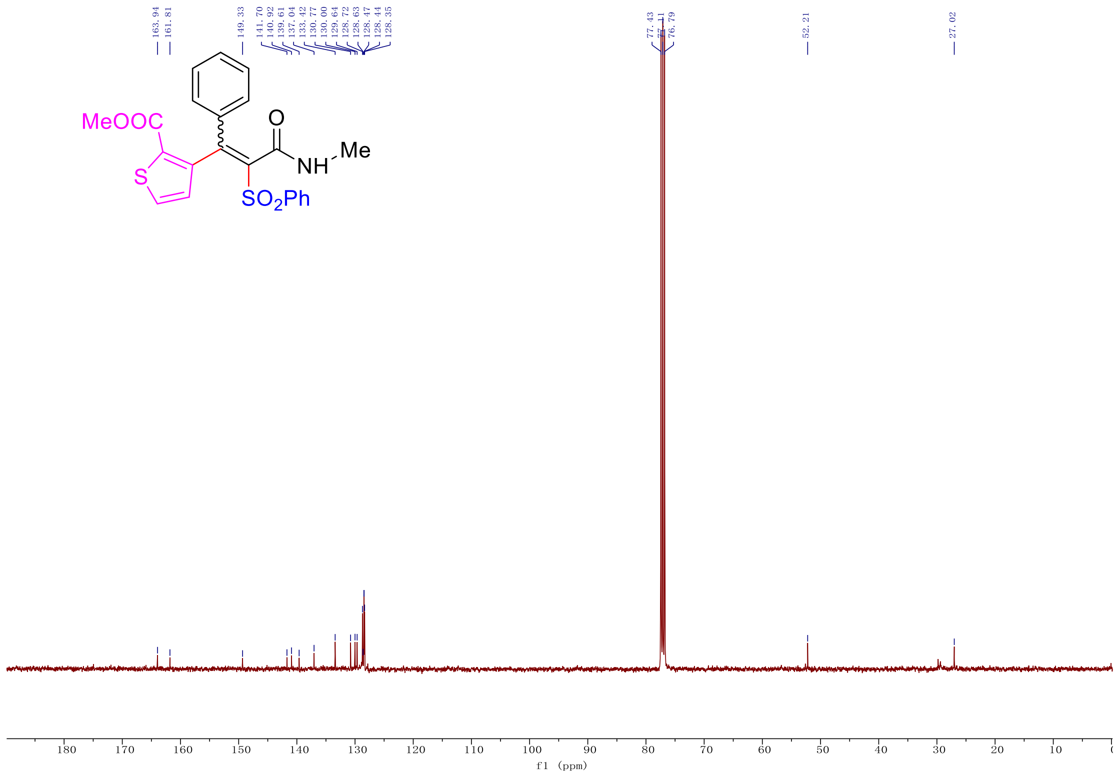

^{1}H NMR Spectrum of (*E*)-N-methyl-N-phenyl-3-(phenylsulfonyl)acrylamide (43, $CDCl_3$ as solvent, 400 MHz)

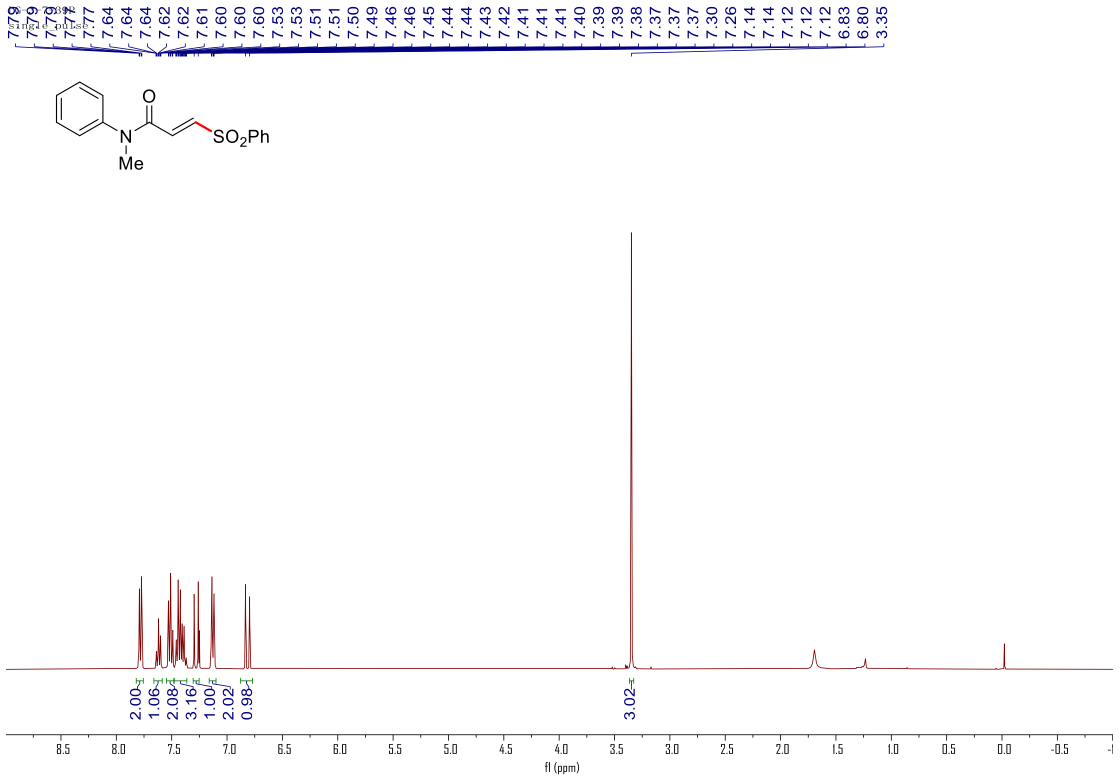

^{13}C {^{1}H} NMR Spectrum of (E)-N-methyl-N-phenyl-3-(phenylsulfonyl)acrylamide (43, $CDCl_3$ as solvent, 101 MHz)

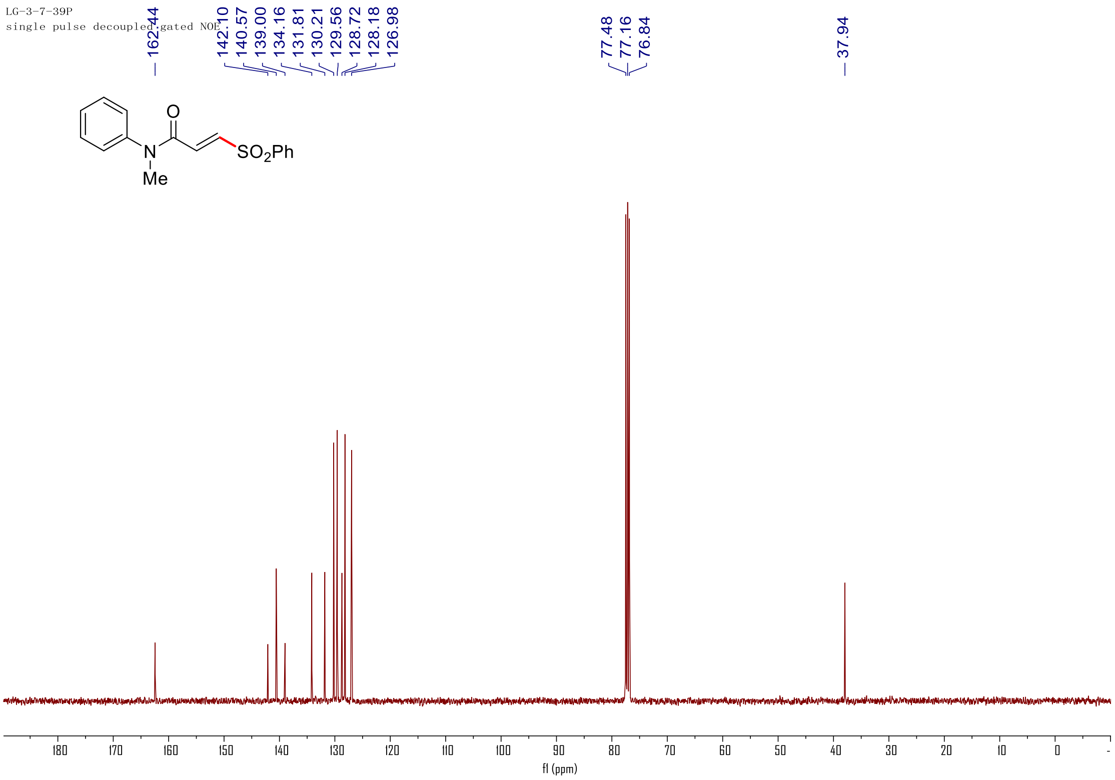

^{1}H NMR Spectrum of *N*-methyl-3,3-diphenyl-2-tosylacrylamide (44, $CDCl_3$ as solvent, 400 MHz)

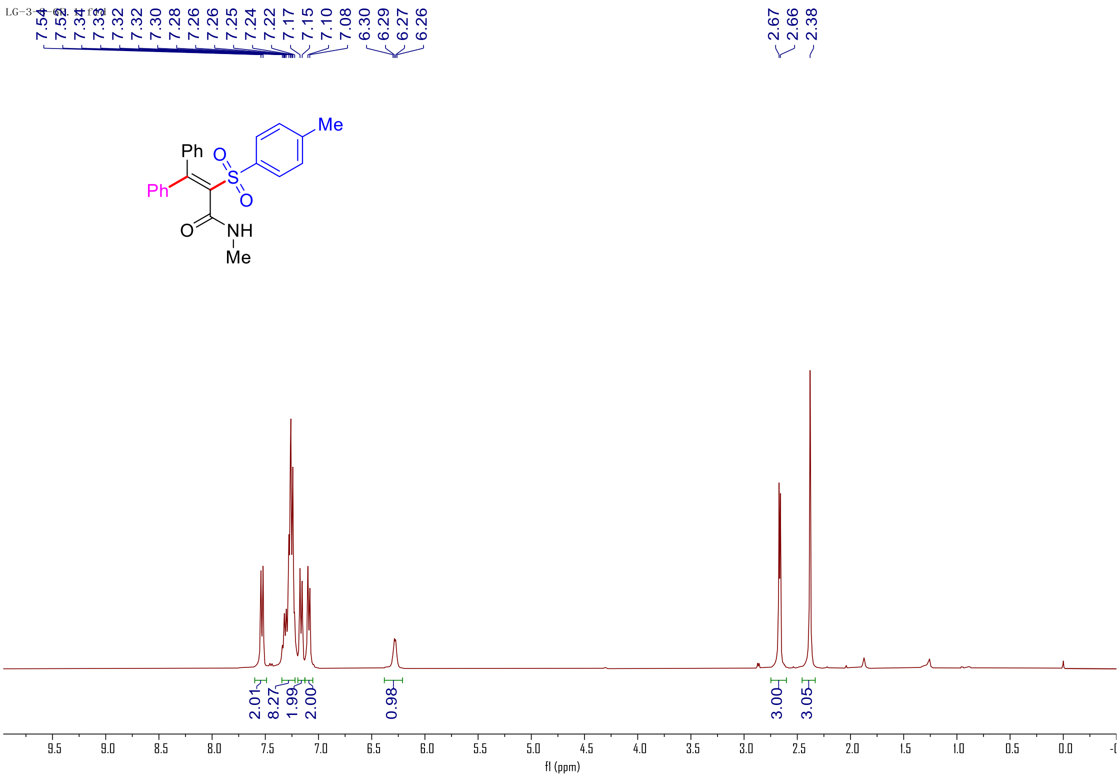

^{13}C {^{1}H} NMR Spectrum of *N*-methyl-3,3-diphenyl-2-tosylacrylamide (44, $CDCl_3$ as solvent, 101 MHz)

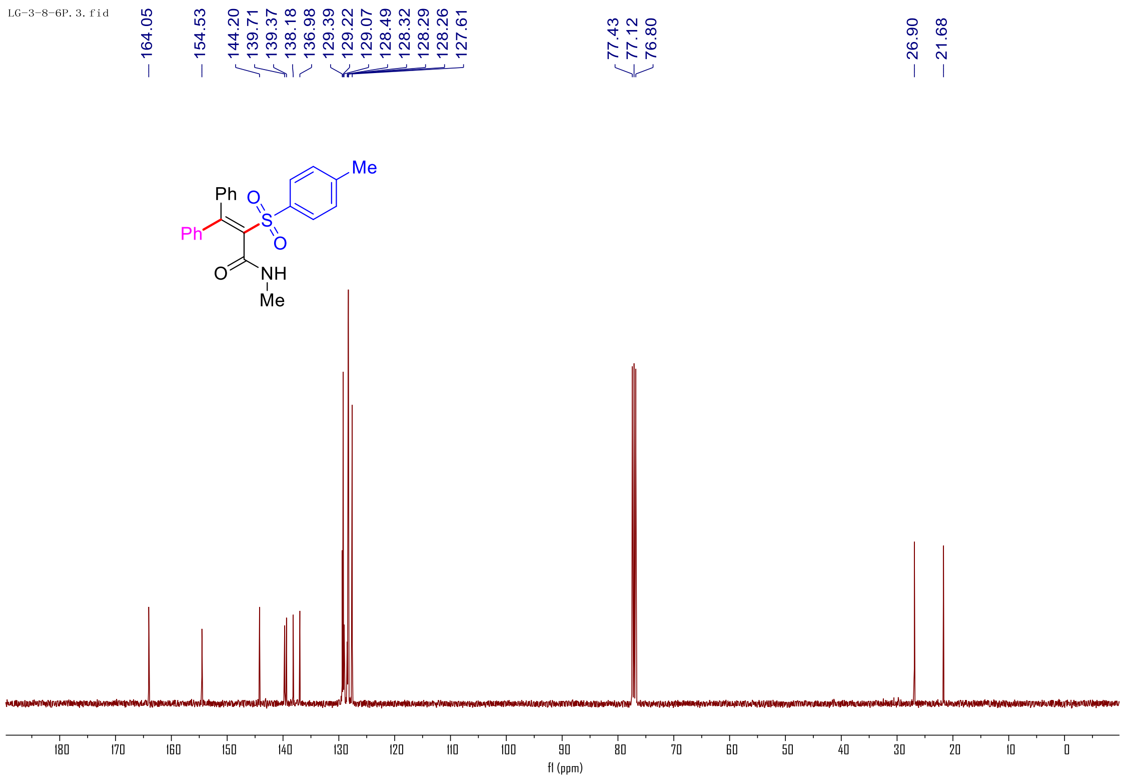

^{1}H NMR Spectrum of 2-((4-isopropylphenyl)sulfonyl)-*N*-methyl-3,3-diphenylacrylamide (45, $CDCl_3$ as solvent, 400 MHz)

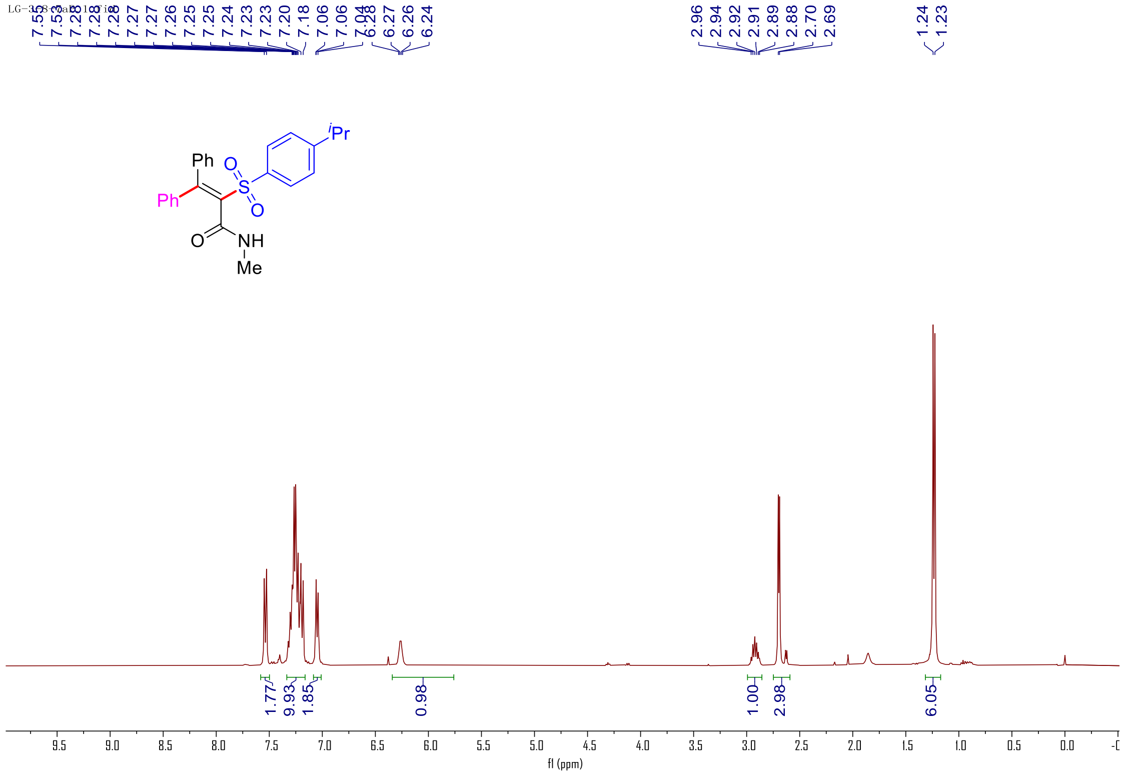

^{13}C {^{1}H} NMR Spectrum of 2-((4-isopropylphenyl)sulfonyl)-*N*-methyl-3,3-diphenylacrylamide (45, $CDCl_3$ as solvent, 101 MHz)

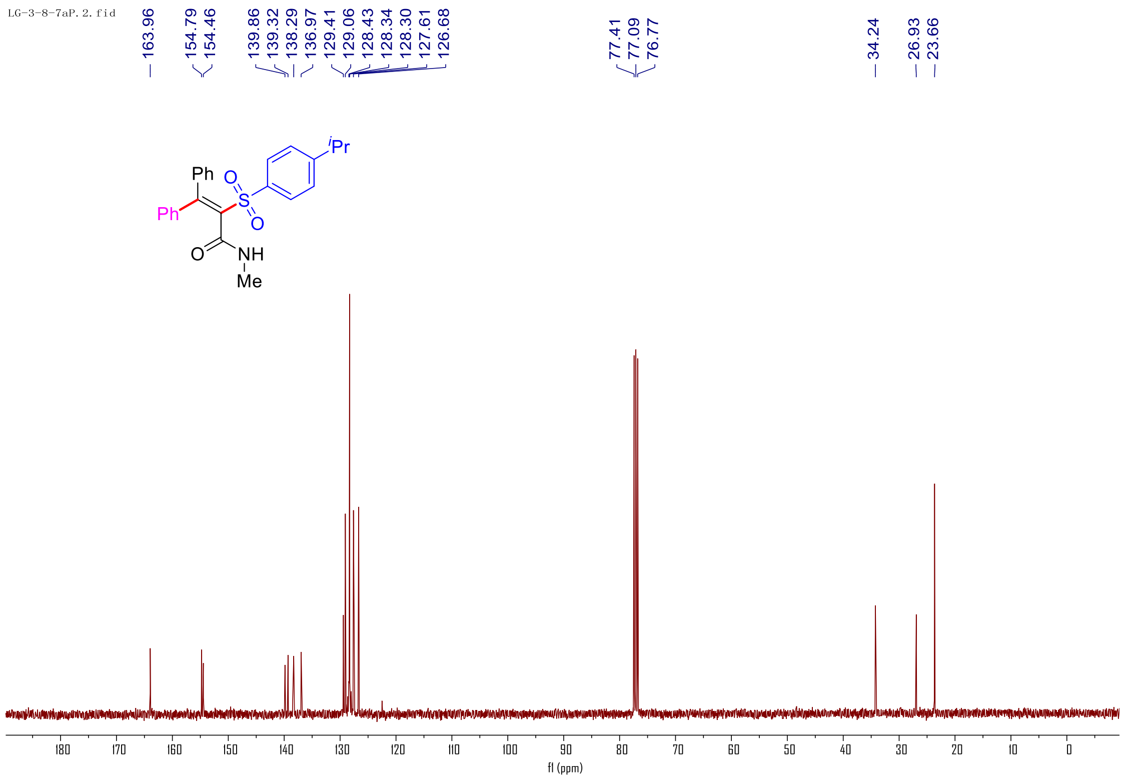

^{1}H NMR Spectrum of 2-((4-chlorophenyl)sulfonyl)-*N*-methyl-3,3-diphenylacrylamide (46, $CDCl_3$ as solvent, 400 MHz)

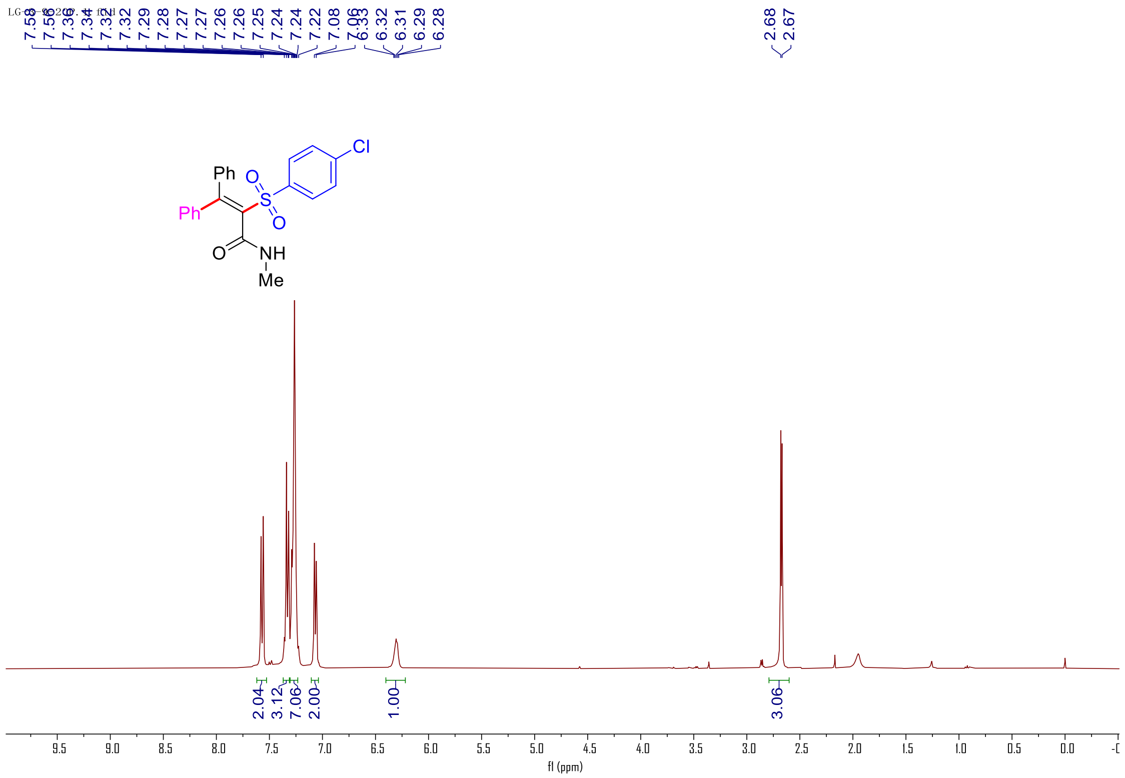

^{13}C {^{1}H} NMR Spectrum of 2-((4-chlorophenyl)sulfonyl)-*N*-methyl-3,3-diphenylacrylamide (46, $CDCl_3$ as solvent, 101 MHz)

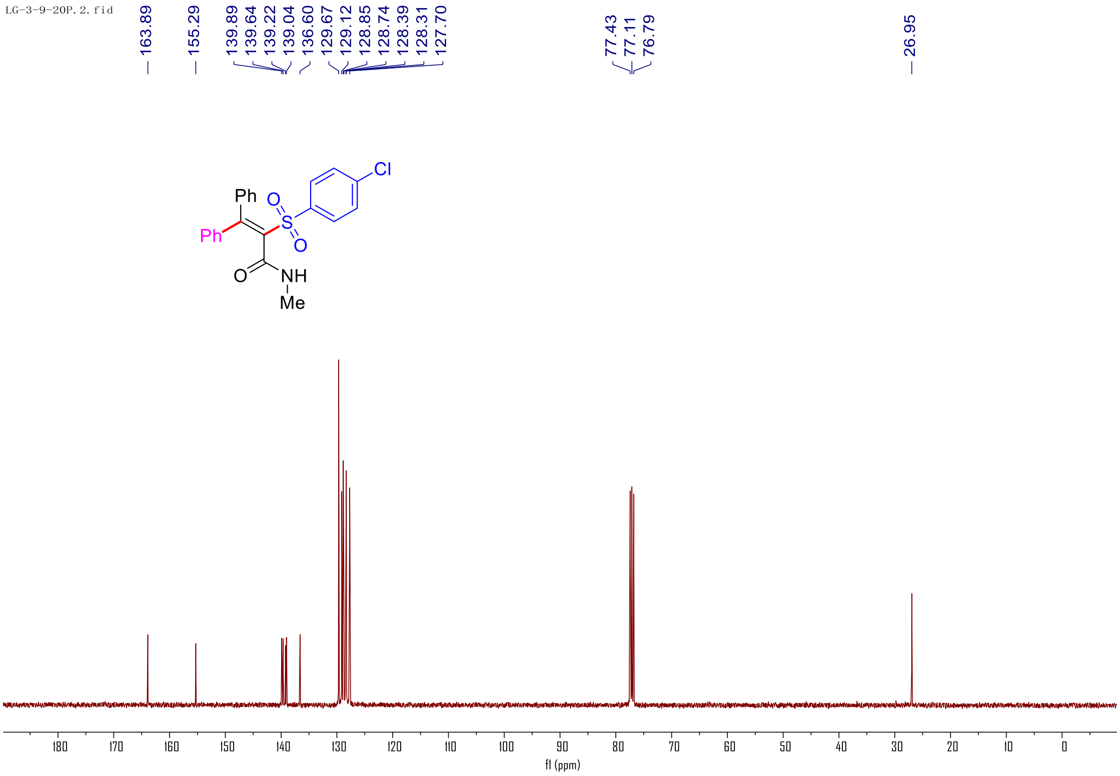

^{1}H NMR Spectrum of *N*-methyl-3,3-diphenyl-2-((4-(trifluoromethyl)phenyl)sulfonyl)acrylamide (47, $CDCl_3$ as solvent, 400 MHz)

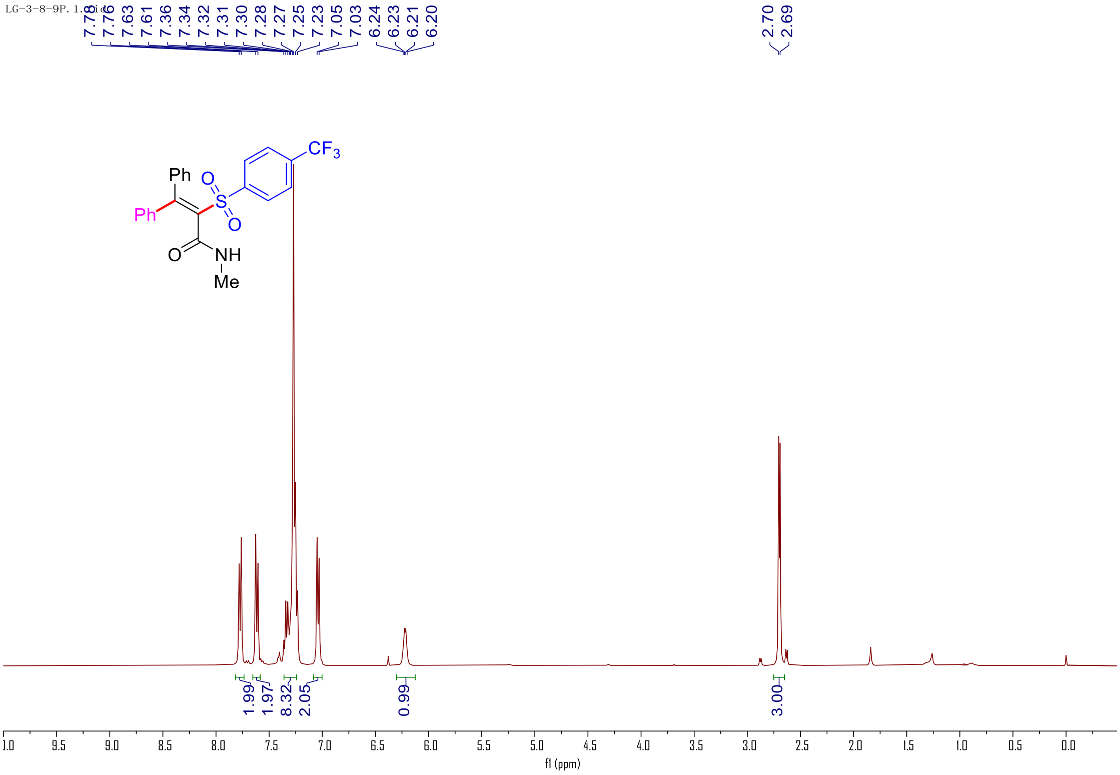

^{13}C {^{1}H} NMR Spectrum of *N*-methyl-3,3-diphenyl-2-((4-(trifluoromethyl)phenyl)sulfonyl)acrylamide (47, $CDCl_3$ as solvent, 101 MHz)

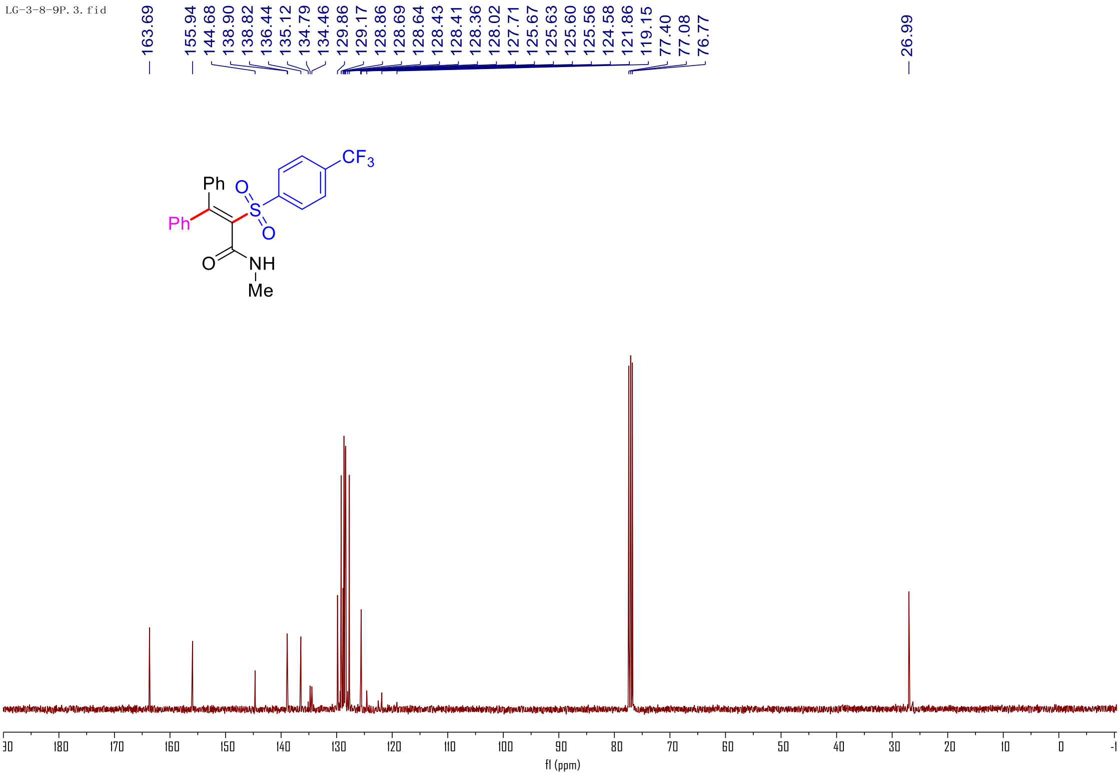

^{19}F NMR Spectrum of *N*-methyl-3,3-diphenyl-2-((3-(trifluoromethyl)phenyl)sulfonyl)acrylamide (47, $CDCl_3$ as solvent, 376 MHz)

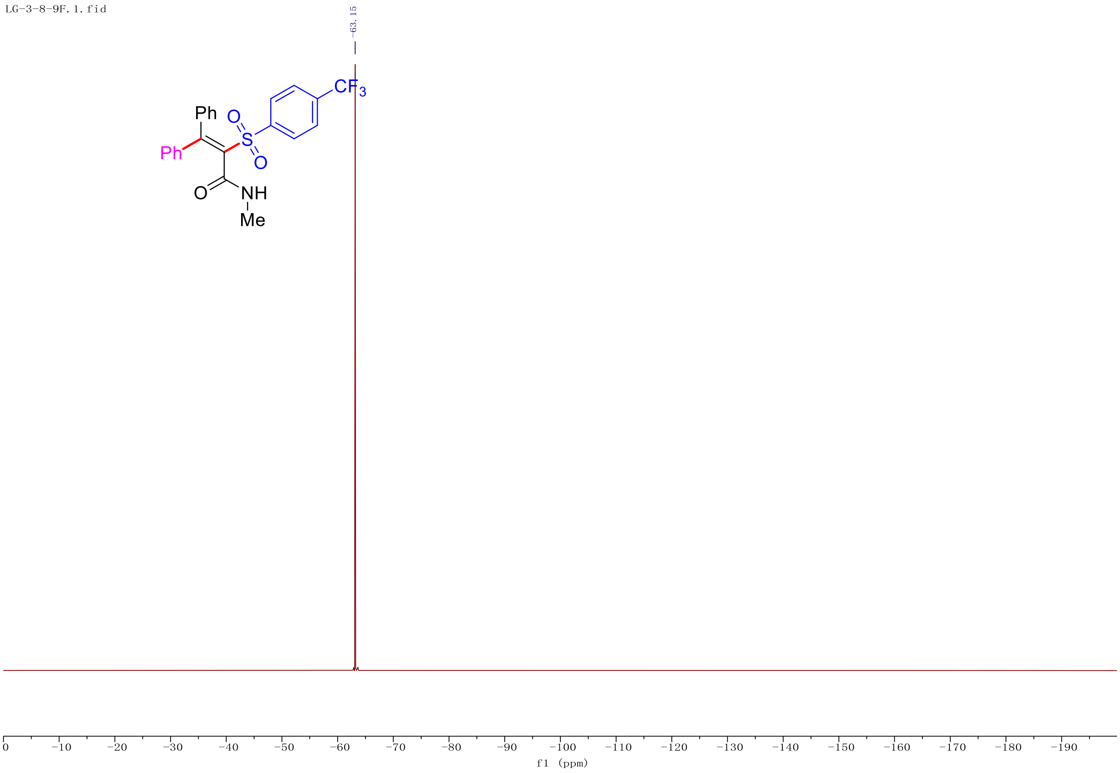

^{1}H NMR Spectrum of 2-((4-cyanophenyl)sulfonyl)-*N*-methyl-3,3-diphenylacrylamide (48, $CDCl_3$ as solvent, 400 MHz)

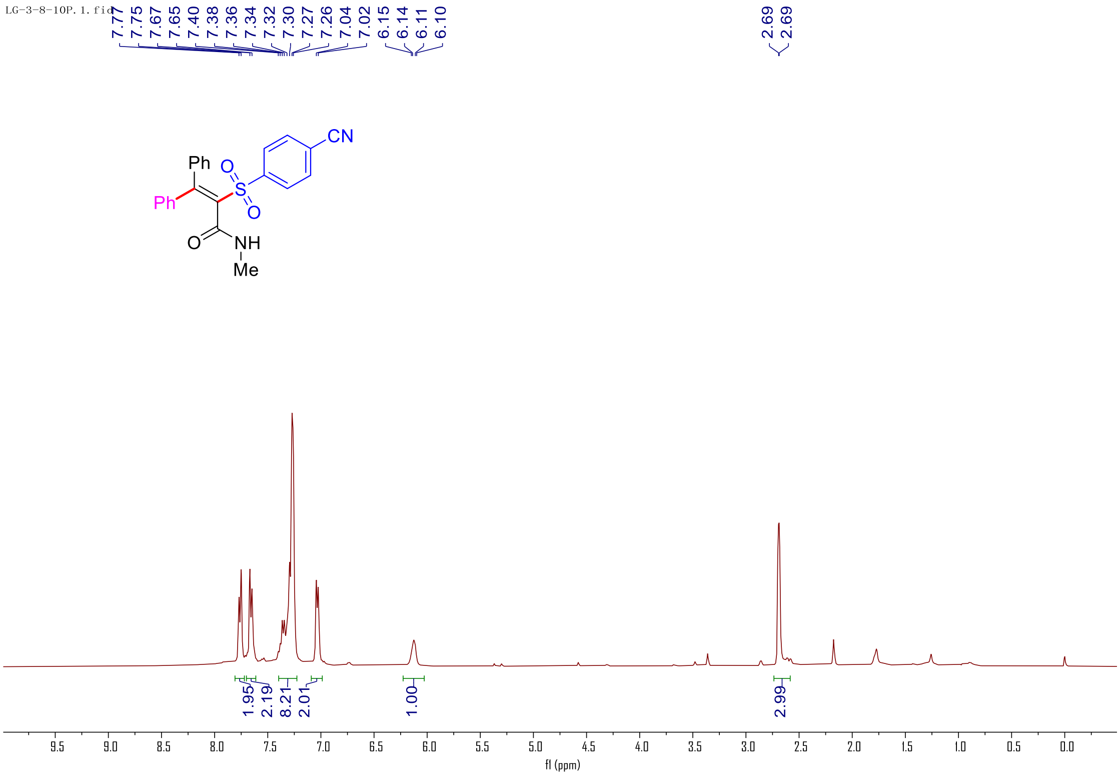

^{13}C {^{1}H} NMR Spectrum of 2-((4-cyanophenyl)sulfonyl)-*N*-methyl-3,3-diphenylacrylamide (48, $CDCl_3$ as solvent, 101 MHz)

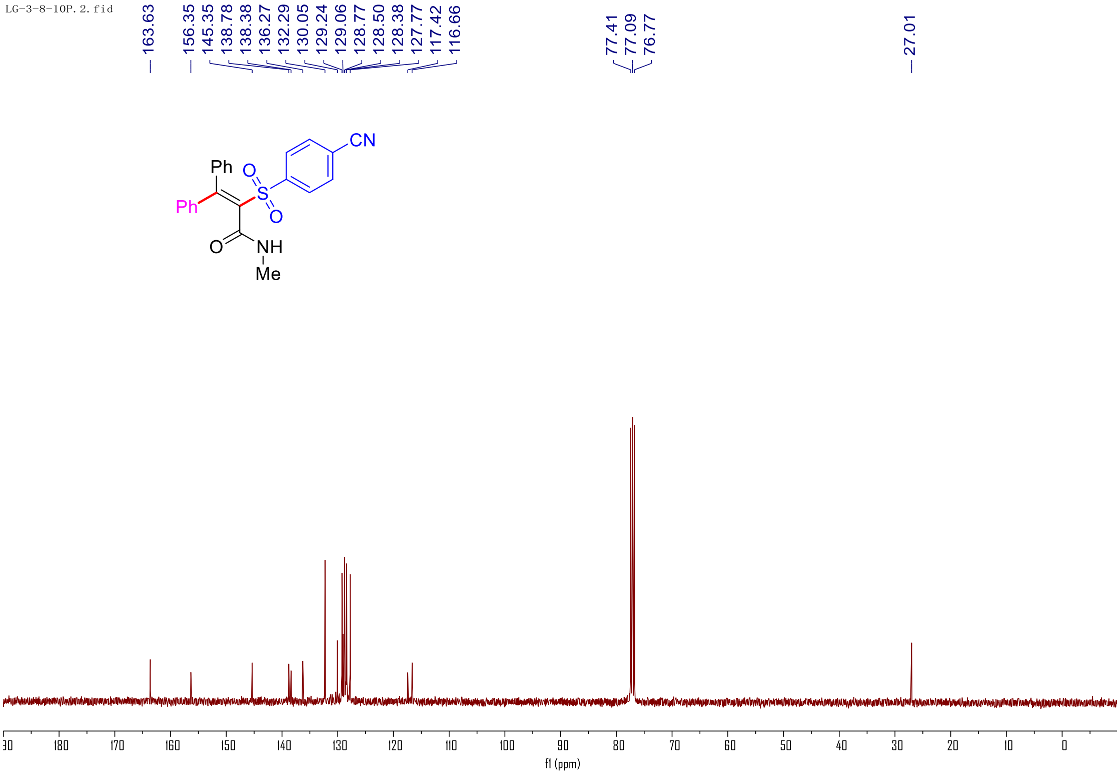

^{1}H NMR Spectrum of 2-((4-acetylphenyl)sulfonyl)-*N*-methyl-3,3-diphenylacrylamide (49, $CDCl_3$ as solvent, 400 MHz)

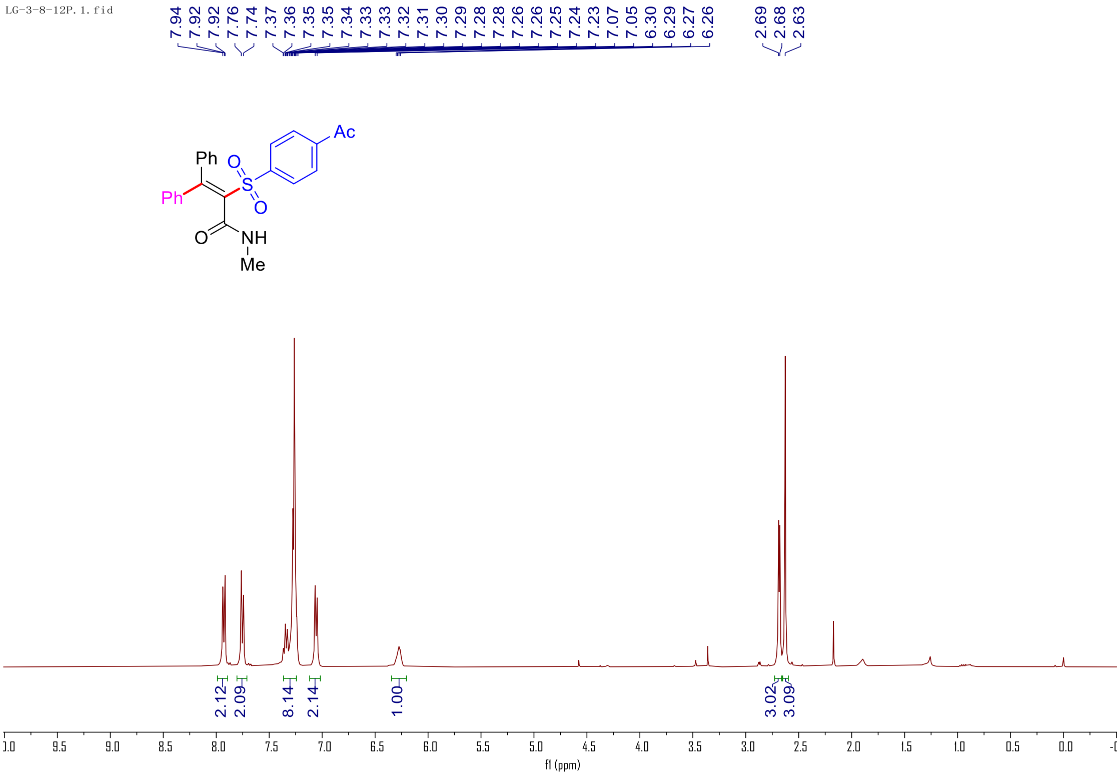

^{13}C {^{1}H} NMR Spectrum of 2-((4-acetylphenyl)sulfonyl)-*N*-methyl-3,3-diphenylacrylamide (49, $CDCl_3$ as solvent, 101 MHz)

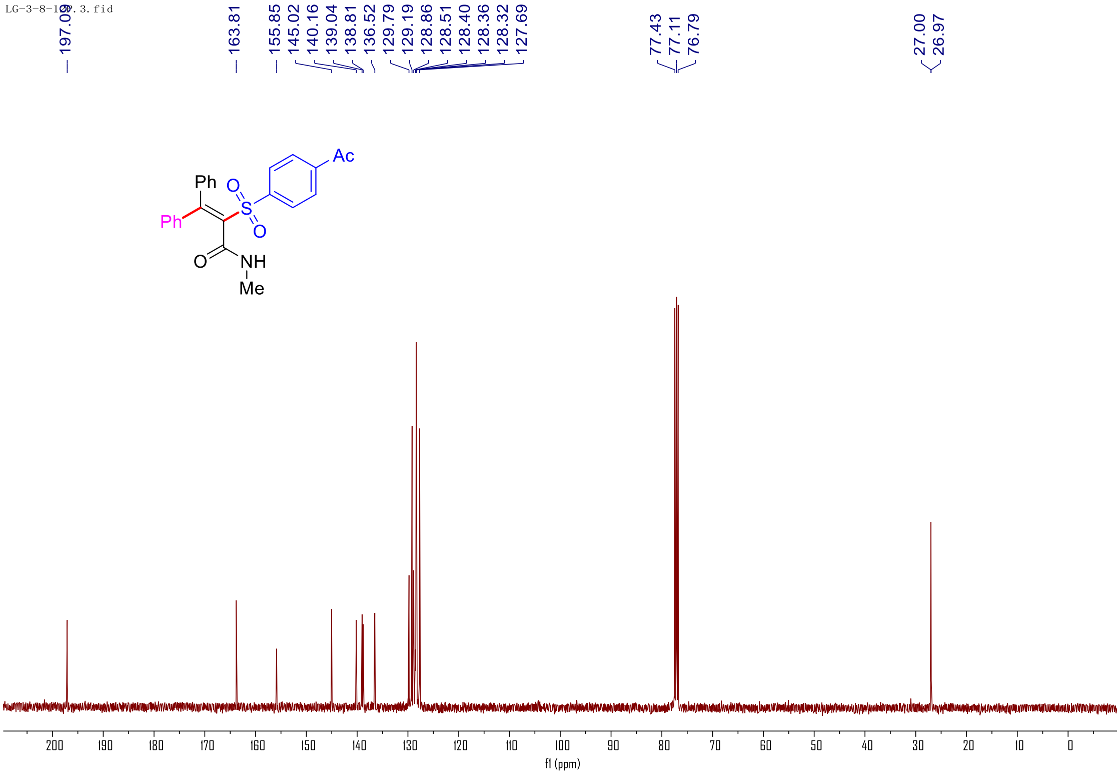

^{1}H NMR Spectrum of 2-((2-methoxyphenyl)sulfonyl)-*N*-methyl-3,3-diphenylacrylamide (**50**, $CDCl_3$ as solvent, 400 MHz)

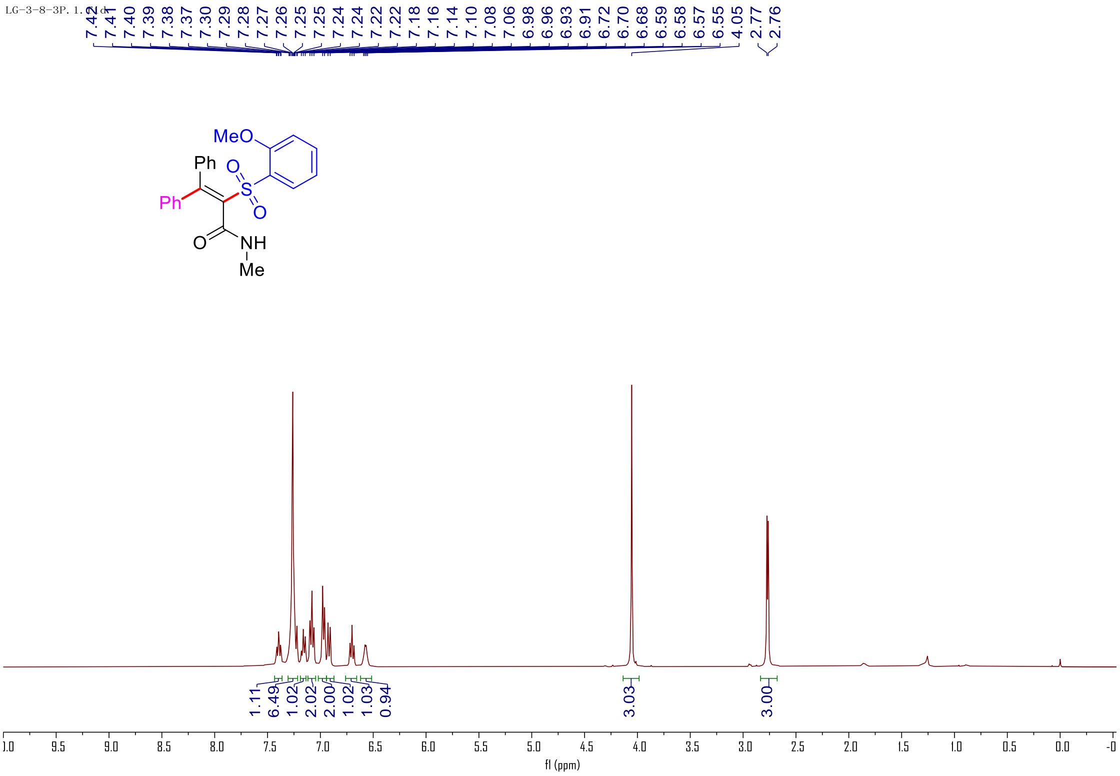

^{13}C {^{1}H} NMR Spectrum of 2-((2-methoxyphenyl)sulfonyl)-*N*-methyl-3,3-diphenylacrylamide (**50**, $CDCl_3$ as solvent, 101 MHz)

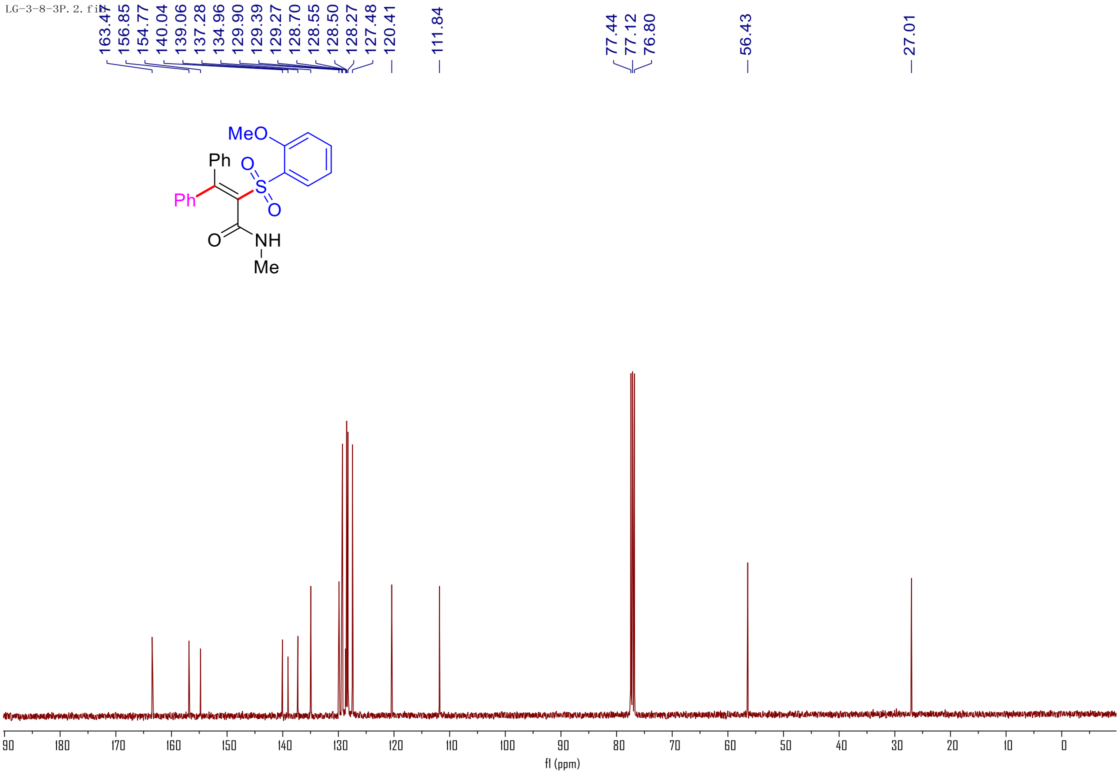

^{1}H NMR Spectrum of 2-((2-fluorophenyl)sulfonyl)-*N*-methyl-3,3-diphenylacrylamide (51, $CDCl_3$ as solvent, 400 MHz)

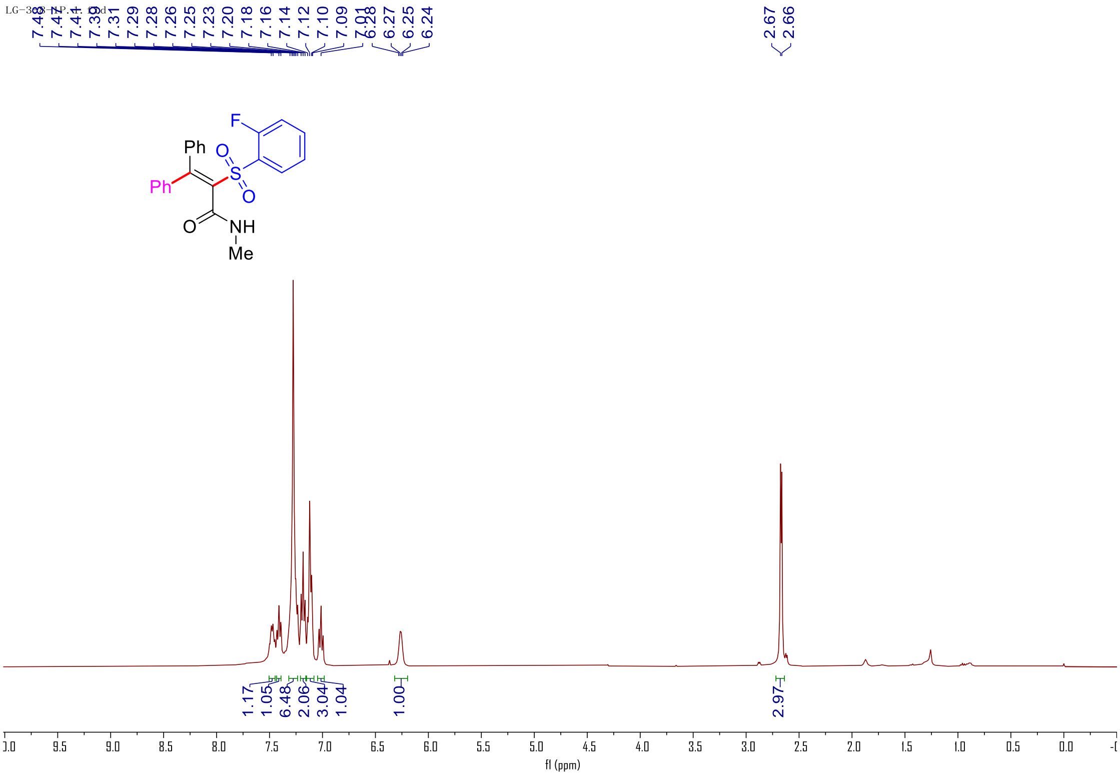

^{13}C {^{1}H} NMR Spectrum of 2-((2-fluorophenyl)sulfonyl)-*N*-methyl-3,3-diphenylacrylamide (51, $CDCl_3$ as solvent, 101 MHz)

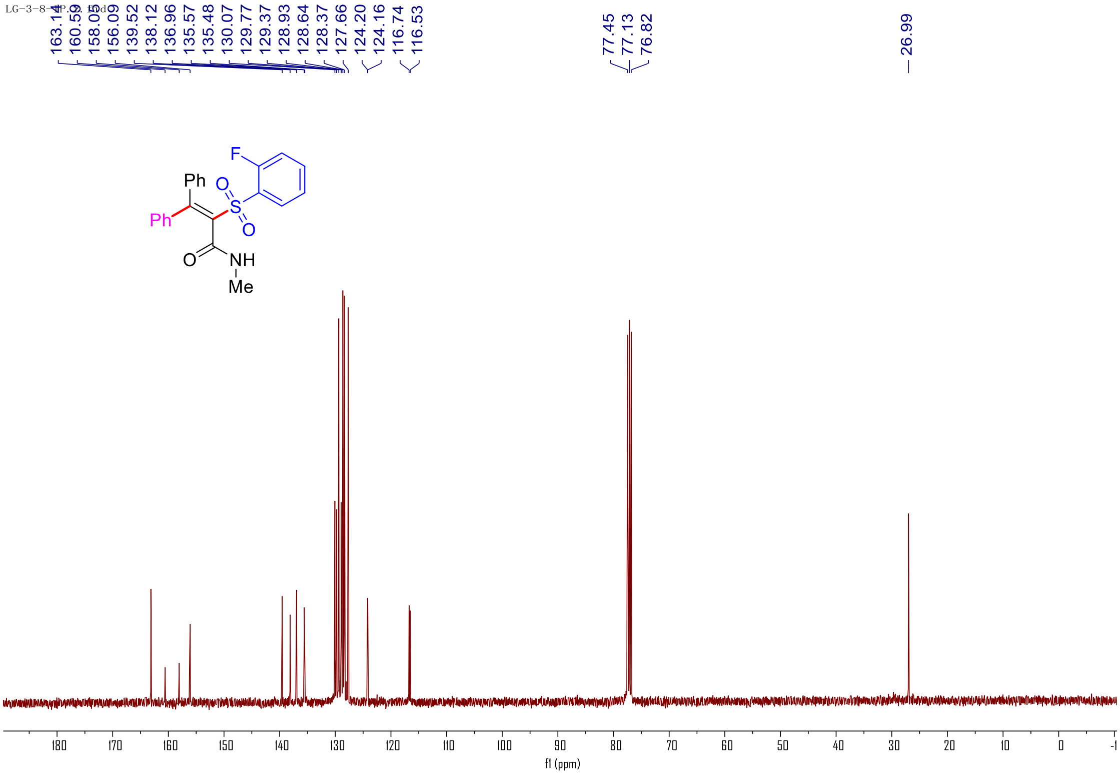

^{19}F NMR Spectrum of 2-((2-fluorophenyl)sulfonyl)-*N*-methyl-3,3-diphenylacrylamide (51, $CDCl_3$ as solvent, 376 MHz)

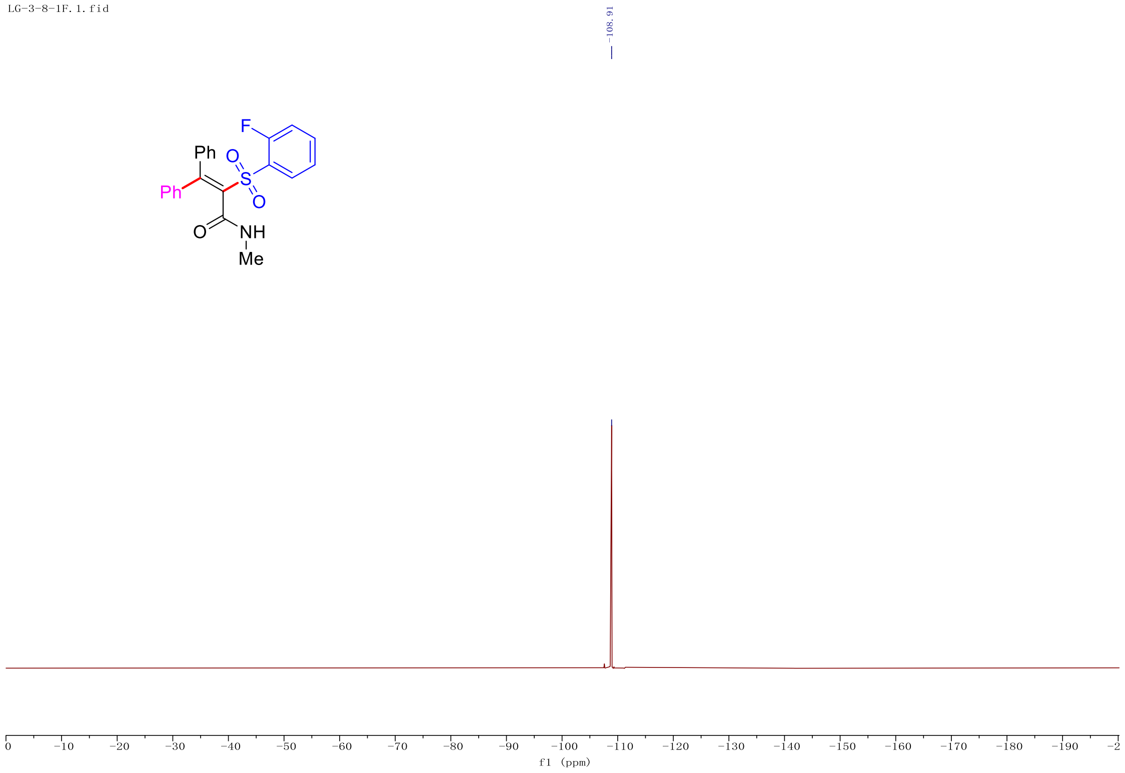

^{1}H NMR Spectrum of methyl 2-((3-(methylamino)-3-oxo-1,1-diphenylprop-1-en-2-yl)sulfonyl)benzoate (52, $CDCl_3$ as solvent, 400 MHz)

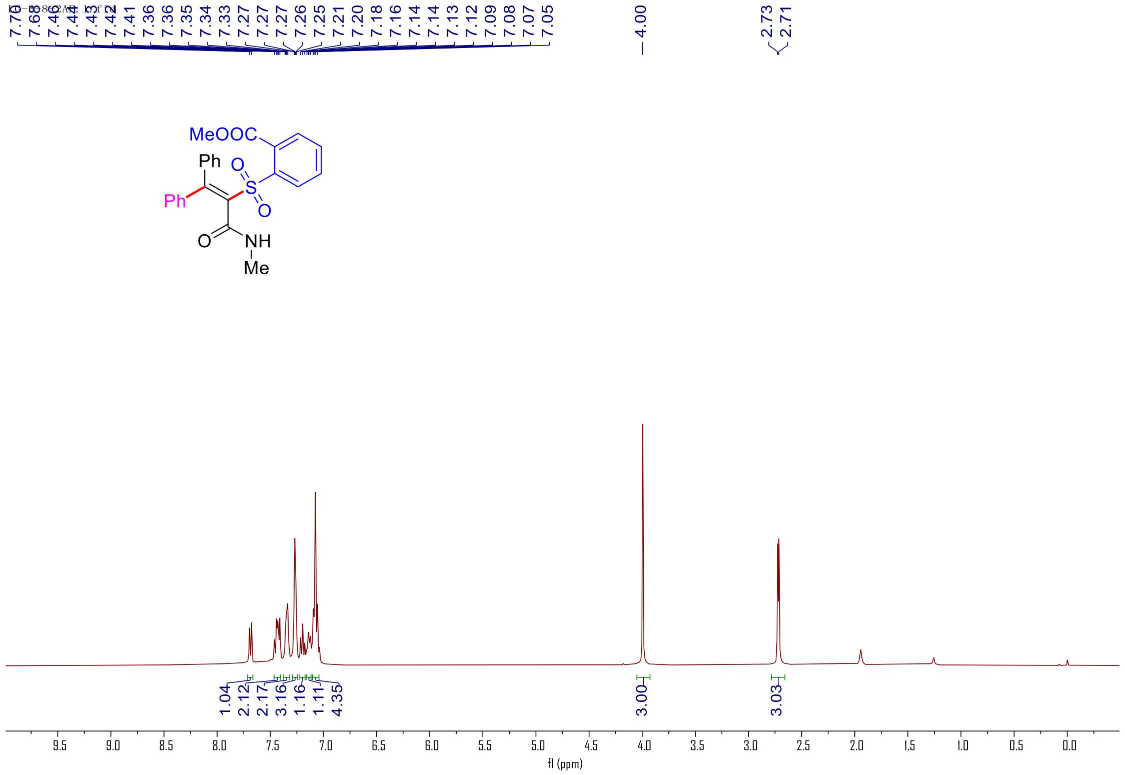

^{13}C {^{1}H} NMR Spectrum of methyl 2-((3-(methylamino)-3-oxo-1,1-diphenylprop-1-en-2-yl)sulfonyl)benzoate (52, $CDCl_3$ as solvent, 101 MHz)

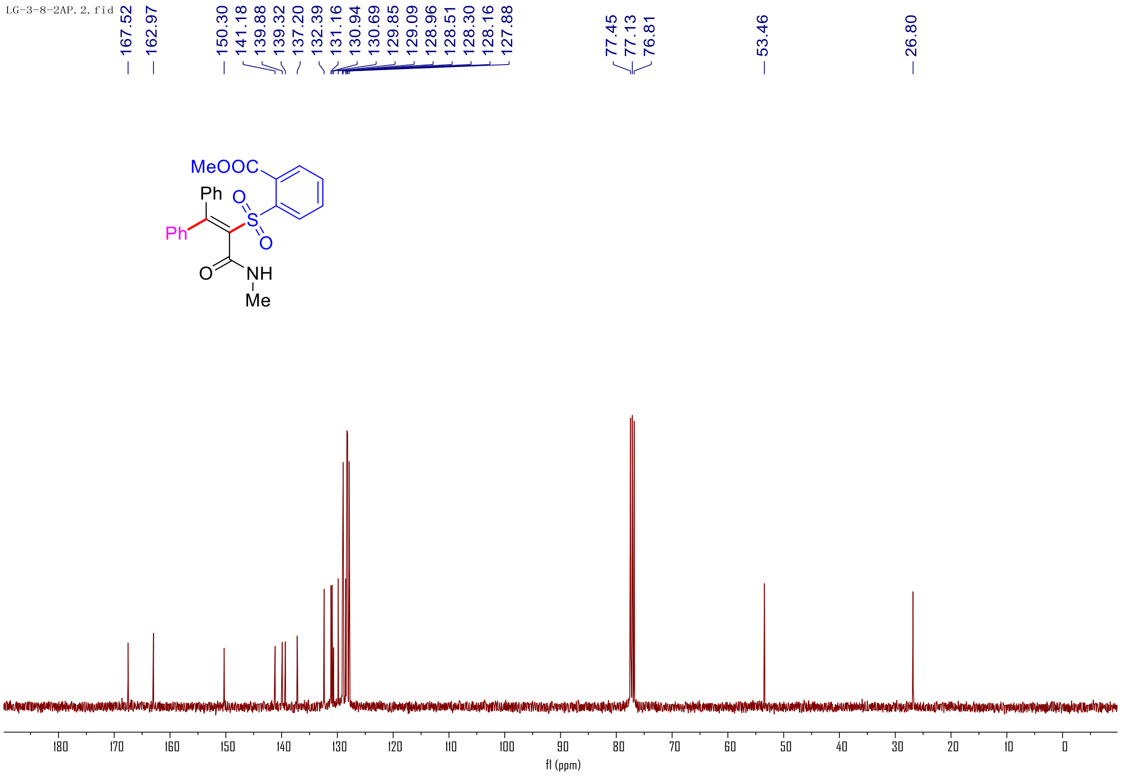

^{1}H NMR Spectrum of 2-((3-bromophenyl)sulfonyl)-*N*-methyl-3,3-diphenylacrylamide (53, $CDCl_3$ as solvent, 400 MHz)

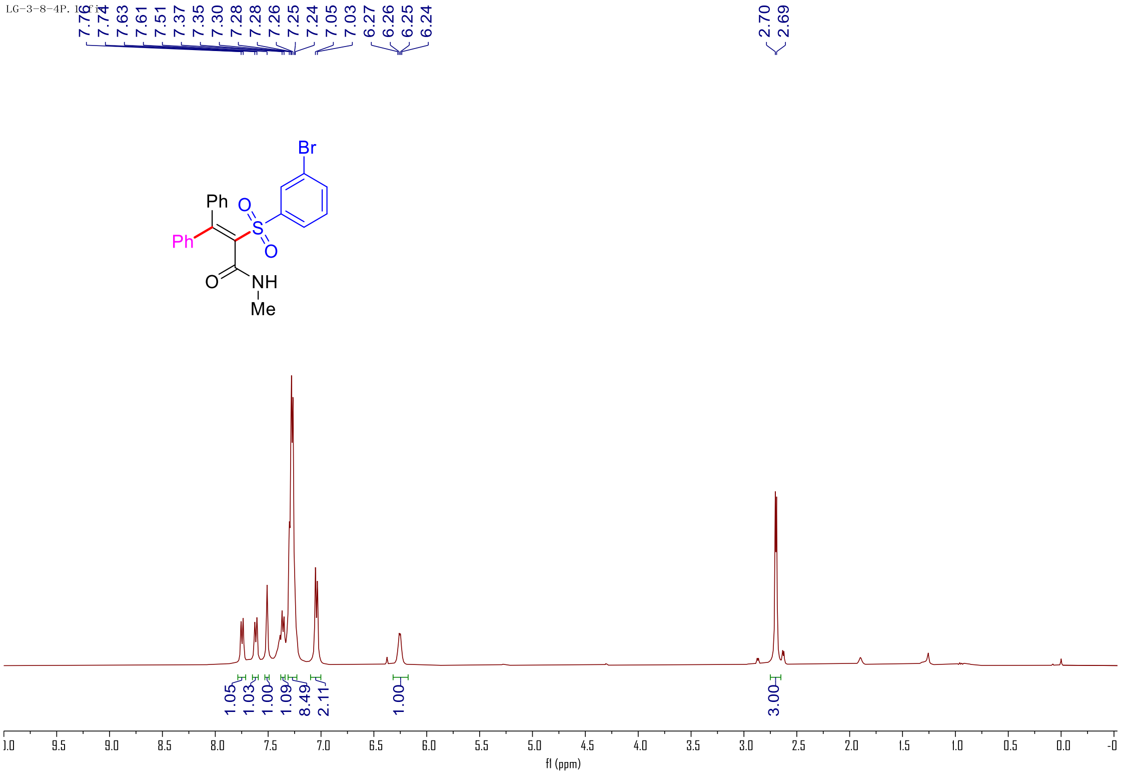

^{13}C {^{1}H} NMR Spectrum of 2-((3-bromophenyl)sulfonyl)-*N*-methyl-3,3-diphenylacrylamide (53, $CDCl_3$ as solvent, 101 MHz)

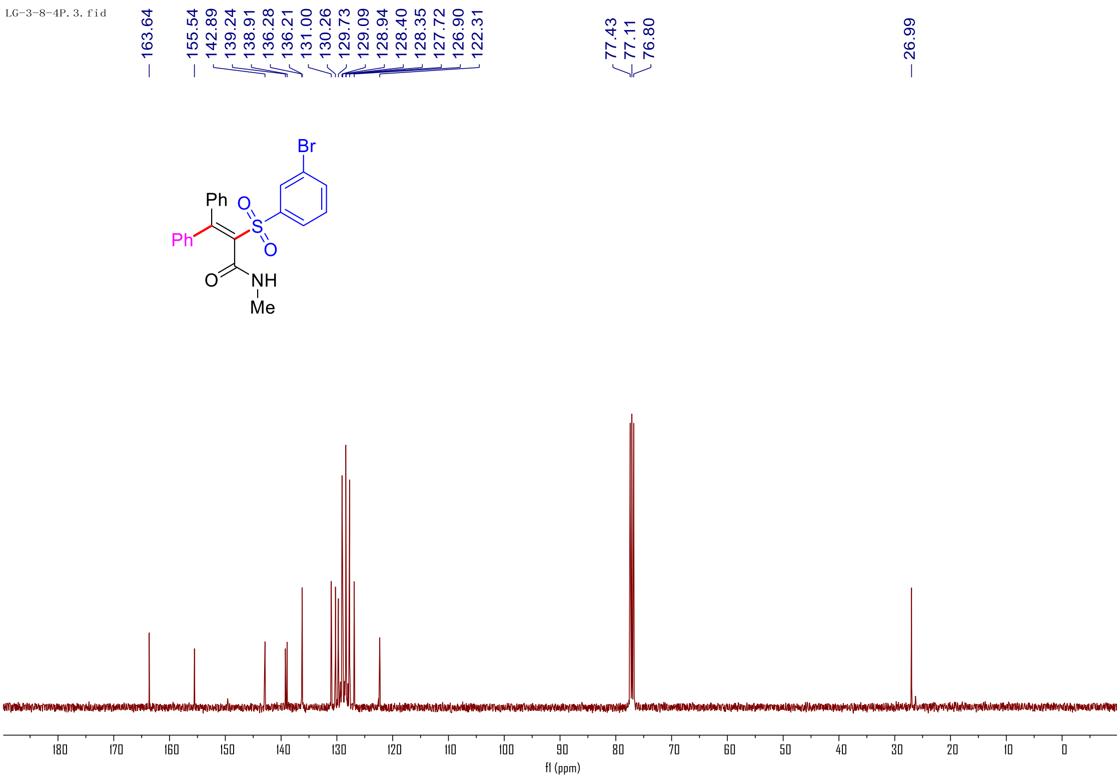

^{1}H NMR Spectrum of *N*-methyl-3,3-diphenyl-2-((3-(trifluoromethyl)phenyl)sulfonyl)acrylamide (54, $CDCl_3$ as solvent, 400 MHz)

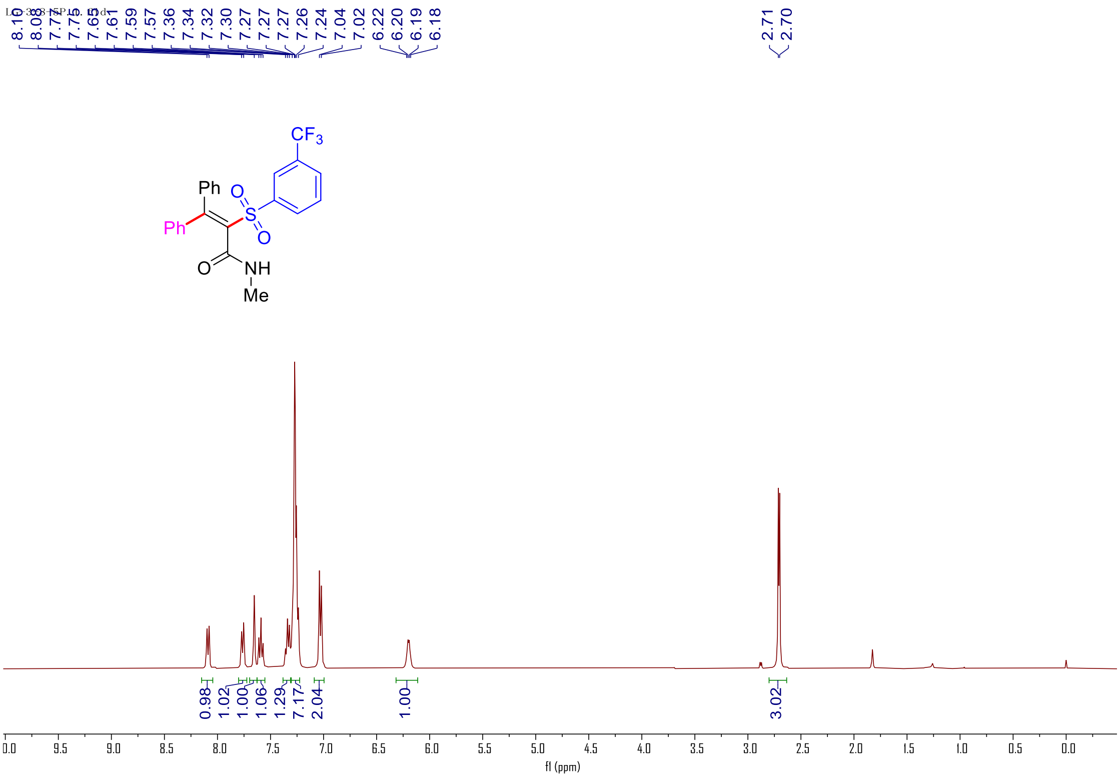

^{13}C {^{1}H} NMR Spectrum of *N*-methyl-3,3-diphenyl-2-((3-(trifluoromethyl)phenyl)sulfonyl)acrylamide (54, $CDCl_3$ as solvent, 101 MHz)

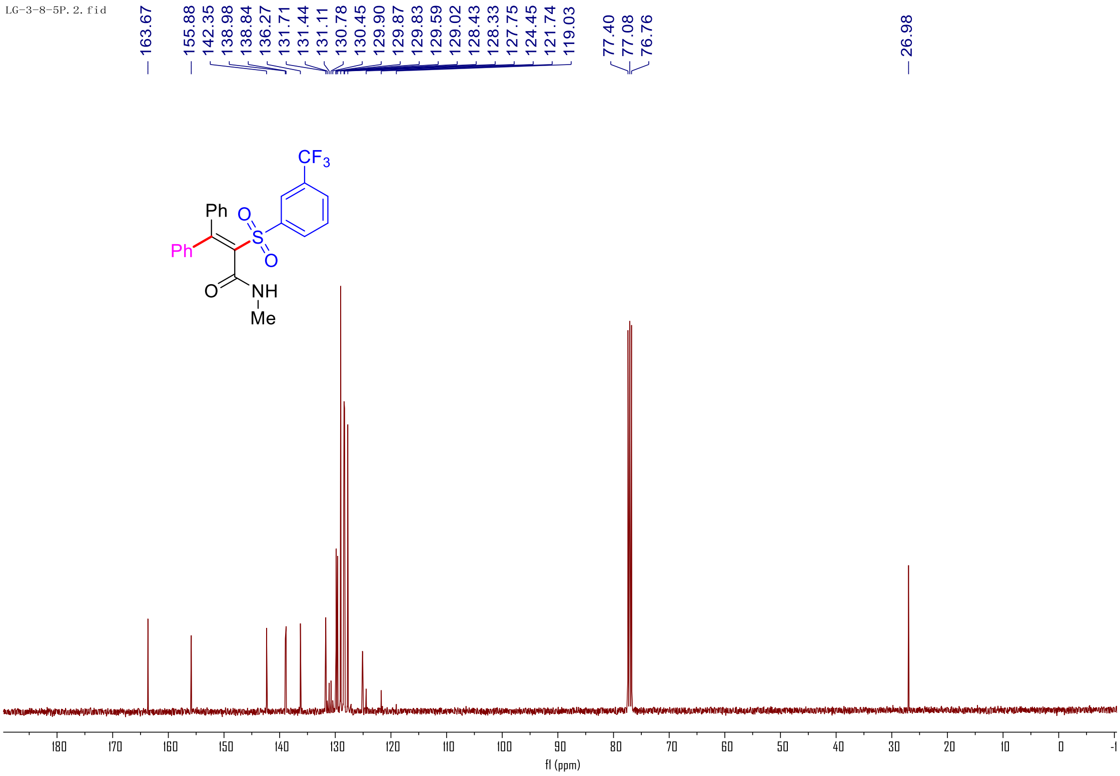

^{19}F NMR Spectrum of *N*-methyl-3,3-diphenyl-2-((3-(trifluoromethyl)phenyl)sulfonyl)acrylamide (54, $CDCl_3$ as solvent, 376 MHz)

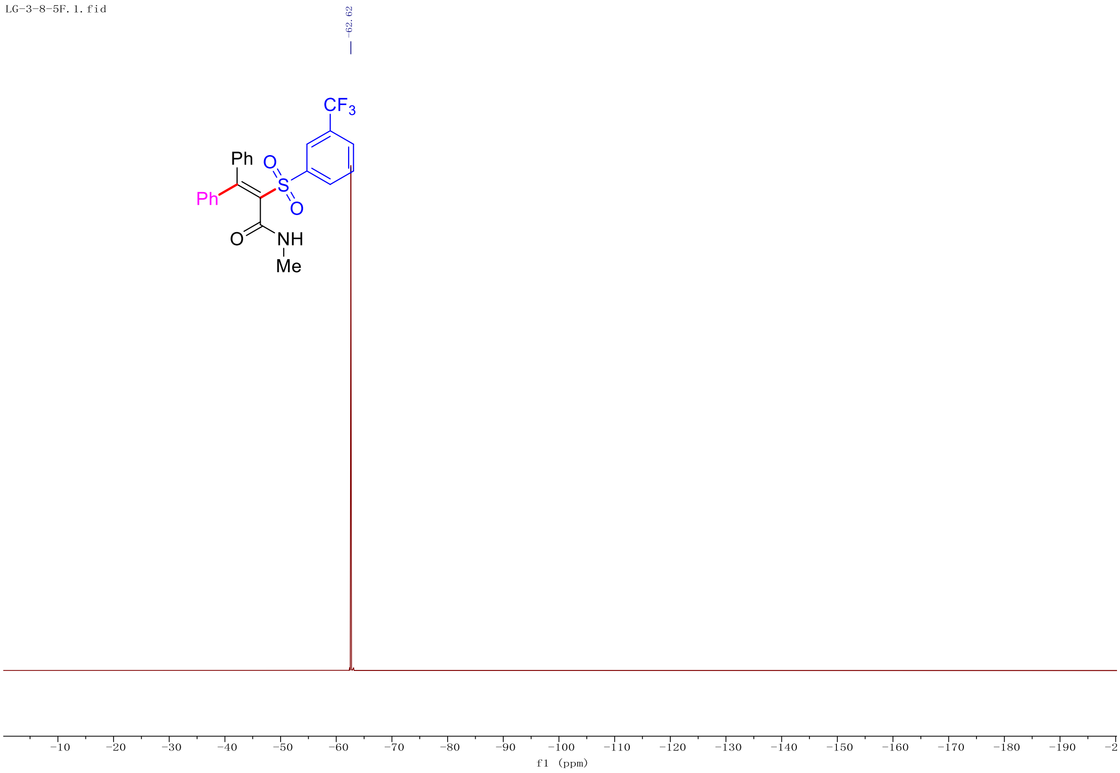

¹H NMR Spectrum of 2-((3,5-dimethylphenyl)sulfonyl)-*N*-methyl-3,3-diphenylacrylamide (55, $CDCl_3$ as solvent, 400 MHz)

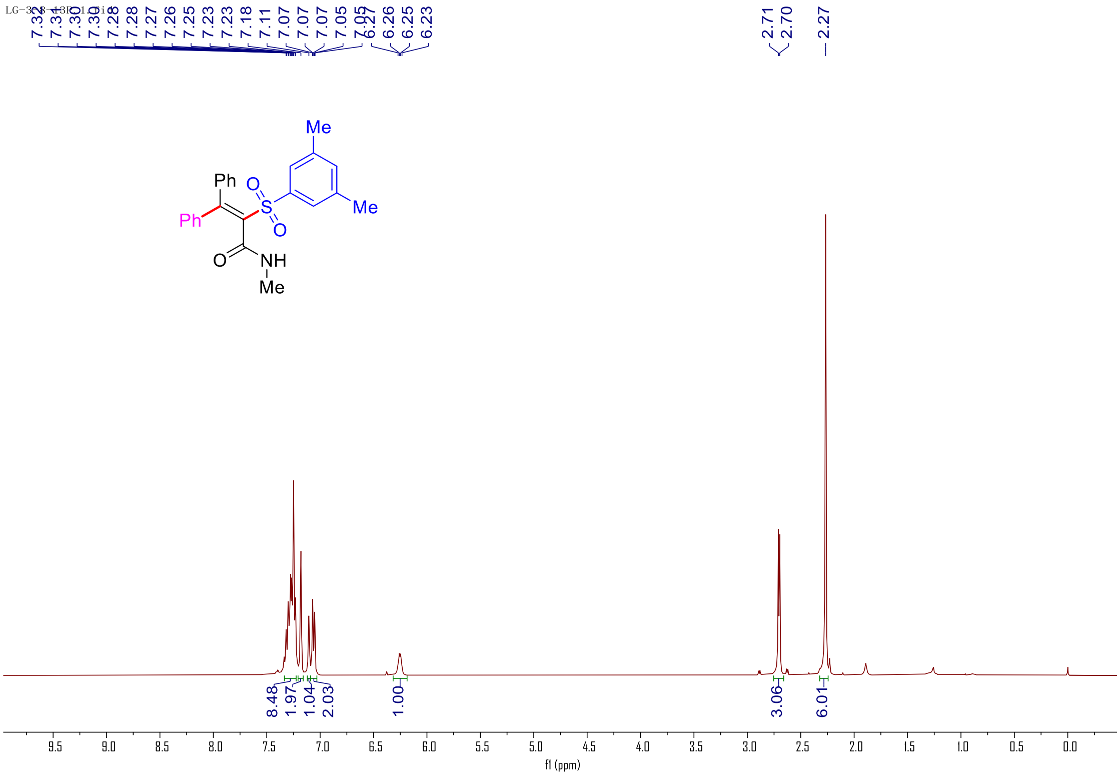

¹³C {¹H} NMR Spectrum of 2-((3,5-dimethylphenyl)sulfonyl)-*N*-methyl-3,3-diphenylacrylamide (55, $CDCl_3$ as solvent, 101 MHz)

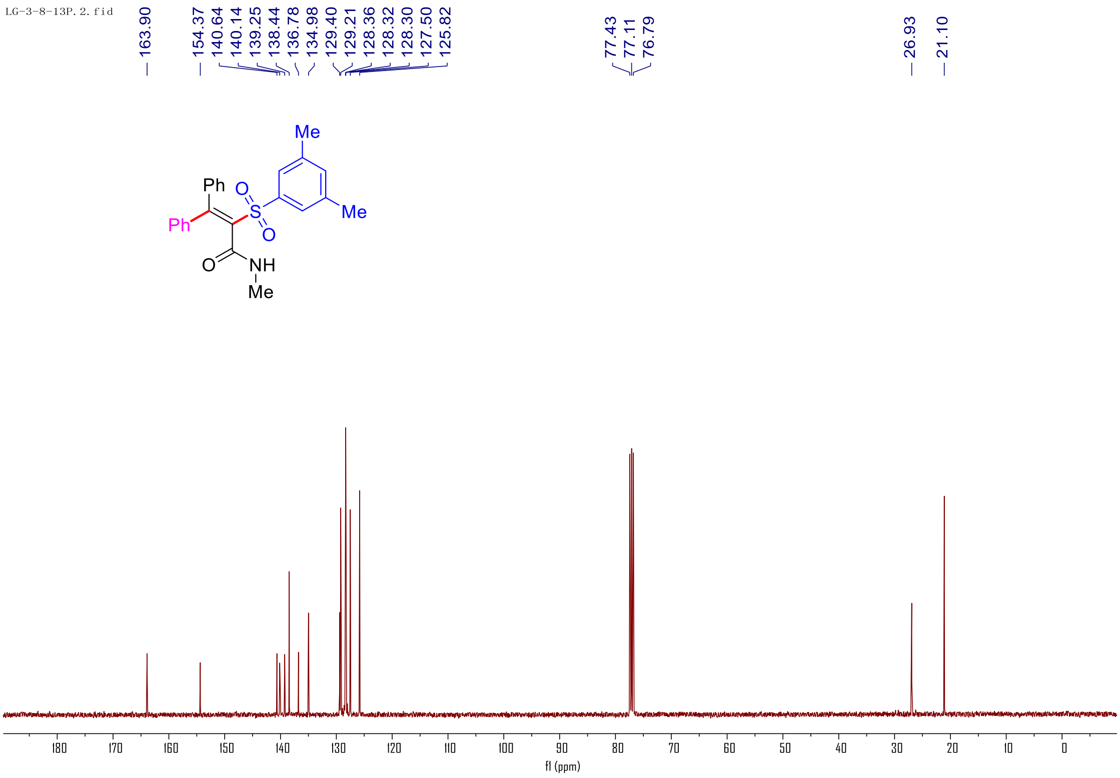

^{1}H NMR Spectrum of *N*-methyl-2-(naphthalen-1-ylsulfonyl)-3,3-diphenylacrylamide (56, $CDCl_3$ as solvent, 400 MHz)

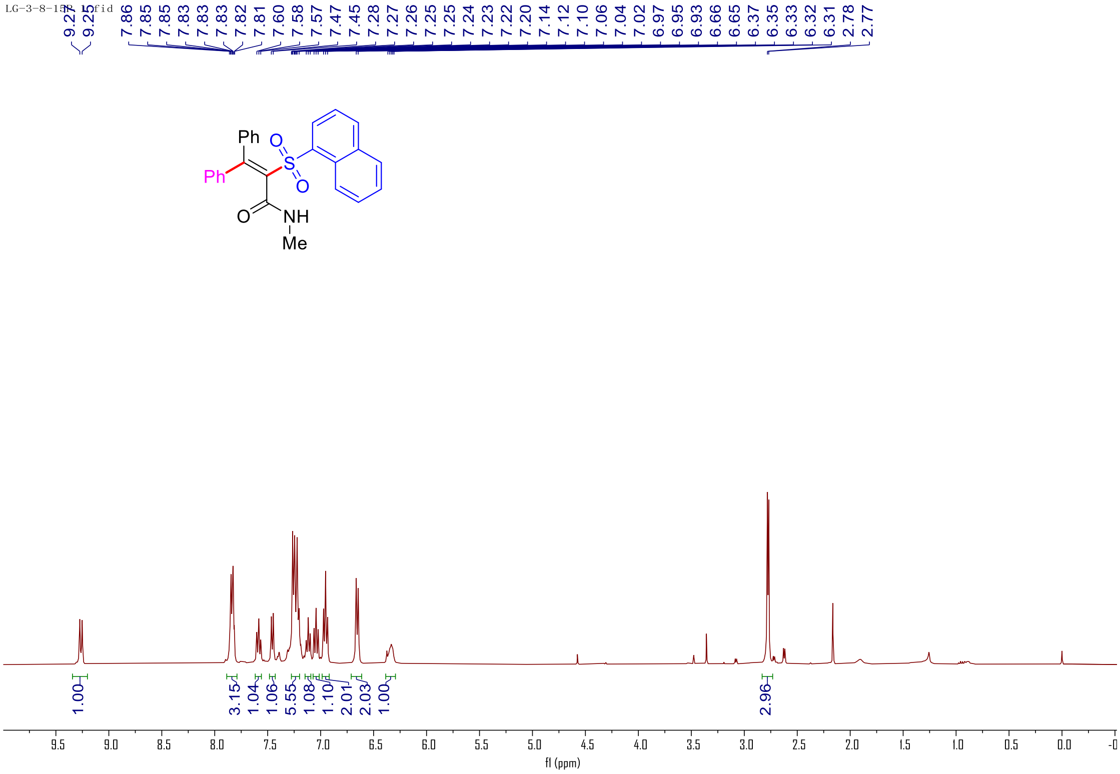

^{13}C {^{1}H} NMR Spectrum of *N*-methyl-2-(naphthalen-1-ylsulfonyl)-3,3-diphenylacrylamide (56, $CDCl_3$ as solvent, 101 MHz)

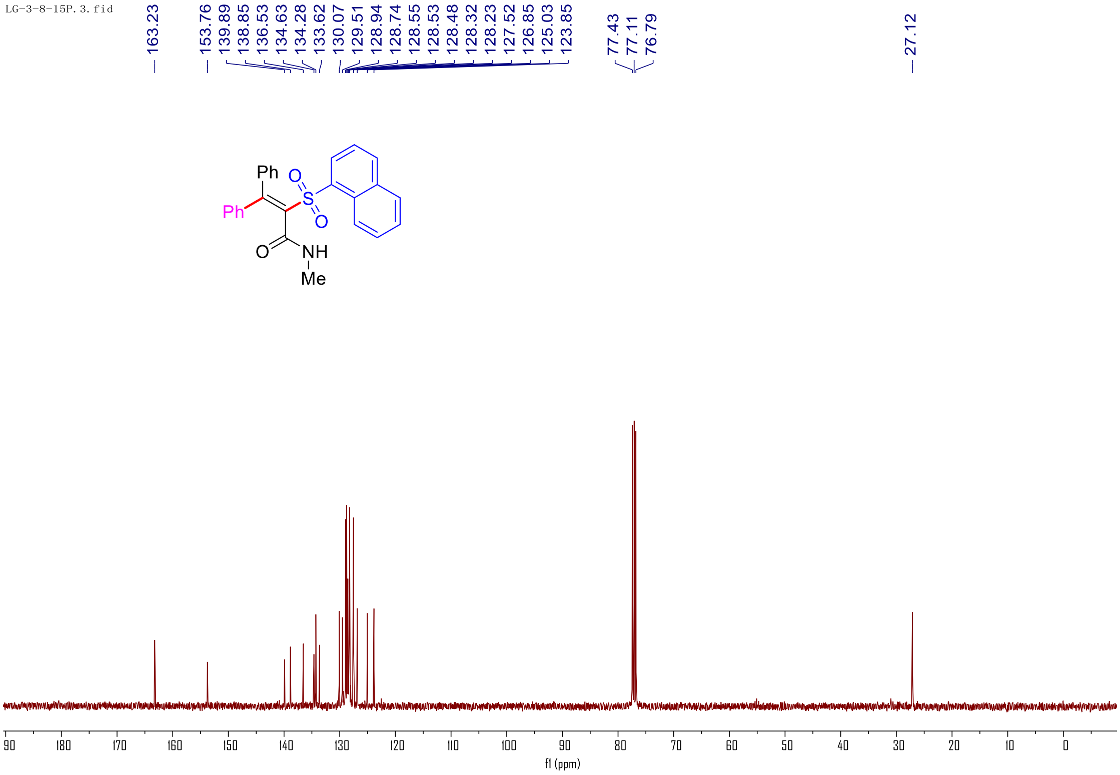

^{1}H NMR Spectrum of *N*-methyl-2-(naphthalen-2-ylsulfonyl)-3,3-diphenylacrylamide (57, $CDCl_3$ as solvent, 400 MHz)

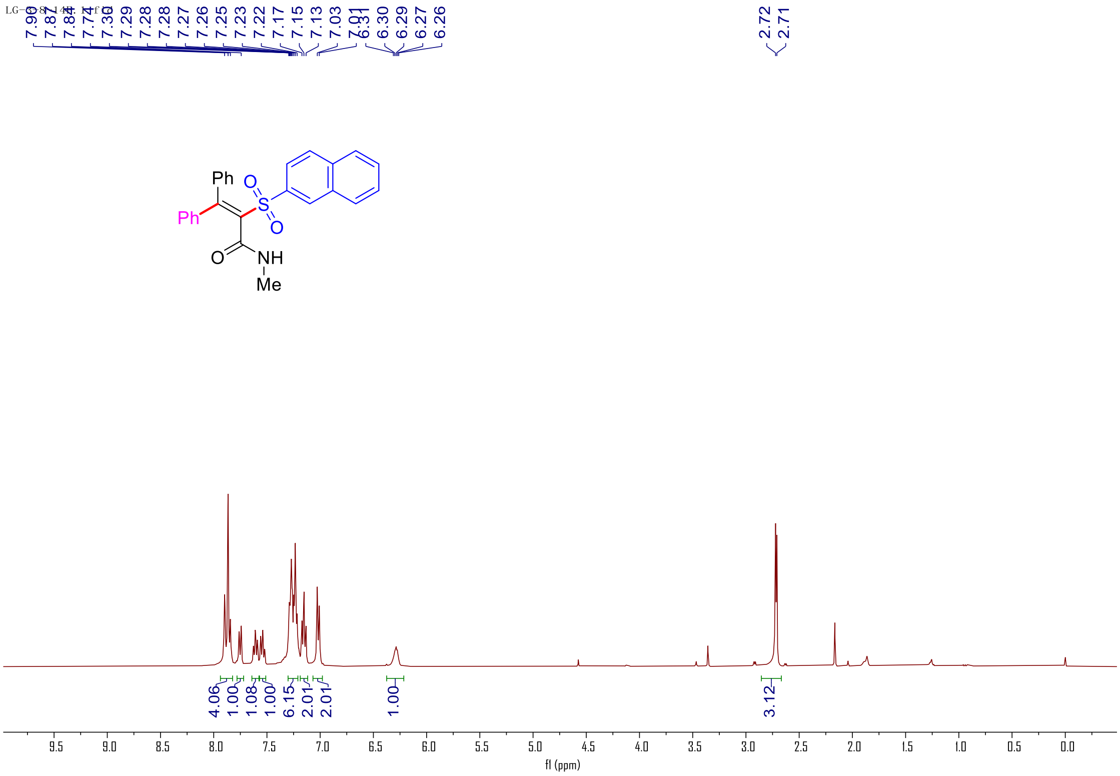

^{13}C {^{1}H} NMR Spectrum of *N*-methyl-2-(naphthalen-2-ylsulfonyl)-3,3-diphenylacrylamide (57, $CDCl_3$ as solvent, 101 MHz)

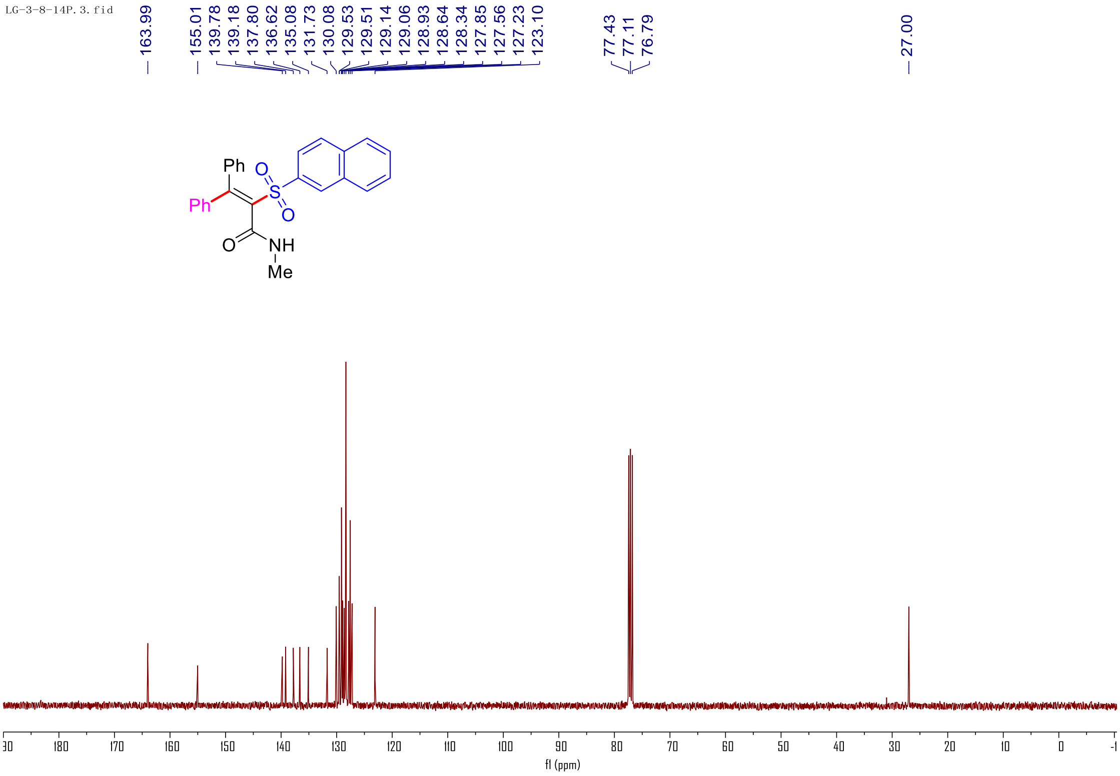

^{1}H NMR Spectrum of *N*-methyl-3,3-diphenyl-2-(pyridin-3-ylsulfonyl)acrylamide (58, $CDCl_3$ as solvent, 400 MHz)

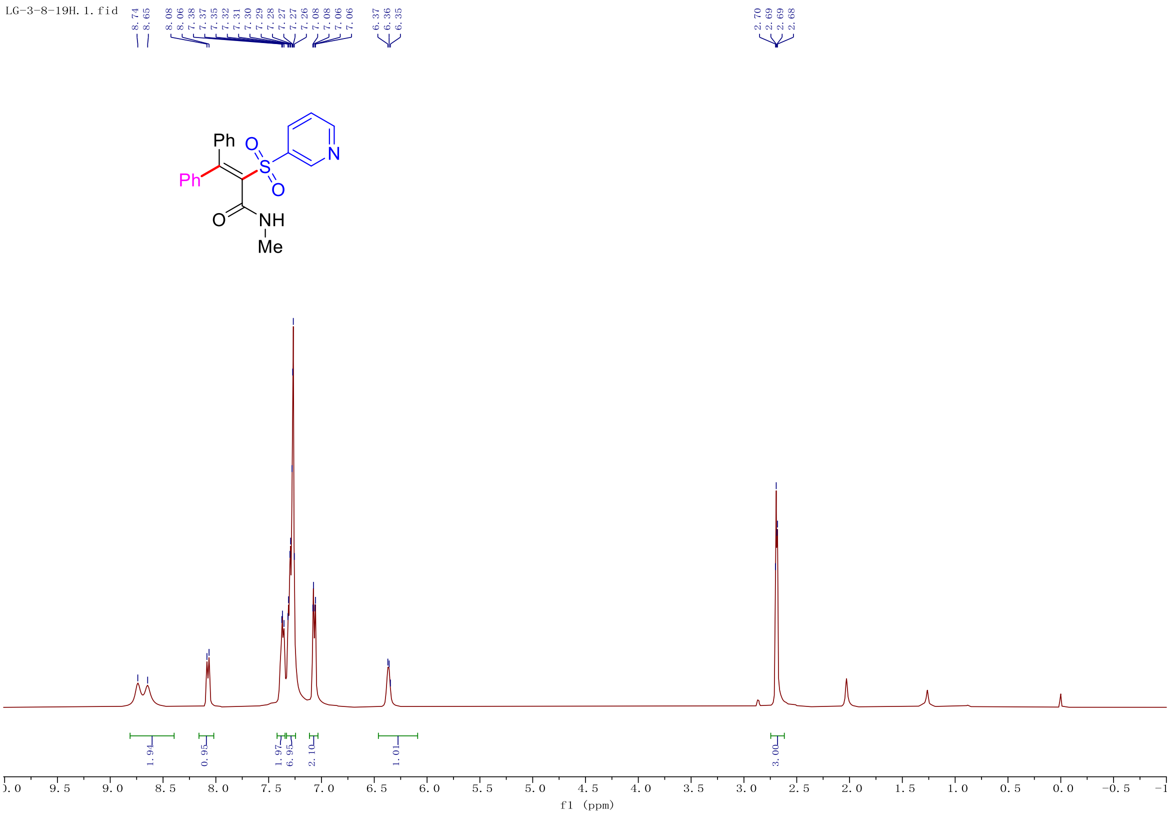

^{13}C {^{1}H} NMR Spectrum of *N*-methyl-3,3-diphenyl-2-(pyridin-3-ylsulfonyl)acrylamide (58, $CDCl_3$ as solvent, 101 MHz)

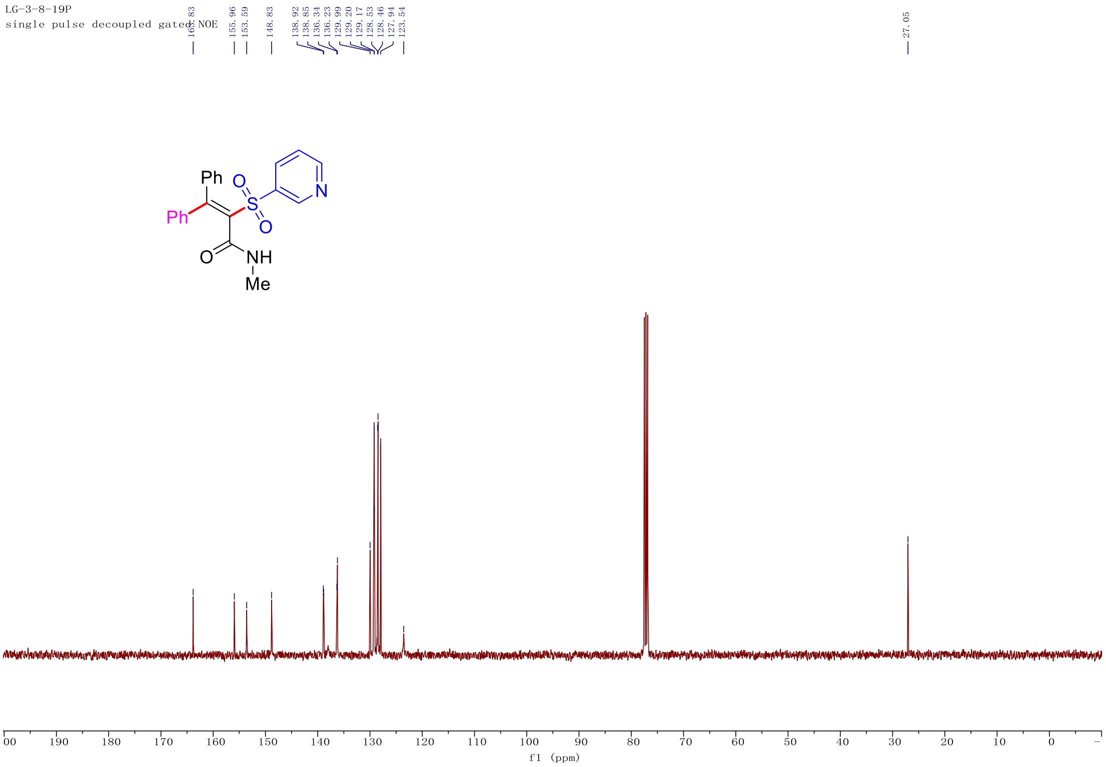

^{1}H NMR Spectrum of *N*-methyl-3,3-diphenyl-2-(quinolin-8-ylsulfonyl)acrylamide (59, $CDCl_3$ as solvent, 400 MHz)

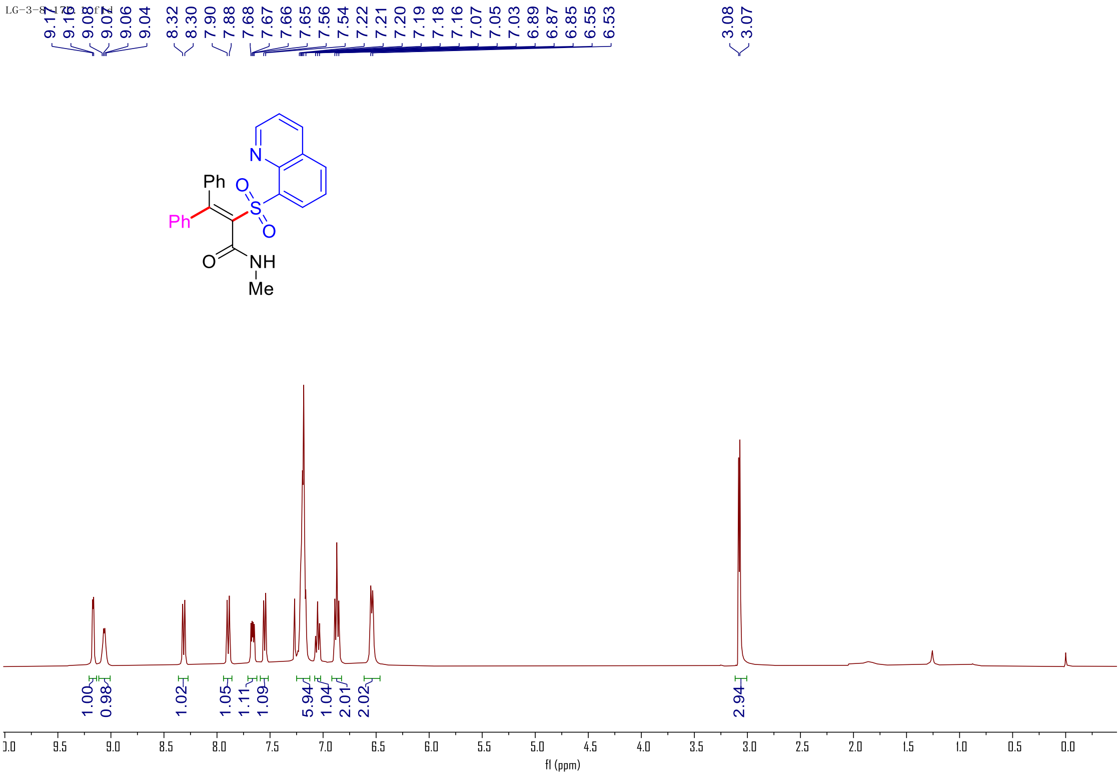

^{13}C {^{1}H} NMR Spectrum of *N*-methyl-3,3-diphenyl-2-(quinolin-8-ylsulfonyl)acrylamide (59, $CDCl_3$ as solvent, 101 MHz)

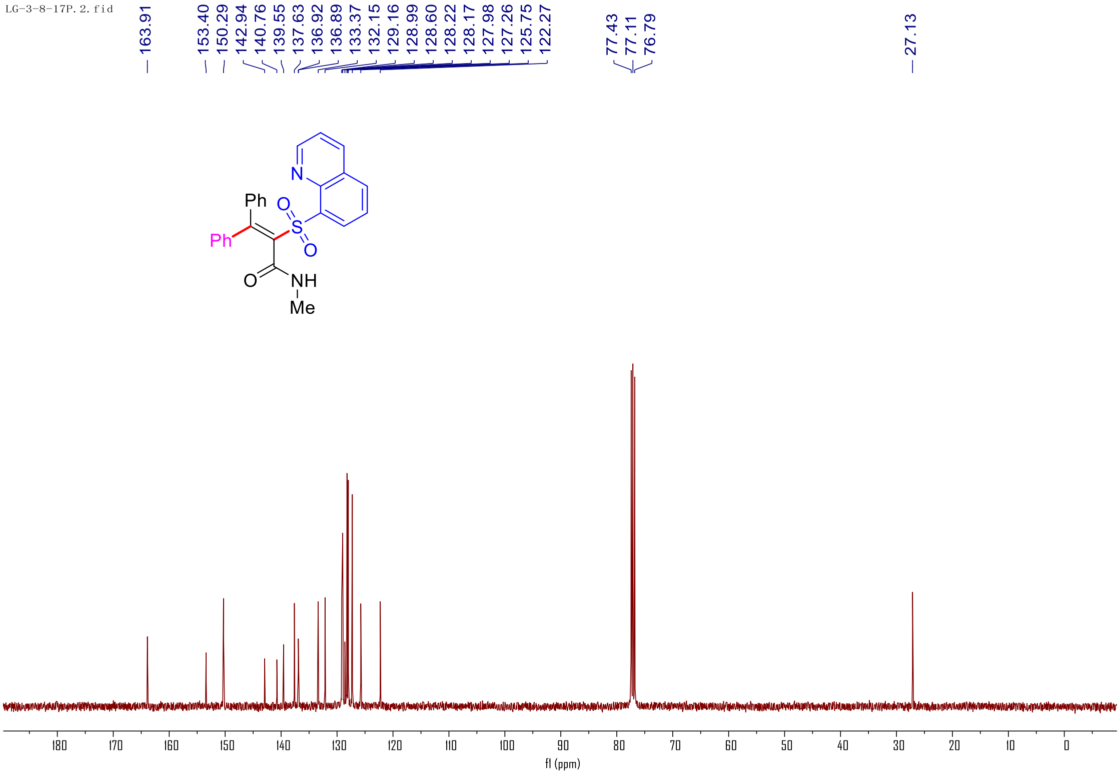

^{1}H NMR Spectrum of *N*-methyl-3,3-diphenyl-2-(thiophen-2-ylsulfonyl)acrylamide (60, $CDCl_3$ as solvent, 400 MHz)

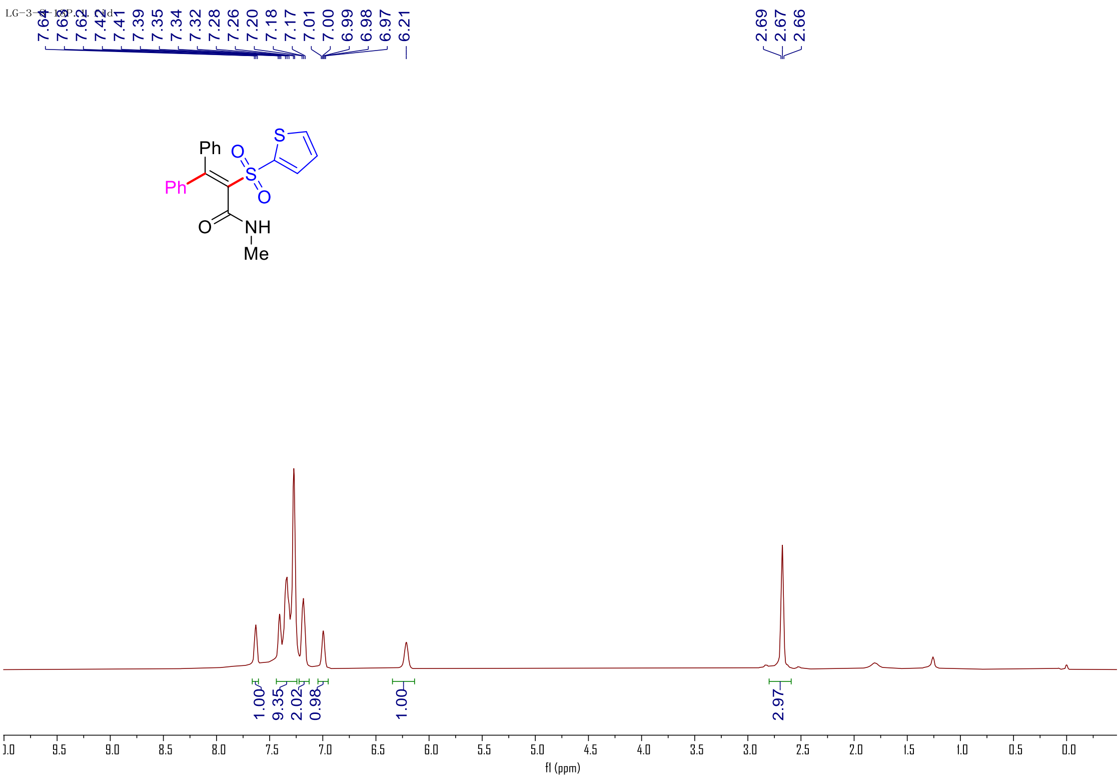

^{13}C {^{1}H} NMR Spectrum of *N*-methyl-3,3-diphenyl-2-(thiophen-2-ylsulfonyl)acrylamide (60, $CDCl_3$ as solvent, 101 MHz)

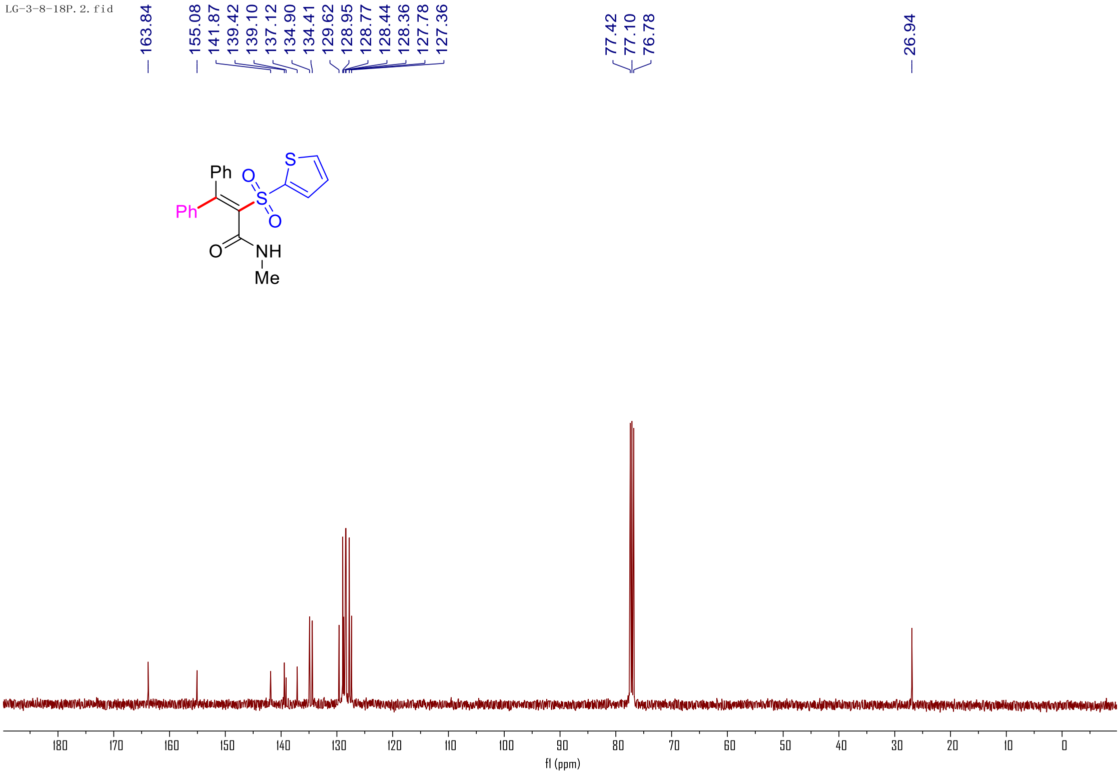

^{1}H NMR Spectrum of *N*-methyl-2-(methylsulfonyl)-3,3-diphenylacrylamide (61, $CDCl_3$ as solvent, 400 MHz)

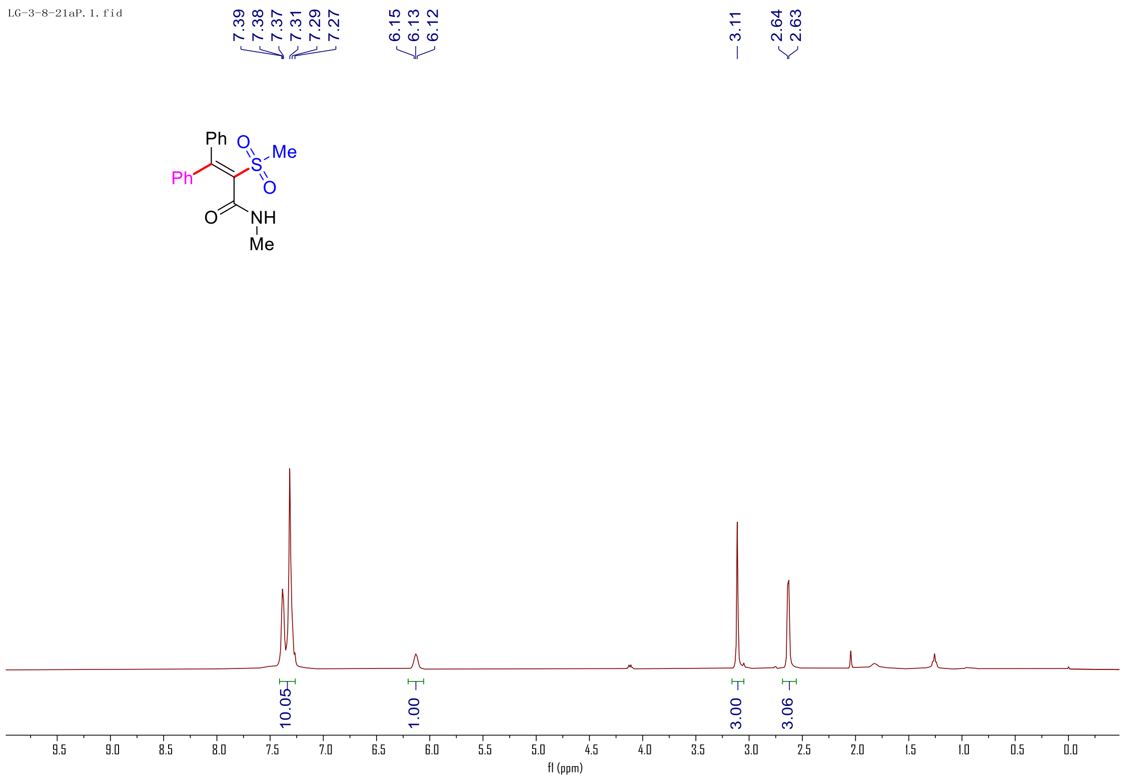

^{13}C {^{1}H} NMR Spectrum of *N*-methyl-2-(methylsulfonyl)-3,3-diphenylacrylamide (61, $CDCl_3$ as solvent, 101 MHz)

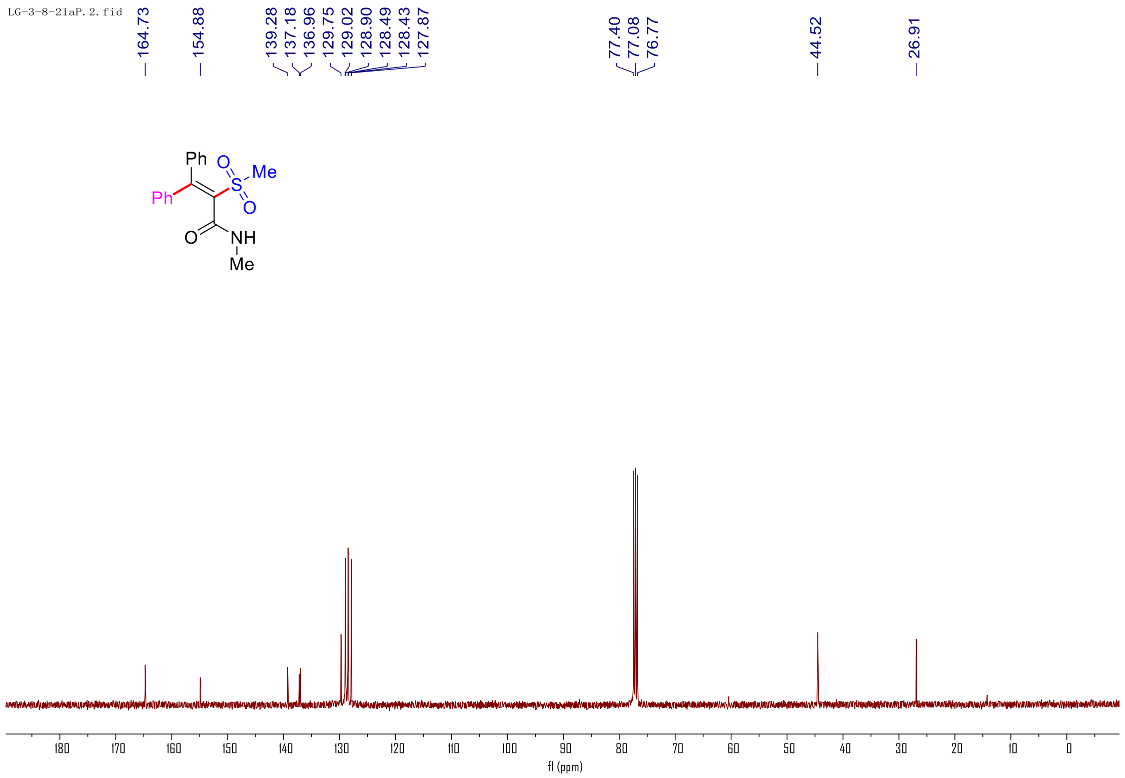

^{1}H NMR Spectrum of *N*-methyl-2-((4-(5-methyl-3-phenylisoxazol-4-yl)phenyl)sulfonyl)-3,3-diphenylacrylamide (62, $CDCl_3$ as solvent, 400 MHz)

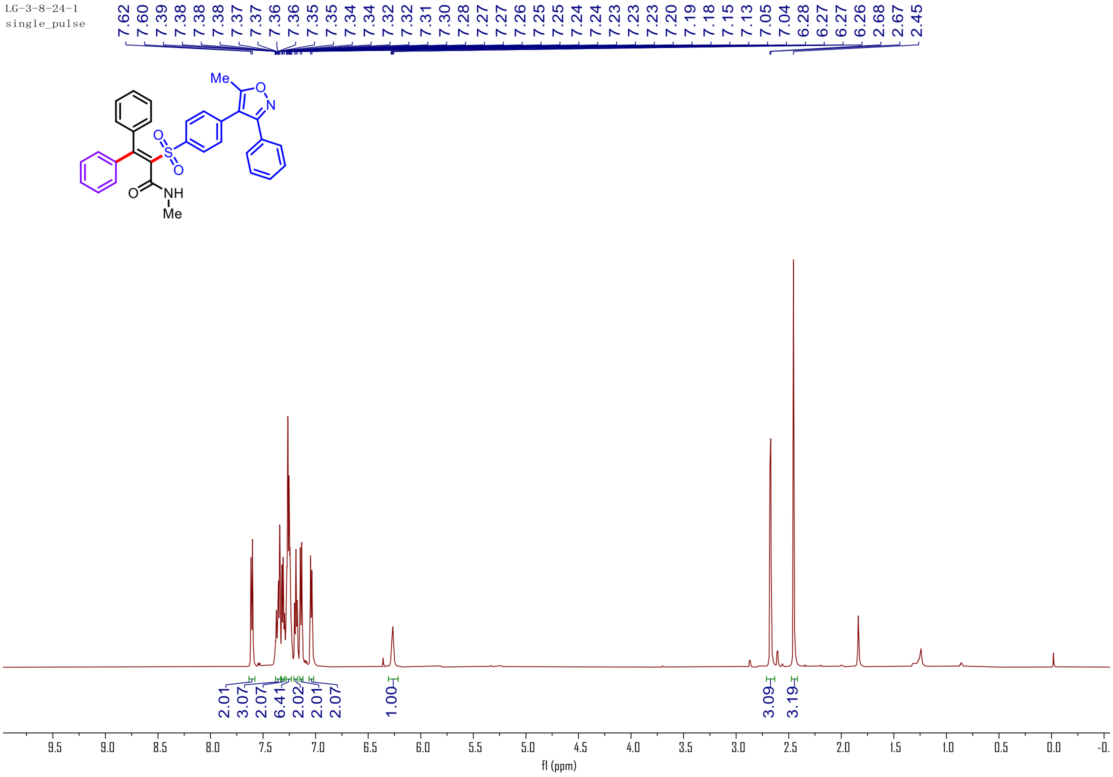

^{13}C {^{1}H} NMR Spectrum of *N*-methyl-2-((4-(5-methyl-3-phenylisoxazol-4-yl)phenyl)sulfonyl)-3,3-diphenylacrylamide (62, $CDCl_3$ as solvent, 101 MHz)

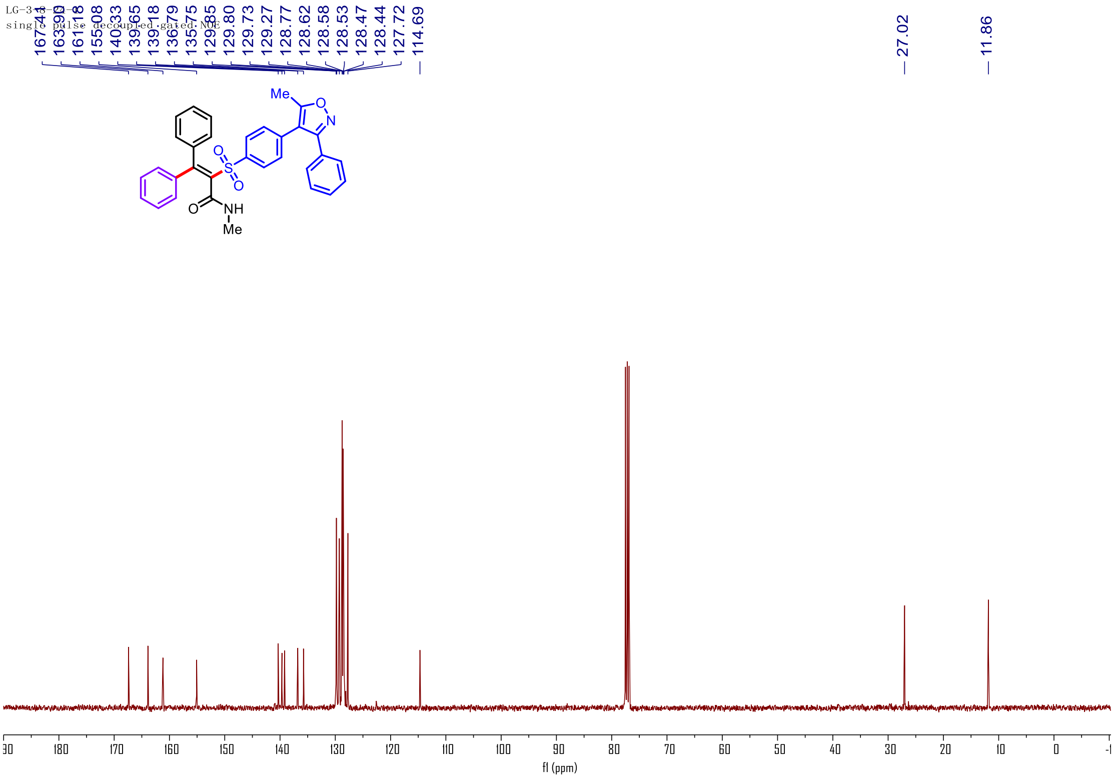

^{1}H NMR Spectrum of 2-((4-ethoxy-3-(1-methyl-7-oxo-3-propyl-6,7-dihydro-1H-pyrazolo[4,3-d]pyrimidin-5-yl)phenyl)sulfonyl)-*N*-methyl-3,3-diphenylacrylamide (63, $CDCl_3$ as solvent, 400 MHz)

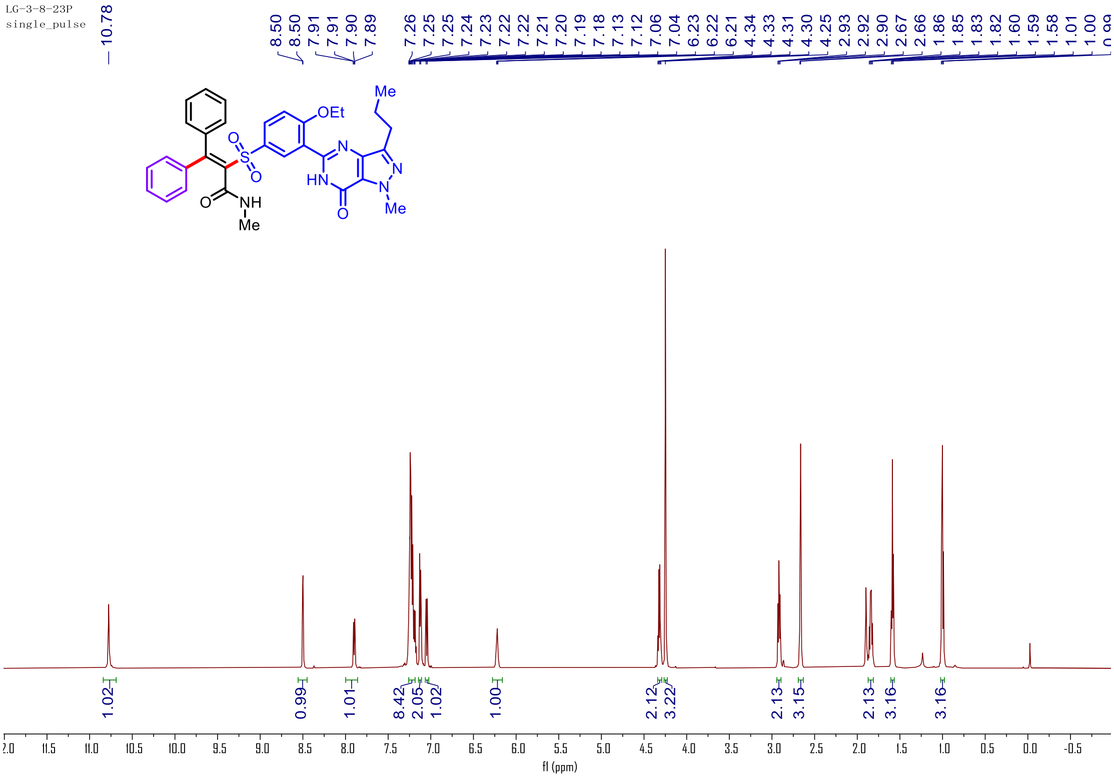

^{13}C {^{1}H} NMR Spectrum of 2-((4-ethoxy-3-(1-methyl-7-oxo-3-propyl-6,7-dihydro-1H-pyrazolo[4,3-d]pyrimidin-5-yl)phenyl)sulfonyl)-*N*-methyl-3,3-diphenylacrylamide (63, $CDCl_3$ as solvent, 101 MHz)

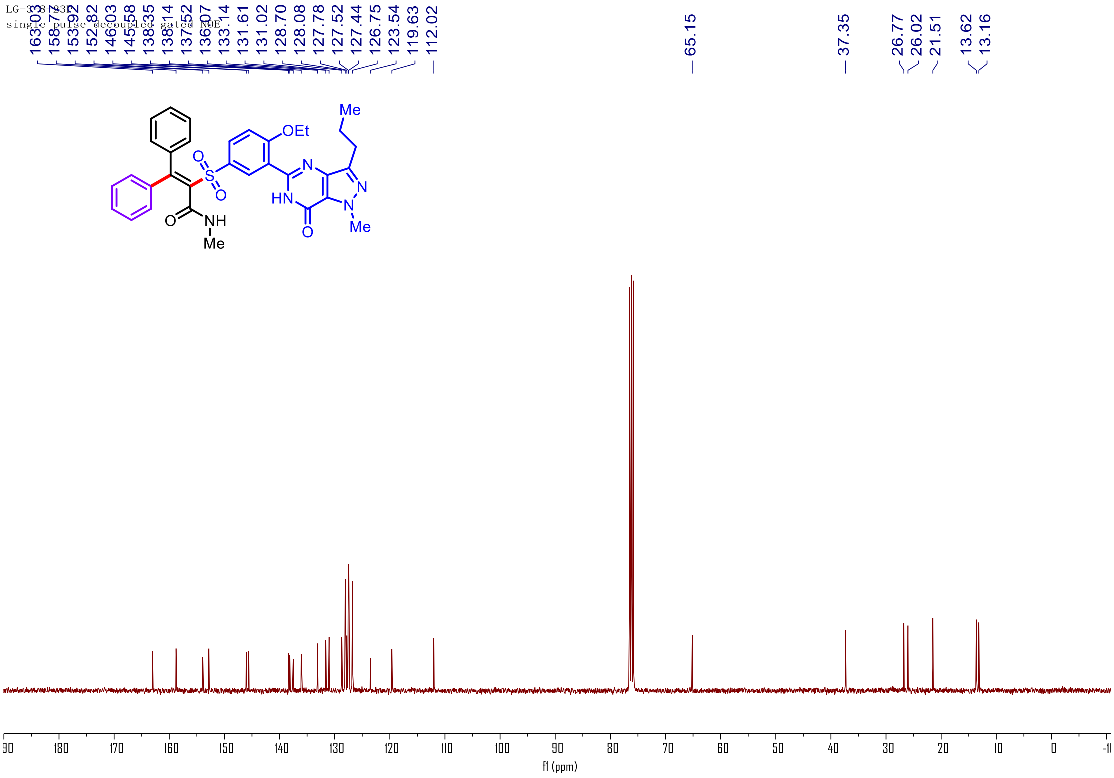

^{1}H NMR Spectrum of *N*-ethyl-3,3-diphenylacrylamide (64, $CDCl_3$ as solvent, 400 MHz)

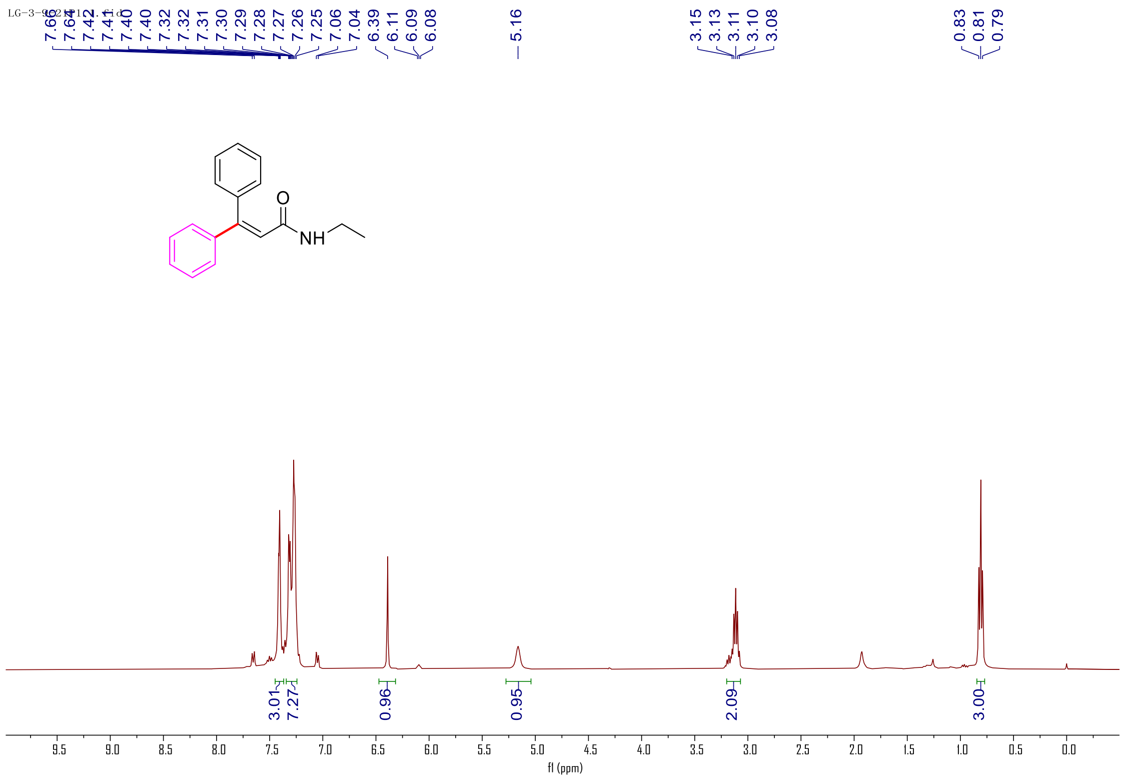

^{13}C {^{1}H} NMR Spectrum of *N*-ethyl-3,3-diphenylacrylamide (64, $CDCl_3$ as solvent, 101 MHz)

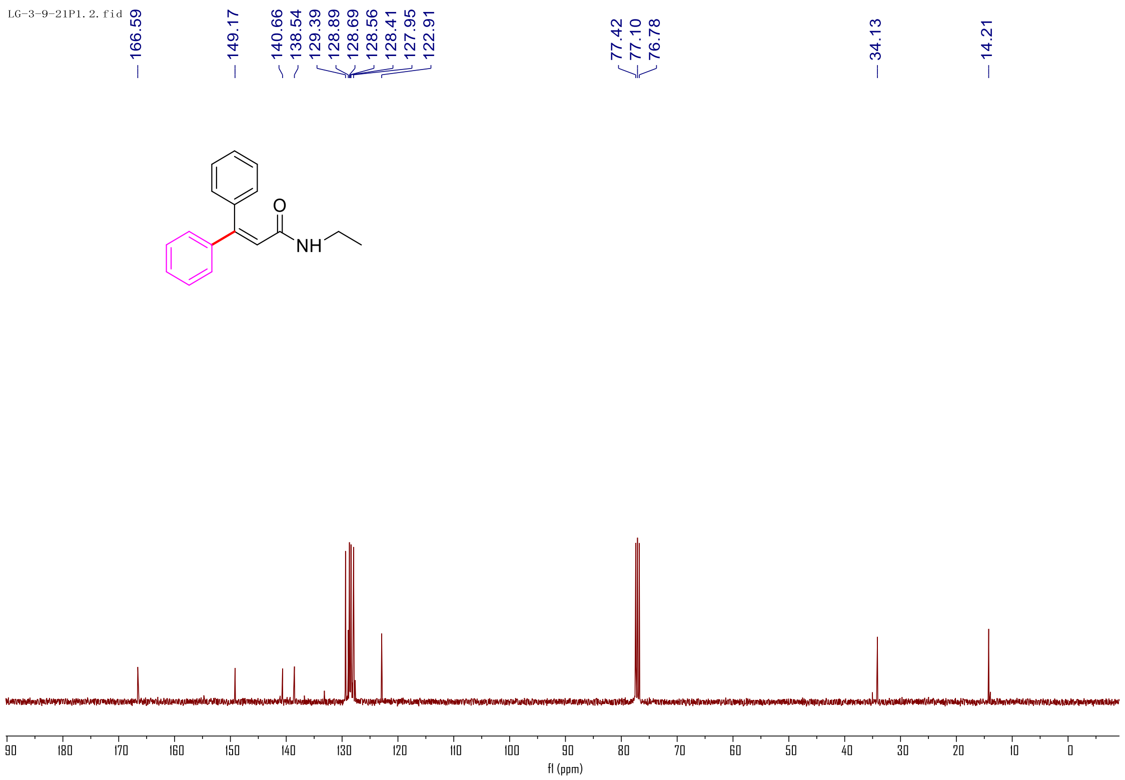

^{1}H NMR Spectrum of *N*-benzyl-3,3-diphenylacrylamide (65, $CDCl_3$ as solvent, 400 MHz)

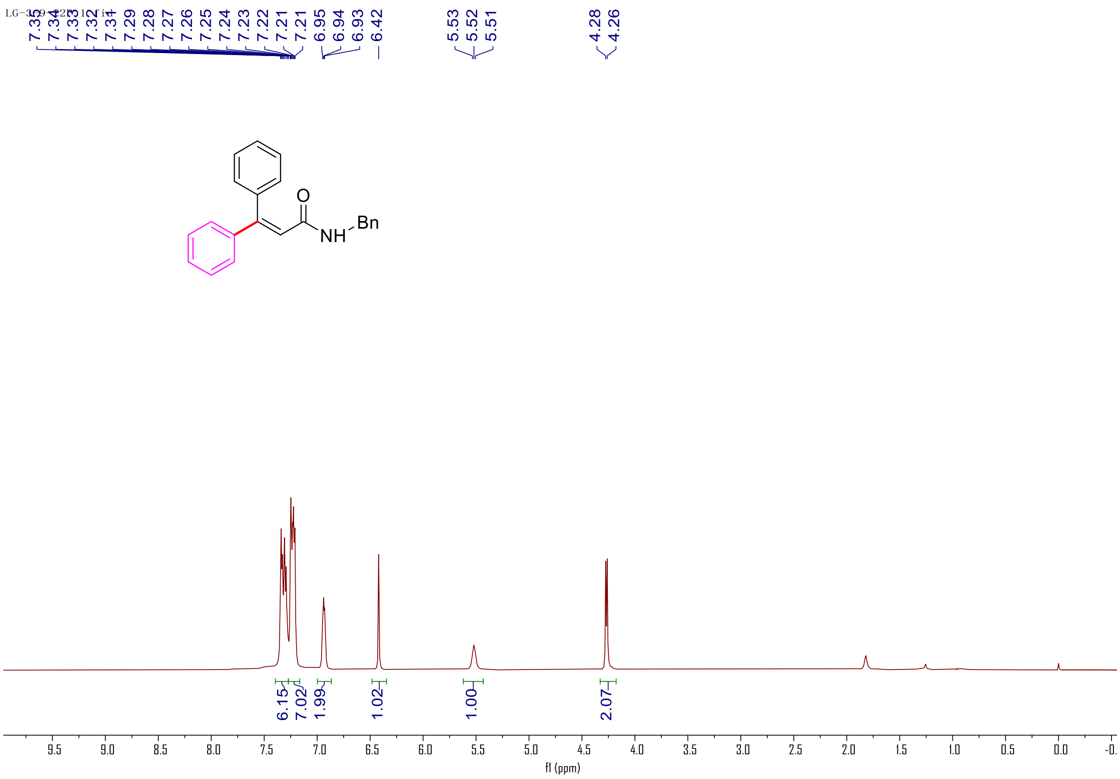

^{13}C {^{1}H} NMR Spectrum of *N*-benzyl-3,3-diphenylacrylamide (65, $CDCl_3$ as solvent, 101 MHz)

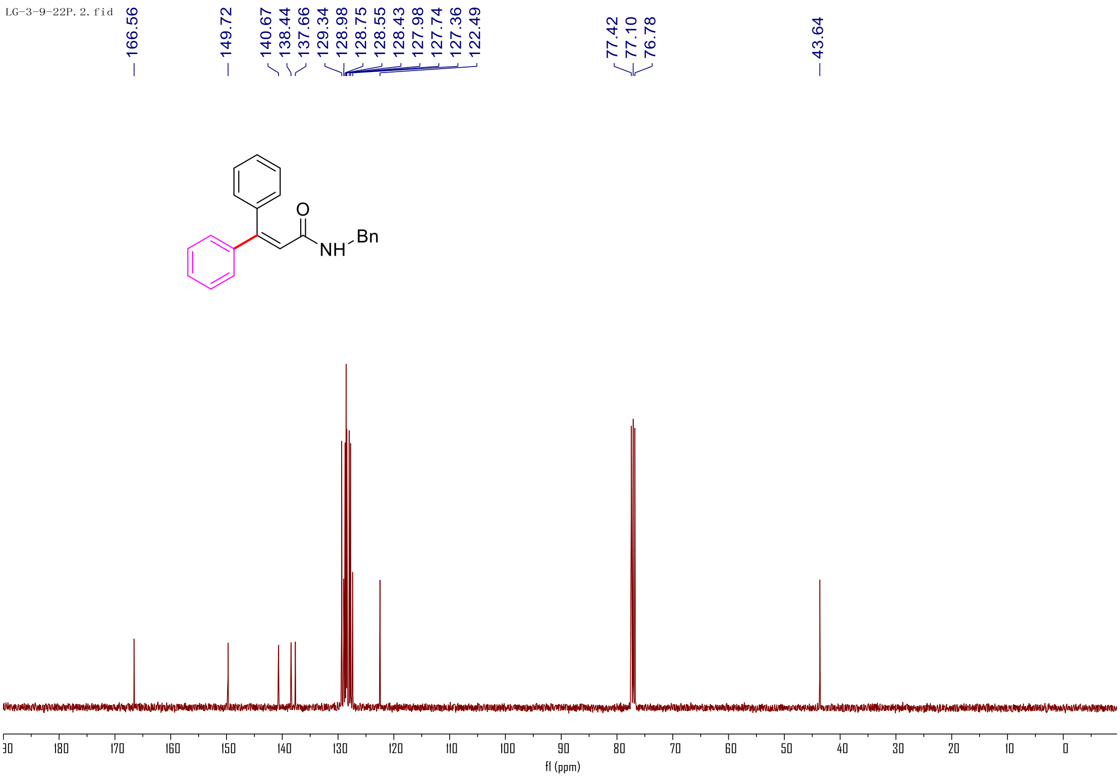

^{1}H NMR Spectrum of 3,3-diphenyl-*N*-(1-phenylethyl)acrylamide (66, $CDCl_3$ as solvent, 400 MHz)

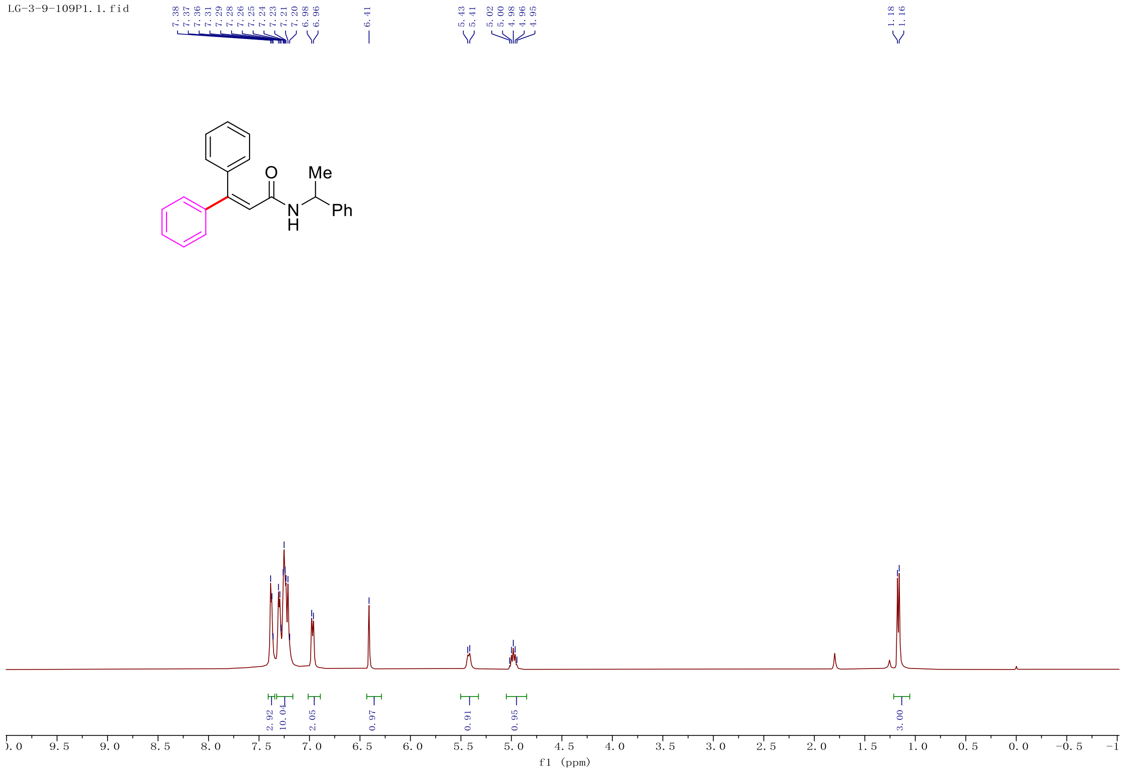

^{13}C {^{1}H} NMR Spectrum of 3,3-diphenyl-*N*-(1-phenylethyl)acrylamide (66, $CDCl_3$ as solvent, 101 MHz)

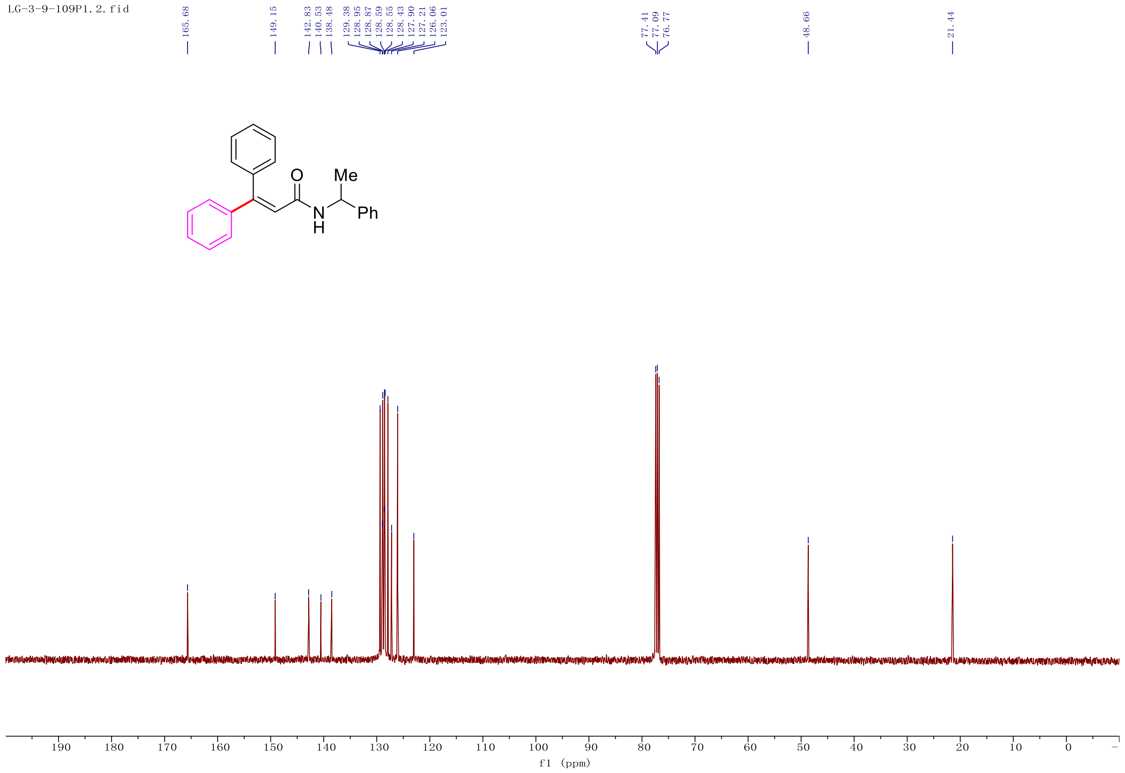

^{1}H NMR Spectrum of *N*-methyl-3-phenyl-3-(p-tolyl)acrylamide (67, $CDCl_3$ as solvent, 400 MHz)

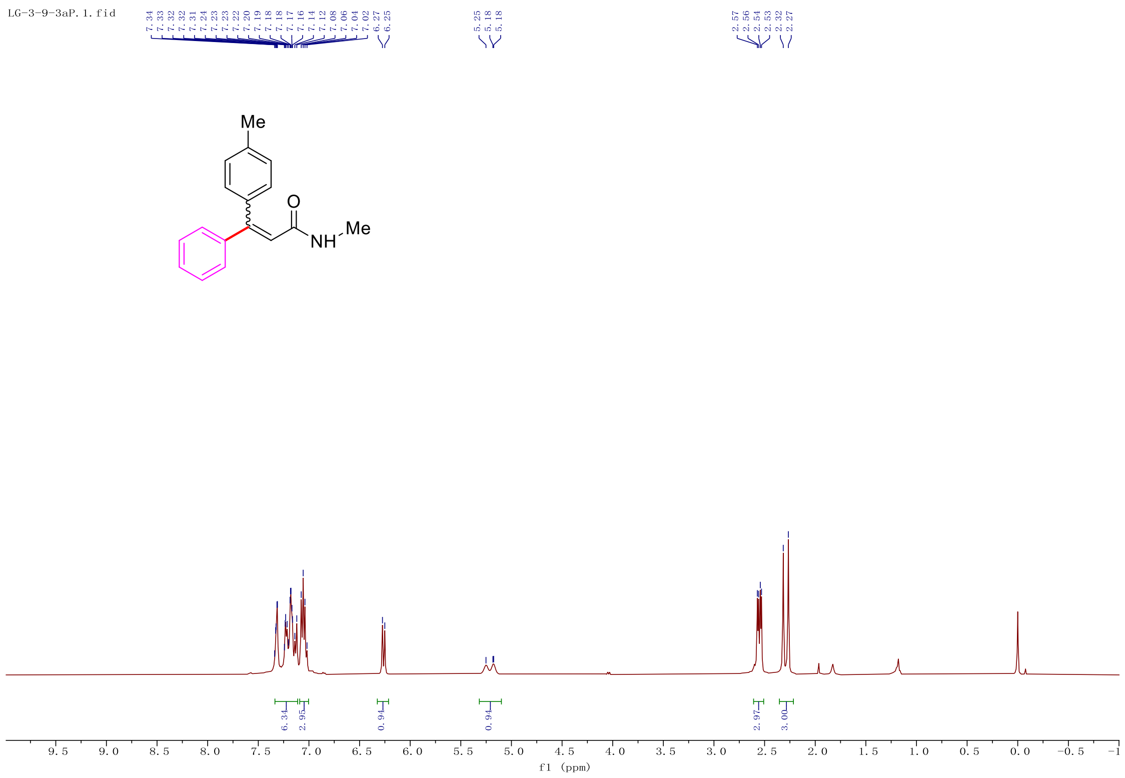

^{13}C {^{1}H} NMR Spectrum of *N*-methyl-3-phenyl-3-(p-tolyl)acrylamide (67, $CDCl_3$ as solvent, 101 MHz)

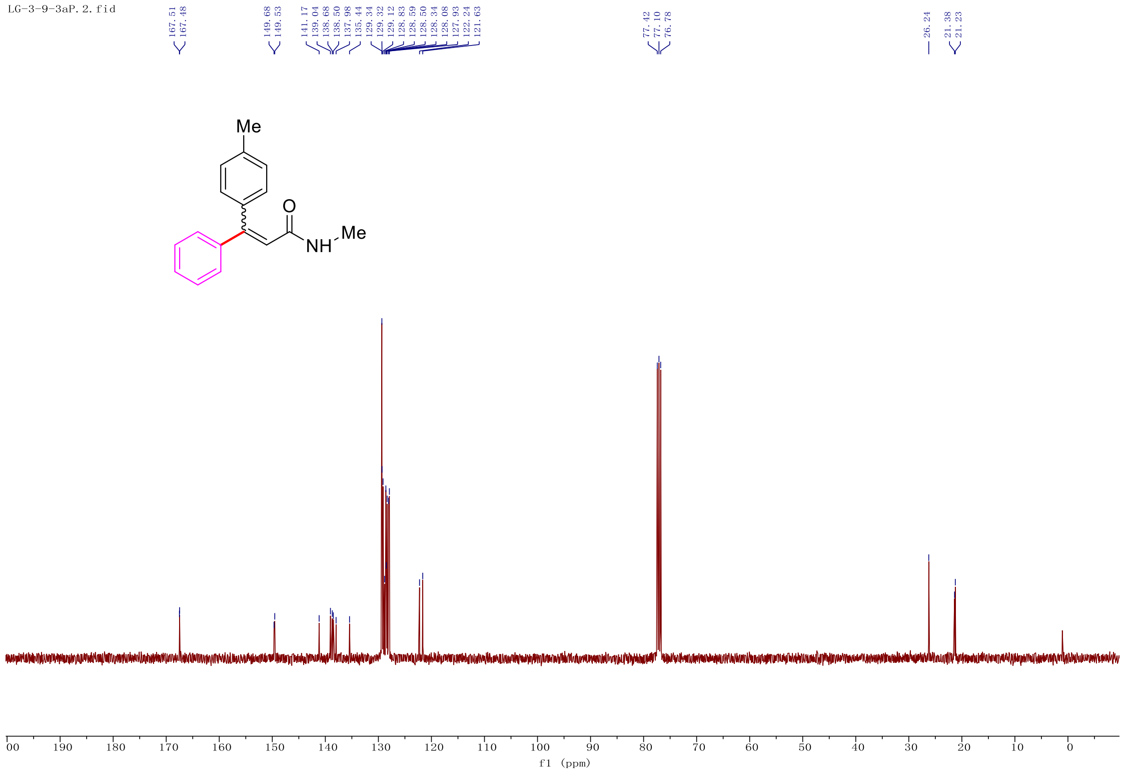

^{1}H NMR Spectrum of 3-(4-methoxyphenyl)-N-methyl-3-phenylacrylamide (68, $CDCl_3$ as solvent, 400 MHz)

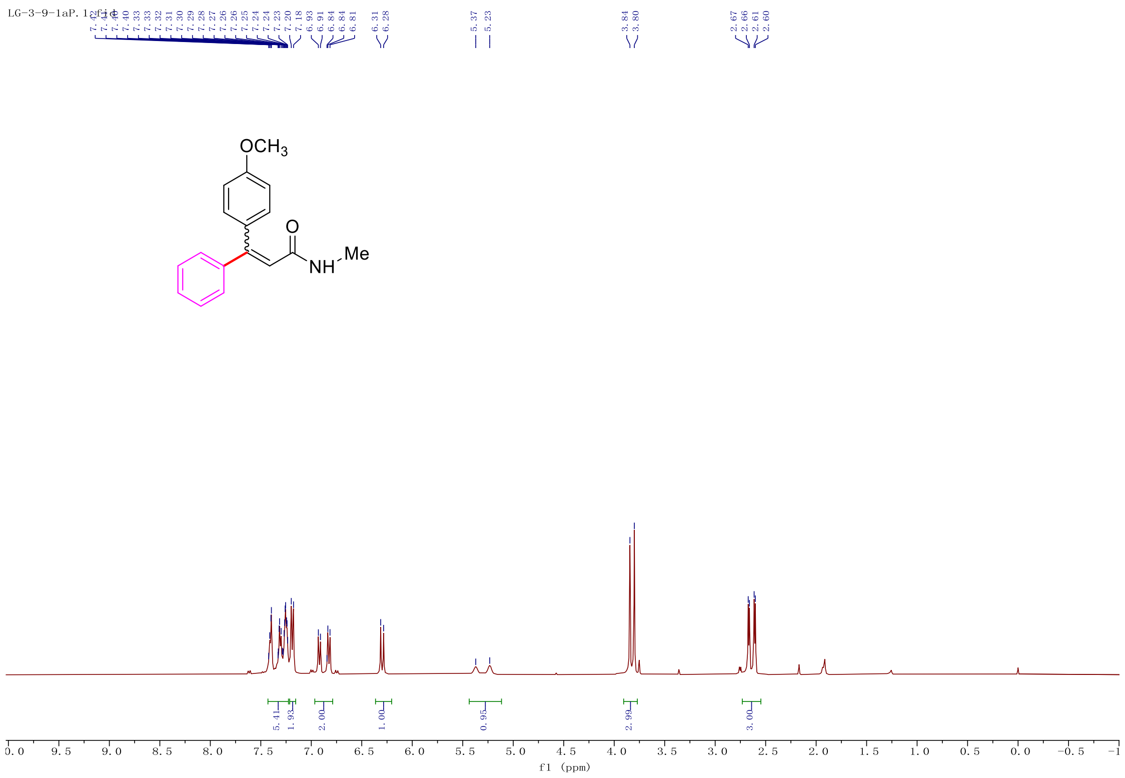

^{13}C {^{1}H} NMR Spectrum of 3-(4-methoxyphenyl)-N-methyl-3-phenylacrylamide (68, $CDCl_3$ as solvent, 101 MHz)

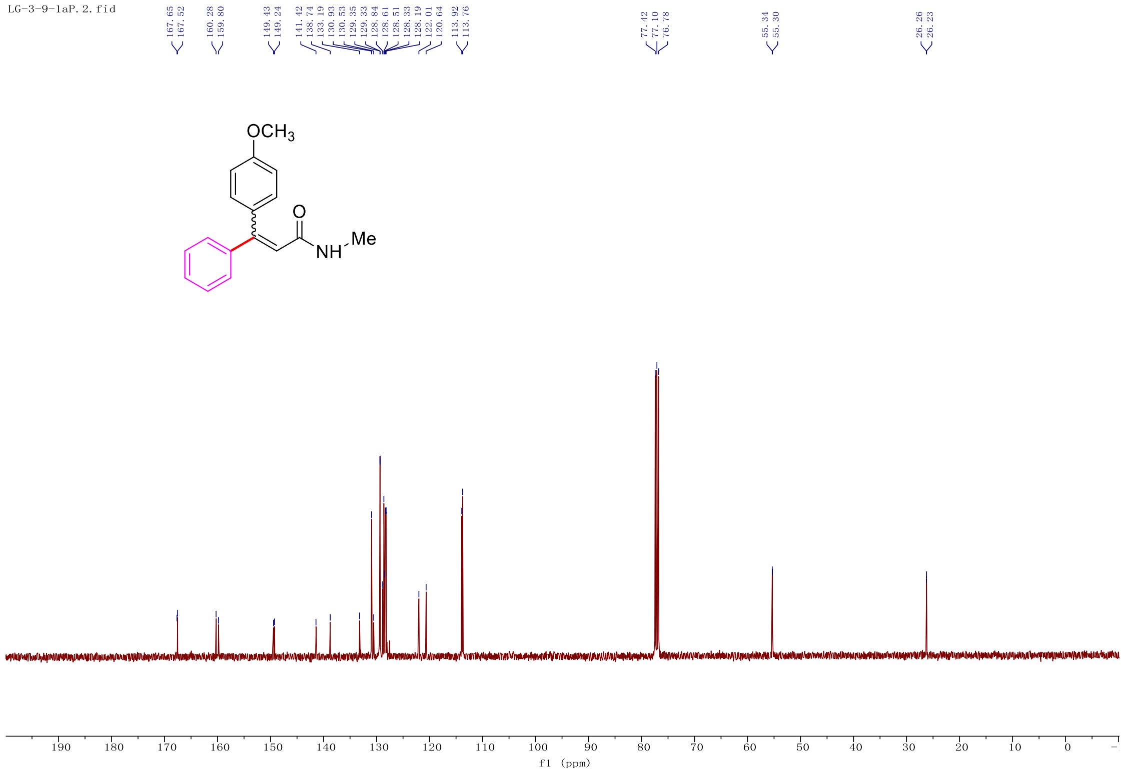

^{1}H NMR Spectrum of *N*-methyl-3-phenyl-3-(4-(trifluoromethoxy)phenyl)acrylamide (69, $CDCl_3$ as solvent, 400 MHz)

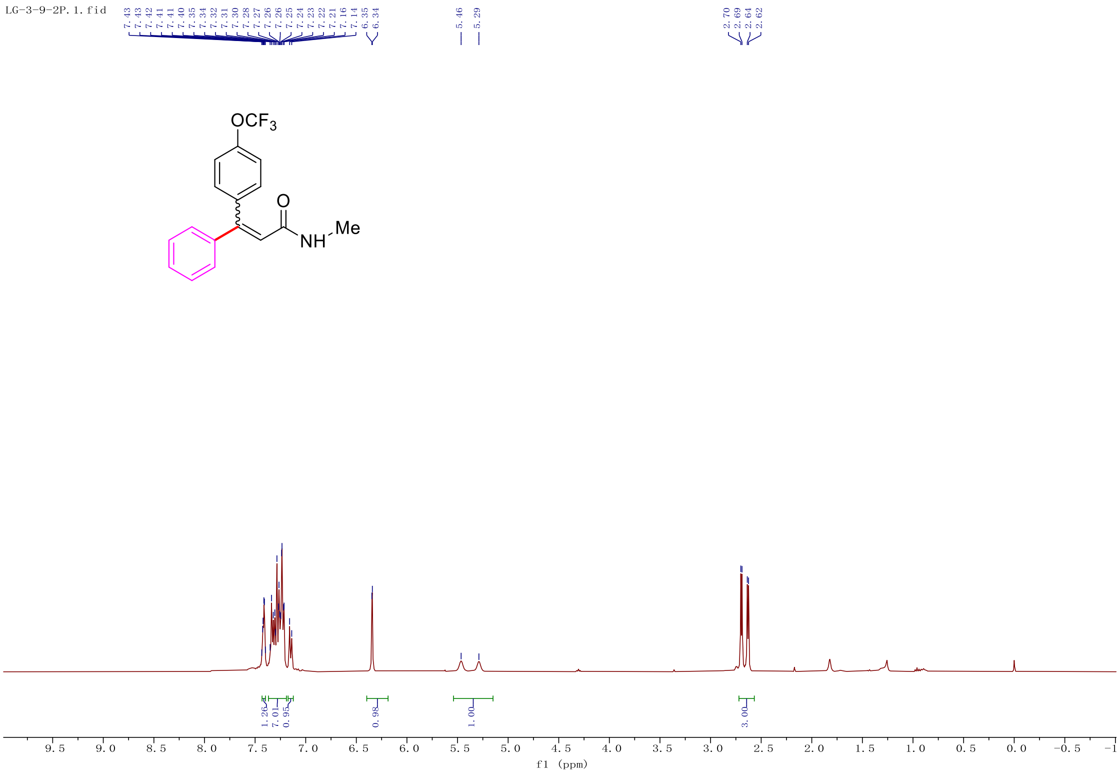

^{13}C {^{1}H} NMR Spectrum of *N*-methyl-3-phenyl-3-(4-(trifluoromethoxy)phenyl)acrylamide (69, $CDCl_3$ as solvent, 101 MHz)

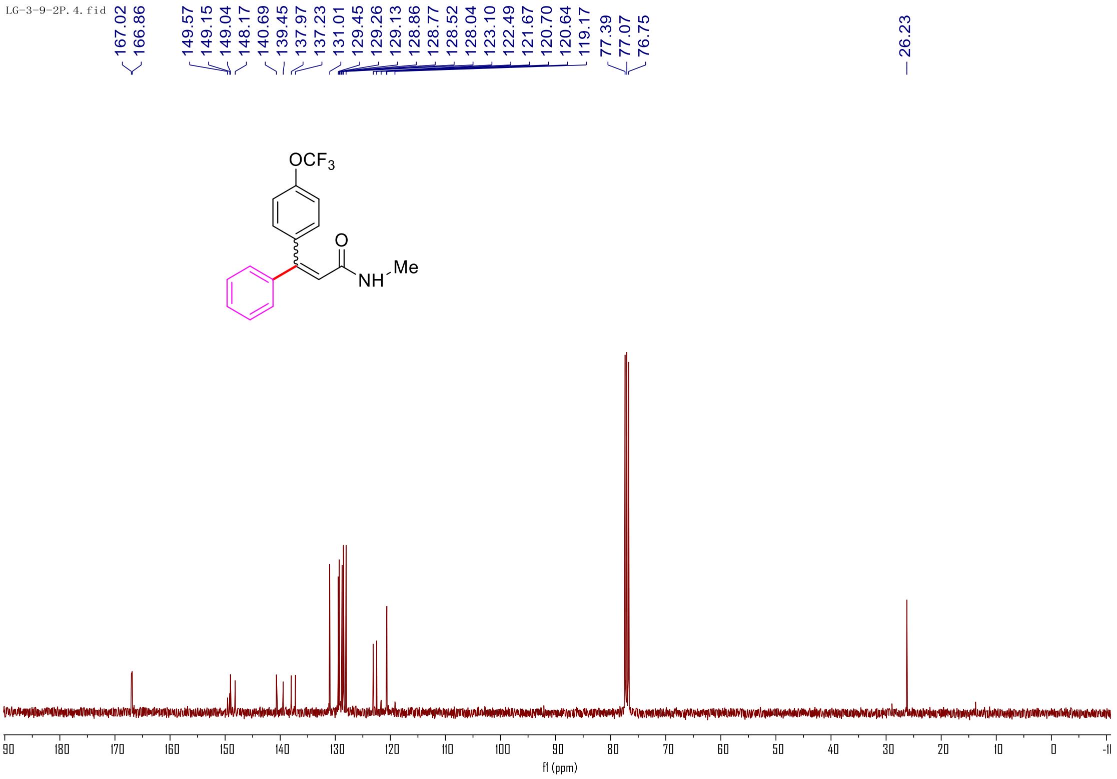

^{19}F NMR Spectrum of *N*-methyl-3-phenyl-3-(4-(trifluoromethoxy)phenyl)acrylamide (69, $CDCl_3$ as solvent, 376 MHz)

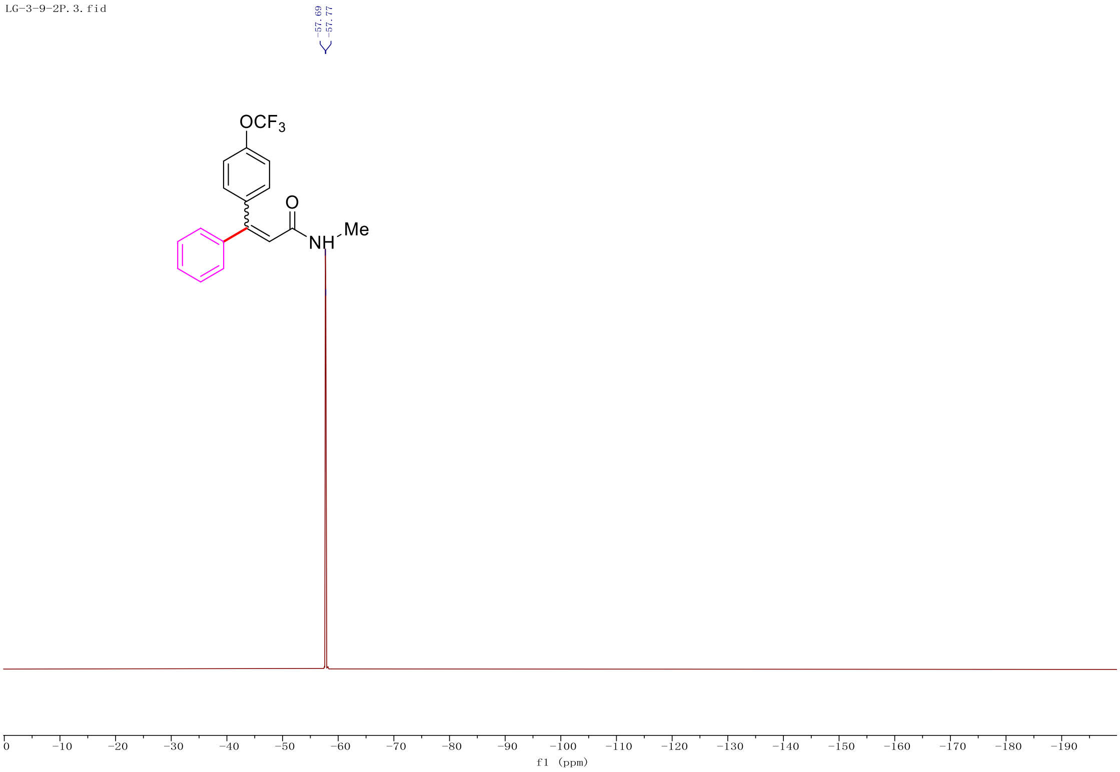

^{1}H NMR Spectrum of (*Z*)-*N*-methyl-3-phenyl-3-(4-(trifluoromethyl)phenyl)acrylamide (*Z*-70, $CDCl_3$ as solvent, 400 MHz)

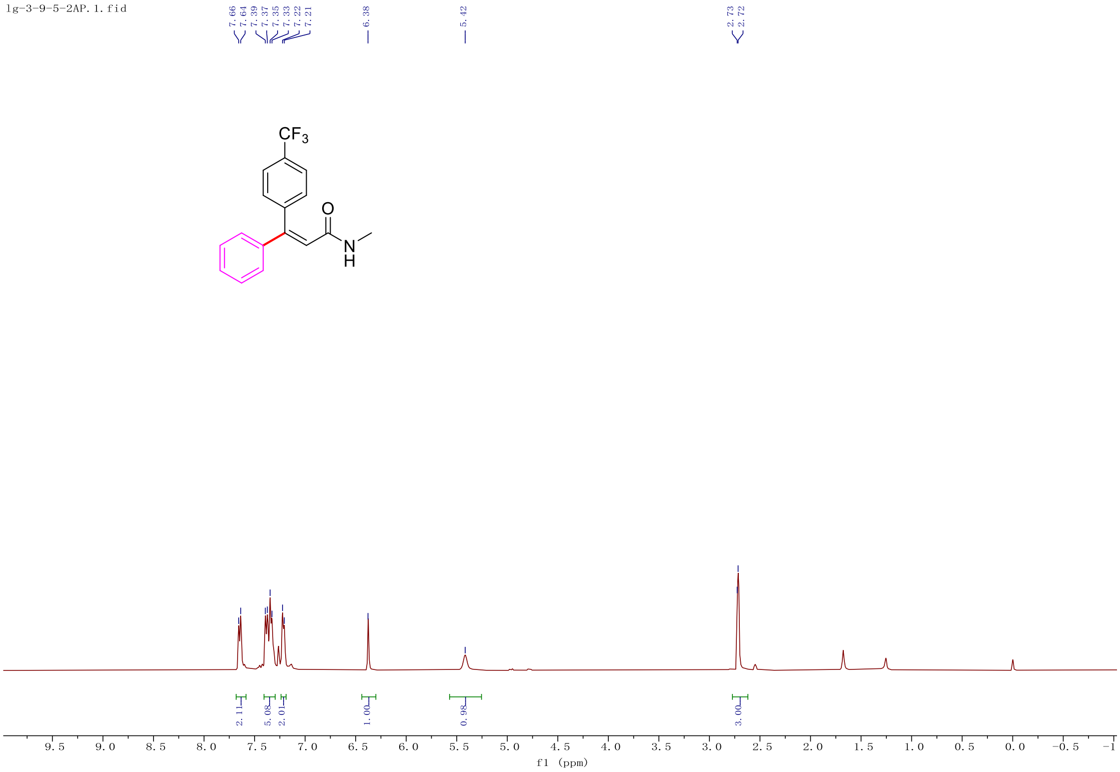

^{13}C {^{1}H} NMR Spectrum of (*Z*)-*N*-methyl-3-phenyl-3-(4-(trifluoromethyl)phenyl)acrylamide (*Z*-70, $CDCl_3$ as solvent, 101 MHz)

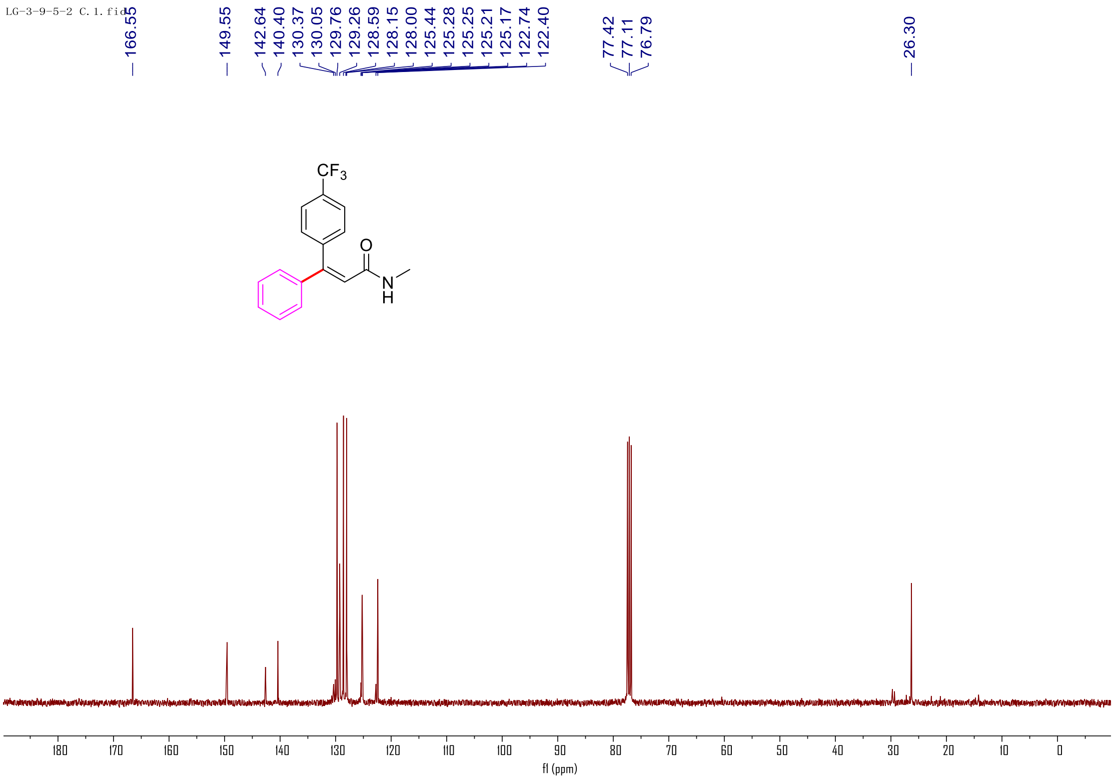

^{19}F NMR Spectrum of (*Z*)-*N*-methyl-3-phenyl-3-(4-(trifluoromethyl)phenyl)acrylamide (*Z*-70, $CDCl_3$ as solvent, 376 MHz)

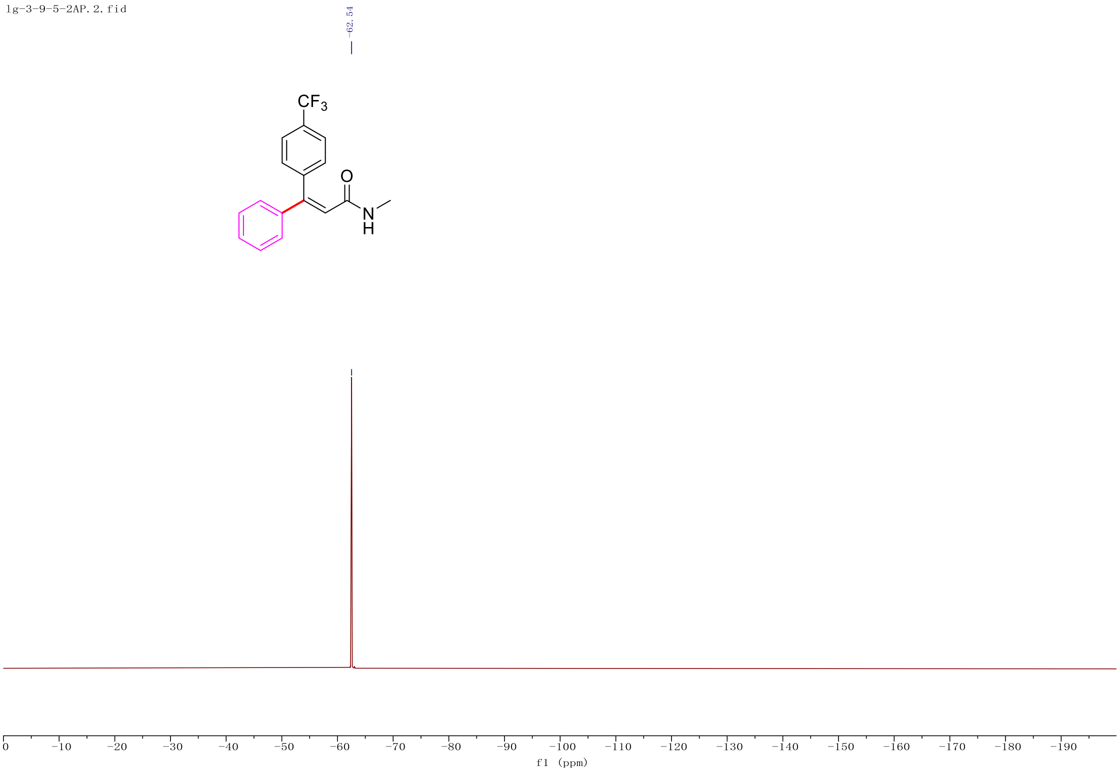

^{1}H NMR Spectrum of (*E*)-*N*-methyl-3-phenyl-3-(4-(trifluoromethyl)phenyl)acrylamide (*E*-70, $CDCl_3$ as solvent, 400 MHz)

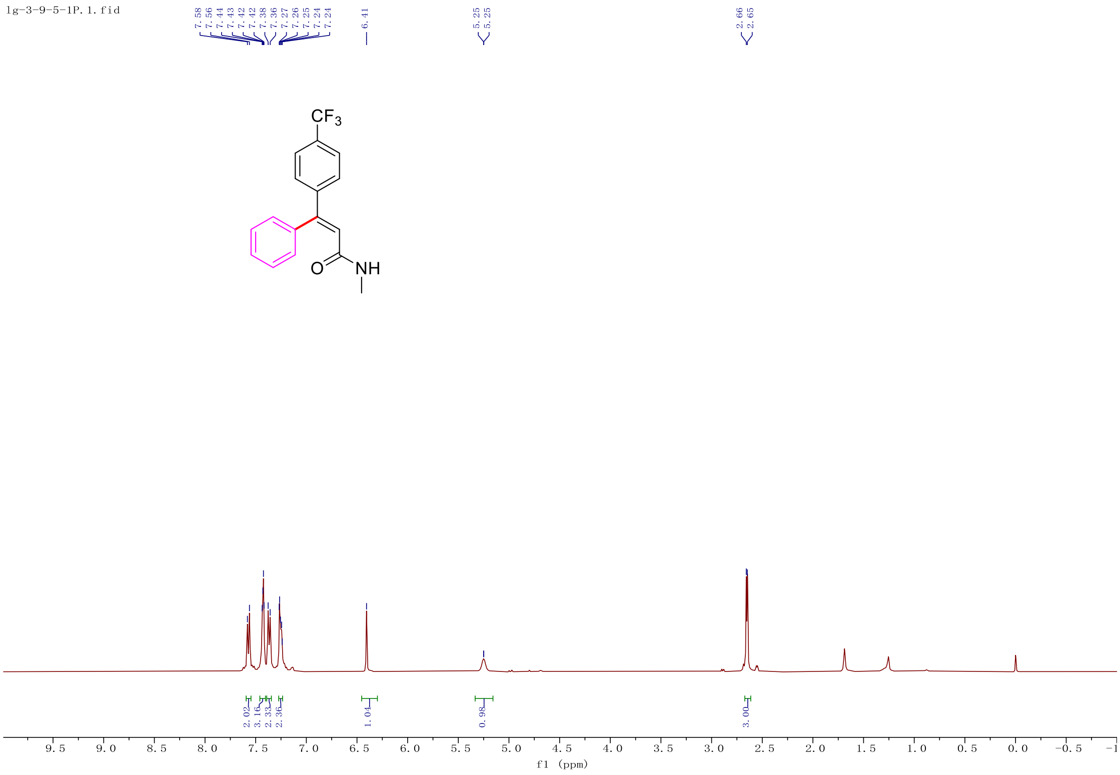

^{13}C {^{1}H} NMR Spectrum of (*E*)-*N*-methyl-3-phenyl-3-(4-(trifluoromethyl)phenyl)acrylamide (*E*-70, $CDCl_3$ as solvent, 101 MHz)

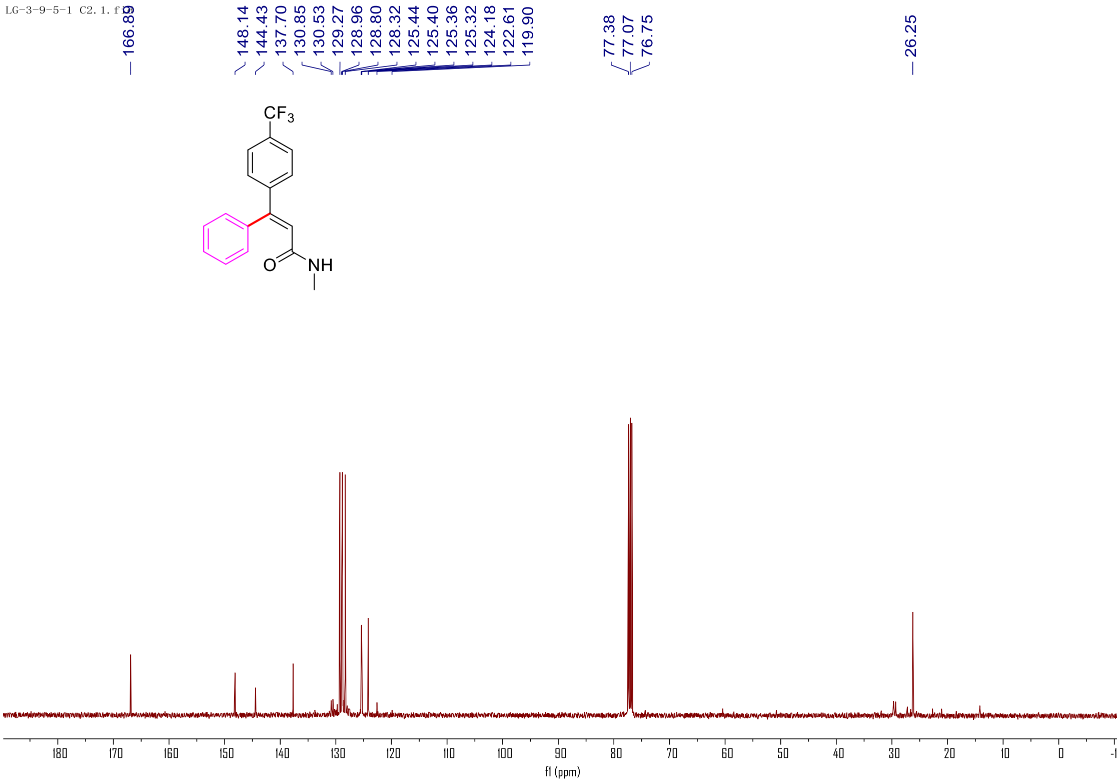

^{19}F NMR Spectrum of (*E*)-*N*-methyl-3-phenyl-3-(4-(trifluoromethyl)phenyl)acrylamide (*E*-70, $CDCl_3$ as solvent, 376 MHz)

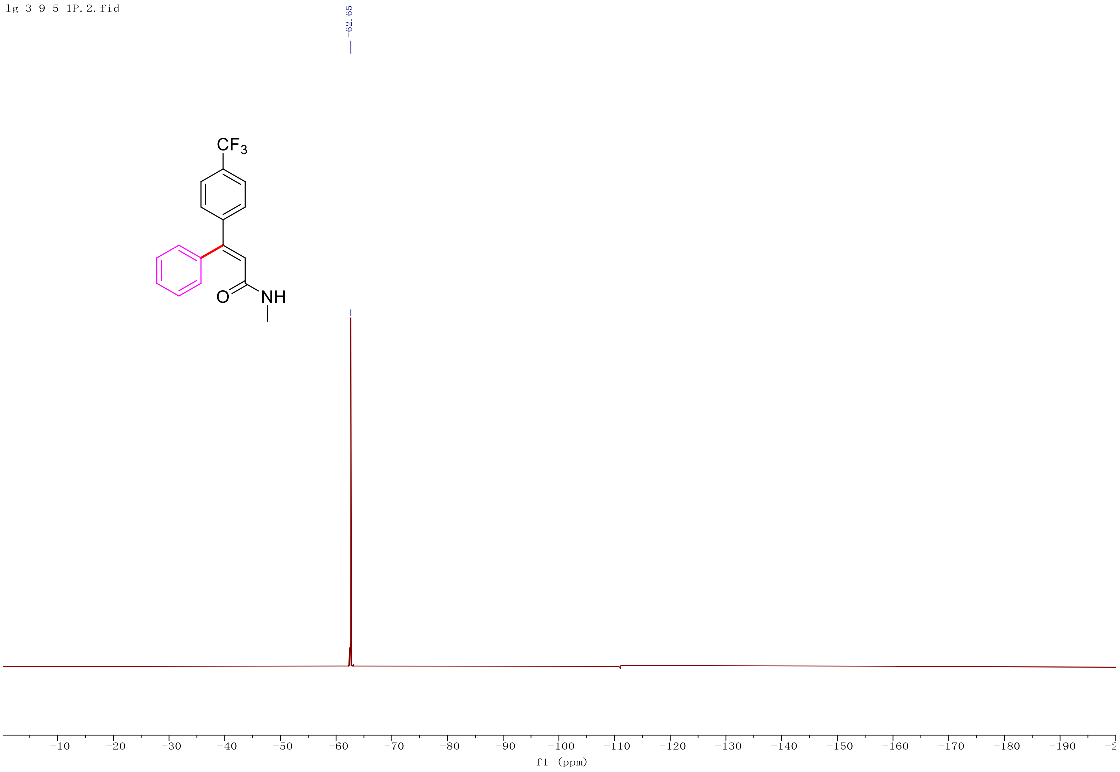

^{1}H NMR Spectrum of *N*-methyl-3-phenyl-3-(m-tolyl)acrylamide (71, $CDCl_3$ as solvent, 400 MHz)

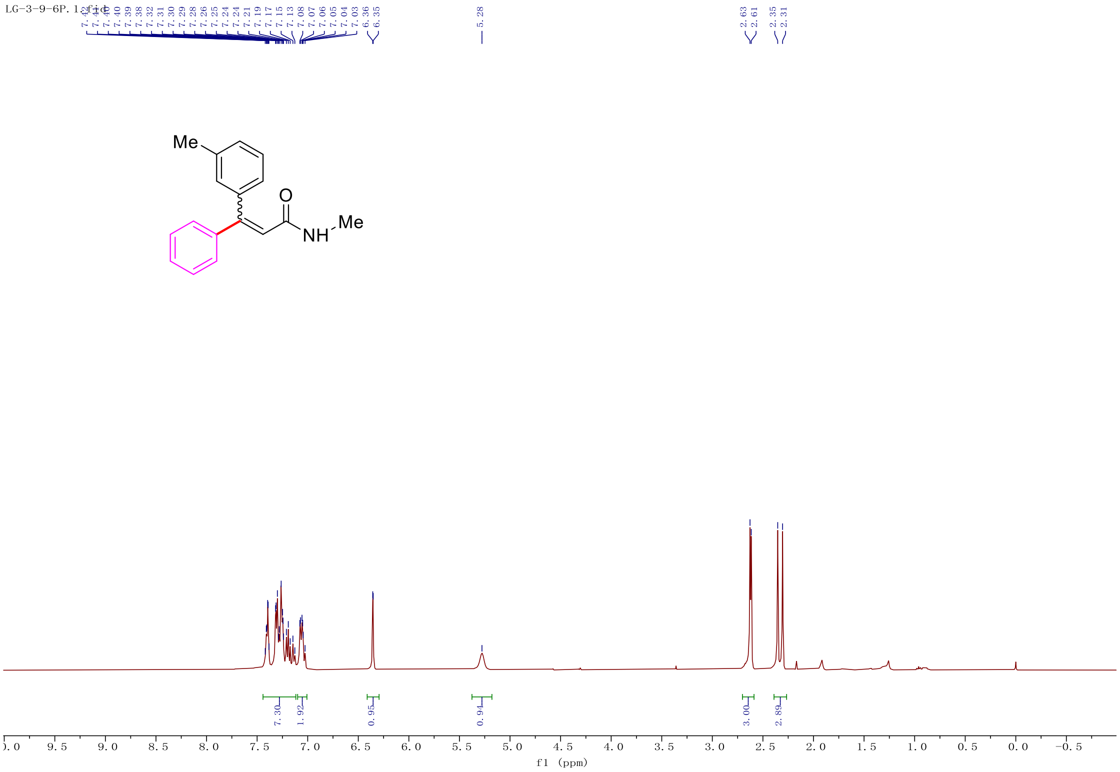

^{13}C {^{1}H} NMR Spectrum of *N*-methyl-3-phenyl-3-(m-tolyl)acrylamide (71, $CDCl_3$ as solvent, 101 MHz)

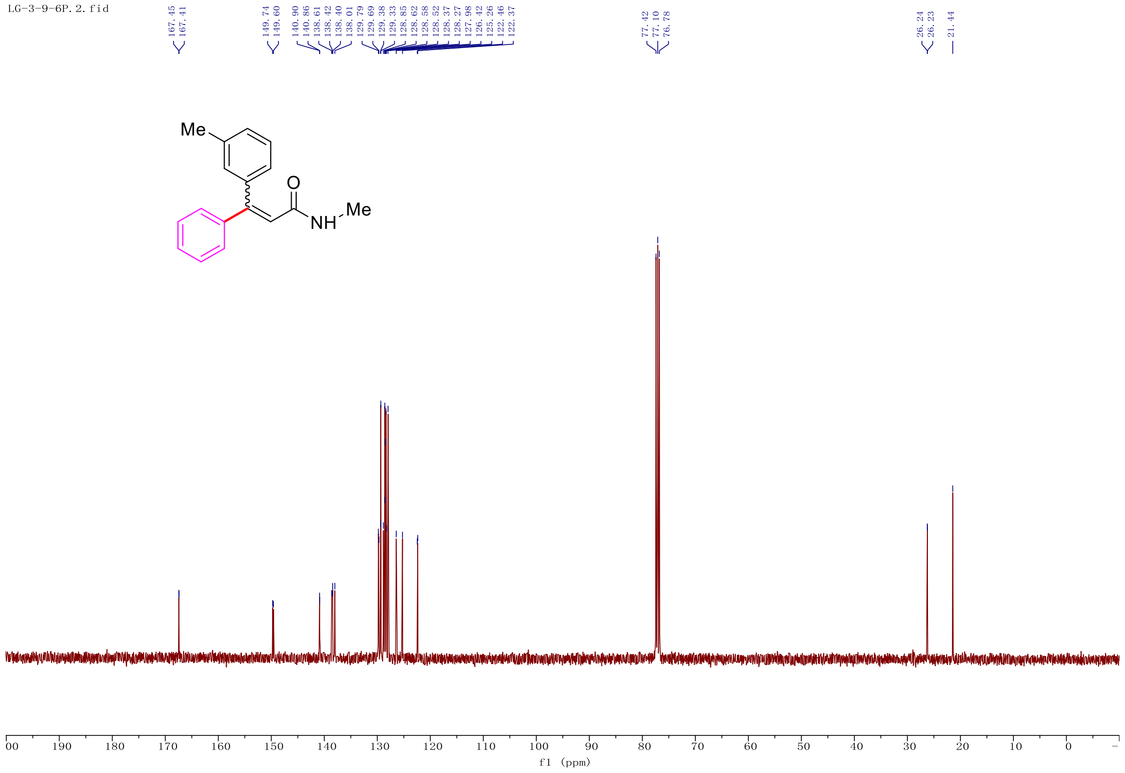

^{1}H NMR Spectrum of *N*-methyl-3-(naphthalen-1-yl)-3-phenylacrylamide (72, $CDCl_3$ as solvent, 400 MHz)

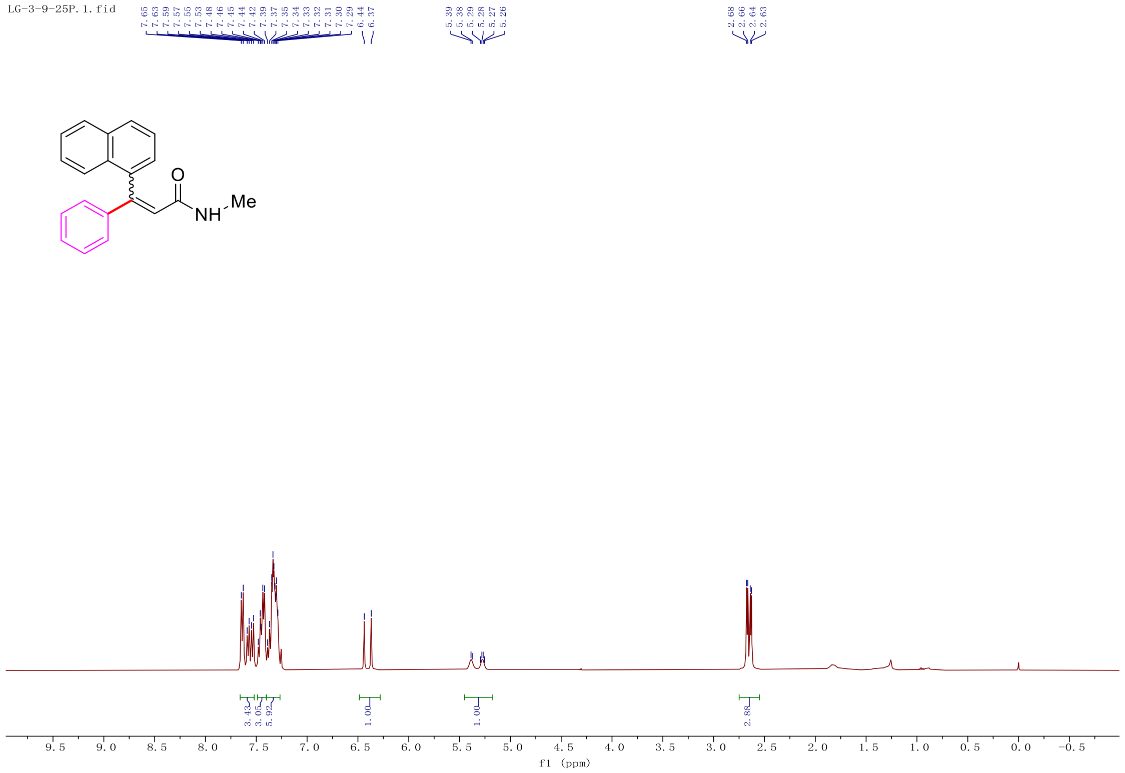

^{1}H NMR Spectrum for *Z/E* isomerization of product 72 ($CDCl_3$ as solvent, 400 MHz)

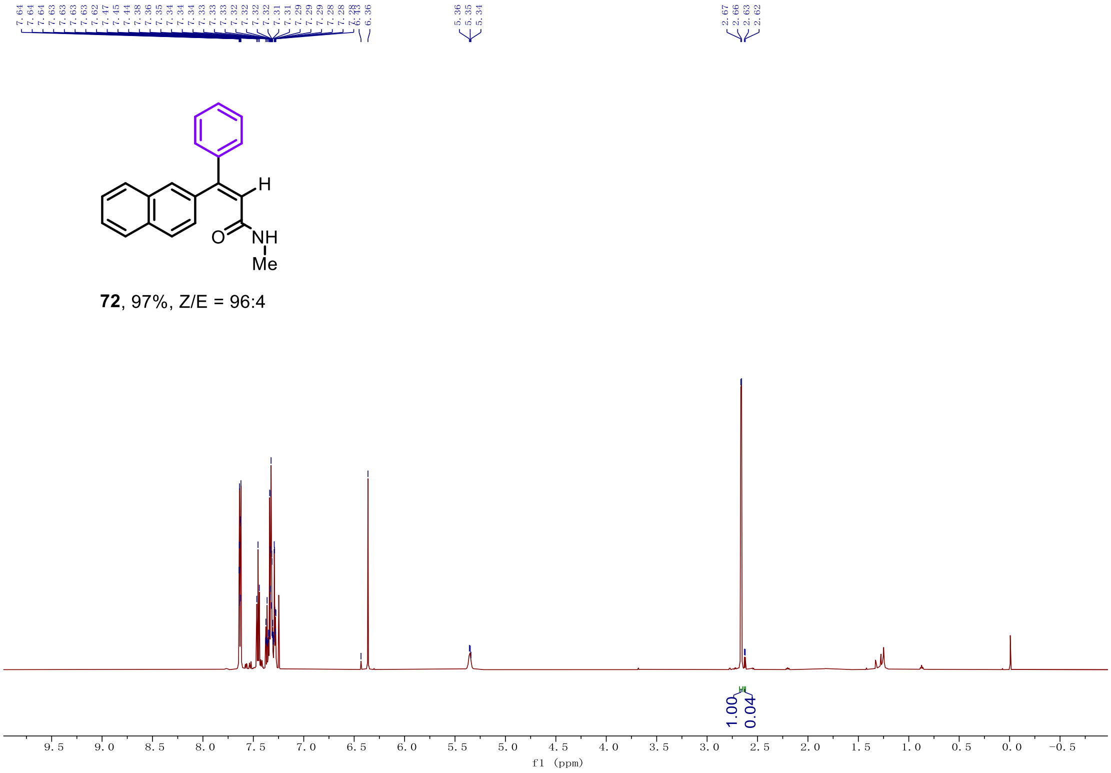

^{13}C {^{1}H} NMR Spectrum of *N*-methyl-3-(naphthalen-1-yl)-3-phenylacrylamide (72, $CDCl_3$ as solvent, 101 MHz)

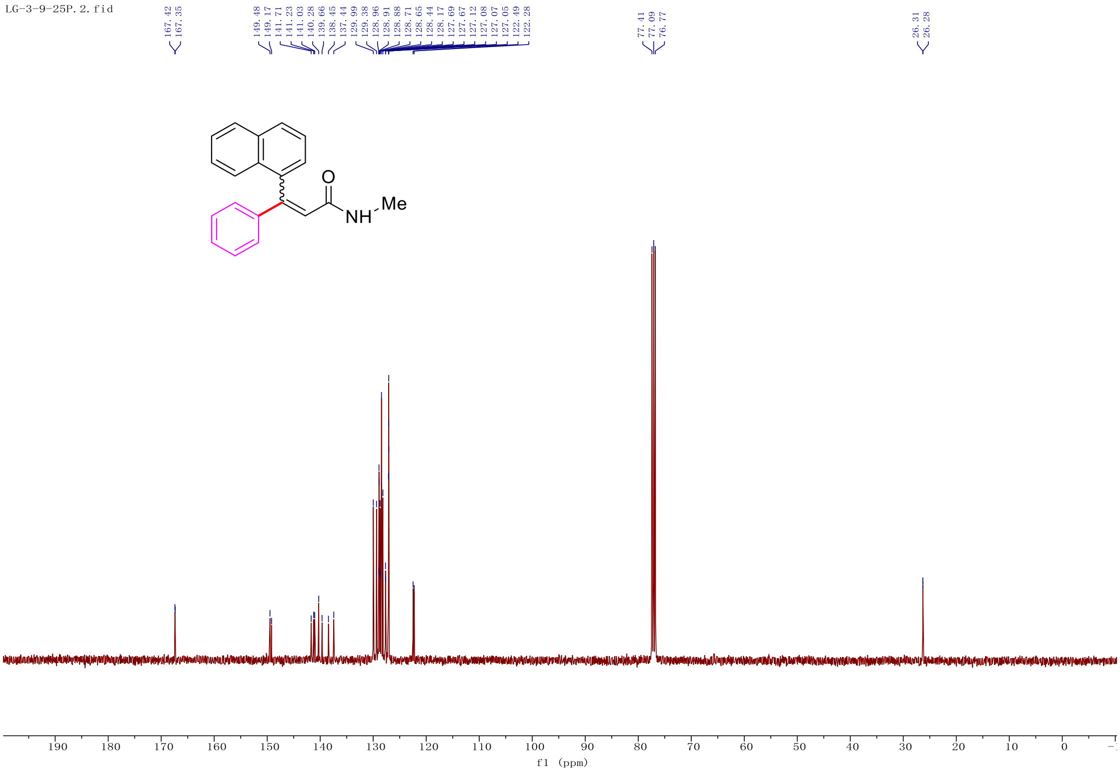

^{1}H NMR Spectrum of *N*-methyl-3-(1-methyl-1*H*-indol-5-yl)-3-phenylacrylamide (73, $CDCl_3$ as solvent, 400 MHz)

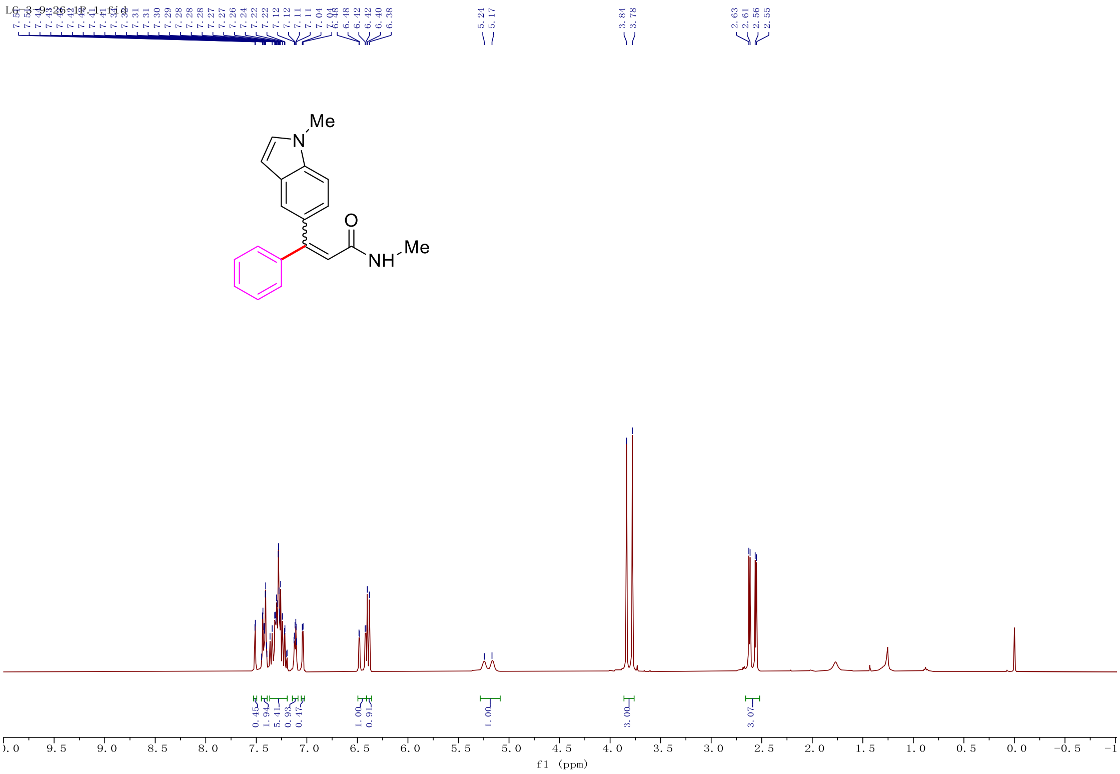

^{13}C {^{1}H} NMR Spectrum of *N*-methyl-3-(1-methyl-1*H*-indol-5-yl)-3-phenylacrylamide (73, $CDCl_3$ as solvent, 101 MHz)

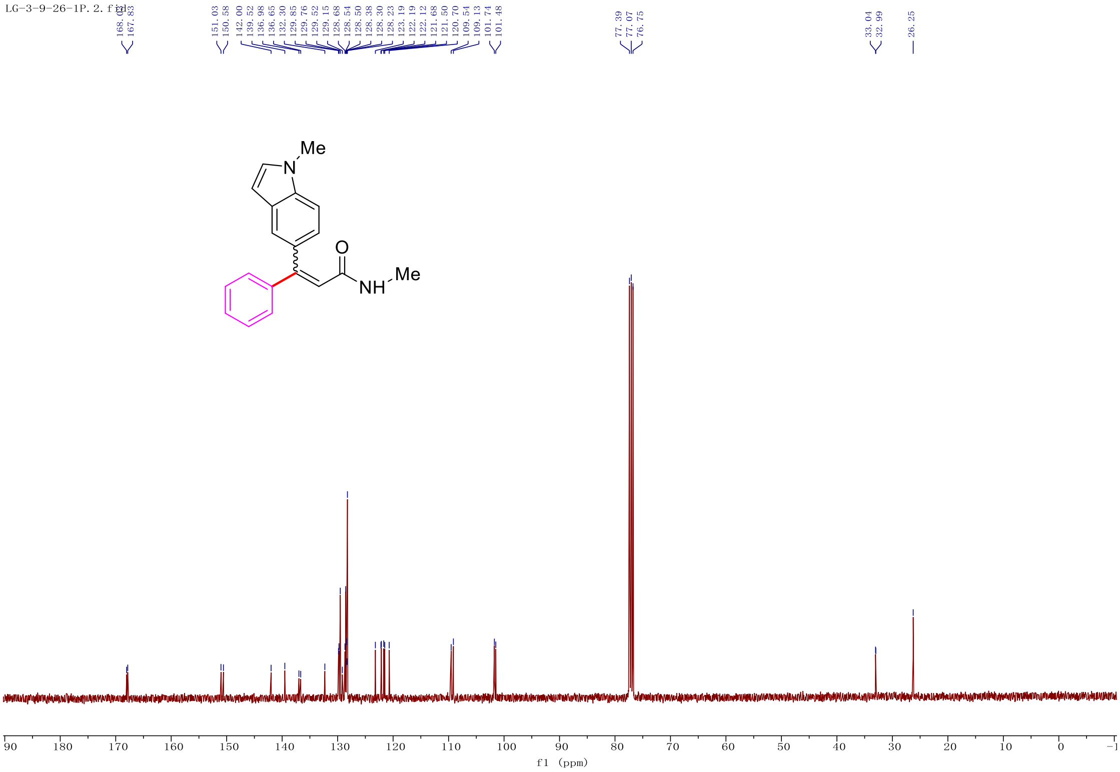

^{1}H NMR Spectrum of 3-(dibenzo[b,d]thiophen-4-yl)-*N*-methyl-3-phenylacrylamide (74, $CDCl_3$ as solvent, 400 MHz)

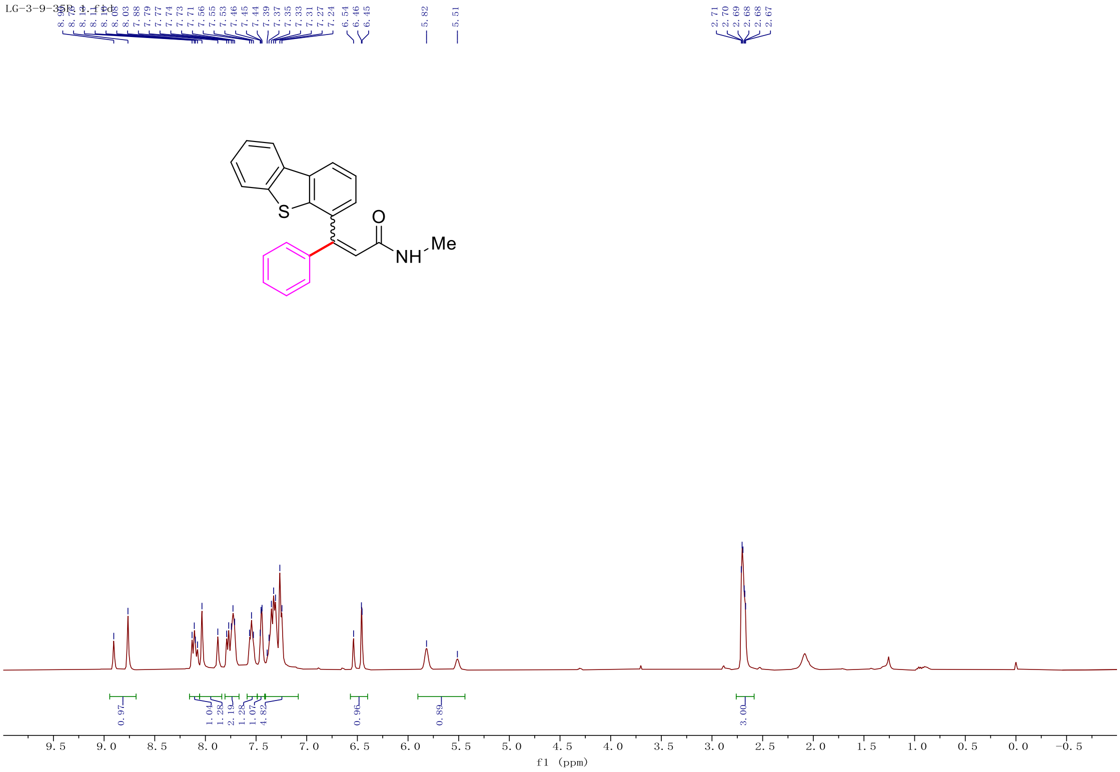

^{1}H NMR Spectrum for *Z/E* isomerization of product 74 ($CDCl_3$ as solvent, 400 MHz)

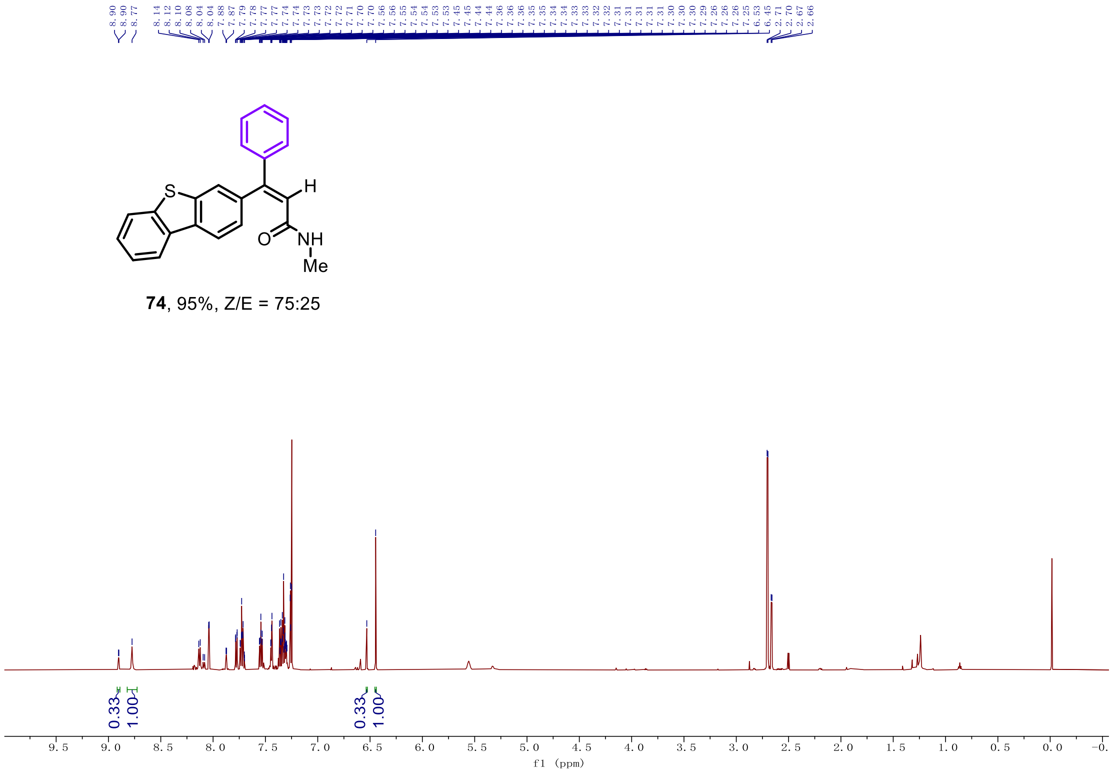

74, 95%, Z/E = 75:25

^{13}C {^{1}H} NMR Spectrum of 3-(dibenzo[b,d]thiophen-4-yl)-*N*-methyl-3-phenylacrylamide (74, $CDCl_3$ as solvent, 101 MHz)

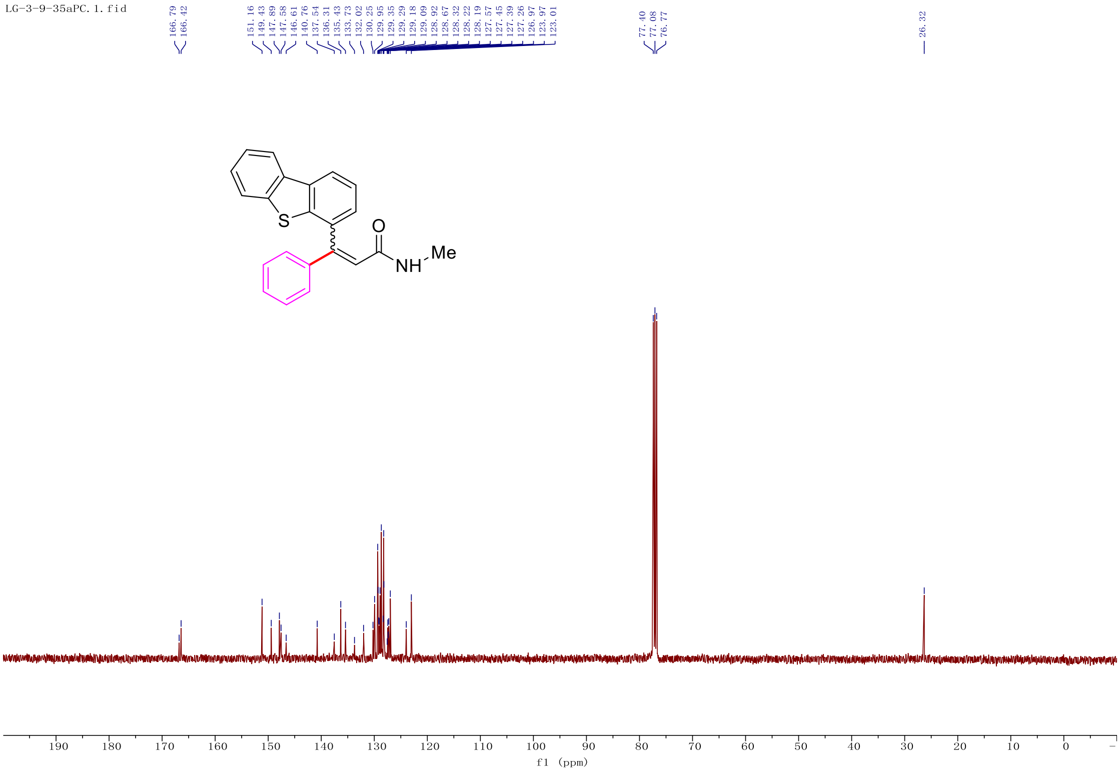

^{1}H NMR Spectrum of 4-(3-(methylamino)-3-oxo-1-phenylprop-1-en-1-yl)benzyl 2-(3-benzoylphenyl)propanoate (75, $CDCl_3$ as solvent, 400 MHz)

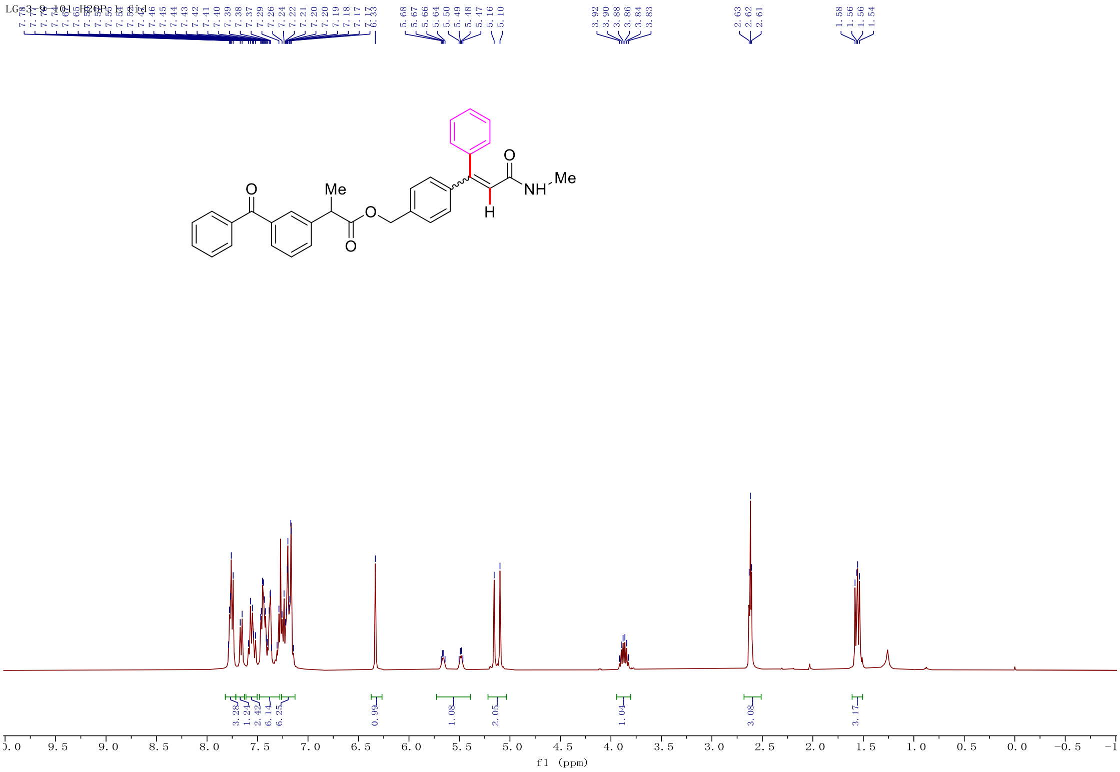

^{13}C {^{1}H} NMR Spectrum of 4-(3-(methylamino)-3-oxo-1-phenylprop-1-en-1-yl)benzyl 2-(3-benzoylphenyl)propanoate (75, $CDCl_3$ as solvent, 101 MHz)

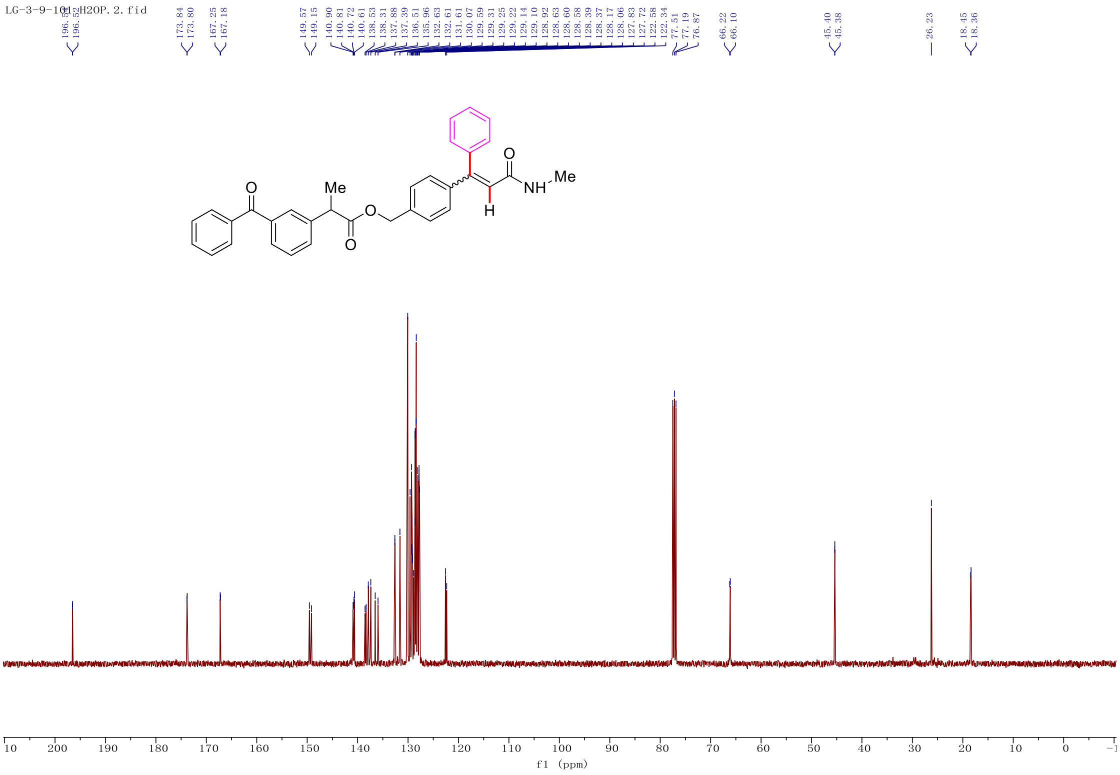

^{1}H NMR Spectrum of 4-(3-(methylamino)-3-oxo-1-phenylprop-1-en-1-yl)phenyl 4-(*N*,*N*-dipropylsulfamoyl)benzoate (76, $CDCl_3$ as solvent, 400 MHz)

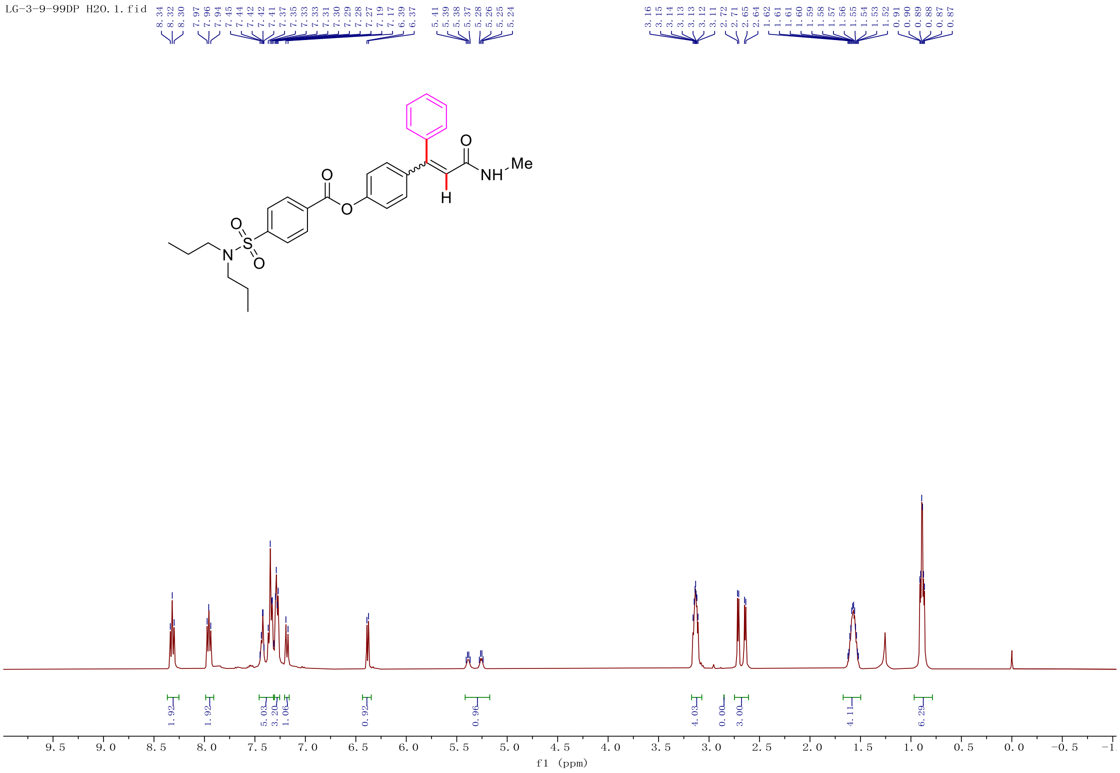

^{13}C {^{1}H} NMR Spectrum of 4-(3-(methylamino)-3-oxo-1-phenylprop-1-en-1-yl)phenyl 4-(*N*,*N*-dipropylsulfamoyl)benzoate (76, $CDCl_3$ as solvent, 101 MHz)

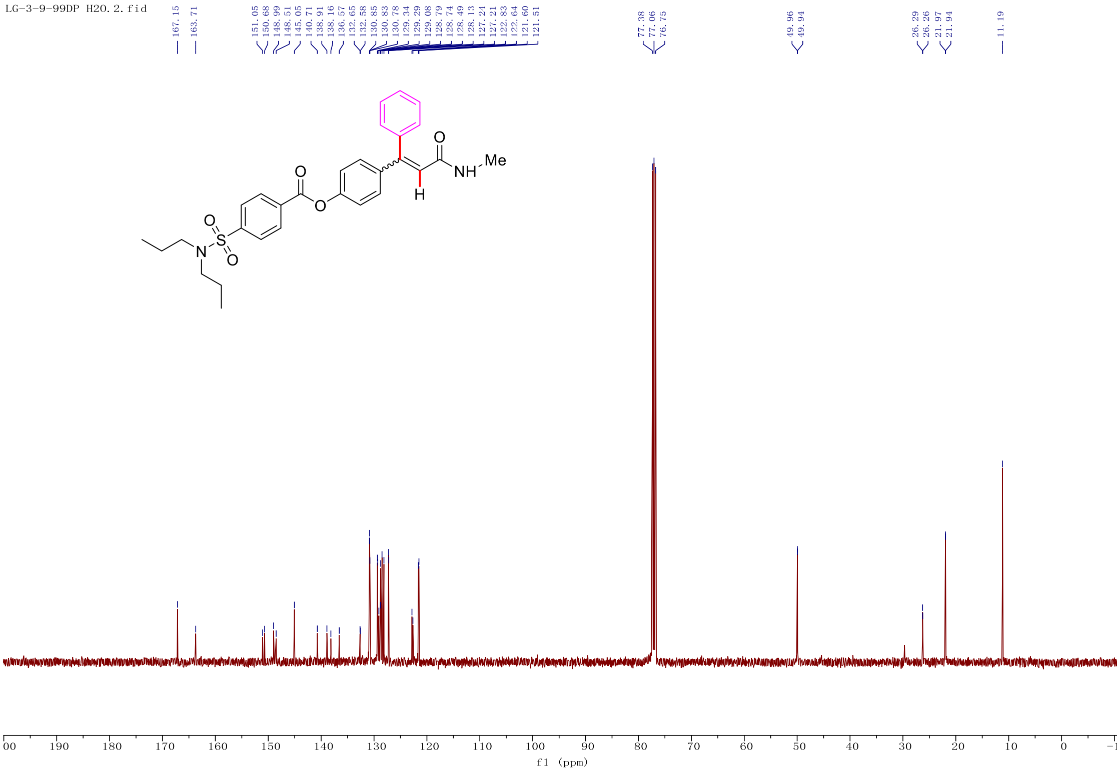

^{1}H NMR Spectrum of (8S,9R,13R,14R)-13-methyl-17-oxo-7,8,9,11,12,13,14,15,16,17-decahydro-6H-cyclopenta[a]phenanthren-3-yl 4-(3-(methylamino)-3-oxo-1-phenylprop-1-en-1-yl)benzoate (77, $CDCl_3$ as solvent, 400 MHz)

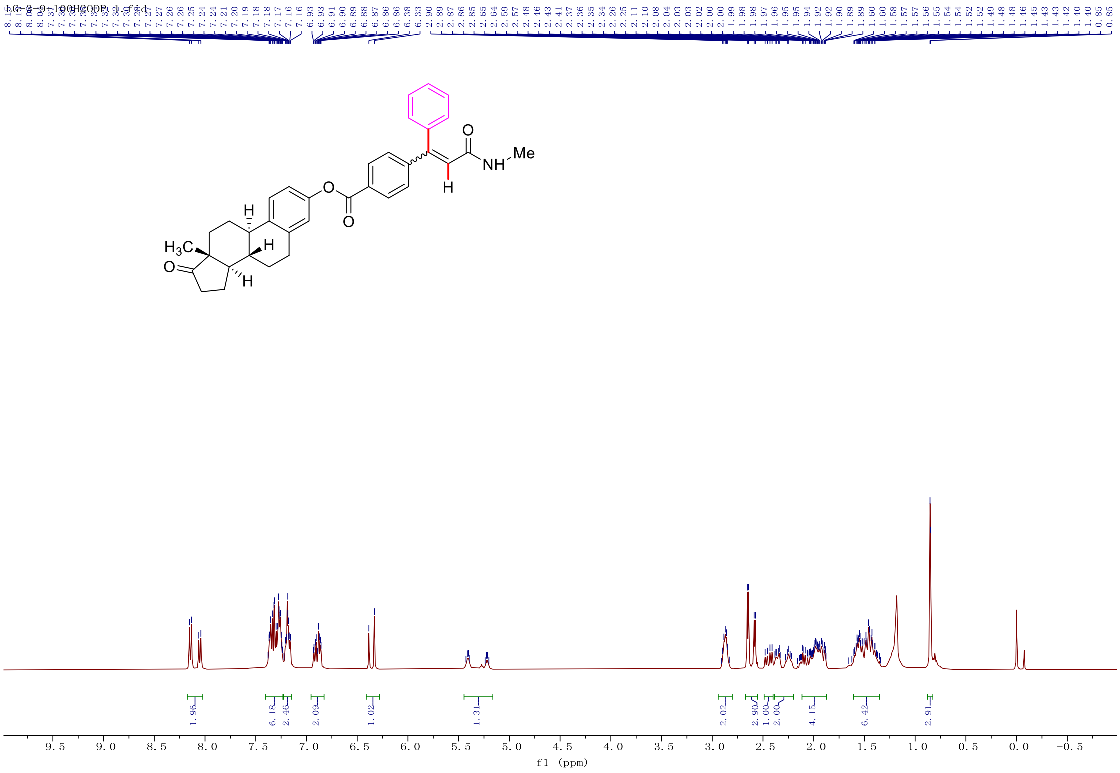

^{13}C {^{1}H} NMR Spectrum of (8S,9R,13R,14R)-13-methyl-17-oxo-7,8,9,11,12,13,14,15,16,17-decahydro-6H-cyclopenta[a]phenanthren-3-yl 4-(3-(methylamino)-3-oxo-1-phenylprop-1-en-1-yl)benzoate (77, $CDCl_3$ as solvent, 101 MHz)

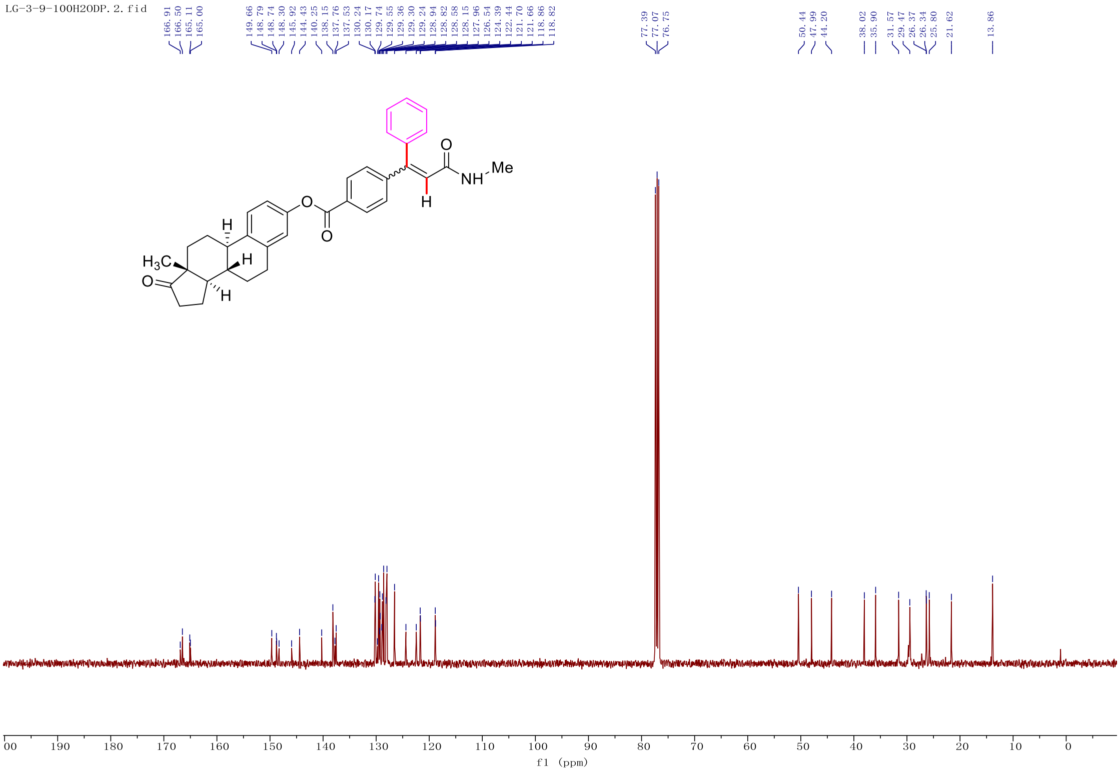

^{1}H NMR Spectrum of *N*-methyl-3-phenyl-3-(*p*-tolyl)acrylamide (78, $CDCl_3$ as solvent, 400 MHz)

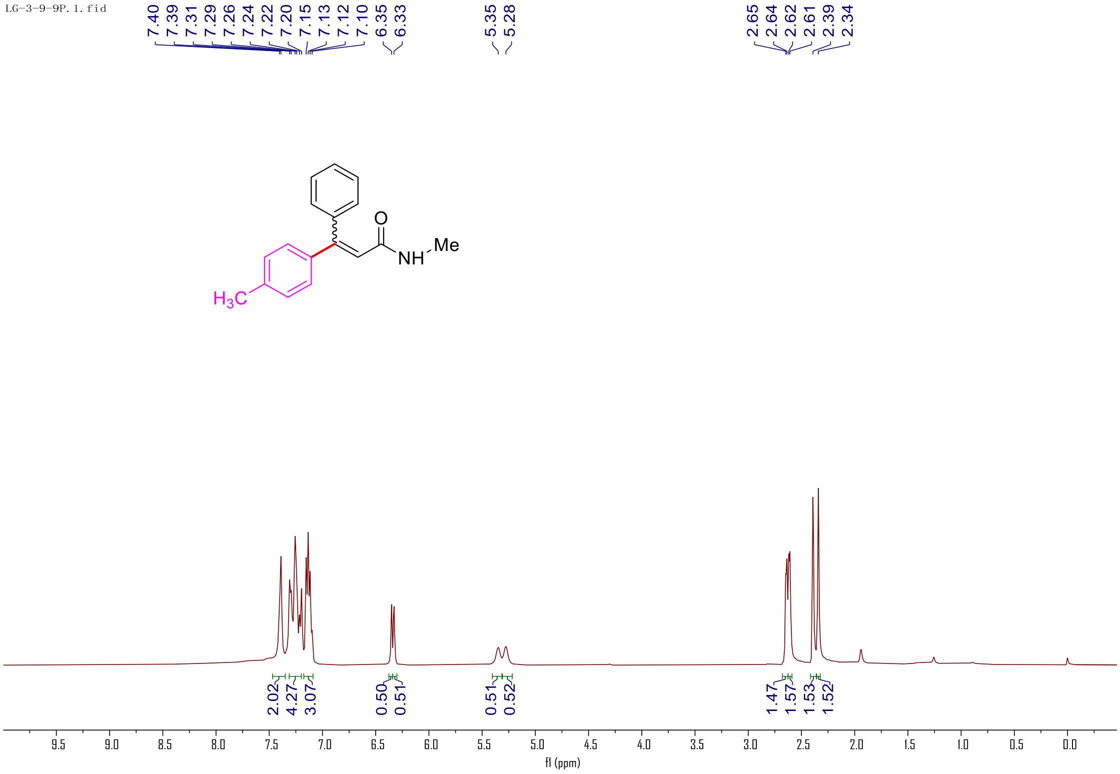

^{13}C {^{1}H} NMR Spectrum of *N*-methyl-3-phenyl-3-(*p*-tolyl)acrylamide (78, $CDCl_3$ as solvent, 101 MHz)

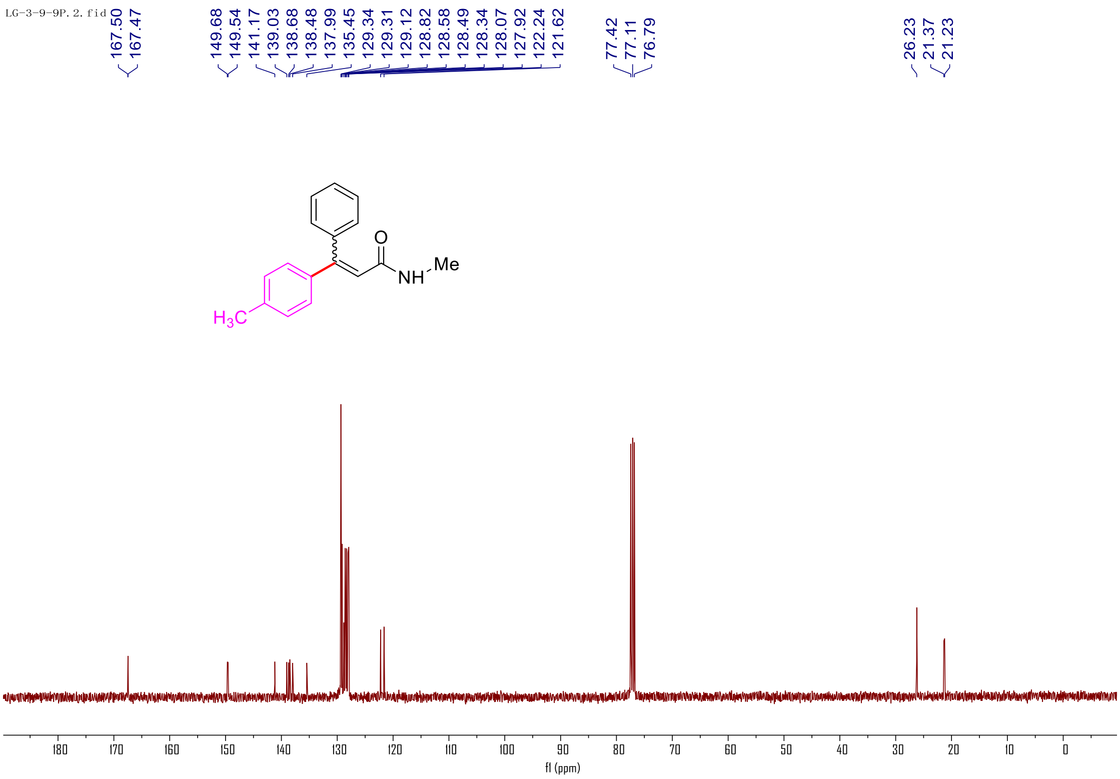

^{1}H NMR Spectrum of 3-([1,1'-biphenyl]-4-yl)-*N*-methyl-3-phenylacrylamide (79, $CDCl_3$ as solvent, 400 MHz)

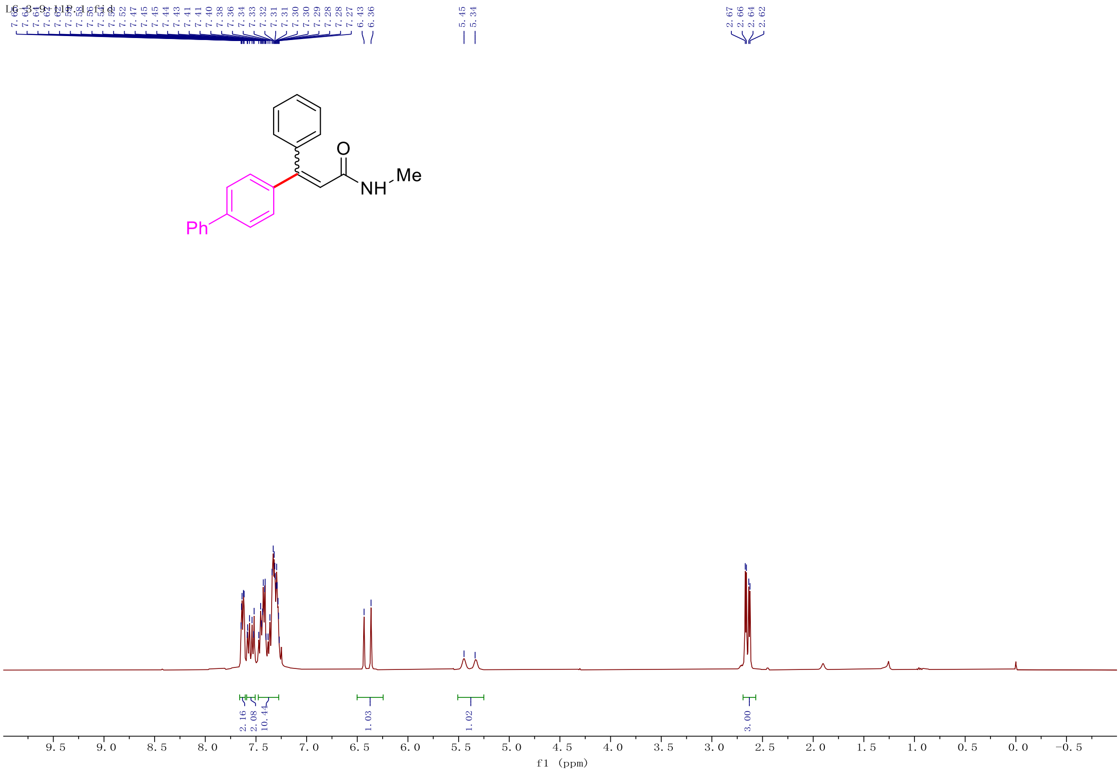

^{1}H NMR Spectrum for *Z/E* isomerization of product 79 ($CDCl_3$ as solvent, 600 MHz)

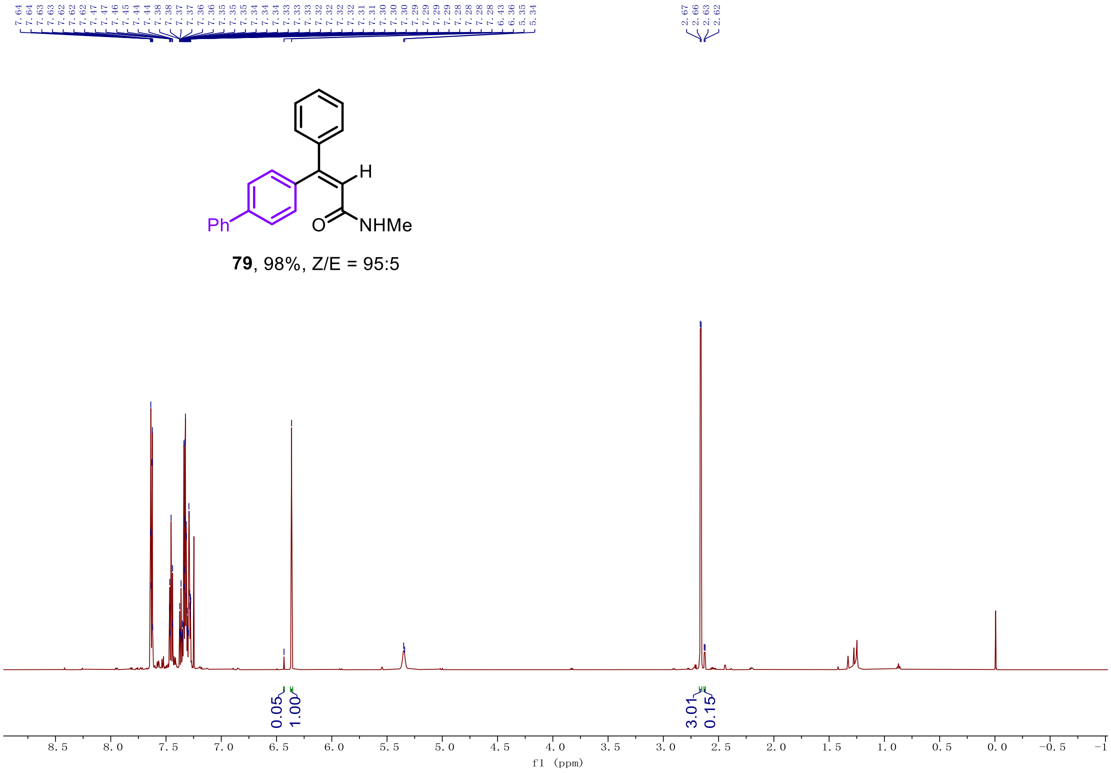

^{13}C {^{1}H} NMR Spectrum of 3-([1,1'-biphenyl]-4-yl)-*N*-methyl-3-phenylacrylamide (79, $CDCl_3$ as solvent, 101 MHz)

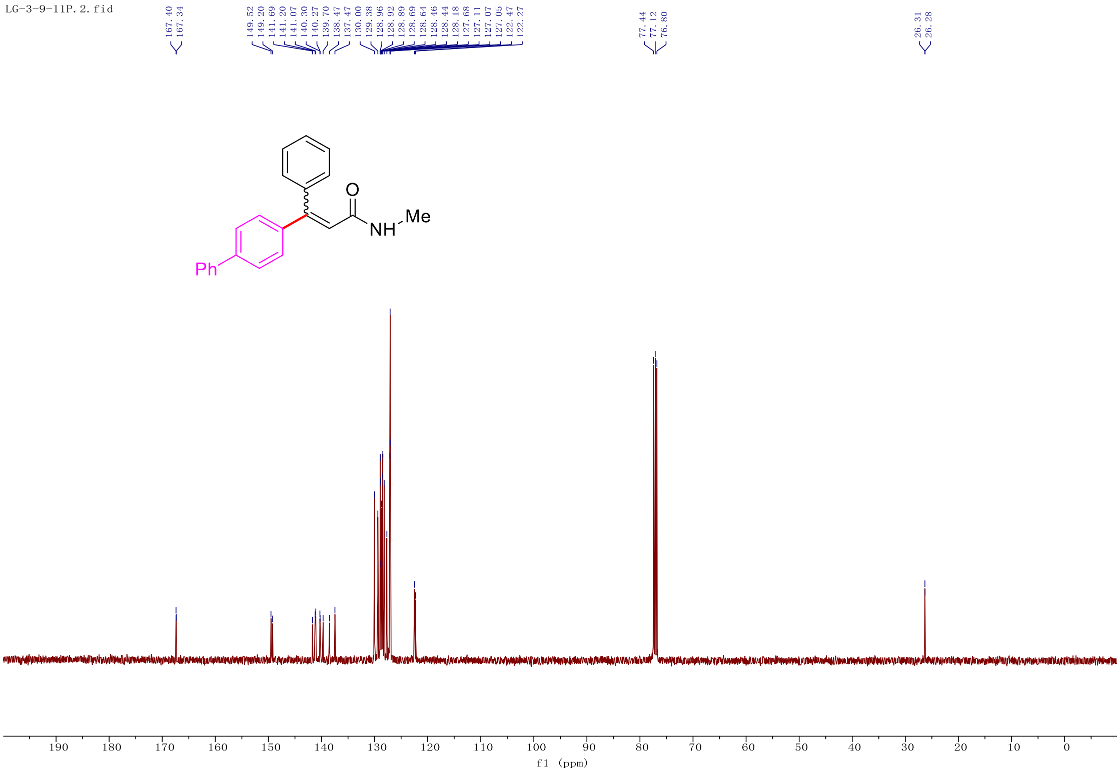

^{1}H NMR Spectrum of 3-(4-methoxyphenyl)-*N*-methyl-3-phenylacrylamide (80, $CDCl_3$ as solvent, 400 MHz)

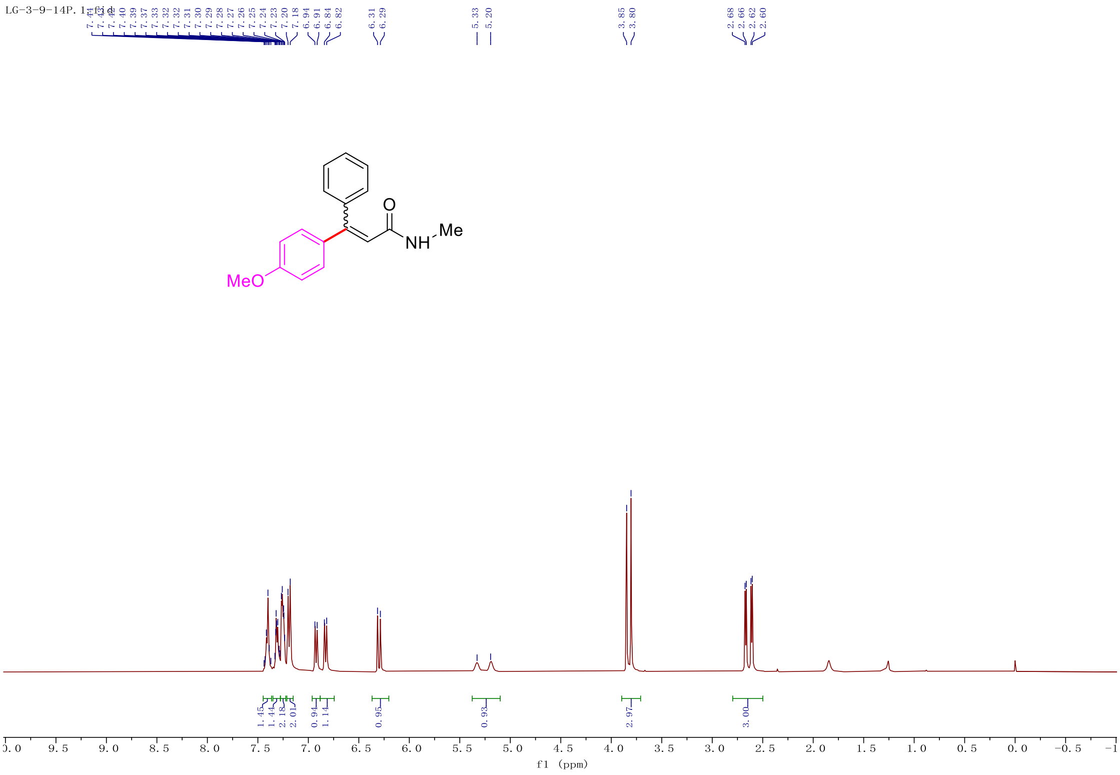

^{13}C {^{1}H} NMR Spectrum of 3-(4-methoxyphenyl)-*N*-methyl-3-phenylacrylamide (80, $CDCl_3$ as solvent, 101 MHz)

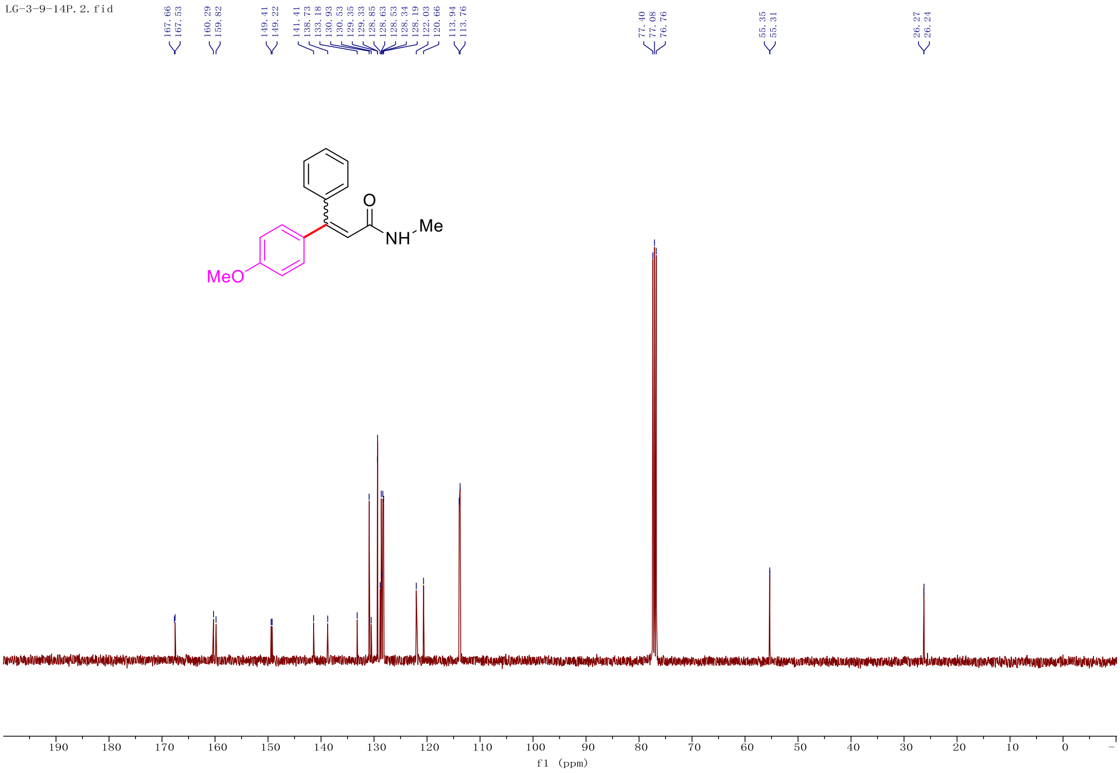

^{1}H NMR Spectrum of 3-(4-fluorophenyl)-*N*-methyl-3-phenylacrylamide (81, $CDCl_3$ as solvent, 400 MHz)

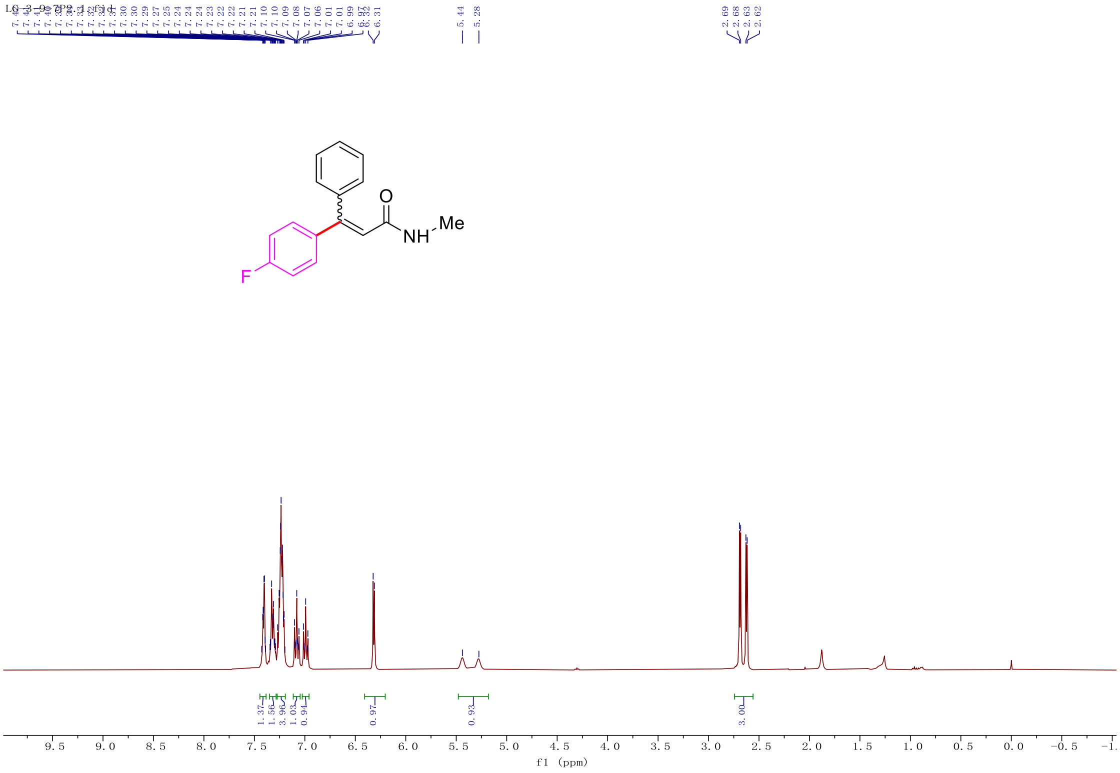

^{13}C {^{1}H} NMR Spectrum of 3-(4-fluorophenyl)-*N*-methyl-3-phenylacrylamide (81, $CDCl_3$ as solvent, 101 MHz)

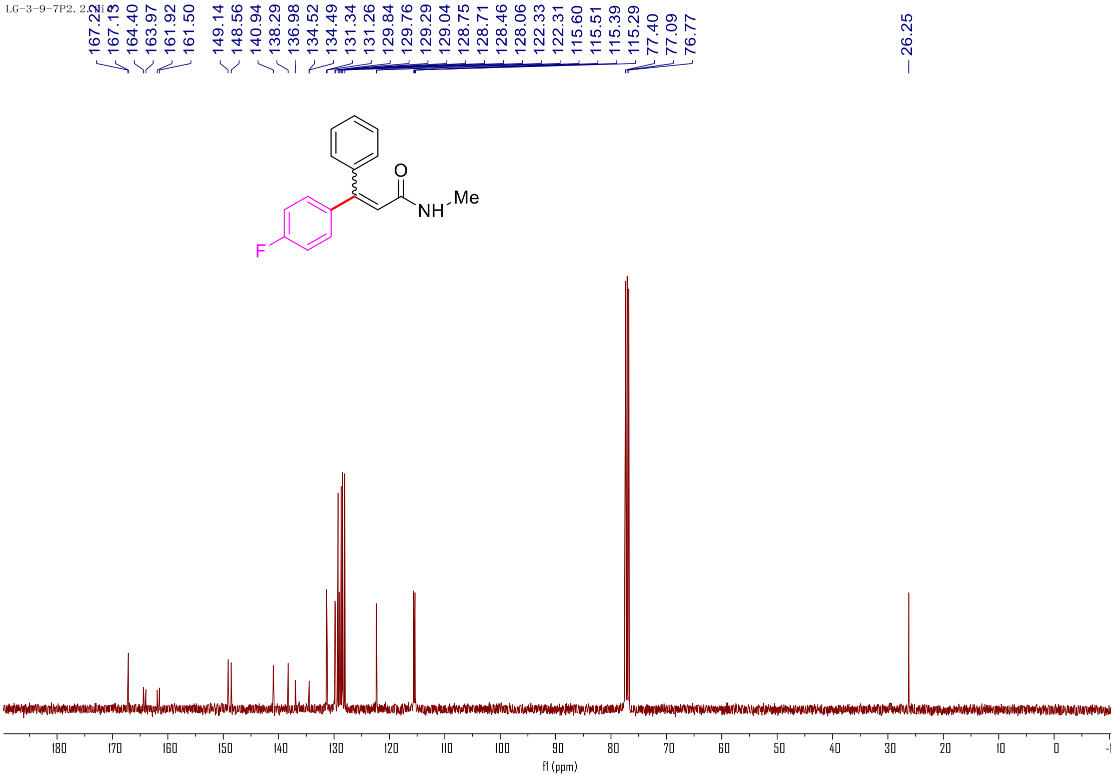

^{19}F NMR Spectrum of 3-(4-fluorophenyl)-*N*-methyl-3-phenylacrylamide (81, $CDCl_3$ as solvent, 376 MHz)

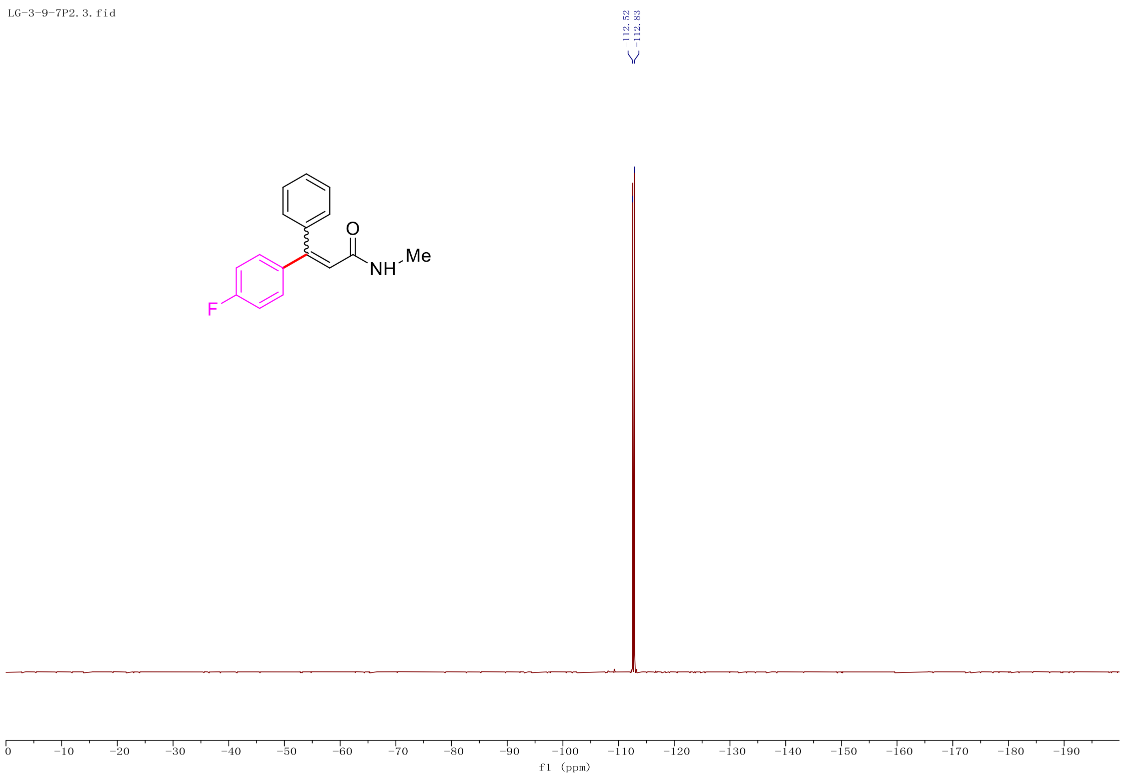

^{1}H NMR Spectrum of 3-(4-chlorophenyl)-*N*-methyl-3-phenylacrylamide (82, $CDCl_3$ as solvent, 400 MHz)

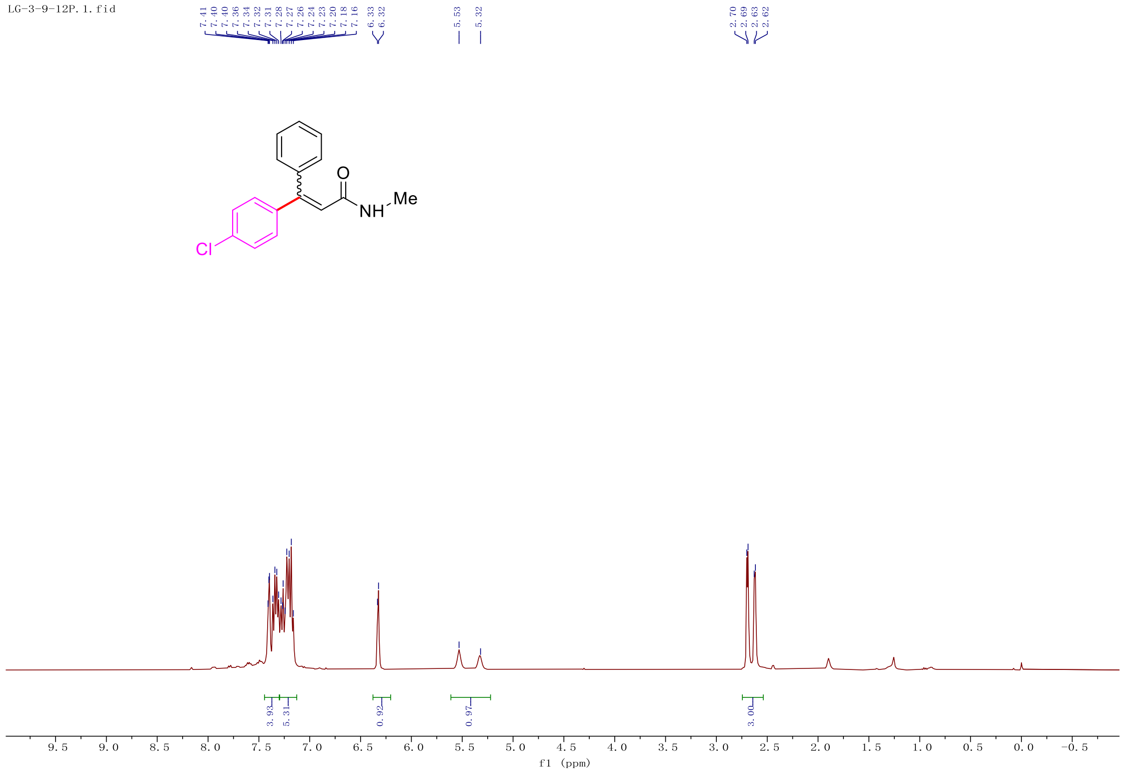

^{1}H NMR Spectrum for *Z/E* isomerization of product 82 ($CDCl_3$ as solvent, 600 MHz)

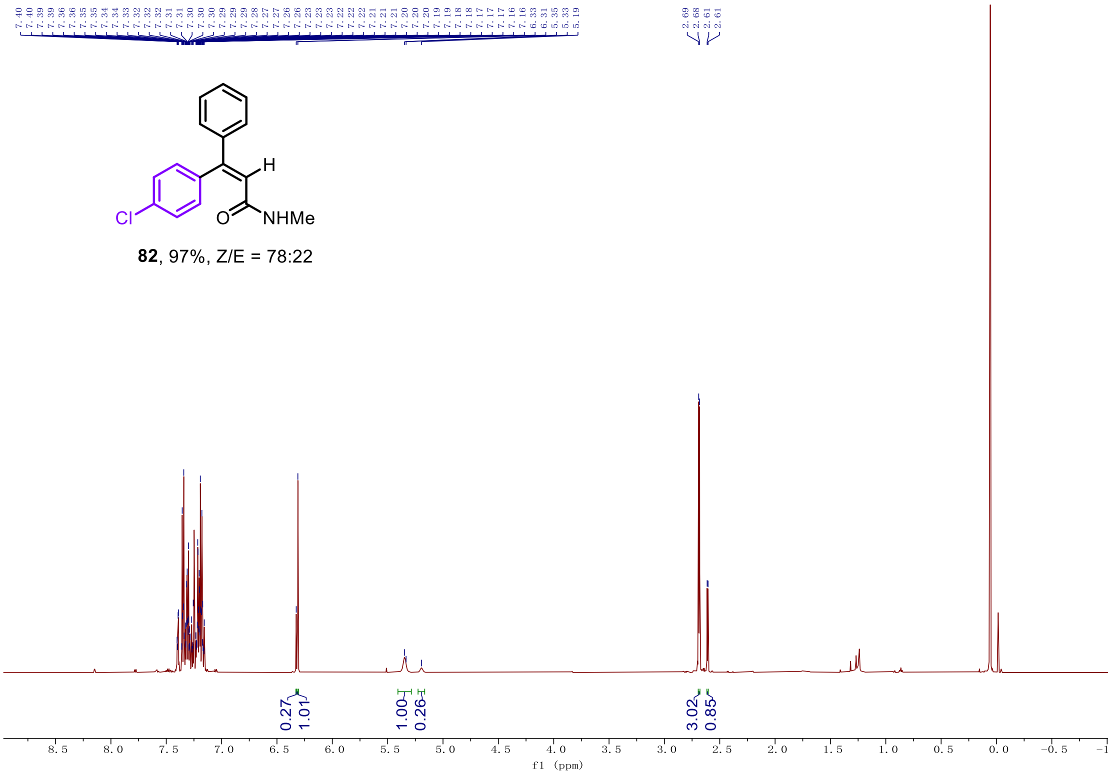

^{13}C {^{1}H} NMR Spectrum of 3-(4-chlorophenyl)-*N*-methyl-3-phenylacrylamide (82, $CDCl_3$ as solvent, 101 MHz)

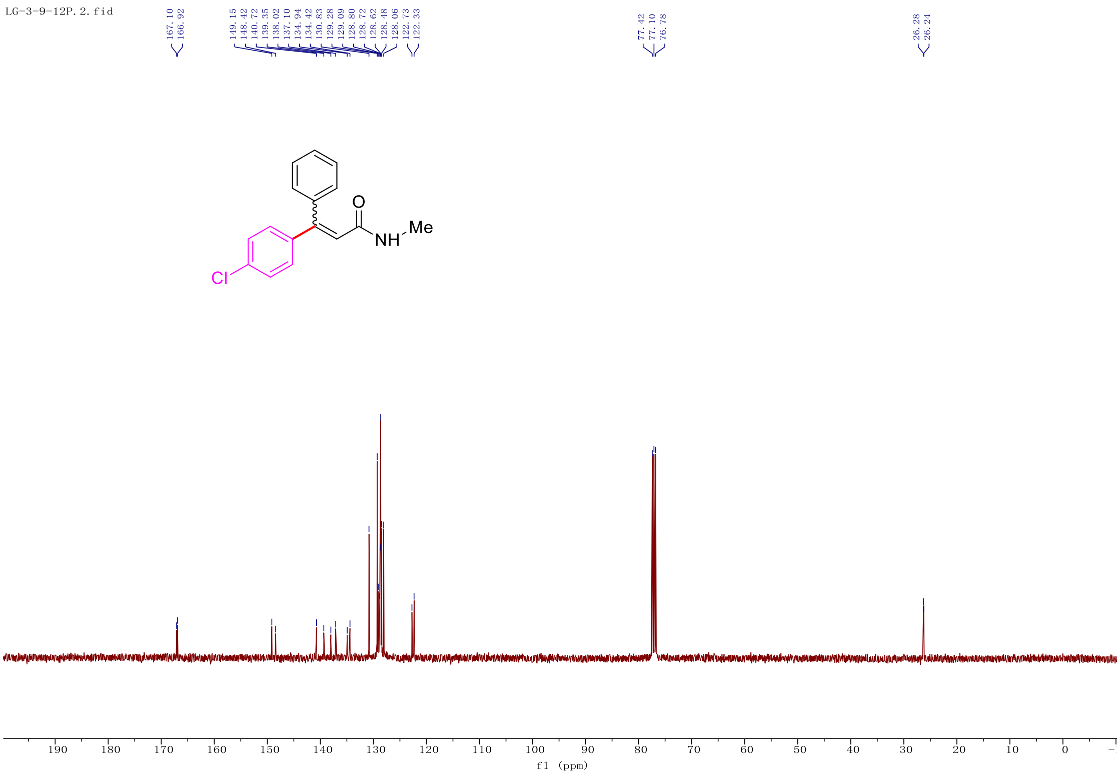

^{1}**H NMR Spectrum of 3-(4-bromophenyl)-*N*-methyl-3-phenylacrylamide (83, CDCl$_3$ as solvent, 400 MHz)**

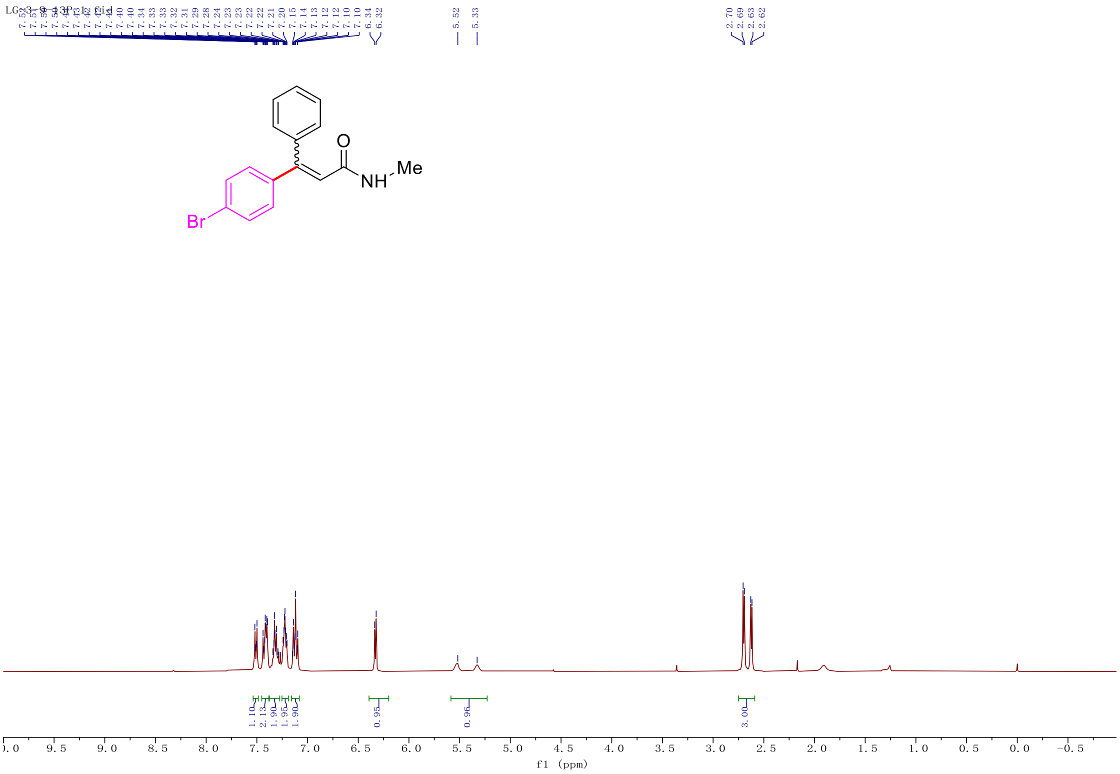

^{1}**H NMR Spectrum for *Z/E* isomerization of product 83 (CDCl$_3$ as solvent, 600 MHz)**

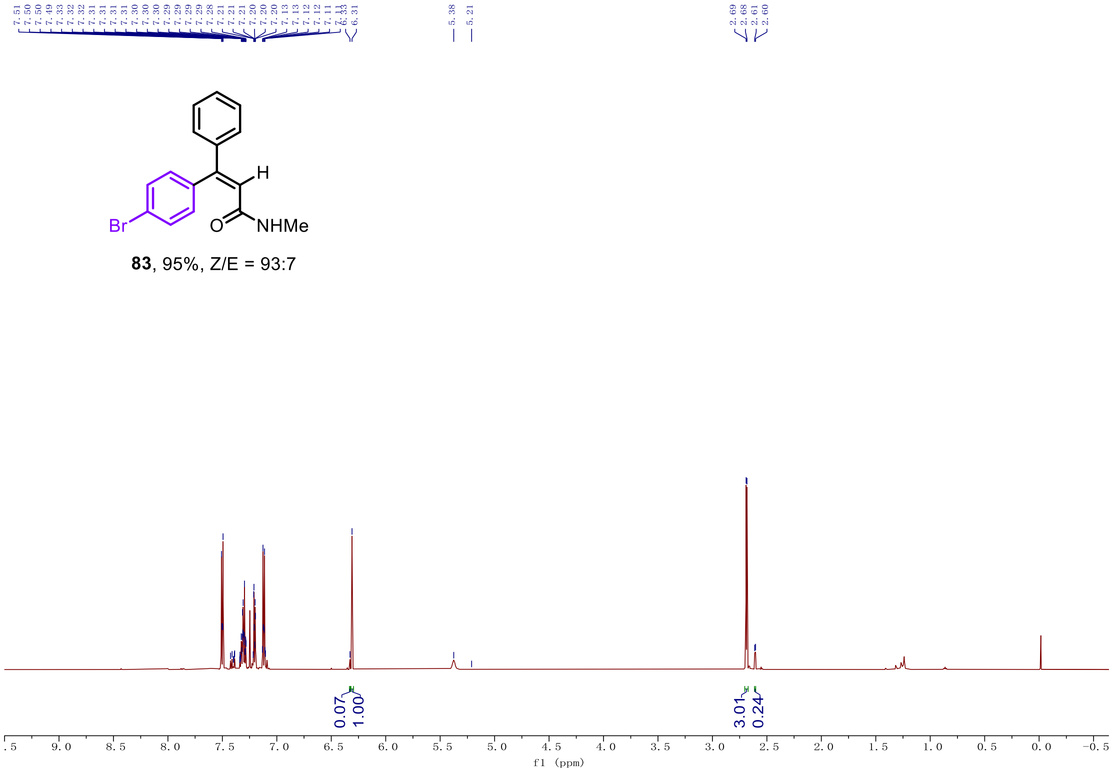

83, 95%, Z/E = 93:7

$^{13}C\ \{^{1}H\}$ NMR Spectrum of 3-(4-bromophenyl)-*N*-methyl-3-phenylacrylamide (83, $CDCl_3$ as solvent, 101 MHz)

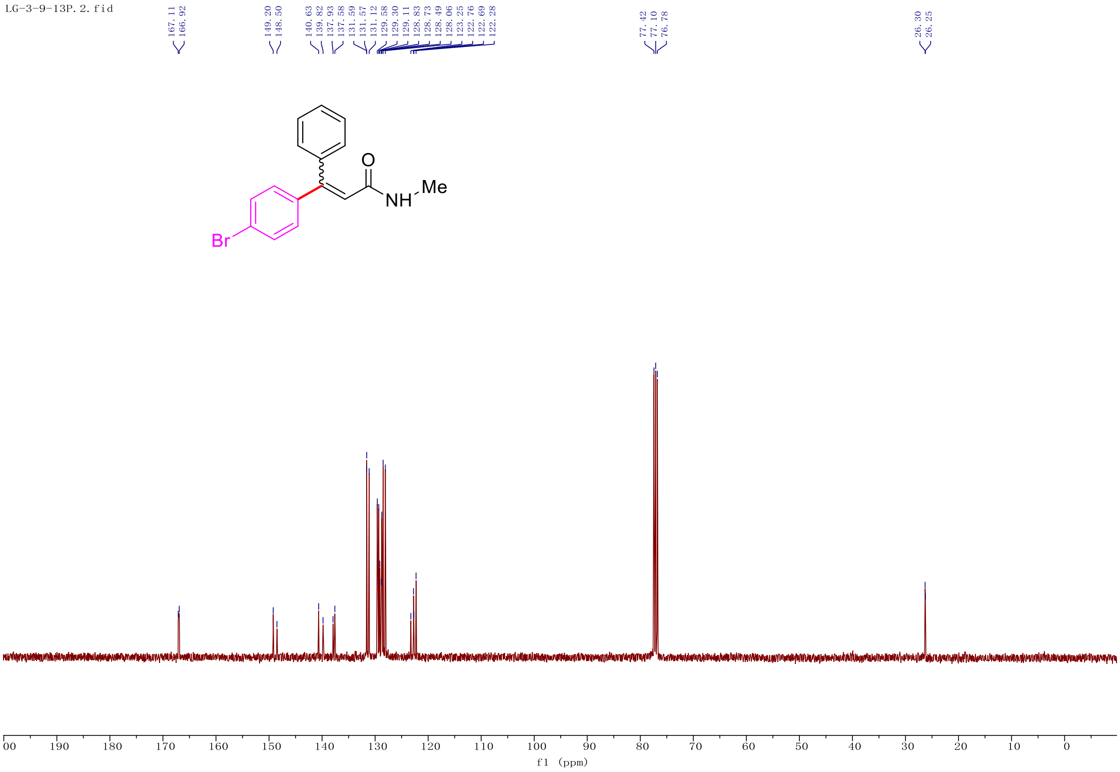

^{1}H NMR Spectrum of *N*-methyl-3-phenyl-3-(4-(trifluoromethyl)phenyl)acrylamide (84, $CDCl_3$ as solvent, 400 MHz)

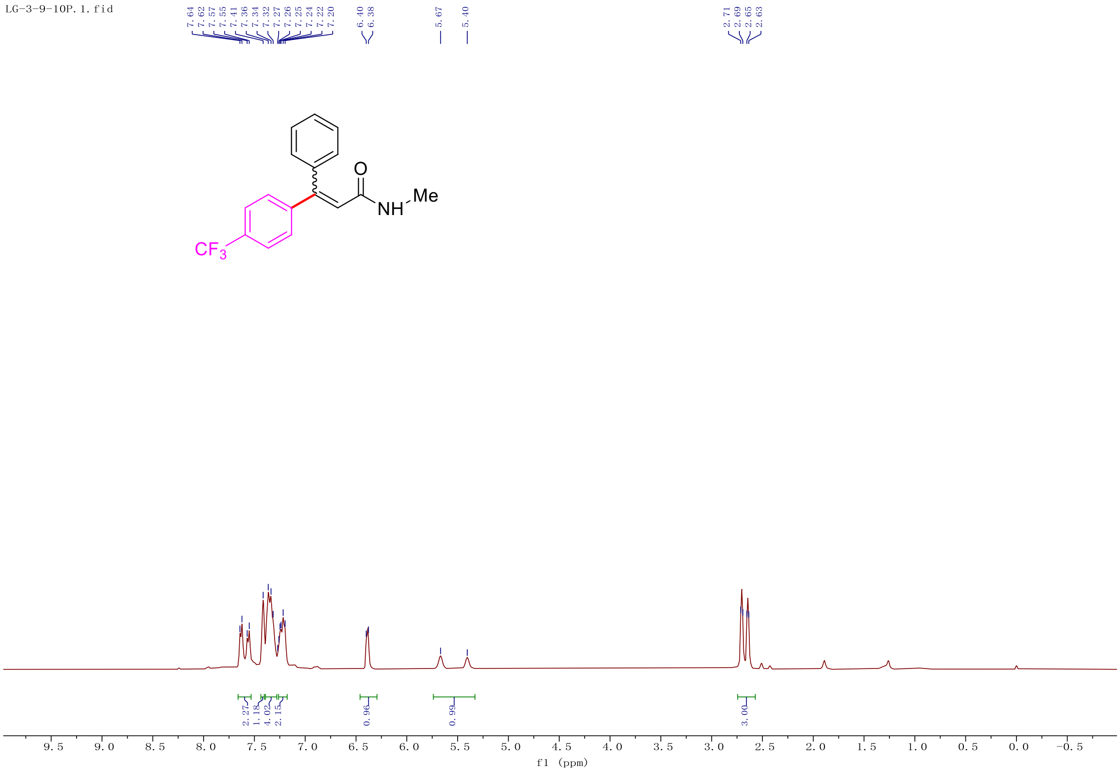

^{13}C {^{1}H} NMR Spectrum of *N*-methyl-3-phenyl-3-(4-(trifluoromethyl)phenyl)acrylamide (84, $CDCl_3$ as solvent, 101 MHz)

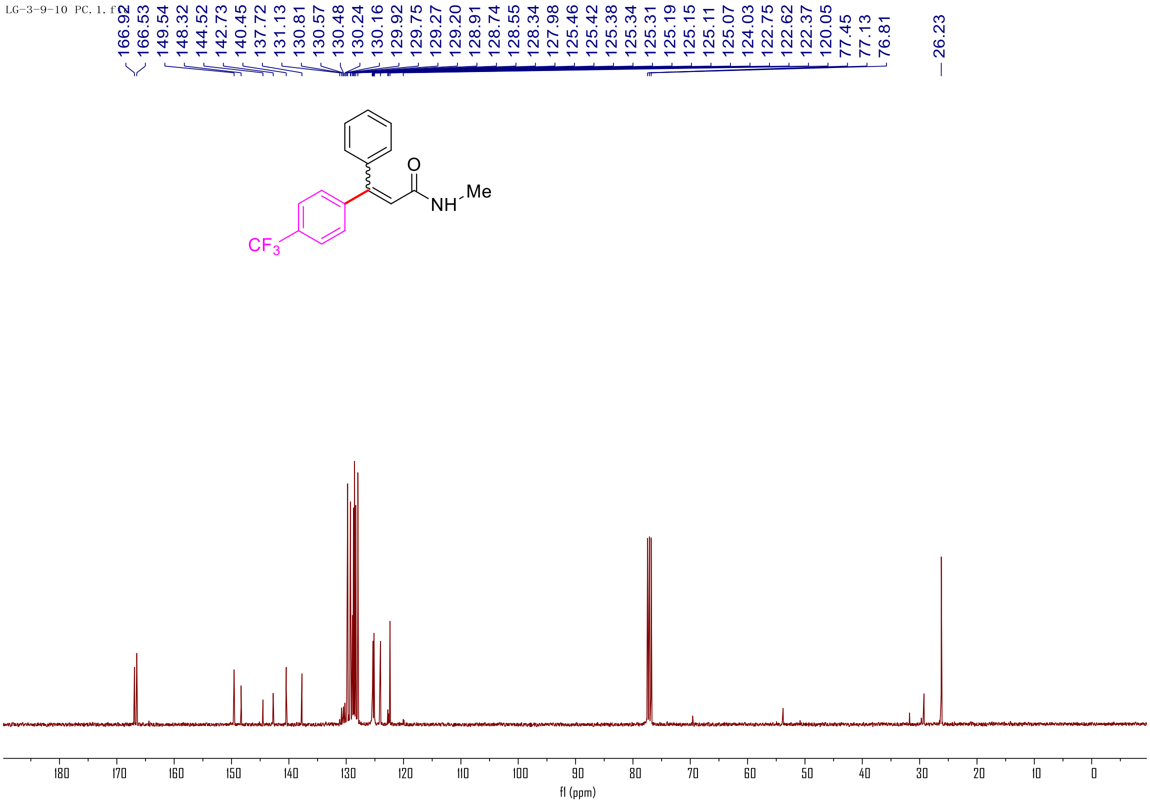

^{19}F NMR Spectrum of *N*-methyl-3-phenyl-3-(4-(trifluoromethyl)phenyl)acrylamide (84, $CDCl_3$ as solvent, 376 MHz)

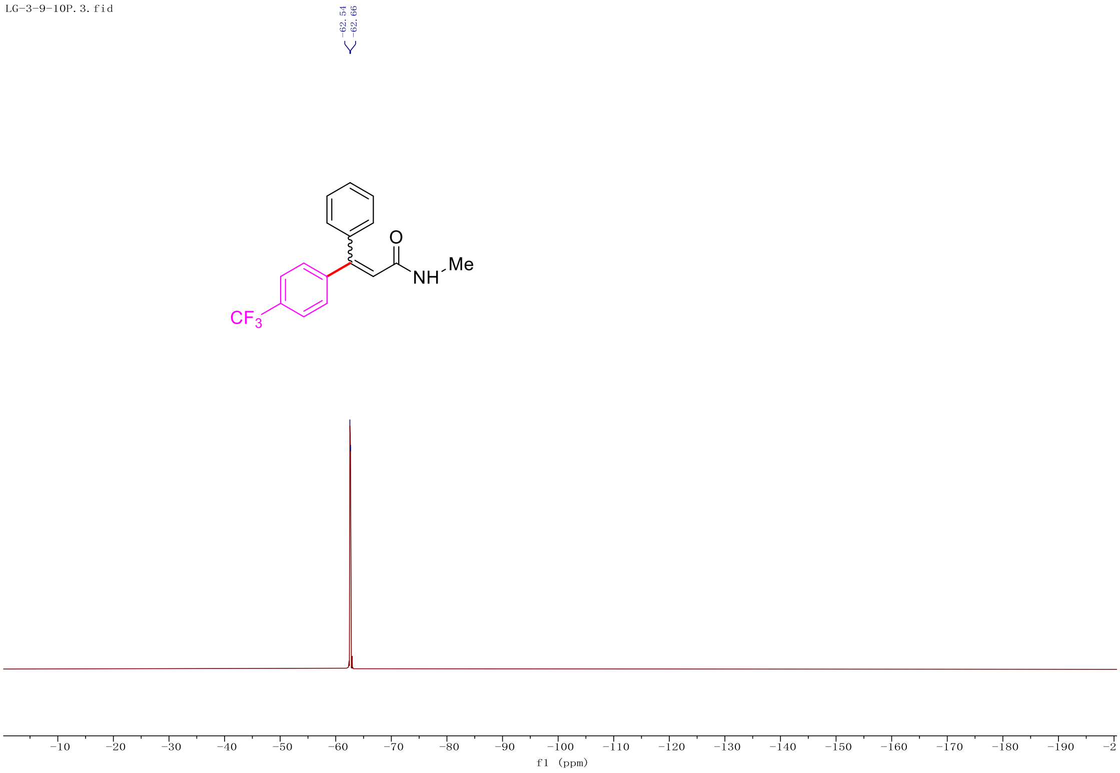

^{1}H NMR Spectrum of methyl 4-(3-(methylamino)-3-oxo-1-phenylprop-1-en-1-yl)benzoate (85, $CDCl_3$ as solvent, 400 MHz)

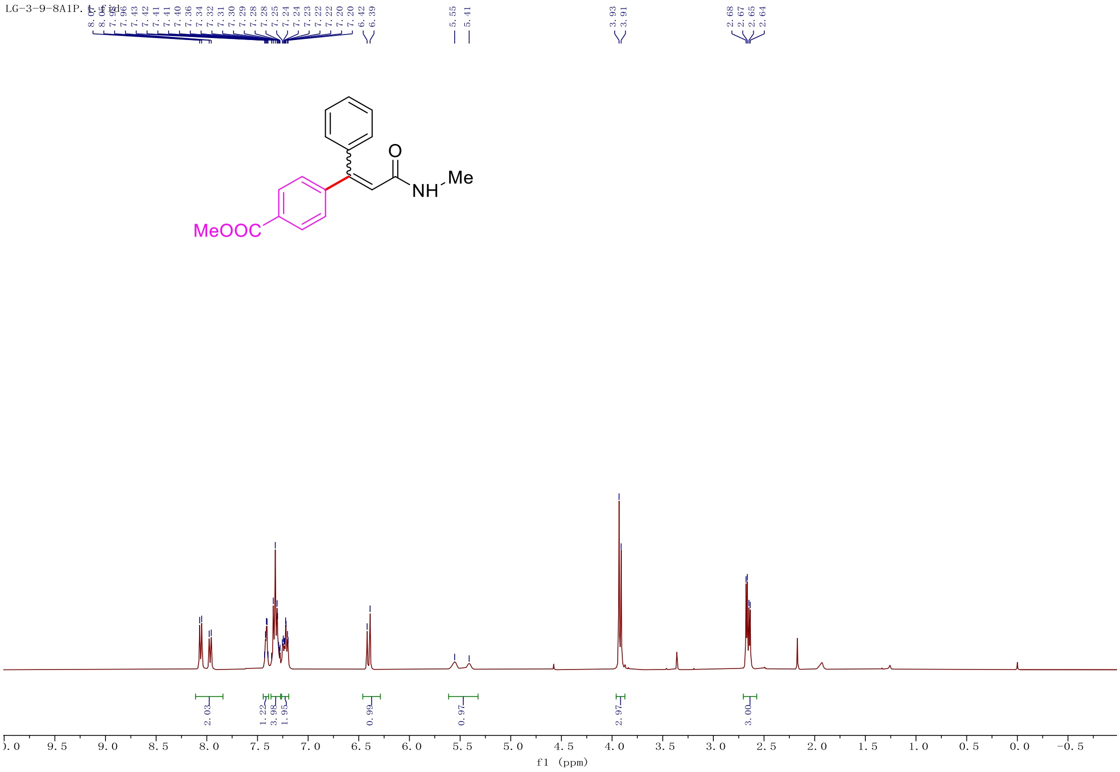

^{13}C {^{1}H} NMR Spectrum of methyl 4-(3-(methylamino)-3-oxo-1-phenylprop-1-en-1-yl)benzoate (85, $CDCl_3$ as solvent, 101 MHz)

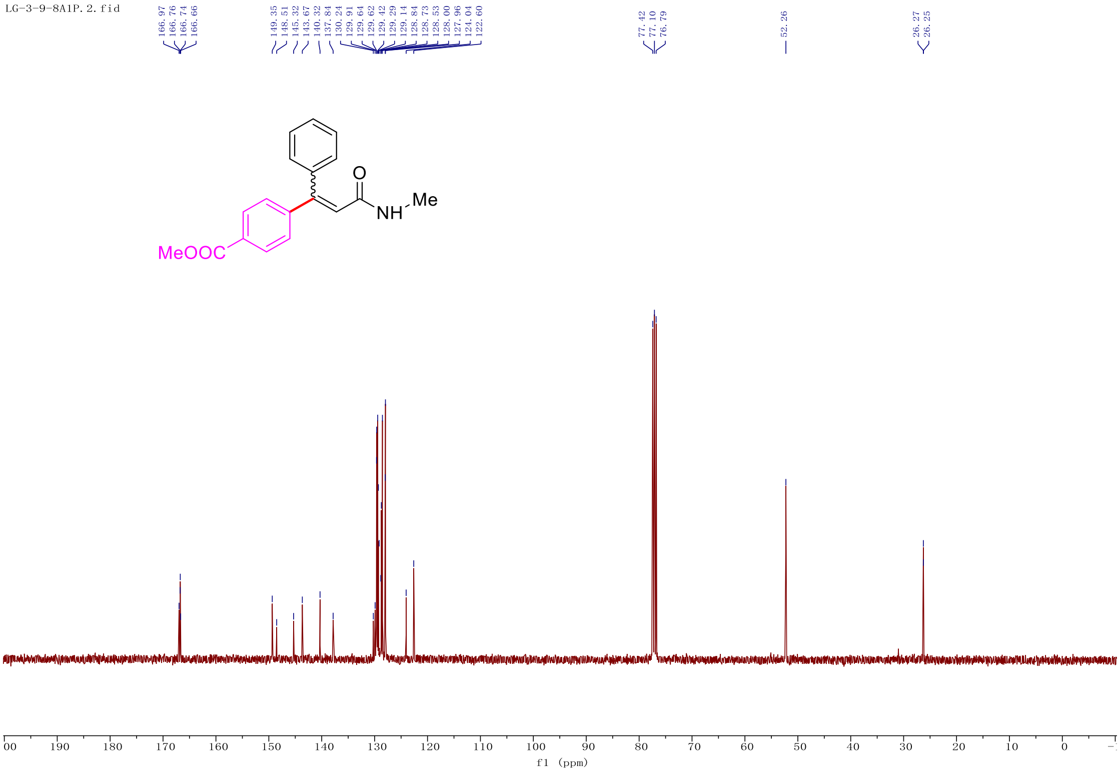

^{1}H NMR Spectrum of 3-(3-bromophenyl)-*N*-methyl-3-phenylacrylamide (86, $CDCl_3$ as solvent, 400 MHz)

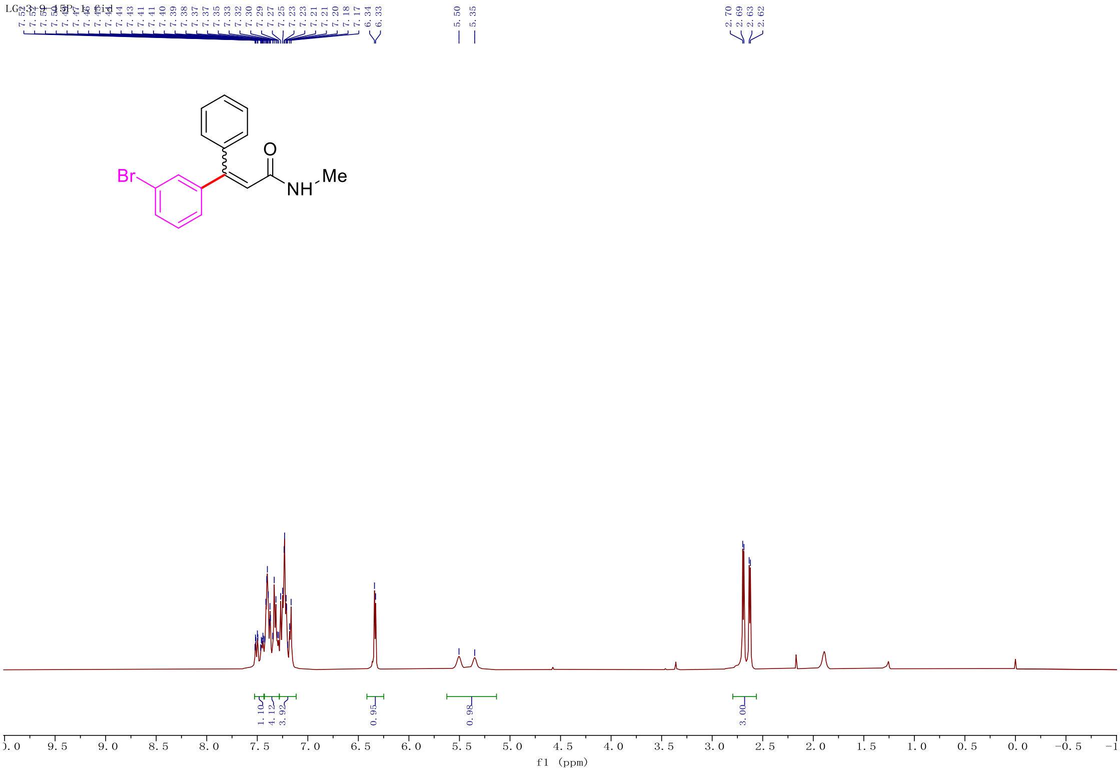

^{1}H NMR Spectrum for *Z/E* isomerization of product 86 ($CDCl_3$ as solvent, 600 MHz)

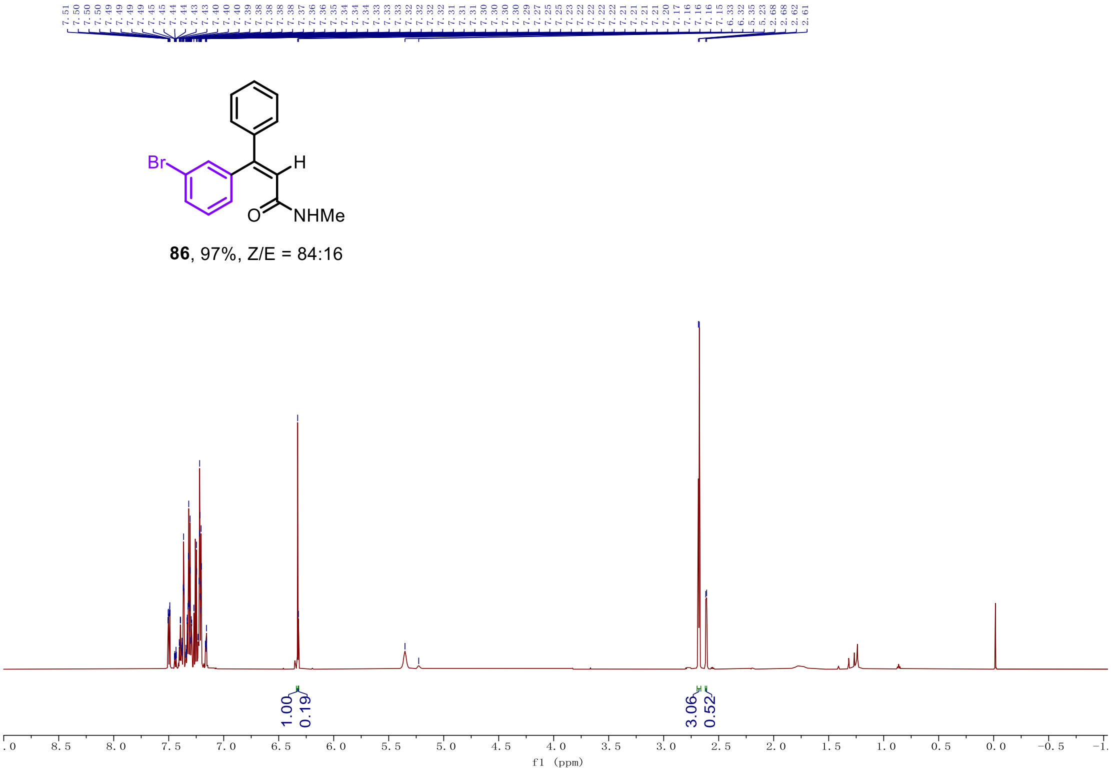

^{13}C {^{1}H} NMR Spectrum of 3-(3-bromophenyl)-*N*-methyl-3-phenylacrylamide (86, $CDCl_3$ as solvent, 101 MHz)

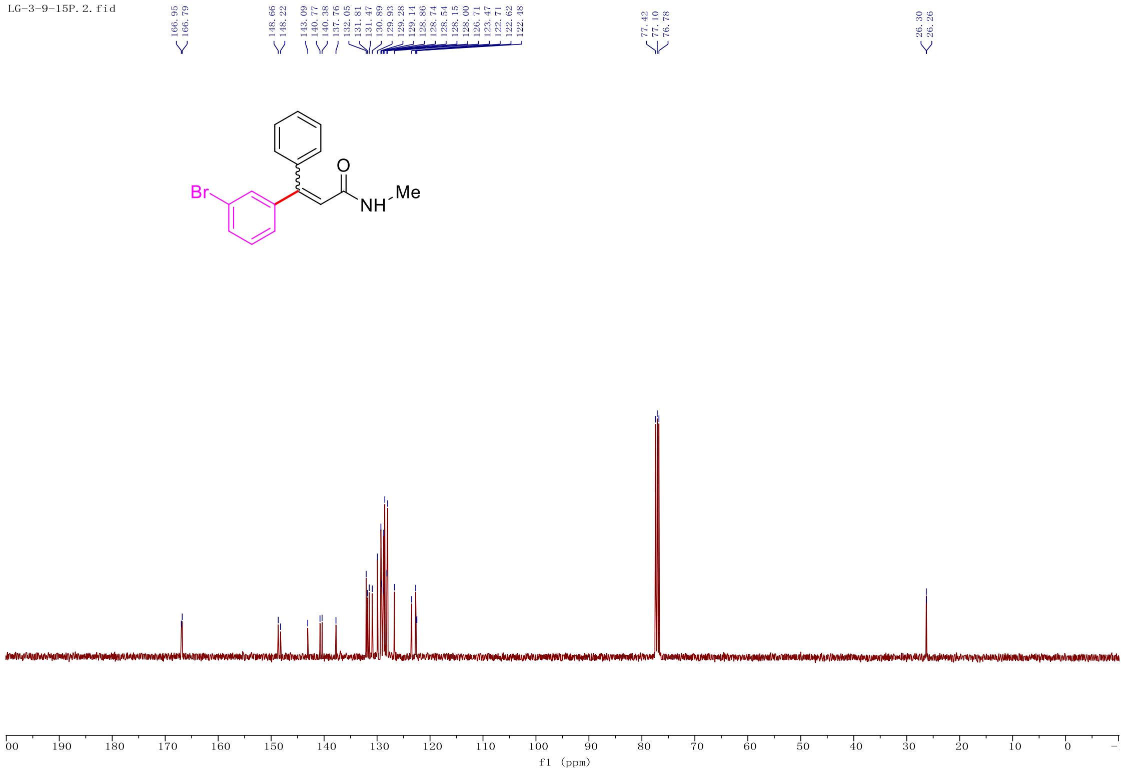

^{1}H NMR Spectrum of 3-(3-iodophenyl)-*N*-methyl-3-phenylacrylamide (87, $CDCl_3$ as solvent, 400 MHz)

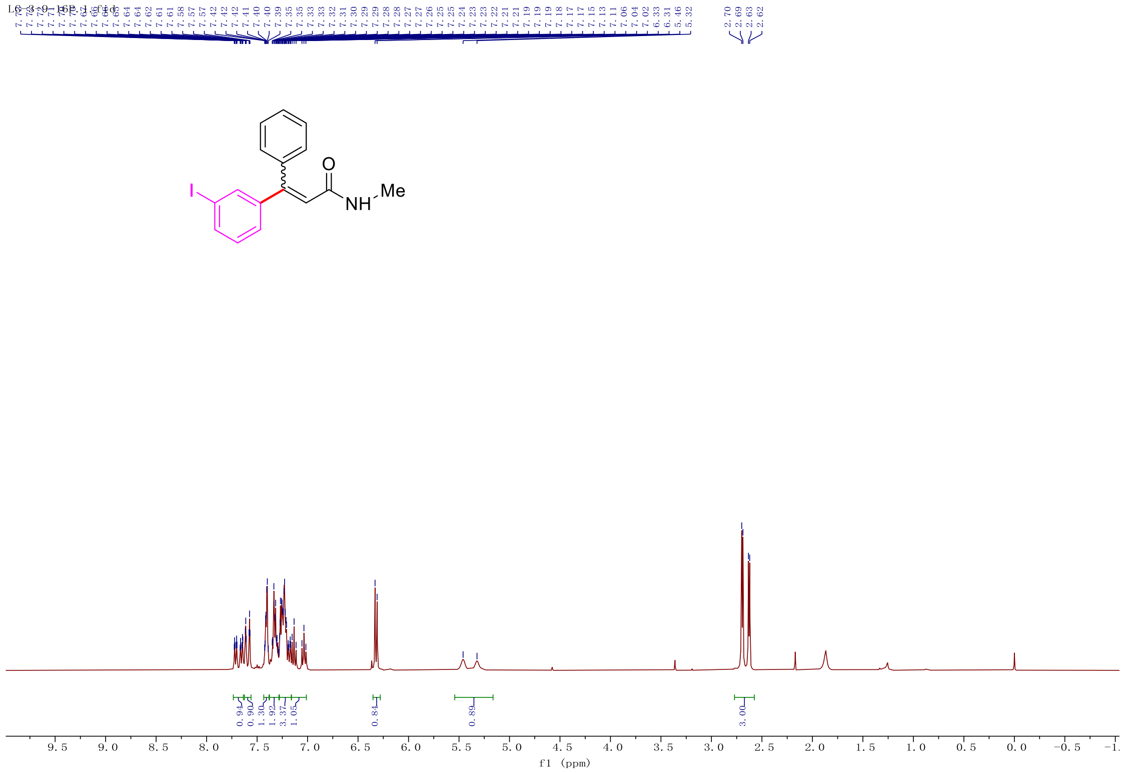

^{1}H NMR Spectrum for *Z/E* isomerization of product 86 ($CDCl_3$ as solvent, 600 MHz)

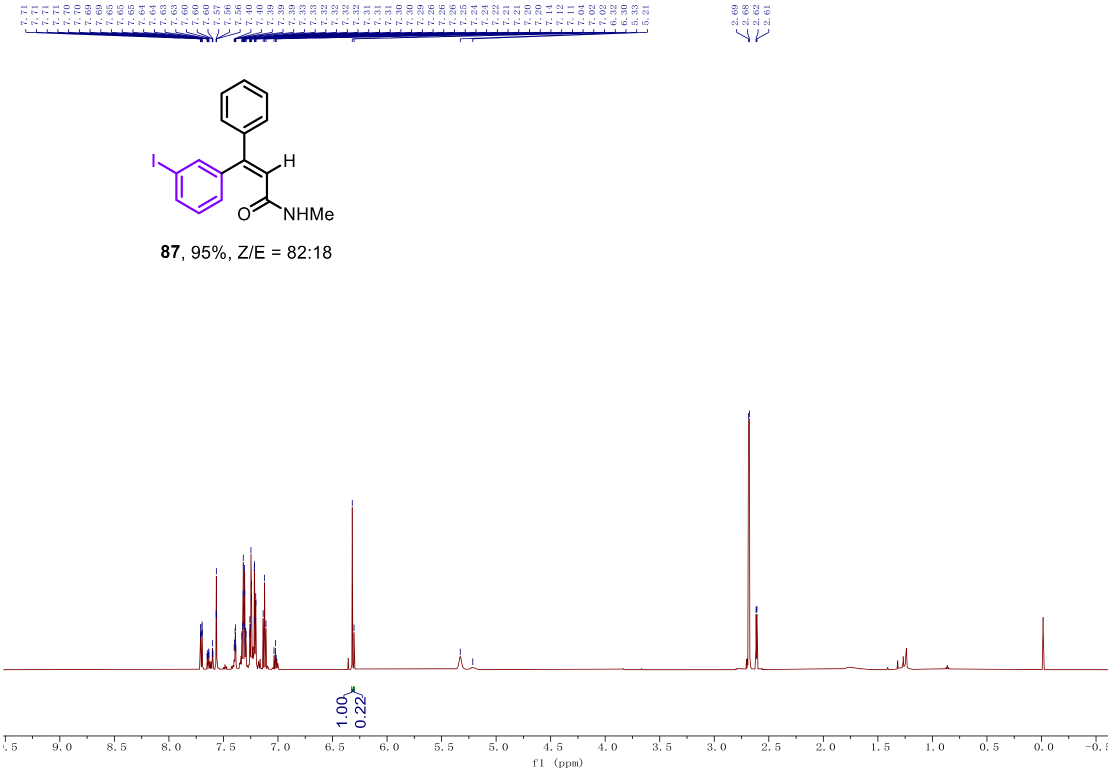

^{13}C {^{1}H} NMR Spectrum of 3-(3-iodophenyl)-*N*-methyl-3-phenylacrylamide (87, $CDCl_3$ as solvent, 101 MHz)

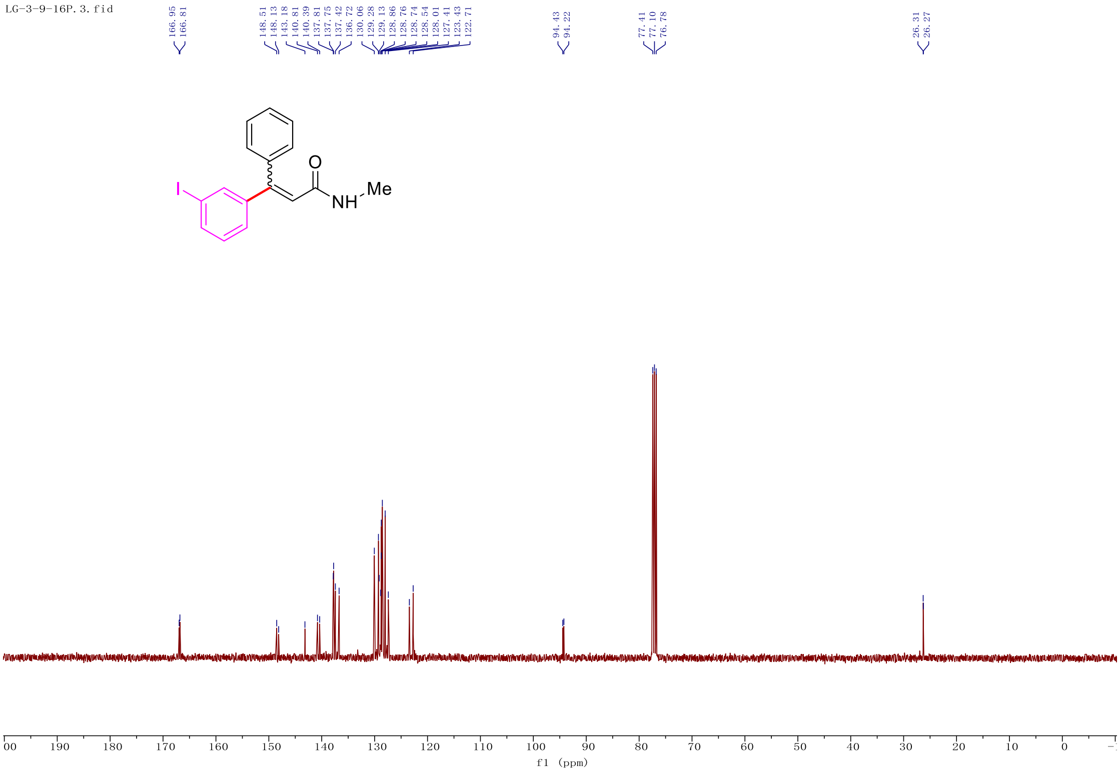

^{1}H NMR Spectrum of 3-(4-chlorophenyl)-*N*-methyl-3-phenylacrylamide (88, $CDCl_3$ as solvent, 400 MHz)

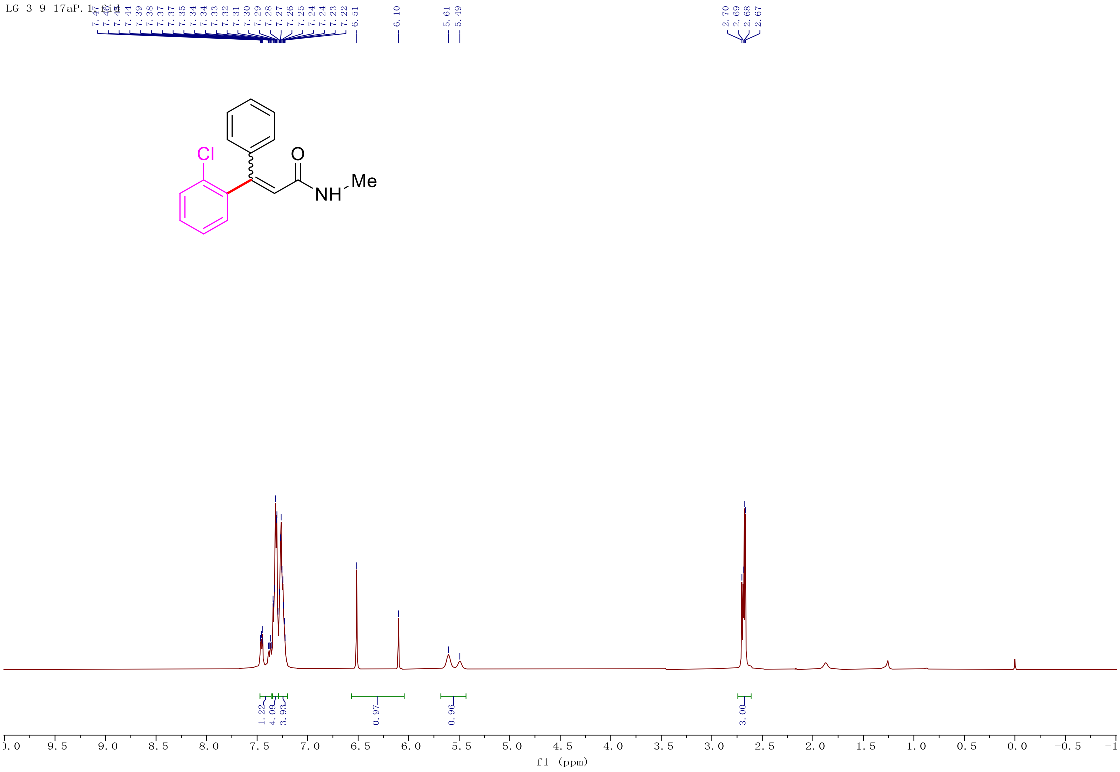

^{13}C {^{1}H} NMR Spectrum of 3-(4-chlorophenyl)-*N*-methyl-3-phenylacrylamide (88, $CDCl_3$ as solvent, 101 MHz)

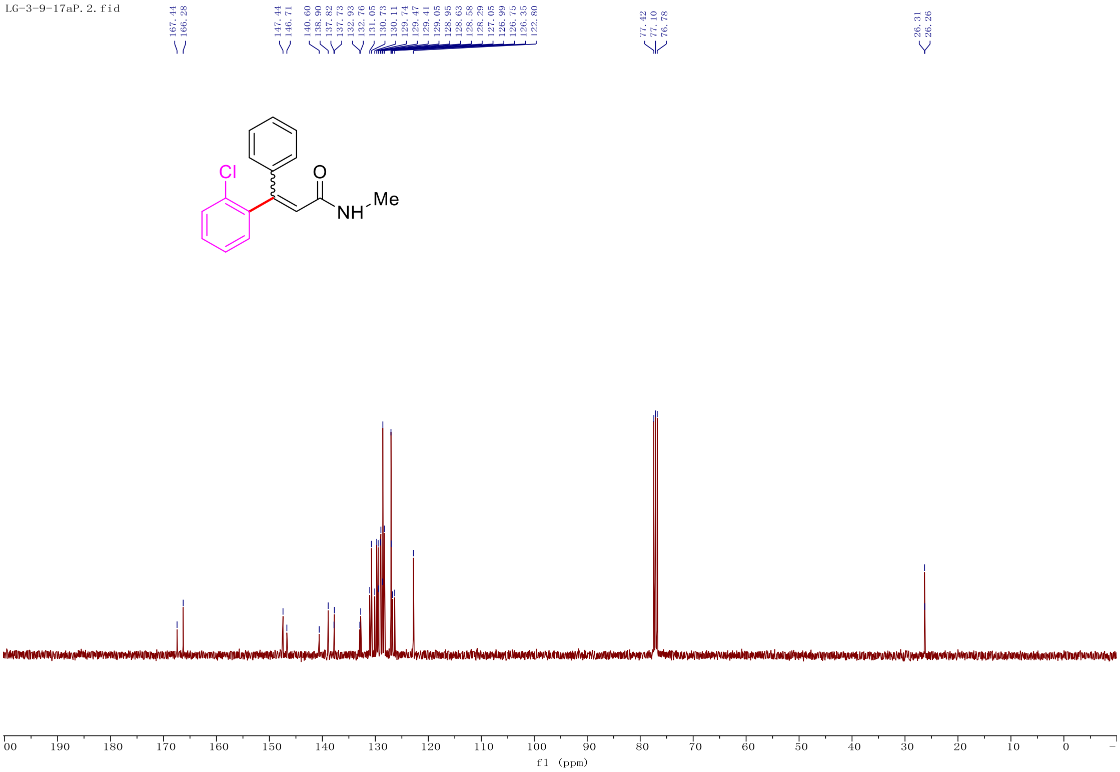

¹H NMR Spectrum of 3-(dibenzo[b,d]furan-3-yl)-*N*-methyl-3-phenylacrylamide (89, $CDCl_3$ as solvent, 400 MHz)

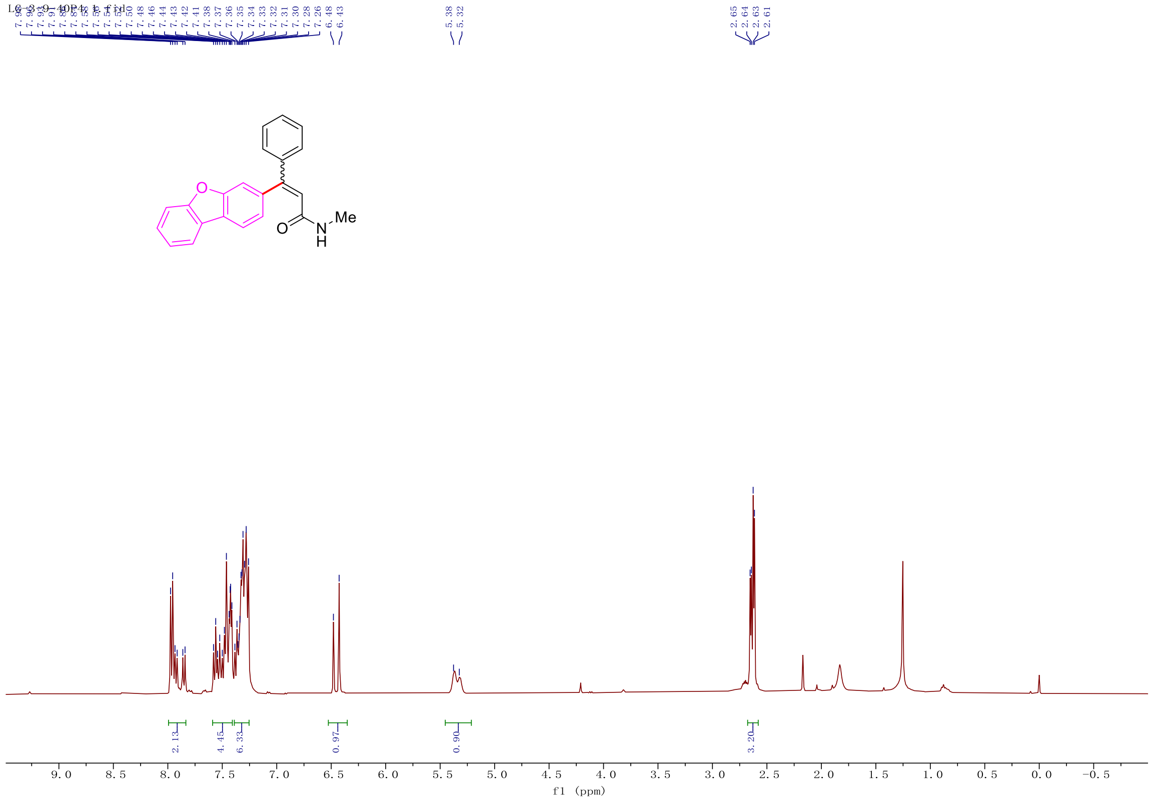

¹H NMR Spectrum for *Z/E* isomerization of product 89 ($CDCl_3$ as solvent, 600 MHz)

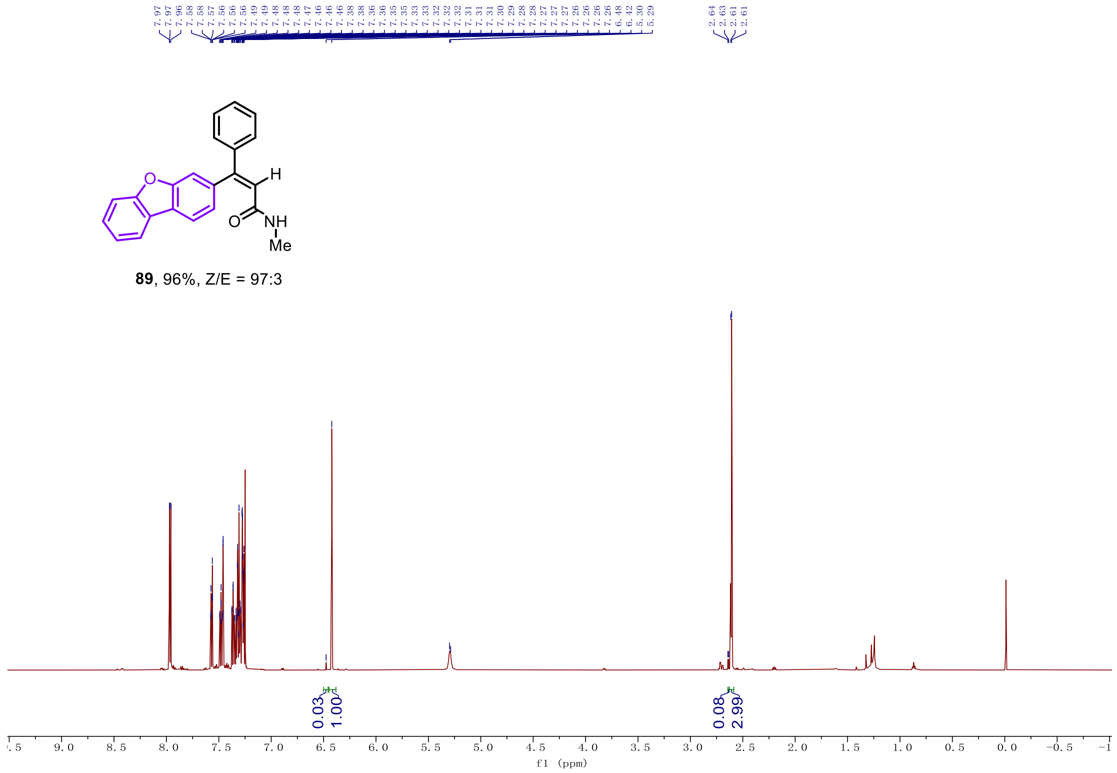

^{13}C {^{1}H} NMR Spectrum of 3-(dibenzo[b,d]furan-3-yl)-*N*-methyl-3-phenylacrylamide (89, $CDCl_3$ as solvent, 101 MHz)

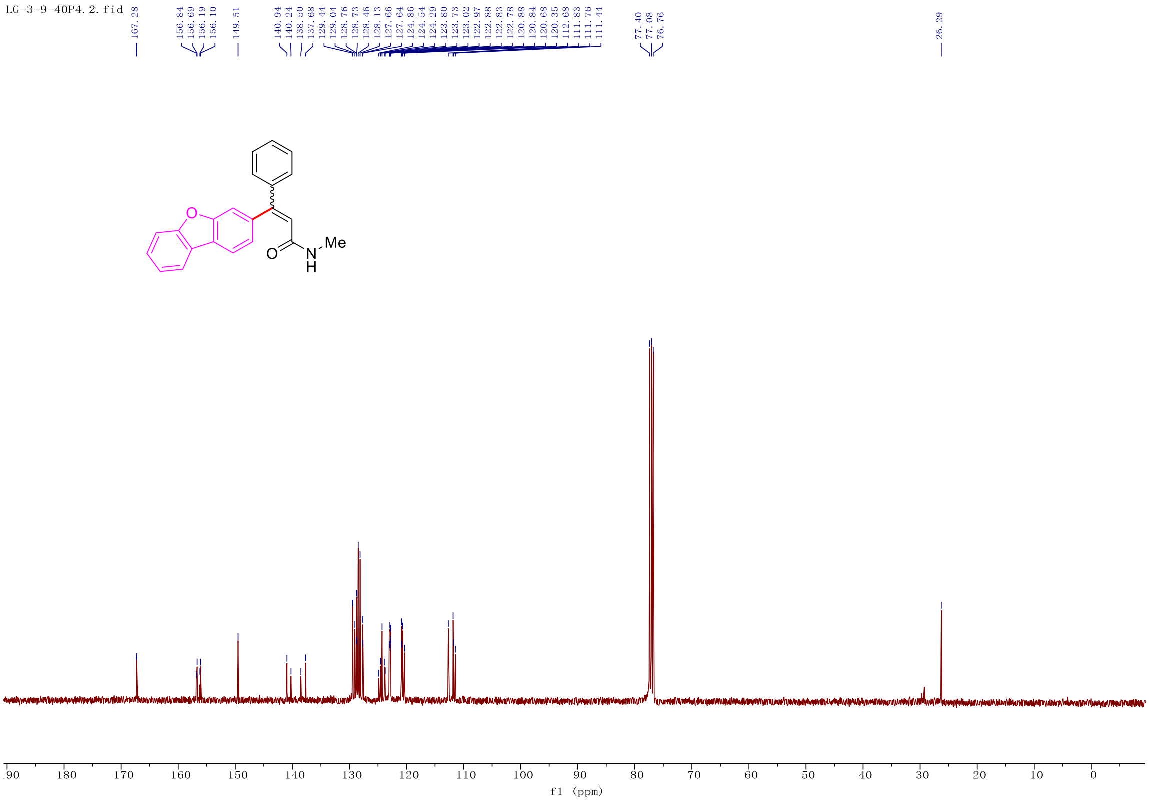

^{1}H NMR Spectrum of 3-(4-bromophenyl)-*N*-methyl-3-(pyridin-3-yl)acrylamide (90, $CDCl_3$ as solvent, 400 MHz)

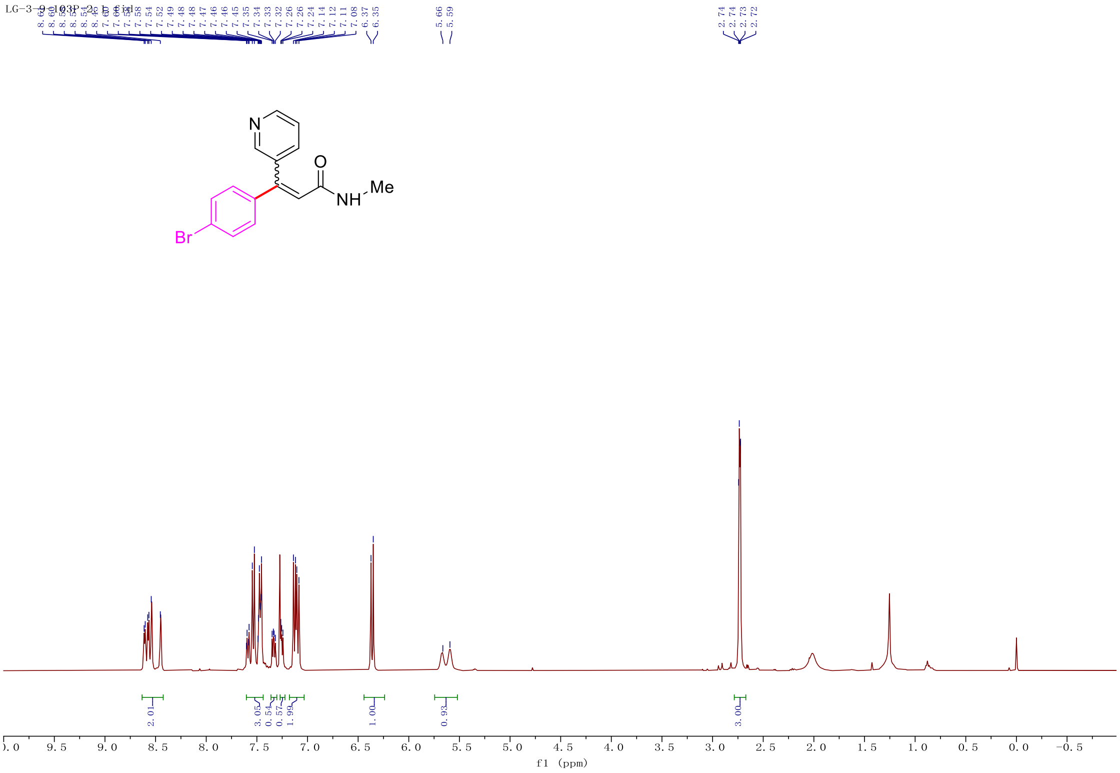

^{13}C {^{1}H} NMR Spectrum of 3-(4-bromophenyl)-*N*-methyl-3-(pyridin-3-yl)acrylamide (90, $CDCl_3$ as solvent, 101 MHz)

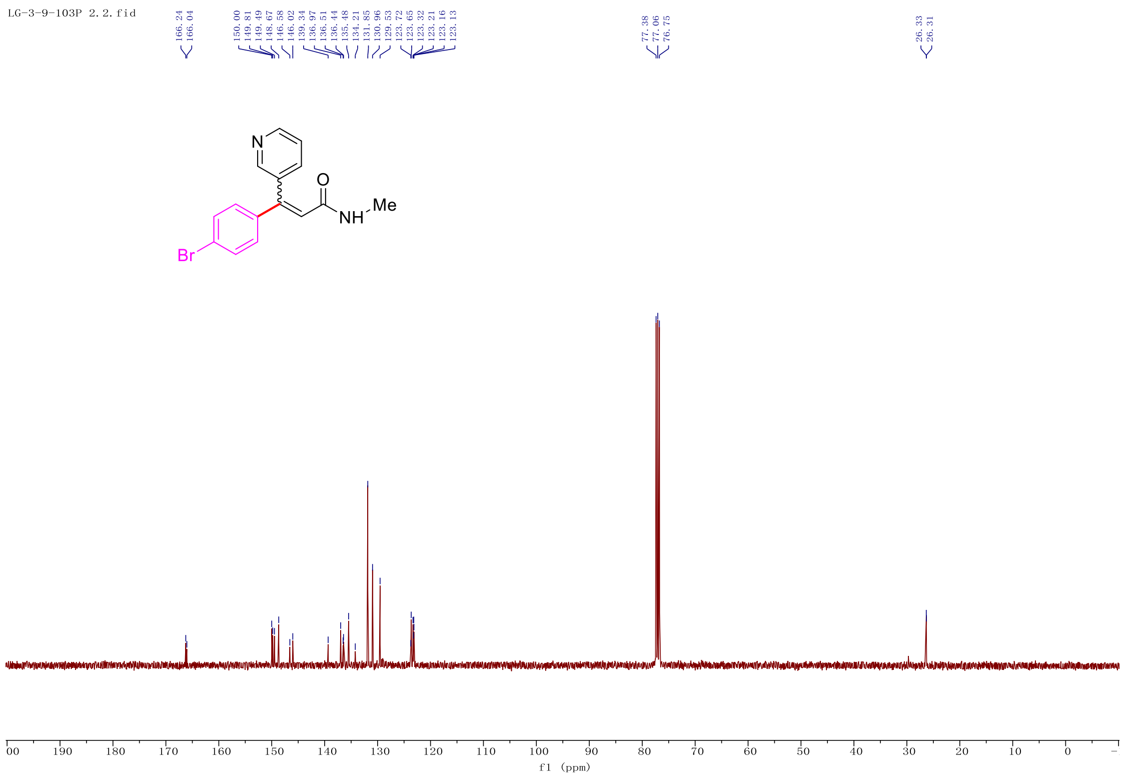

^{1}H NMR Spectrum of *N*-methyl-3,3-diphenyl-2-(trifluoromethyl) acrylamide (91, $CDCl_3$ as solvent, 400 MHz)

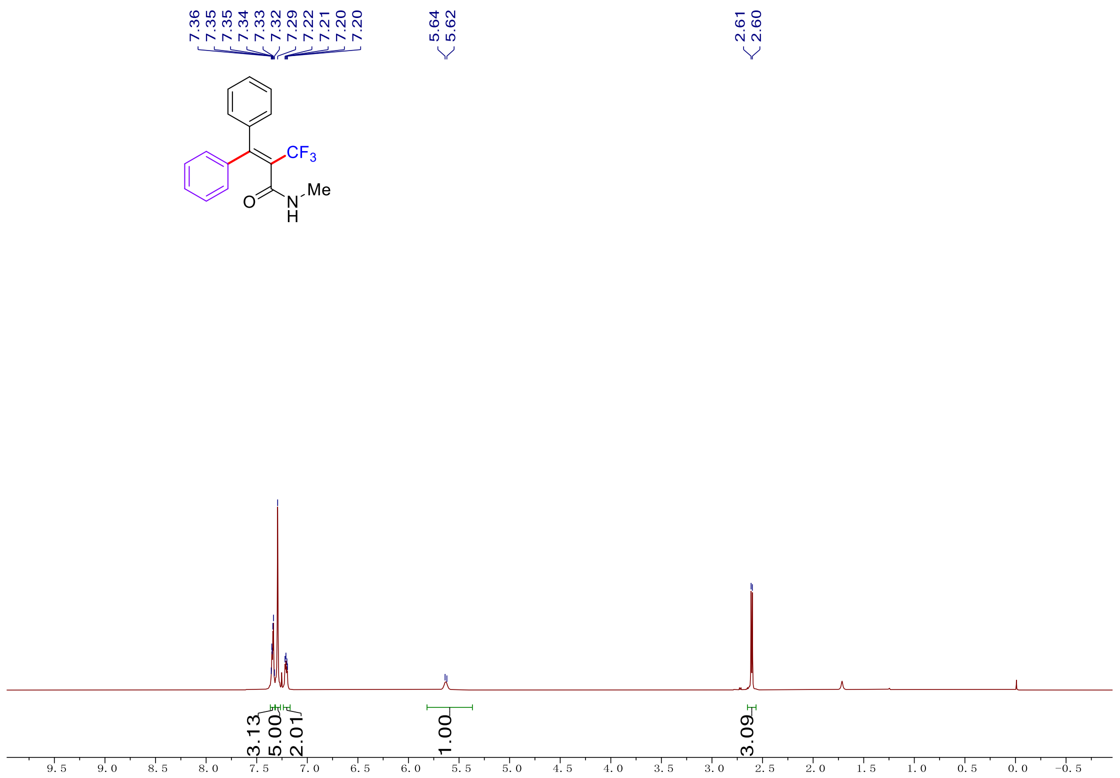

^{13}C {^{1}H} NMR Spectrum of *N*-methyl-3,3-diphenyl-2-(trifluoromethyl) acrylamide (91, $CDCl_3$ as solvent, 101 MHz)

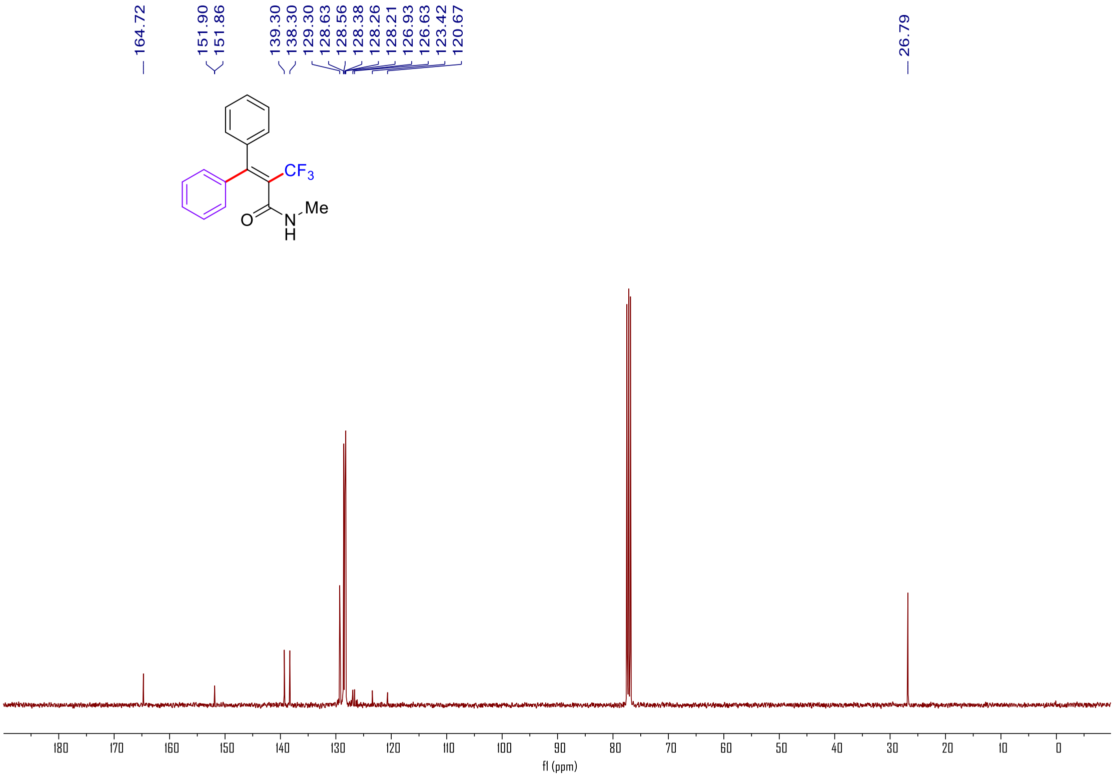

^{19}F NMR Spectrum of *N*-methyl-3,3-diphenyl-2-(trifluoromethyl) acrylamide (91, $CDCl_3$ as solvent, 376 MHz)

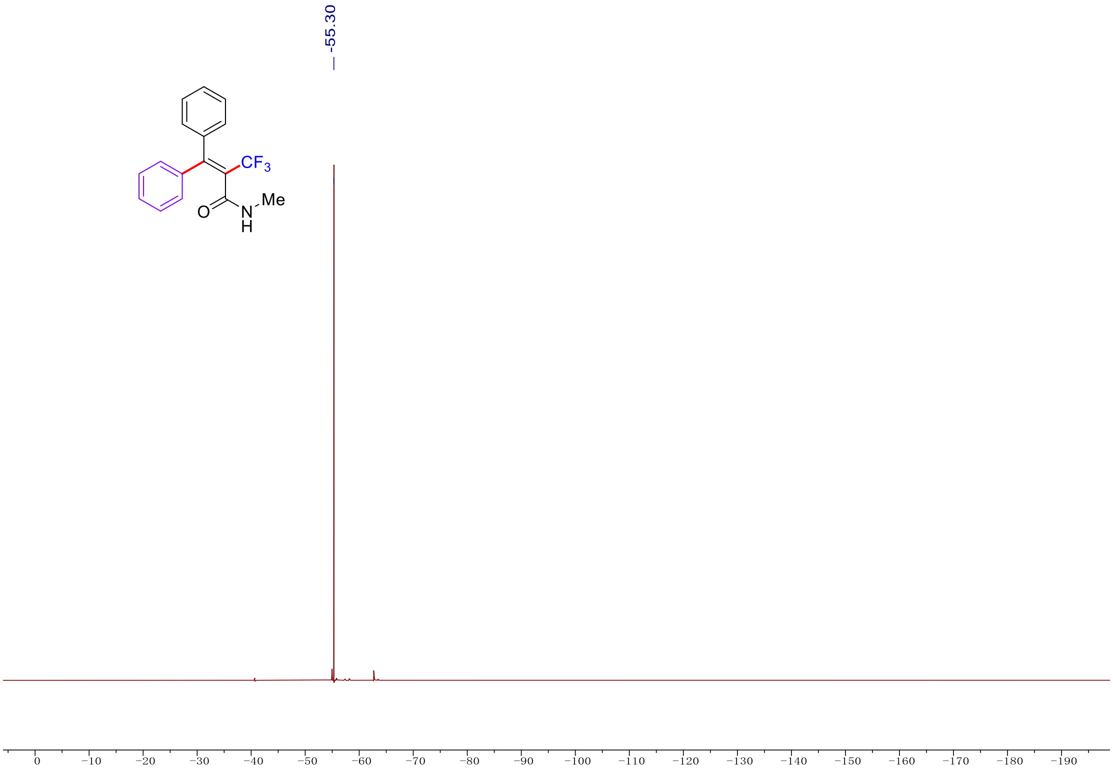

^{1}H NMR Spectrum of (*E*)-*N*-methyl-3-phenyl-3-(p-tolyl)-2-(trifluoromethyl) acrylamide (92, $CDCl_3$ as solvent, 400 MHz)

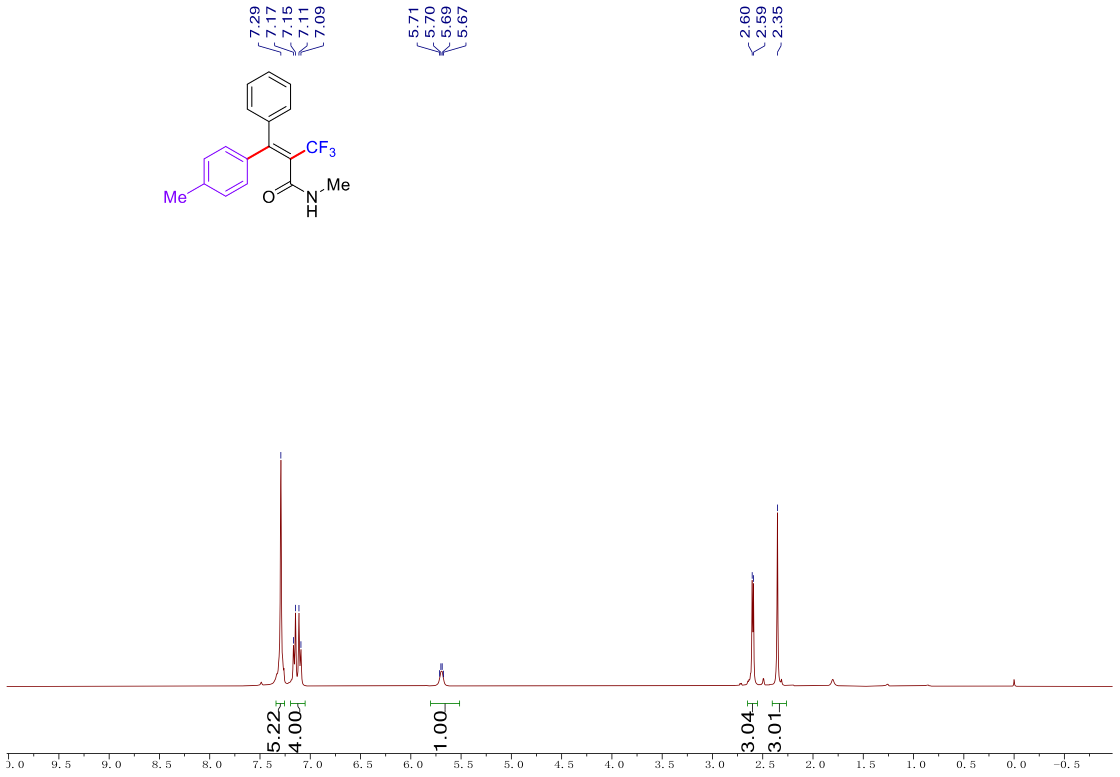

^{13}C {^{1}H} NMR Spectrum of (*E*)-*N*-methyl-3-phenyl-3-(p-tolyl)-2-(trifluoromethyl) acrylamide (92, $CDCl_3$ as solvent, 101 MHz)

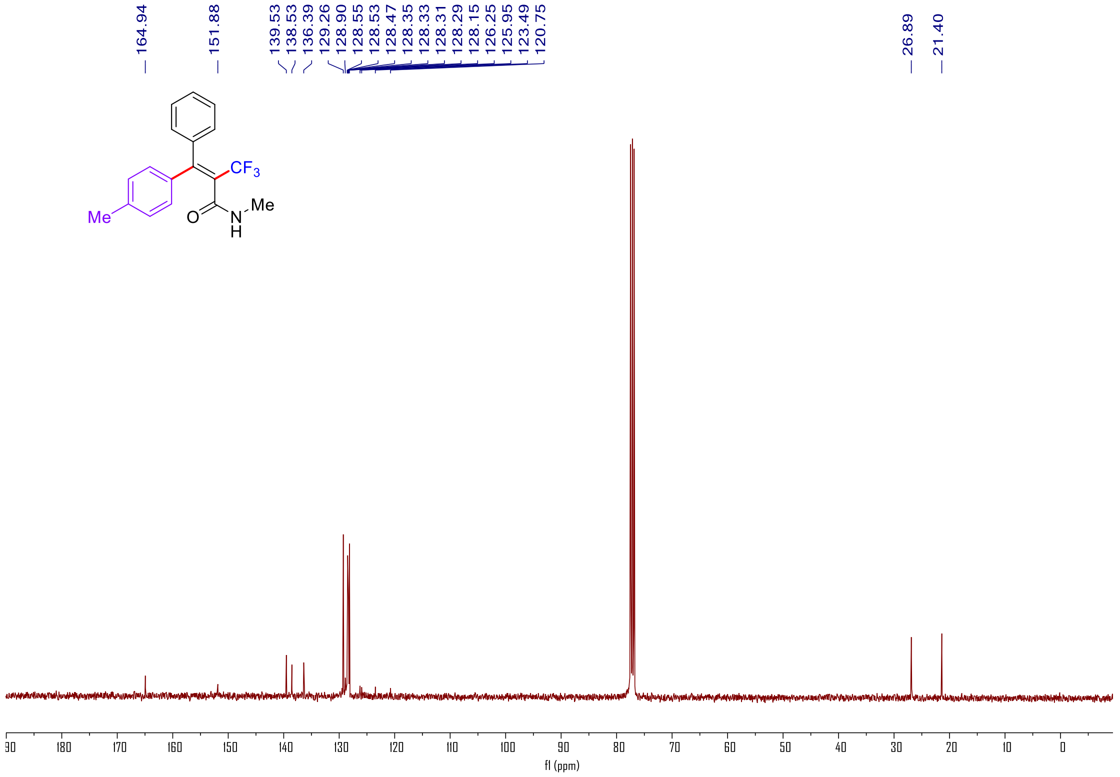

^{19}F NMR Spectrum of (*E*)-*N*-methyl-3-phenyl-3-(p-tolyl)-2-(trifluoromethyl) acrylamide (92, $CDCl_3$ as solvent, 376 MHz)

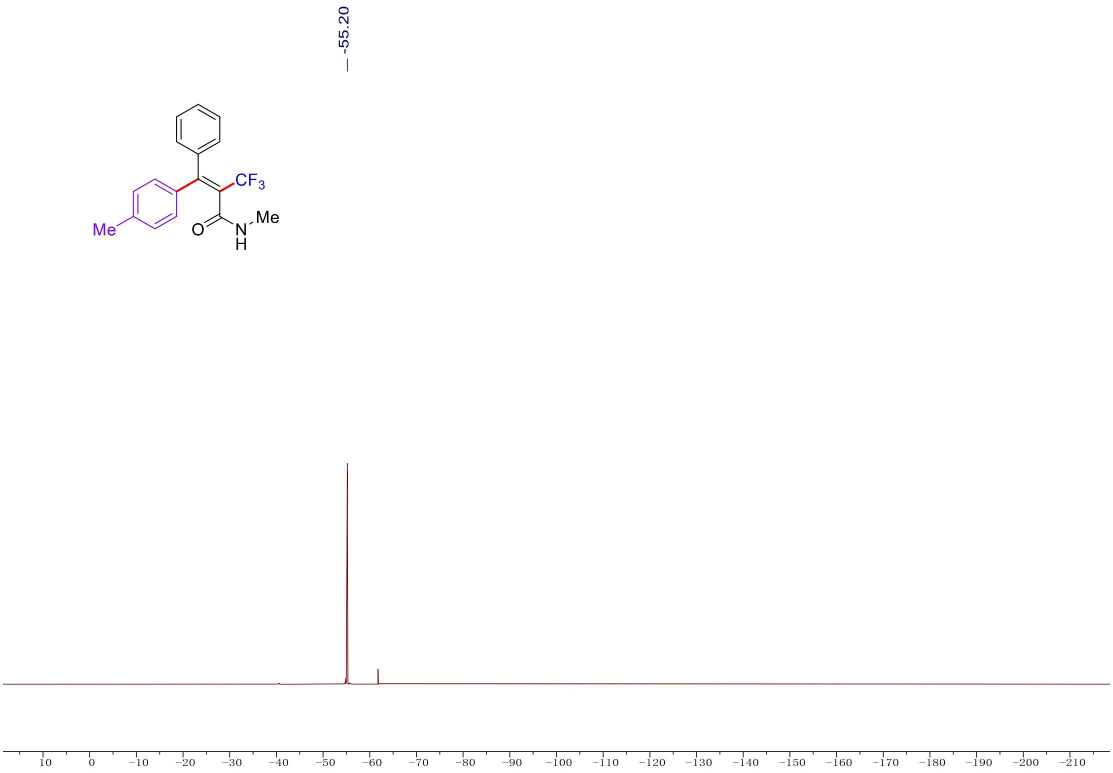

^{1}H NMR Spectrum of (*E*)-3-(3-iodophenyl)-*N*-methyl-3-phenyl-2-(trifluoromethyl)acrylamide (93, $CDCl_3$ as solvent, 400 MHz)

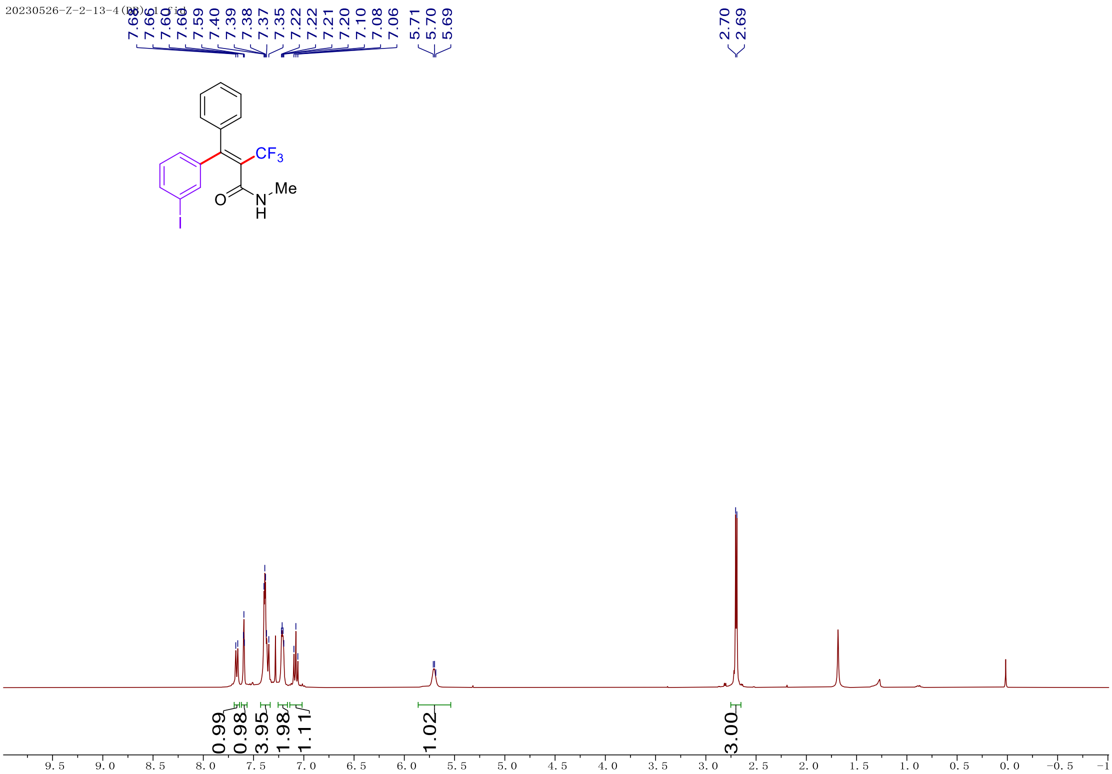

^{13}C {^{1}H} NMR Spectrum of (*E*)-3-(3-iodophenyl)-*N*-methyl-3-phenyl-2-(trifluoromethyl)acrylamide (93, $CDCl_3$ as solvent, 101 MHz)

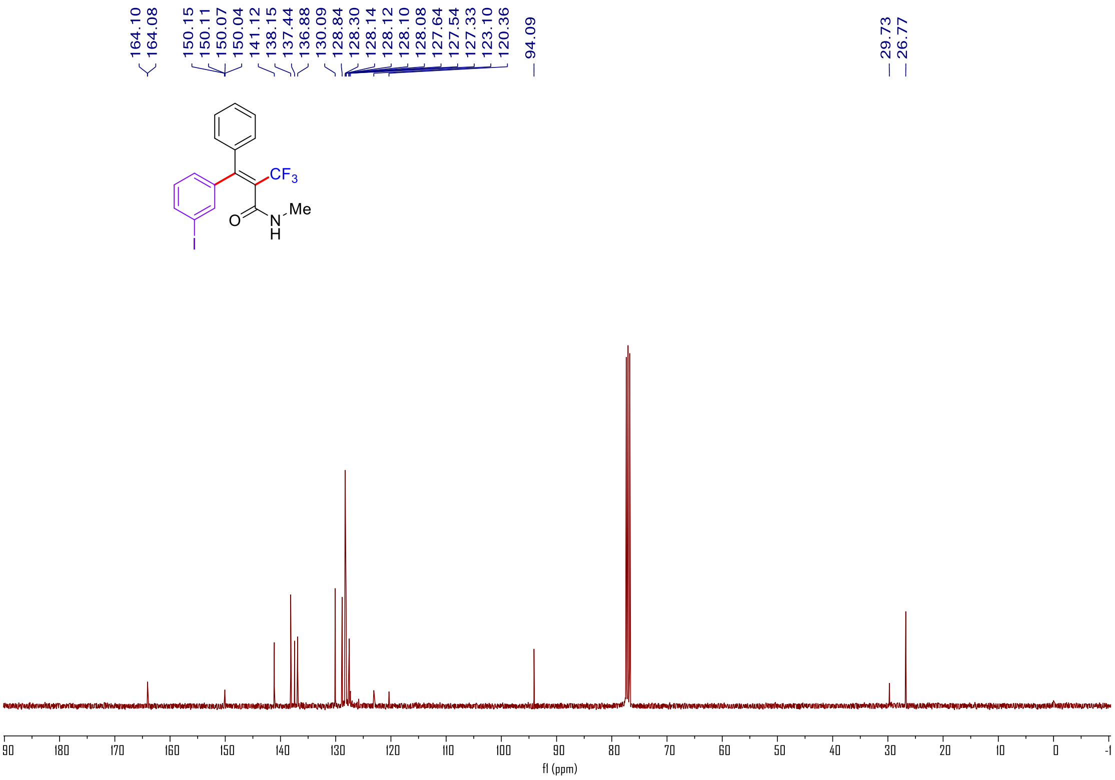

^{19}F NMR Spectrum of (*E*)-3-(3-iodophenyl)-*N*-methyl-3-phenyl-2-(trifluoromethyl)acrylamide (93, $CDCl_3$ as solvent, 376 MHz)

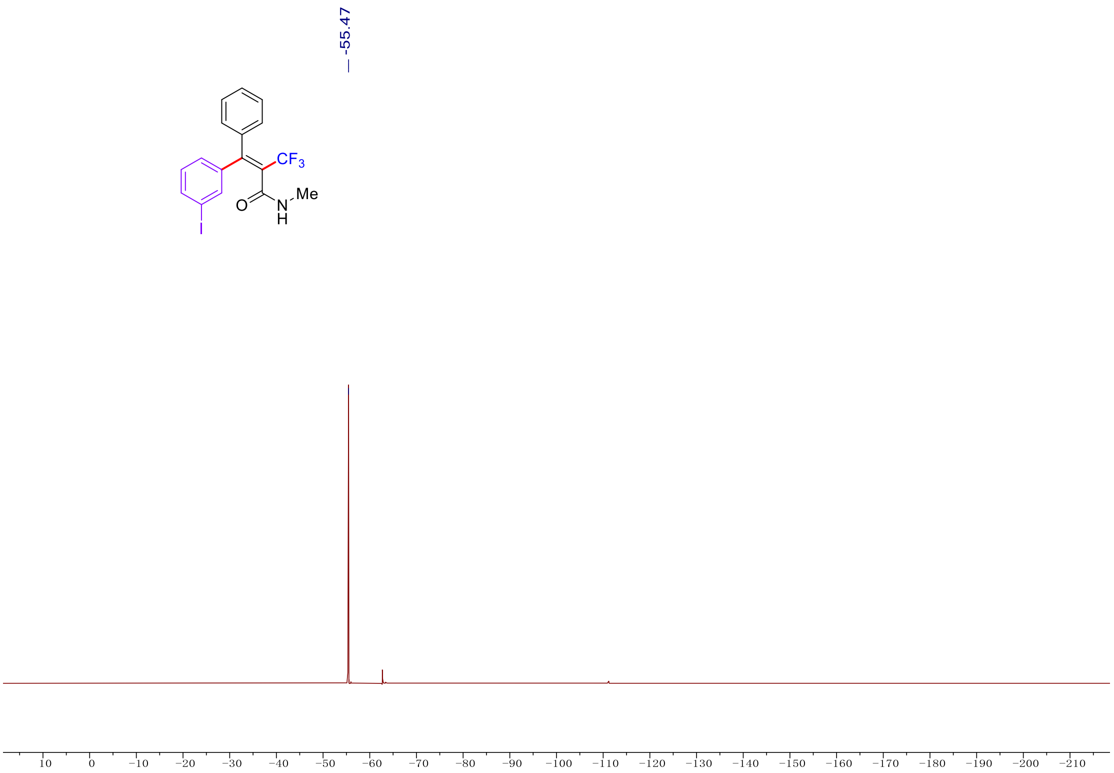

^{1}H NMR Spectrum of (*Z*)-*N*-methyl-3-phenyl-3-(p-tolyl)-2-(trifluoromethyl)acrylamide (94, $CDCl_3$ as solvent, 400 MHz)

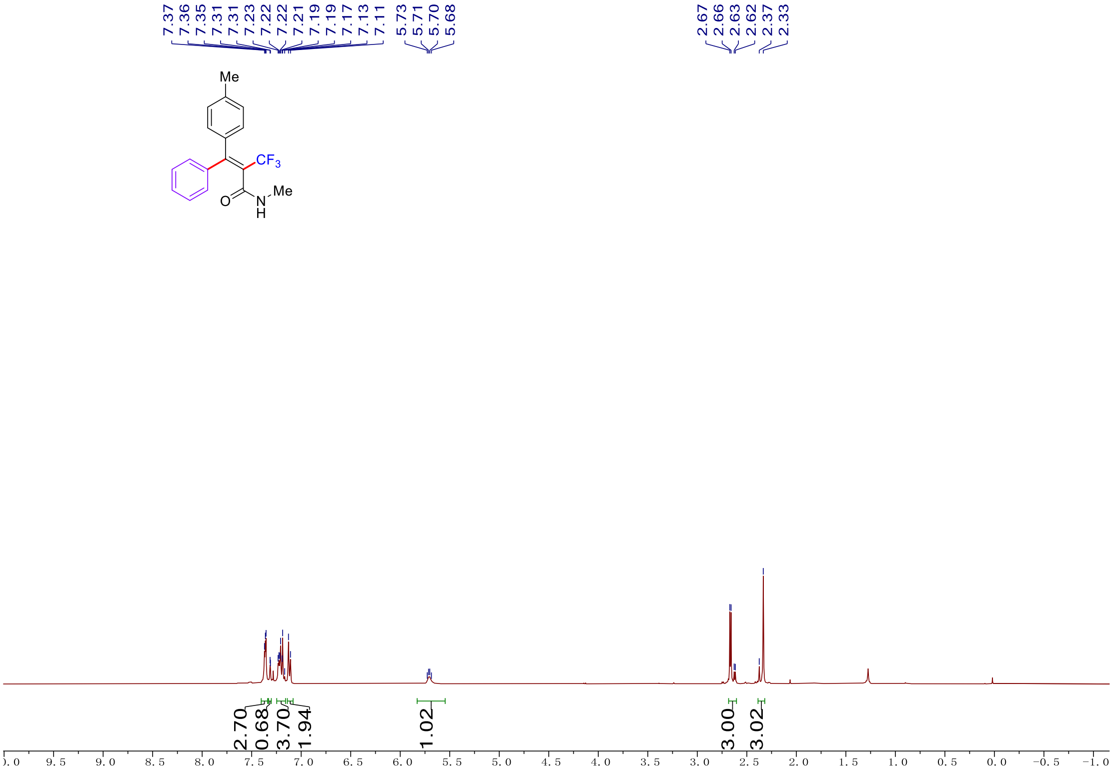

^{13}C {^{1}H} NMR Spectrum of (*Z*)-*N*-methyl-3-phenyl-3-(p-tolyl)-2-(trifluoromethyl)acrylamide (94, $CDCl_3$ as solvent, 101 MHz)

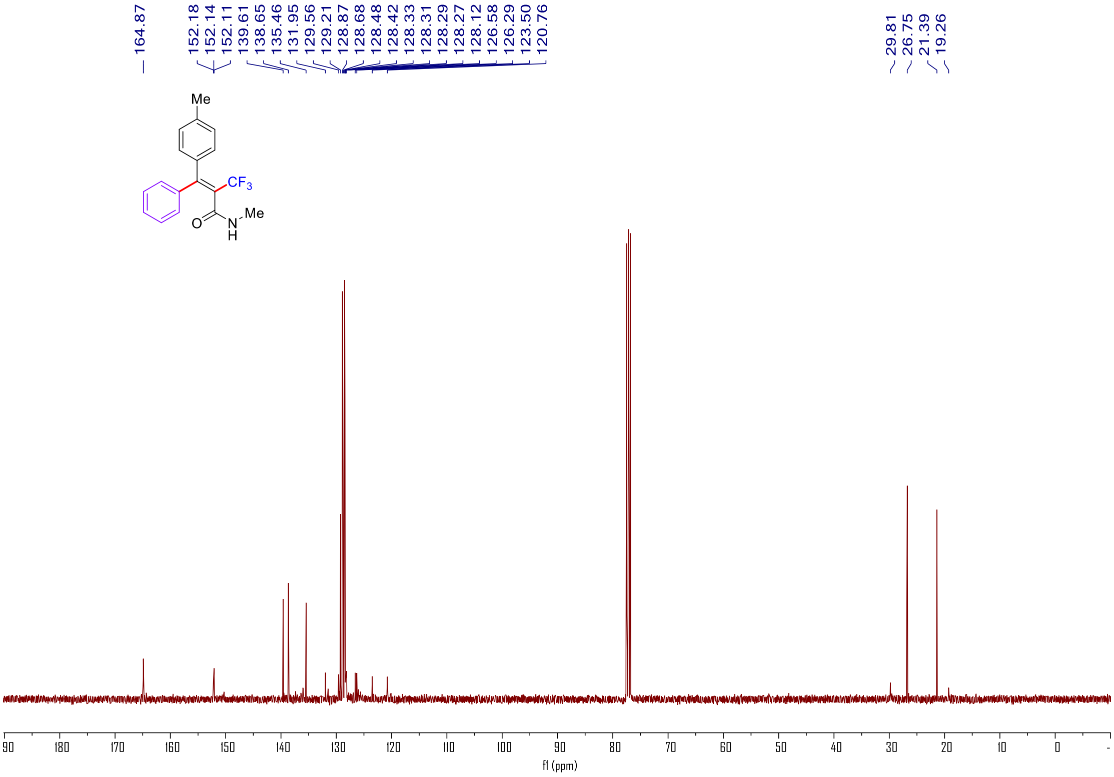

^{19}F NMR Spectrum of (*Z*)-*N*-methyl-3-phenyl-3-(p-tolyl)-2-(trifluoromethyl)acrylamide (94, $CDCl_3$ as solvent, 376 MHz)

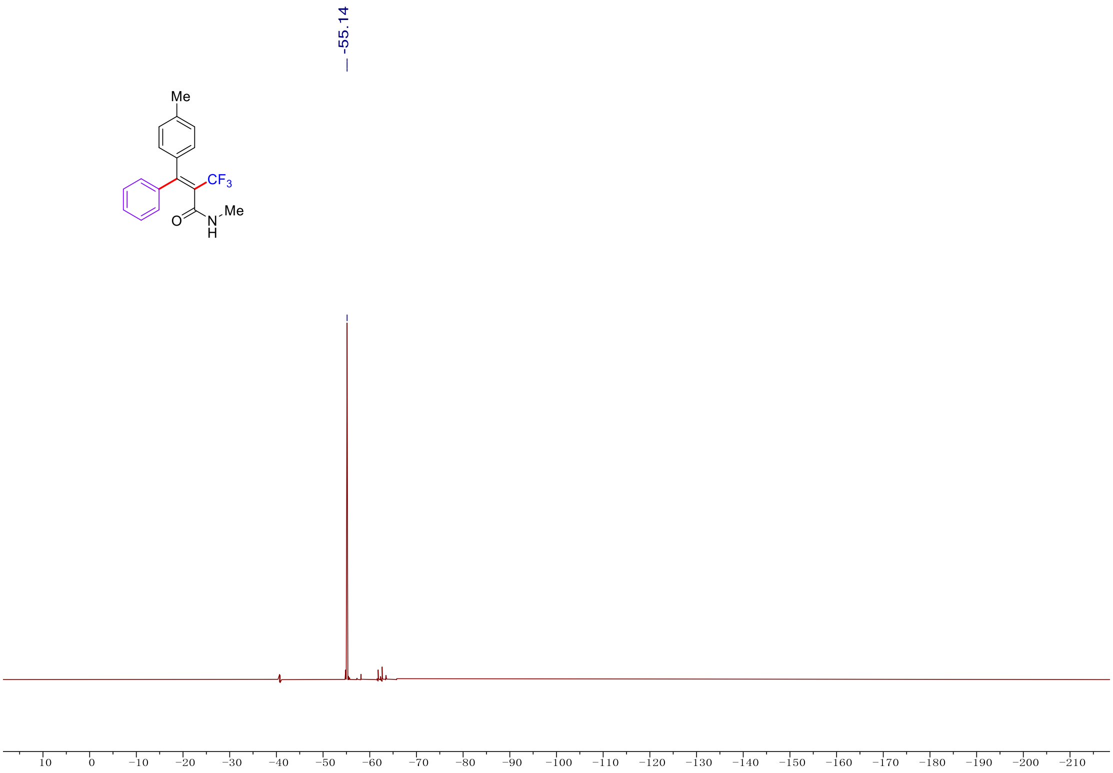

^{1}H NMR Spectrum of methyl (*Z*)-4-(3,3,3-trifluoro-2-(methylcarbamoyl)-1-phenylprop-1-en-1-yl)benzoate (95, $CDCl_3$ as solvent, 400 MHz)

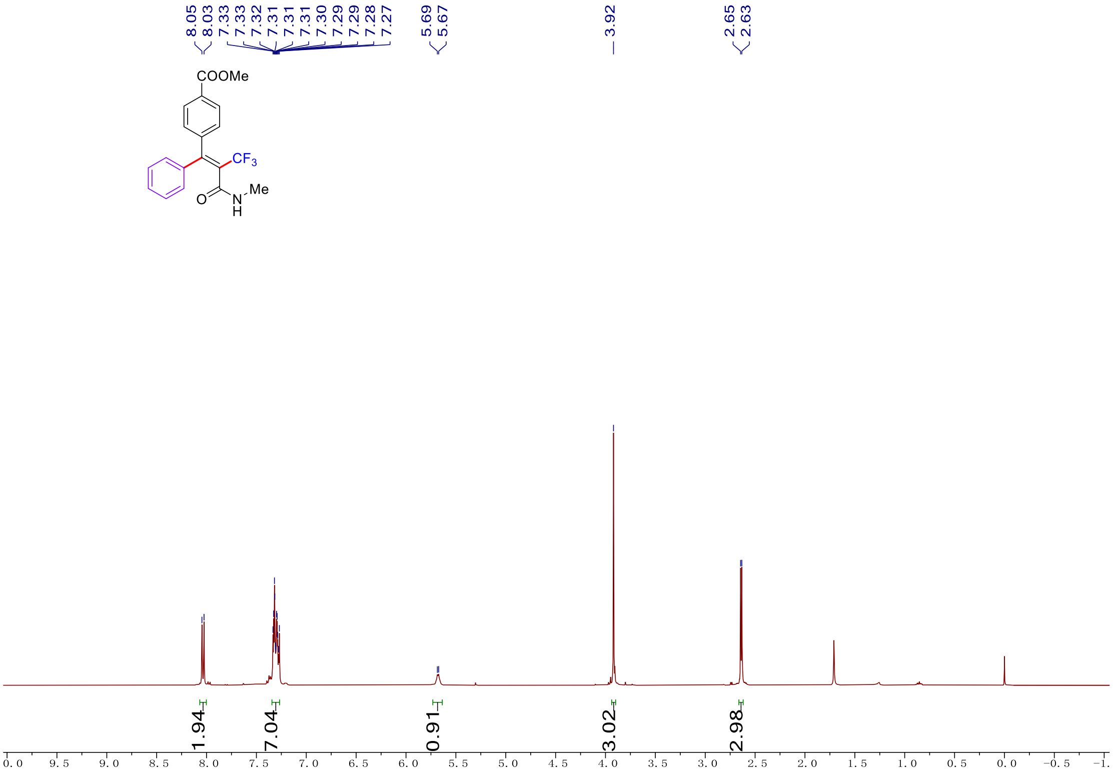

^{13}C {^{1}H} NMR Spectrum of (*Z*)-4-(3,3,3-trifluoro-2-(methylcarbamoyl)-1-phenylprop-1-en-1-yl)benzoate (95, $CDCl_3$ as solvent, 101 MHz)

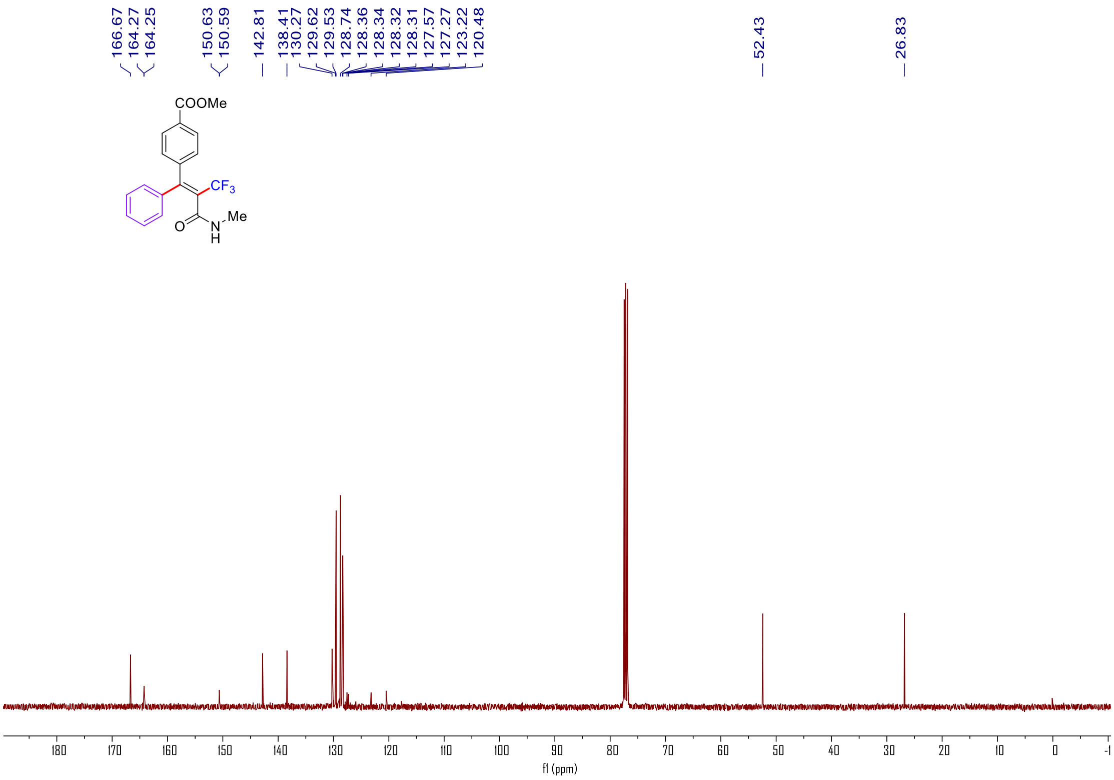

^{19}F NMR Spectrum of (*Z*)-4-(3,3,3-trifluoro-2-(methylcarbamoyl)-1-phenylprop-1-en-1-yl)benzoate (95, $CDCl_3$ as solvent, 376 MHz)

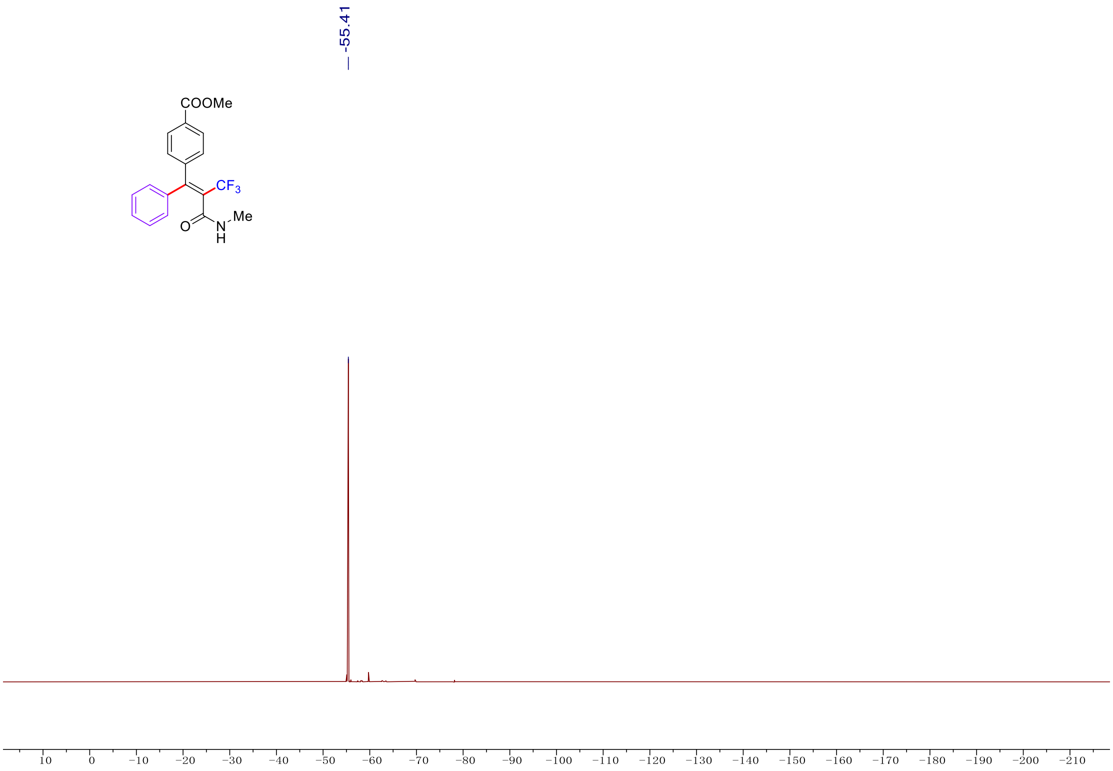

^{1}H NMR Spectrum of (5-(phenylsulfonyl)pent-3-ene-1,4-diyl)dibenzene (97, $CDCl_3$ as solvent, 400 MHz)

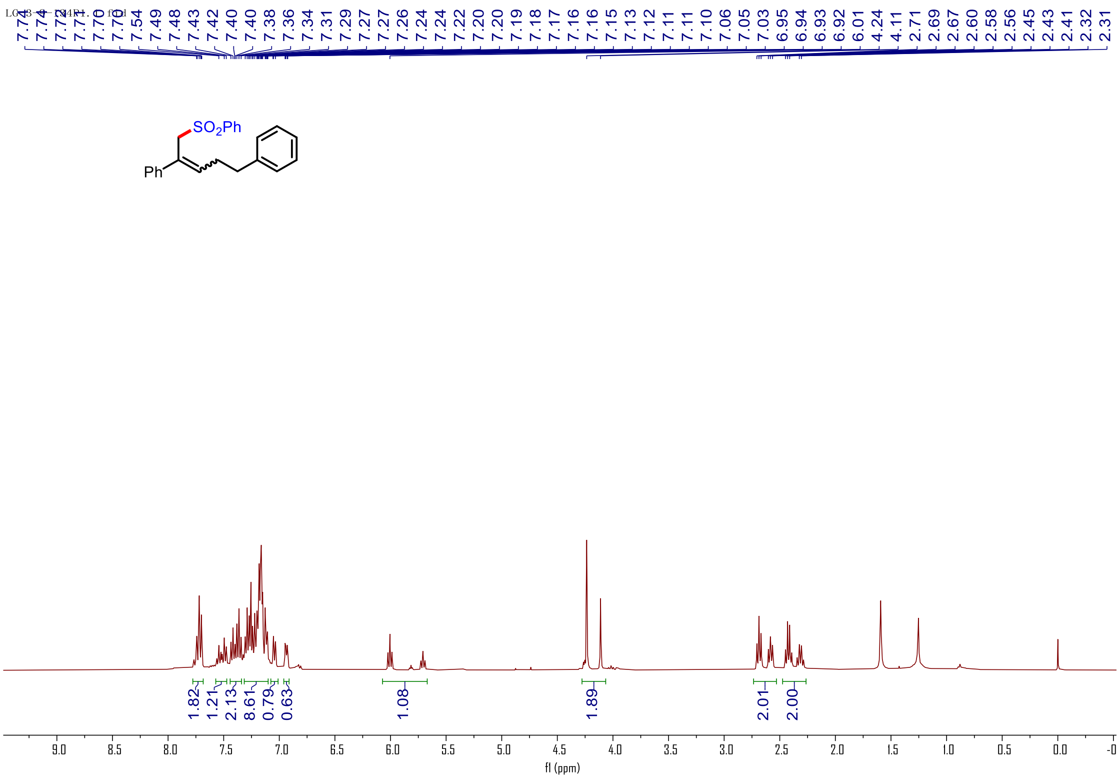

^{13}C {^{1}H} NMR Spectrum of (5-(phenylsulfonyl)pent-3-ene-1,4-diyl)dibenzene (97, $CDCl_3$ as solvent, 101 MHz)

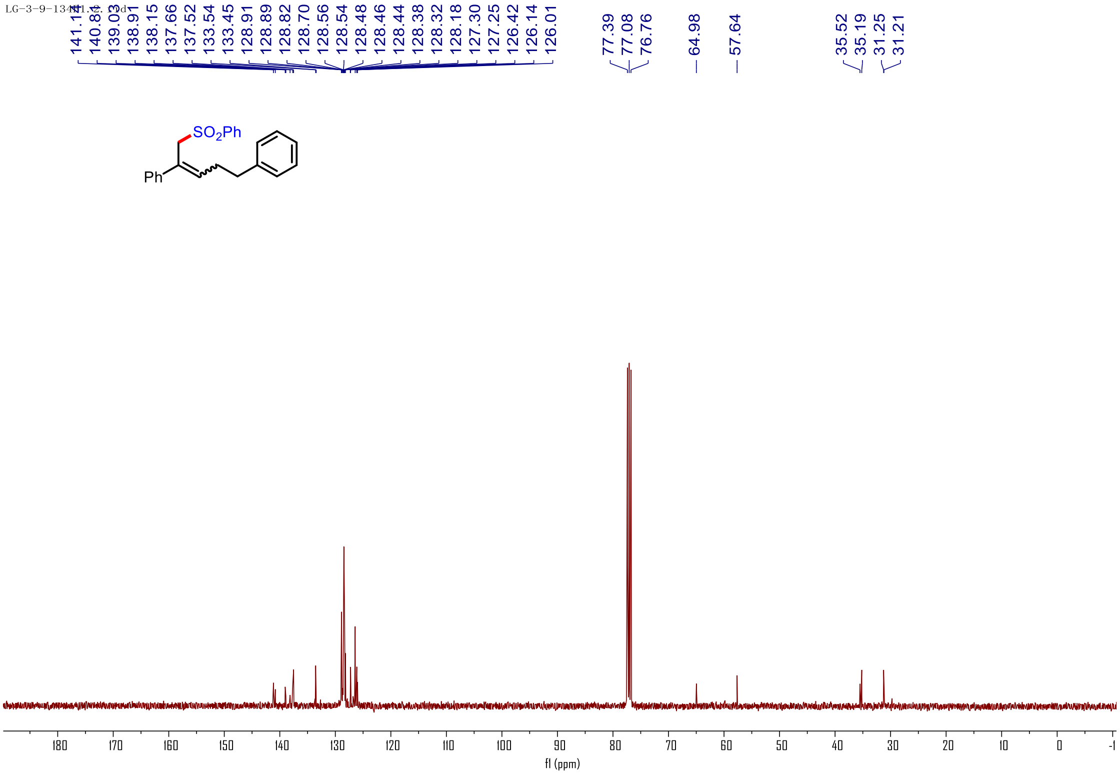

5. X-Ray Crystallographic Spectrum of 3, 4, 12a, *Z*-32, *Z*-70 and *E*-70

5.1 Crystallographic spectrum of product 3 (CCDC number: 2282321)

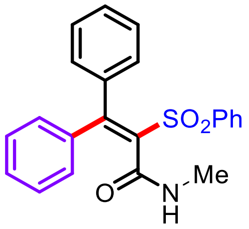

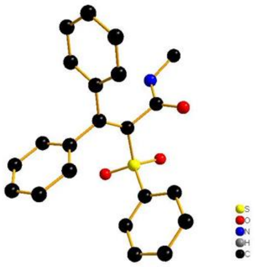

Table S7. Crystal data and structure refinement for LG-3-6-12_auto.

Identification code	lg-3-6-12_auto	
Empirical formula	C22 H19 N O3 S	
Formula weight	377.44	
Temperature	293(2) K	
Wavelength	1.54184 Å	
Crystal system	Monoclinic	
Space group	P 1 21/n 1	
Unit cell dimensions	a = 10.1362(2) Å	a= 90°.
	b = 11.6468(2) Å	b= 94.2210(10)°.
	c = 16.5295(2) Å	g = 90°.
Volume	1946.09(6) $Å^3$	
Z	4	
Density (calculated)	1.288 Mg/m^3	
Absorption coefficient	1.654 mm^{-1}	
F(000)	792	
Crystal size	0.05 x 0.01 x 0.01 mm^3	
Theta range for data collection	4.649 to 76.619°.	
Index ranges	-12<=h<=12, -10<=k<=14, -20<=l<=19	
Reflections collected	12471	
Independent reflections	3911 [R(int) = 0.0428]	
Completeness to theta = 67.684°	99.7 %	
Absorption correction	Semi-empirical from equivalents	
Max. and min. transmission	1.00000 and 0.52982	
Refinement method	Full-matrix least-squares on F^2	
Data / restraints / parameters	3911 / 0 / 245	
Goodness-of-fit on F^2	1.111	
Final R indices [I>2sigma(I)]	R1 = 0.0513, wR2 = 0.1284	
R indices (all data)	R1 = 0.0609, wR2 = 0.1348	
Extinction coefficient	n/a	
Largest diff. peak and hole	0.307 and -0.619 e.$Å^{-3}$	

5.2 Crystallographic spectrum of product 4 (CCDC number: 2282323)

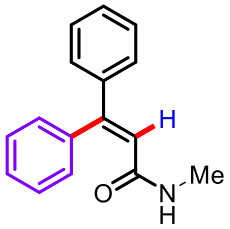

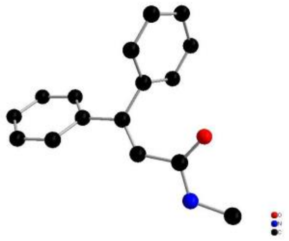

Table S8. Crystal data and structure refinement for 9495.

Identification code	9495	
Empirical formula	C16H15NO	
Formula weight	36.51	
Temperature	153(2) K	
Wavelength	1.54178 Å	
Crystal system	Monoclinic	
Space group	P2(1)/c	
Unit cell dimensions	a = 11.9280(2) Å	a= 90°.
	b = 9.0750(2) Å	b= 91.988(2)°.
	c = 11.8685(2) Å	g = 90°.
Volume	1283.95(4) Å^3	
Z	26	
Density (calculated)	1.228 Mg/m^3	
Absorption coefficient	0.600 mm^{-1}	
F(000)	504	
Crystal size	0.2 x 0.1 x 0.1 mm^3	
Theta range for data collection	6.13 to 74.67°.	
Index ranges	-13<=h<=10, -11<=k<=10, -12<=l<=14	
Reflections collected	4309	
Independent reflections	2148 [R(int) = 0.0175]	
Completeness to theta = 74.67°	81.5 %	
Absorption correction	None	
Max. and min. transmission	1 and 0.92030	
Refinement method	Full-matrix least-squares on F^2	
Data / restraints / parameters	2148 / 0 / 164	
Goodness-of-fit on F^2	1.033	
Final R indices [I>2sigma(I)]	R1 = 0.0348, wR2 = 0.0866	
R indices (all data)	R1 = 0.0369, wR2 = 0.0885	
Largest diff. peak and hole	0.156 and -0.207 e.Å^{-3}	

5.3 Crystallographic spectrum of product 12a (CCDC number: 2380222)

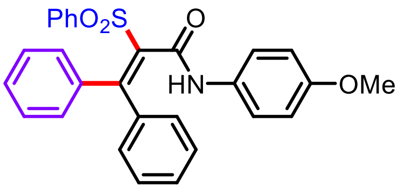

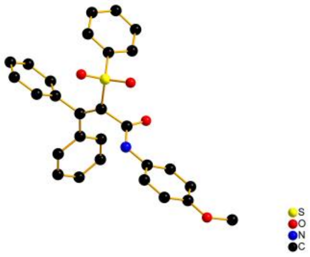

Table S9. Crystal data and structure refinement for gyz-3-7-48_auto.

Identification code	lg-3-7-48_auto	
Empirical formula	C28 H22 N O4 S	
Formula weight	468.52	
Temperature	293(2) K	
Wavelength	1.54184 Å	
Crystal system	Monoclinic	
Space group	P 1 21/n 1	
Unit cell dimensions	a = 10.2861(3) Å	a= 90 °.
	b = 10.2878(2) Å	b= 97.897(2) °.
	c = 22.6964(5) Å	g = 90 °.
Volume	2378.99(10) $Å^3$	
Z	4	
Density (calculated)	1.308 Mg/m^3	
Absorption coefficient	1.495 mm^{-1}	
F(000)	980	
Crystal size	0.9 x 0.7 x 0.5 mm^3	
Theta range for data collection	3.933 to 79.567 °.	
Index ranges	-12<=h<=12, -8<=k<=12, -28<=l<=28	
Reflections collected	15242	
Independent reflections	4887 [R(int) = 0.0240]	
Completeness to theta = 67.684 °	99.6 %	
Absorption correction	Semi-empirical from equivalents	
Max. and min. transmission	1.00000 and 0.83197	
Refinement method	Full-matrix least-squares on F^2	
Data / restraints / parameters	4887 / 0 / 312	
Goodness-of-fit on F^2	1.095	
Final R indices [I>2sigma(I)]	R1 = 0.0458, wR2 = 0.1172	
R indices (all data)	R1 = 0.0599, wR2 = 0.1262	
Extinction coefficient	n/a	
Largest diff. peak and hole	0.153 and -0.360 e.$Å^{-3}$	

5.4 Crystallographic spectrum of product *Z*-32 (CCDC number: 2282339)

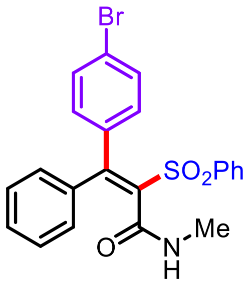

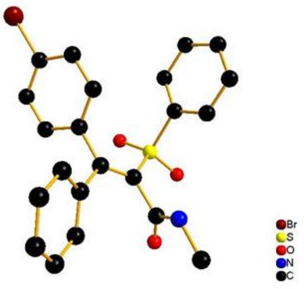

Table S10. Crystal data and structure refinement for LG-3-7-14-1_auto.

Identification code	lg-3-7-14-1_auto	
Empirical formula	C22 H18 Br N O3 S	
Formula weight	456.34	
Temperature	293(2) K	
Wavelength	1.54184 Å	
Crystal system	Monoclinic	
Space group	P 1 21/c 1	
Unit cell dimensions	a = 10.4315(2) Å	a= 90°.
	b = 18.2913(3) Å	b= 97.9060(10)°.
	c = 10.65010(10) Å	g = 90°.
Volume	2012.78(5) $Å^3$	
Z	4	
Density (calculated)	1.506 Mg/m^3	
Absorption coefficient	3.944 mm^{-1}	
F(000)	928	
Crystal size	0.02 x 0.01 x 0.01 mm^3	
Theta range for data collection	4.279 to 74.887°.	
Index ranges	-13<=h<=13, -22<=k<=20, -11<=l<=13	
Reflections collected	12012	
Independent reflections	3984 [R(int) = 0.0264]	
Completeness to theta = 67.684°	99.8 %	
Absorption correction	Semi-empirical from equivalents	
Max. and min. transmission	1.00000 and 0.85814	
Refinement method	Full-matrix least-squares on F^2	
Data / restraints / parameters	3984 / 0 / 254	
Goodness-of-fit on F^2	1.059	
Final R indices [I>2sigma(I)]	R1 = 0.0328, wR2 = 0.0841	
R indices (all data)	R1 = 0.0361, wR2 = 0.0865	
Extinction coefficient	n/a	
Largest diff. peak and hole	0.541 and -0.626 e.$Å^{-3}$	

5.5 Crystallographic spectrum of product *E*-70 (CCDC number: 2283382)

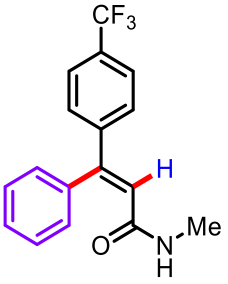

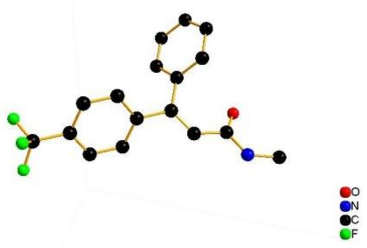

Table S11. Crystal data and structure refinement for LG-3-9-5-1_auto.

Identification code	lg-3-9-5-1_auto	
Empirical formula	C17 H14 F3 N O	
Formula weight	305.29	
Temperature	293(2) K	
Wavelength	1.54184 Å	
Crystal system	Monoclinic	
Space group	I 1 2/a 1	
Unit cell dimensions	a = 9.5082(5) Å	a= 90°.
	b = 15.2885(6) Å	b= 101.919(6)°.
	c = 21.8191(14) Å	g = 90°.
Volume	3103.4(3) $Å^3$	
Z	8	
Density (calculated)	1.307 Mg/m^3	
Absorption coefficient	0.904 mm^{-1}	
F(000)	1264	
Crystal size	0.02 x 0.01 x 0.01 mm^3	
Theta range for data collection	3.556 to 75.373°.	
Index ranges	-11<=h<=11, -14<=k<=18, -26<=l<=27	
Reflections collected	8090	
Independent reflections	3029 [R(int) = 0.0292]	
Completeness to theta = 67.684°	97.7 %	
Absorption correction	Semi-empirical from equivalents	
Max. and min. transmission	1.00000 and 0.61549	
Refinement method	Full-matrix least-squares on F^2	
Data / restraints / parameters	3029 / 36 / 227	
Goodness-of-fit on F^2	1.214	
Final R indices [I>2sigma(I)]	R1 = 0.0900, wR2 = 0.2339	
R indices (all data)	R1 = 0.1215, wR2 = 0.2476	
Extinction coefficient	n/a	
Largest diff. peak and hole	0.208 and -0.206 e.$Å^{-3}$	

5.6 Crystallographic spectrum of product *Z*-70 (CCDC number: 2283384)

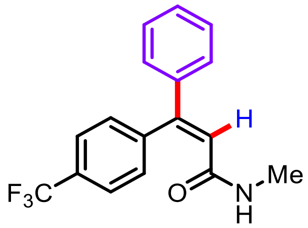

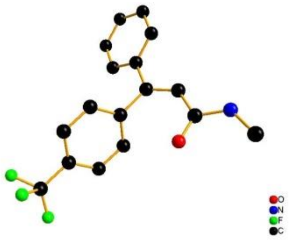

Table S12. Crystal data and structure refinement for LG-3-9-5-2_auto.

Identification code	lg-3-9-5-2_auto	
Empirical formula	C17 H14 F3 N O	
Formula weight	305.29	
Temperature	293(2) K	
Wavelength	1.54184 Å	
Crystal system	Trigonal	
Space group	$P3_2$	
Unit cell dimensions	a = 10.5850(4) Å	a= 90 °.
	b = 10.5850(4) Å	b= 90 °.
	c = 11.6376(4) Å	g = 120 °.
Volume	1129.21(9) $Å^3$	
Z	3	
Density (calculated)	1.347 Mg/m^3	
Absorption coefficient	0.932 mm^{-1}	
F(000)	474	
Crystal size	0.02 x 0.01 x 0.01 mm^3	
Theta range for data collection	4.824 to 76.717 °.	
Index ranges	-13<=h<=11, -12<=k<=9, -14<=l<=14	
Reflections collected	3872	
Independent reflections	2321 [R(int) = 0.0371]	
Completeness to theta = 67.684 °	99.5 %	
Absorption correction	Semi-empirical from equivalents	
Max. and min. transmission	1.00000 and 0.54638	
Refinement method	Full-matrix least-squares on F^2	
Data / restraints / parameters	2321 / 437 / 227	
Goodness-of-fit on F^2	1.057	
Final R indices [I>2sigma(I)]	R1 = 0.0651, wR2 = 0.2349	
R indices (all data)	R1 = 0.0751, wR2 = 0.2429	
Absolute structure parameter	0.0(3)	
Extinction coefficient	n/a	
Largest diff. peak and hole	0.295 and -0.231 e.$Å^{-3}$	

6. References

1. Wang, C.-S., Roisnel, T., Dixneuf, P. H. & Soulé, J.-F. *Adv. Synth. Catal*. **361**, 445–450 (2019).

2. Ghosh, S. & Jana, C. K. *Org. Lett.* **18**, 5788–5791 (2016).

3. Patal, S. & Ikan, R. *J. Org. Chem*. **21**, 1379–1381 (1956).

4. Li, M., Li, S.-X., Chen, D.-p., Gao, F., Qiu, Y.-F., Wang, X.-C., Quan, Z.-J. & Liang, Y.-M. *Org. Lett.* **25**, 2761–2766 (2023).

5. Iyer, P., Matthew L., Villa, C, M., Ryan, C. S. & Robert, J. W. CN101316818B [P], 2011-08-31.

6. Frisch, M. J., Trucks, G. W., Schlegel, H. B., Scuseria, G. E., Robb, M. A., Cheeseman, J. R., Scalmani, G., Barone, V., Petersson, G. A., Nakatsuji, H., Li, X.; Caricato, M., Marenich, A. V., Bloino, J., Janesko, B. G., Gomperts, R., Mennucci, B., Hratchian, H. P., Ortiz, J. V., Izmaylov, A. F., Sonnenberg, J. L., Williams-Young, D., Ding, F., Lipparini, F., Egidi, F., Goings, J., Peng, B., Petrone, A., Henderson, T., Ranasinghe, D., Zakrzewski, V. G., Gao, J., Rega, N., Zheng, G., Liang, W., Hada, M., Ehara, M., Toyota, K., Fukuda, R., Hasegawa, J., Ishida, M., Nakajima, T., Honda, Y., Kitao, O., Nakai, H., Vreven, T., Throssell, K., Montgomery, J. A., Jr., Peralta, J. E., Ogliaro, F., Bearpark, M. J., Heyd, J. J., Brothers, E. N., Kudin, K. N., Staroverov, V. N., Keith, T. A., Kobayashi, R., Normand, J., Raghavachari, K., Rendell, A. P., Burant, J. C., Iyengar, S. S., Tomasi, J., Cossi, M., Millam, J. M., Klene, M., Adamo, C., Cammi, R., Ochterski, J. W., Martin, R. L., Morokuma, K., Farkas, O., Foresman, J. B., Fox, D. J. Gaussian Inc 16, Revision B.01. Gaussian Inc., Wallingford CT. 2016.

7. Becke, A. D. *Phys. Rev. A* **38**, 3098−3100 (1988).

8. Becke, A. D. *J. Chem. Phys.* **98**, 5648−5652 (1993).

9. Lee, C. Yang, W. & Parr, R. G. *Phys. Rev. B: Condens. Matter Mater. Phys.* **37**, 785−789 (1988).

10. Becke, A. D. *J. Chem. Phys.* **98**, 1372−1377 (1993).

11. Weigend, F. Furche, F. & Ahlrichs, R. *J. Chem. Phys.* **119**, 12753−12762 (2003).

12. Grimme, S. Antony, J. Ehrlich, S. & Krieg, H. *J. Chem. Phys.* **132,** 154104 (2010).

13. Legault, C. Y. *CYLview: A Visualizationand Analysis Software for Computational Chemistry, 1.0b*; Université de Sherbrooke, 2009a, http://www.cylview.org.

14. Roth, H. G., Romero, N. A. & Nicewicz, D. A. *Synlett* **27**, 714–723 (2016).

15. Isse, A. A. & Gennaro, A. *J. Phys. Chem. B* **114**, 7894–7899 (2010).
